# Catalytic alkoxysilylation of C–H bonds with *tert*-butyl-substituted alkoxysilyldiazenes†

**DOI:** 10.1039/d5sc02059j

**Published:** 2025-04-24

**Authors:** Lamine Saadi, Loïc Valade, Clément Chauvier

**Affiliations:** a Sorbonne Université, CNRS, Institut Parisien de Chimie Moléculaire 4 Place Jussieu 75005 Paris France clement.chauvier@sorbonne-universite.fr

## Abstract

Organoalkoxysilanes (*e.g.* R–SiMe_3−*n*_(OR′)_*n*_, 1 ≤ *n* ≤ 3 with R = alkyl or aryl) have found various applications in synthetic chemistry and materials science because the silicon-bound alkoxy groups provide unique opportunities for further derivatization and transformations. Among the few catalytic strategies that allow the direct and intermolecular introduction of an alkoxysilyl unit onto an organic substrate, the alkoxysilylation of unactivated C–H bonds has barely been achieved despite its synthetic potential and the atom-economy it conveys. In particular, a catalytic and transition metal-free C–H silylation protocol towards this class of organosilicon compounds has yet to be reported. We herein describe the first general alkoxysilylation of (hetero)arene C(sp^2^)–H and benzylic C(sp^3^)–H bonds under ambient, transition metal-free conditions using newly-prepared *tert*-butyl-substituted alkoxysilyldiazenes (*t*Bu–N=N–SiMe_3−*n*_(OR′)_*n*_, 1 ≤ *n* ≤ 3 with R′ = Et, iPr or *t*Bu) as silylating reagents and *t*BuOK as catalytic promoter.

## Abstract 2

This work introduces the synthesis and use of novel *tert*-butyl-substituted alkoxysilyldiazenes (*t*Bu–N=N–SiMe_3−*n*_(OR′)_*n*_, 1 ≤ *n* ≤ 3 with R′ = alkyl) to expedite the assembly of various organoalkoxysilanes under catalytic transition metal-free conditions.

## Graphical abstract

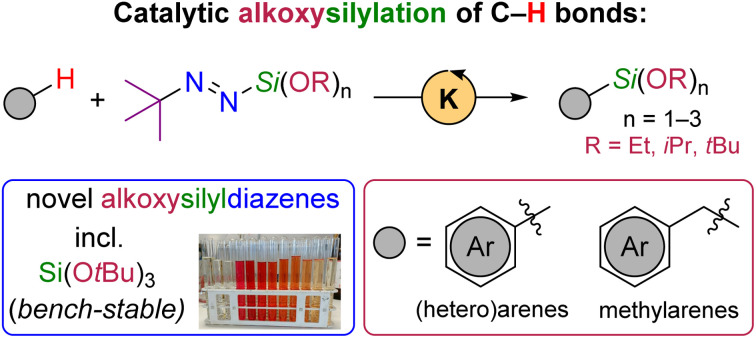

## Introduction

**Scheme 1:**
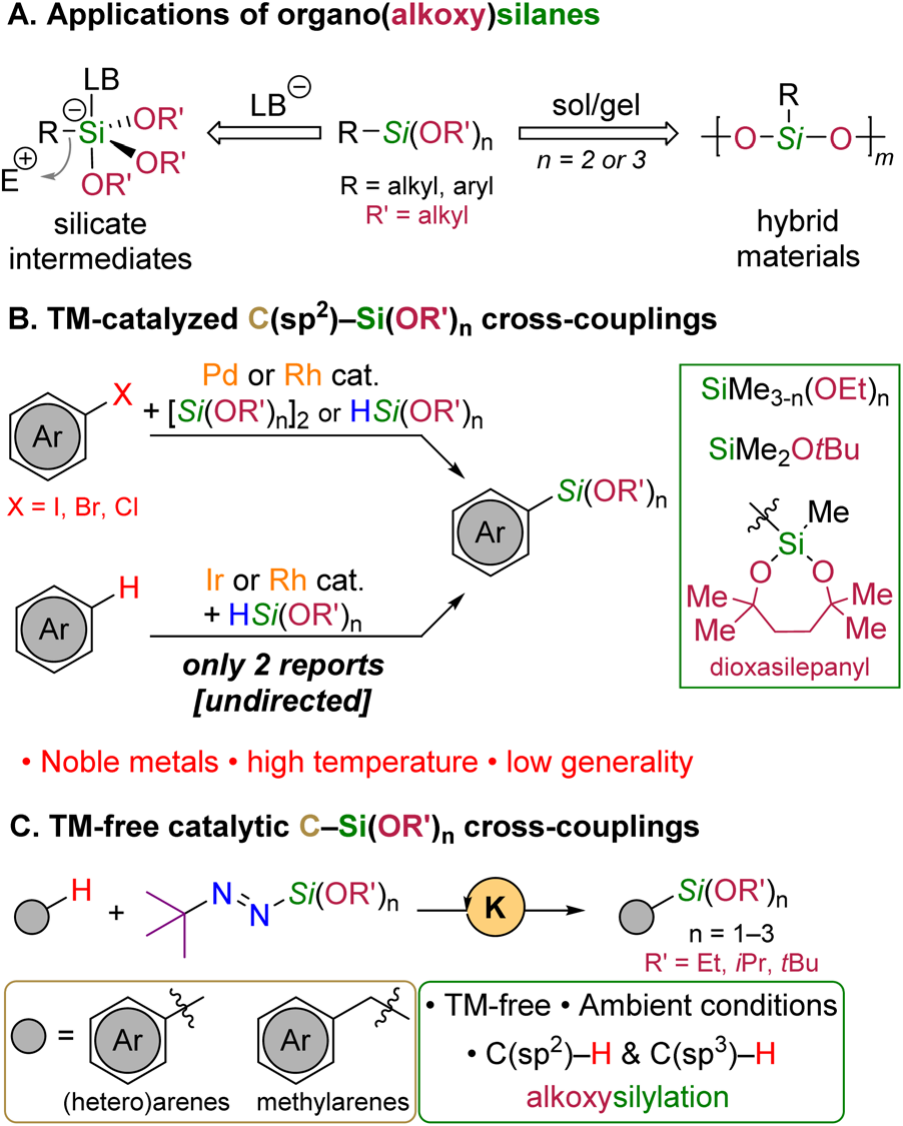
Applications and catalytic synthesis of organo(alkoxy)silanes.

Organosilicon chemistry plays a central role in organic synthesis and materials science because silicon is earth-abundant and displays peculiar properties, including a relatively low electronegativity and a high oxophilicity. Among the myriad of organosilicon compounds available for synthesis, those combining a functionalized organic moiety (“R”) bound to an alkoxysilyl group (*e.g.*, R–SiMe_3−*n*_(OR′)_*n*_, 1 ≤ *n* ≤ 3 with R′ = alkyl) are particularly attractive because they display an application potential (Scheme 1A) much greater than the corresponding triorganosilyl derivatives (*e.g.*, R–SiMe_3_)^1^ while being more stable than the corresponding halosilane derivatives (*e.g.*, R–SiCl_3_). On the one hand, the alkoxy groups can be readily displaced in hydrolytic or non-hydrolytic sol/gel processes to yield functionalized polysiloxane (*n* = 2) or polysilsesquioxane (*n* = 3; R–SiO_1.5_) materials. On the other hand, the electron-withdrawing alkoxy groups facilitate the formation of silicate intermediates that are key species for example in the Hiyama cross-coupling reaction,^2^ the Tamao–Fleming oxidation^3^ or other oxidative processes.^4^

To date, the direct introduction of alkoxysilyl groups onto organic substrates has primarily been achieved through two main strategies: (1) the catalytic hydrosilylation of alkenes or alkynes with alkoxysilanes (*e.g.*, HSi(OEt)_3_)^5^ and (2) the stoichiometric coupling of organolithium or Grignard reagents with chloro(alkoxy)silanes or orthosilicates (Si(OR′)_4_). Because the hydrosilylation approach is effective only for synthesizing alkyl- and vinylsilane derivatives, the stoichiometric route remains indispensable for preparing aryl- and primary benzylalkoxysilanes.^6^ However, the latter methods suffer from several drawbacks, including low tolerance for electrophilic functional groups and the need for cryogenic temperatures to control reactivity and selectivity. Furthermore, they generate substantial amounts of metallic waste, posing significant challenges for large-scale applications.

In recent years, transition metal-catalyzed C–Si cross-couplings have been reported to introduce mono-,^7^ bis-^8^ and trisalkoxy^9^ as well as siloxy^10^ units onto aromatic scaffolds directly from aryl halides and the corresponding hydrosilane or disilane derivatives (Scheme 1B). However, not only are those approaches based on expensive noble metals (Pd or Rh), but they also rely on pre-functionalized halide substrates, selective preparation of which can be difficult and reduces the overall atom economy of the silylation process.

For these reasons, the direct intermolecular silylation of C–H bonds has emerged as a privileged strategy to forge C(sp^2^)– or C(sp^3^)–Si bonds in a catalytic manner.^11^ While this field has substantially evolved over the past decade with useful solutions proposed to overcome both reactivity and site-selectivity issues,^12^ the general introduction of alkoxysilyl units rather than trialkyl or siloxy-based (SiMe(OSiMe_3_)_2_) ones onto hydrocarbon substrates has barely been achieved. As far as arylsilanes are concerned, only two such reports have been described with hydrosilanes as silicon sources (Scheme 1B). Lee and coworkers showed that a rhodium-based complex catalyzes the undirected silylation of unactivated (hetero)arenes with HSiMe_2_OEt as the alkoxysilyl source.^13^ This catalytic system, however, proved inefficient to promote the transfer of alkoxysilyl groups bearing more than one ethoxy unit and the silylated arenes are mostly produced as mixtures of regioisomers. In 2021, Yorimitsu and Shimokawa showed that Hartwig's catalytic dehydrogenative silylation protocol could be modified to introduce the dioxasilepanyl unit, a hydrolytically-stable 7-membered cyclic bisalkoxysilyl group.^14^ Nevertheless, this iridium-catalysed transformation, initially cantoned to the exclusive transfer of the bis-siloxy SiMe(OSiMe_3_)_2_ unit,^15^ could not be further extended to acyclic alkoxysilyl groups derived from common alcohols.

Beyond the alkoxysilylated arenes, benzylsilanes bearing alkoxysilyl units are also promising monomers in silicone science,^16^ which have barely been explored because of the lack of general synthesis. In particular, the catalytic and undirected formation of benzyl(alkoxy)silanes by dehydrogenative silylation of toluene derivatives with HSi(OEt)_3_ has only been reported to occur with heterogeneous metal-based catalysts at high temperature.^17^

From these considerations, we have sought to develop a general method towards the formation of aryl- and benzyl(alkoxy)silanes through the silylation of unactivated (hetero)arene C(sp^2^)–H bonds and benzylic C(sp^3^)–H bonds. To that end, we have recently reported that potent metalating agents can be generated catalytically when *tert*-butyl-substituted silyl diazenes (*t*Bu–N=N–SiR_3_) are treated with a catalytic amount of a potassium salt (typically *t*BuOK). This process has been fruitfully exploited to introduce trialkylsilyl (*e.g.*, SiMe_3_ and SiEt_3_) groups through the catalytic silylation of unactivated C(sp^2^)–H^18^ and C(sp^3^)–H bonds^19^*via* metalation/silylation sequences. We herein demonstrate that such a reactivity manifold can now be applied to the catalytic introduction of alkoxysilyl units from newly-prepared *tert*-butyl-substituted alkoxysilyl diazenes under ambient and transition metal-free conditions (Scheme 1C).

## Results and discussion

**Scheme 2:**
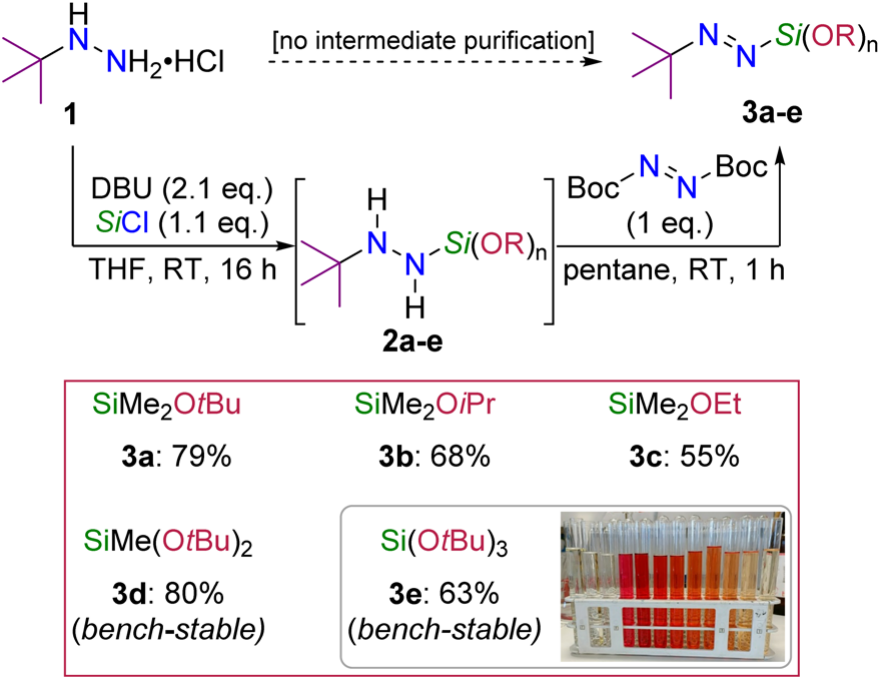
Synthesis of *tert*-butyl-substituted alkoxysilyldiazenes. Yields refer to those of isolated products over two steps (1 → 3a–e).

We initiated our study with the preparation of various *tert*-butyl-substituted alkoxysilyldiazenes (*t*Bu–N=N–Si(OR)_*n*_) by varying the nature (R = Et, iPr and *t*Bu) and/or the number (*n* = 1, 2 or 3) of alkoxy units bound to the silicon atom. To that end, our previously reported protocol starting from commercially-available *tert*-butylhydrazine hydrochloride 1 could largely be used with only marginal modification of the conditions needed in some cases (see the ESI† for details), further demonstrating the robustness of this process.^18,20^ Typically, 1 was mixed with a chloro(alkoxy)silane Cl–SiMe_3−*n*_(OR)_*n*_ (*n* = 1, 2 or 3) under basic conditions to afford the corresponding (alkoxy)silylated hydrazines 2a–e arising from the selective replacement of the chloride by the hydrazide moiety (Scheme 2). The subsequent dehydrogenative oxidation of the non-purified crude hydrazine with DBAD (di-*tert*-butyl azodicarboxylate)^21^ produced the desired red-colored alkoxysilyldiazenes 3a–e bearing one (3a–3c), two (3d) or three (3e) alkoxy units in 55–80% yields over two steps. Importantly, the bulkiest bis- and tris-*tert*-butoxy derivatives 3d and 3e were found bench-stable and could be easily purified by column chromatography on deactivated (NEt_3_) silica gel. In all instances, the *tert*-butyl-substituted alkoxysilyldiazenes 3a–e were found stable towards the intermolecular redistribution of the alkoxy/diazenyl groups (*e.g.* 2 *t*Bu–N=N–SiMe_2_(OR) → (*t*Bu–N=N)_2_–SiMe_2_ + SiMe_2_(OR)_2_) upon storage, though the least bulky congener 3c slowly decomposed over a period of months when kept at room temperature in a glovebox, presumably through such a redistribution pathway.

With these new silyldiazenes in hands, we first studied the transfer of the mono-alkoxysilyl units SiMe_2_OR from diazenes 3a–c to *N*-methylindole (4) through the catalytic deprotonative silylation of the weakly acidic C(sp^2^)–H bond at the 2-position (Table 1, entries 1–8). To that end, the reaction parameters were first optimized with the bulkiest congener 3a (see Table S1 in the ESI† for details). Using *t*BuOK (10 mol%) as an inexpensive catalytic promoter and a slight excess of 3a (1.8 eq.) in THF, 4 was rapidly and cleanly converted to the corresponding silylated indole 4a, which was isolated in 91% yield after 1 h of reaction at room temperature (entry 1, Table 1). Careful analysis by NMR spectroscopy and GC-MS of the crude reaction mixture prior to purification revealed that the remaining mass balance was mainly accounted for by the presence of the chromatography-separable bis-indole product 5 (*ca.* 7%), formulation of which was eventually confirmed by single-crystal X-ray diffraction (see bottom of Table 1). 5 formally arises from a substitution of the *tert*-butoxy group in 4a by a second 2-indolyl moiety.

**Table 1:** Optimization of the C(sp^3^)–H silylation of 4 with diazene 3a^a^

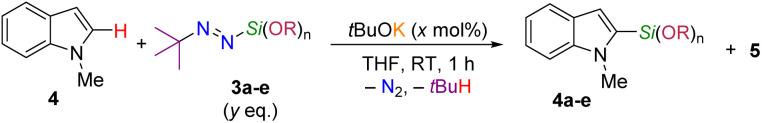					
Entry	*x*	Si(OR)_*n*_ [*y*]	*ρ* [%]	Yield 4a–e [%]	Yield 5 [%]
1	10	3a SiMe_2_O*t*Bu [1.8]	>95	88 (91)	7
2	10	3b SiMe_2_OiPr [1.8]	70	51	9
3	10	3c SiMe_2_OEt [1.8]	65	42	10
4	10	3b SiMe_2_OiPr [3.0]	86	73 (49)	7
5	10	3c SiMe_2_OEt [3.0]	69	63	4
6	50	3a SiMe_2_O*t*Bu [2.5]	91	73	3
7	50	3b SiMe_2_OiPr [2.5]	73	7	40
8	50	3c SiMe_2_OEt [2.5]	73	3	50 (48)
9^b^	10	3d SiMe(O*t*Bu)_2_ [2.2]	—	(99)	n.a.
10^c^	40	3e Si(O*t*Bu)_3_ [2.2]	75	68 (46)	n.a.
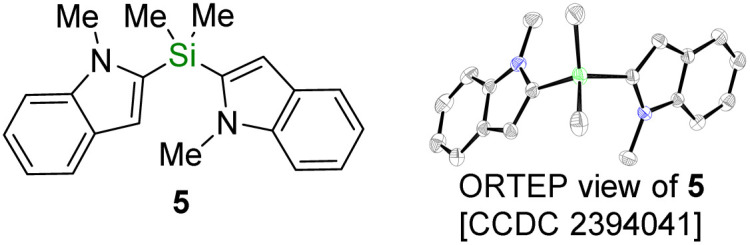					

a Conversion (*ρ*) and yields were determined by ^1^H NMR on the crude reaction mixture with 1,3,5-trimethoxybenzene as internal standard. Isolated yields in brackets. n.a.: not applicable.

b 16 h.

c 24 h.

Under otherwise identical conditions, the less bulky isopropoxy and ethoxy diazenes 3b and 3c reacted more sluggishly as the conversion plateaued at 70% and 65% and the corresponding indole products 4b and 4c were produced in 51% and 42% yield along with 9% and 10% of 5, respectively (entries 2–3). In both cases, the diazenes 3b and 3c were fully consumed prior to reaching full conversion of 4, thereby indicating that a non-productive decomposition occurred in the presence of *t*BuOK. Accordingly, in the absence of 4, 3b and 3c rapidly reacted (<5 min) with *t*BuOK (10 mol%) to afford a range of silylated products, among which silylated di-*tert*-butylhydrazines were produced predominantly (see Fig. S5 in the ESI†). In line with these findings, higher yields of 4b and 4c (73% and 63%) were obtained when 4 was treated with increased amounts of 3b and 3c (3 eq.) to compensate for their non-productive self-degradation (entries 4–5). Taken together, these results indicate that the C(sp^2^)–H silylation reaction benefits from an increased steric bulk of the alkoxide residue, primarily because of the greater stability of the corresponding diazene towards its self-decomposition. In addition, the formation of the doubly-substituted product 5 remains marginal with diazene 3a, while it becomes the major product at higher loading of *t*BuOK with 3b and 3c (entries 6–8).

In contrast to the mono-alkoxysilyldiazenes 3a–c, the bench-stable bis- and tris-*tert*-butoxysilyl derivatives 3d and 3e were found highly resistant to base-catalyzed self-decomposition and barely prone to over-substitution. As an illustration, the product 4e was isolated in quantitative yield after 16 h of reaction and even the extremely bulky Si(O*t*Bu)_3_ unit could be transferred in 68% yield after *ca.* 24 h of reaction (75% conversion) with 40 mol% *t*BuOK (entries 9–10). Importantly, the latter result constitutes the first example of a synthesis of an aromatic trialkoxysilane by catalytic silylation of a C(sp^2^)–H bond.

**Scheme 3:**
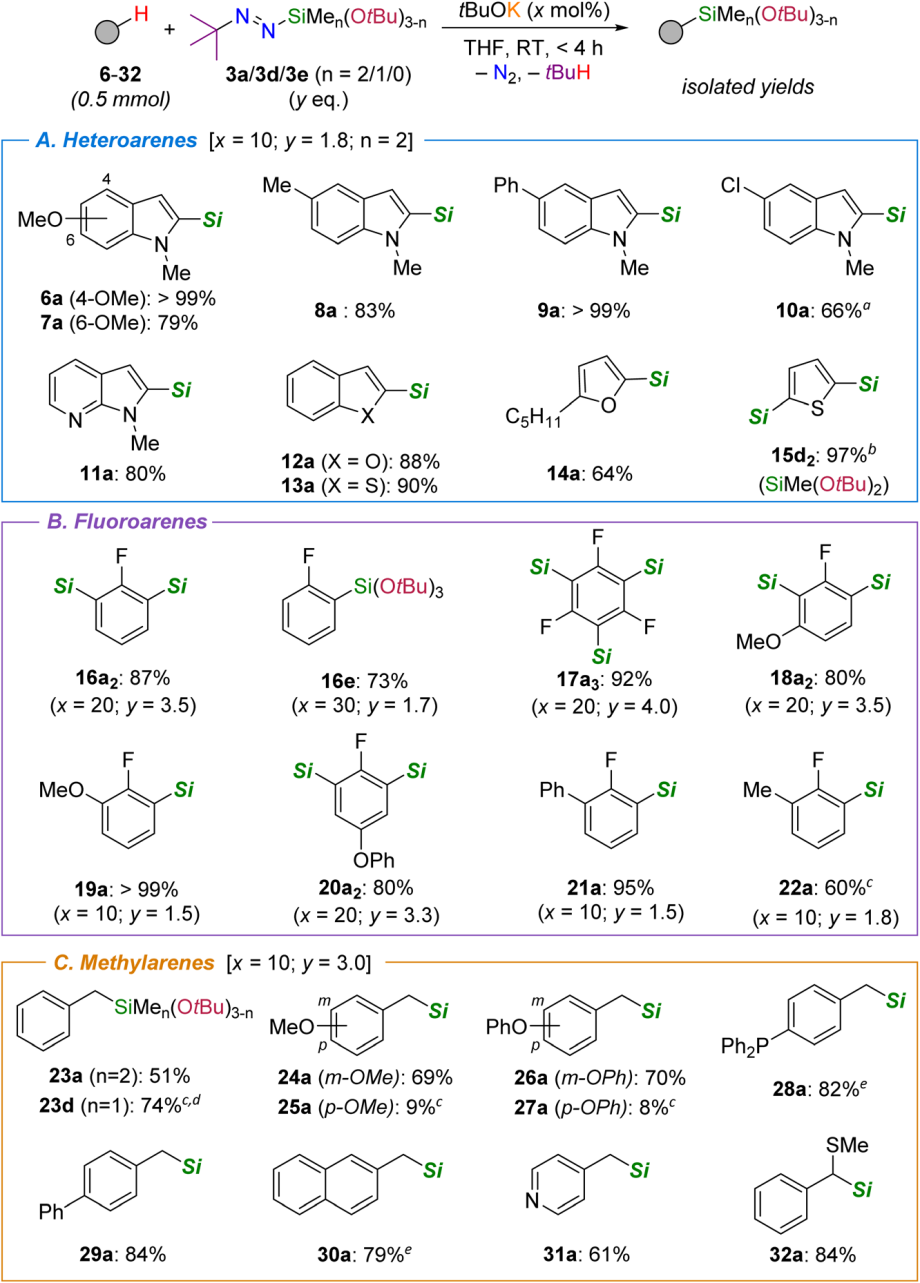
Substrate scope of the silylation reaction (***Si*** = SiMe_2_O*t*Bu from diazene 3a, unless otherwise indicated). Yields refer to those of isolated product after purification by flash chromatography.^*a*^3a (2 eq.), *t*BuOK (20 mol%).^*b*^3d (3.2 eq.).^*c*^ Yield determined by NMR spectroscopy.^*d*^ 3d (3 eq.),^*e*^*t*BuOK (20 mol%).

We next turned our attention to the substrate scope of the silylation reaction (Scheme 3), mostly using diazene 3a as the silicon source not only because it performs better than its less bulky congeners 3b and 3c, but also because the SiMe_2_O*t*Bu unit is stable towards protonolysis on silica gel, thereby simplifying product purification and isolation. At the outset, various indoles bearing electron-donating (6–8) or withdrawing groups (9–10) on the arene core could be silylated in good to excellent yields under the conditions optimized with 4 (10 mol% *t*BuOK, 1.8 eq. 3a). In all instances the silylation occurred selectively at the 2-position even when potentially directing methoxy (in 6–7) and chloride (in 10) groups that acidify proximate hydrogen atoms are present. Likewise, the silylation of the azaindole derivative 11 cleanly delivered 11a in 80% isolated yield. Beyond the indole core, other heteroarenes were also efficiently silylated, including furan (12 & 14) or thiophene (13 & 15) derivatives. Importantly, the bis-silylation of thiophene 15 could easily be achieved with diazene 3d (3.2 eq.) to give 15d_2_ in excellent yield, a bifunctional organosilane that may find applications as monomer in silicone science.

Simple fluoroarenes were also competent substrates (Scheme 3B). As an illustration, one of the *ortho* C–H bonds in fluorobenzene 16, which displays a similar acidity than the one at the 2-position of *N*-methylindole 4 (p*K*a ≈ 37 in DMSO),^22^ was readily silylated with *t*BuOK (10 mol%) and 3a (1.2 eq.) to afford the corresponding SiMe_2_O*t*Bu-substituted arene 16a in 67% yield (not shown). However, under these conditions, the latter was produced along with 24% of the bis-silylated arene 16a_2_ as judged by ^19^F NMR spectroscopy, which could be isolated in 87% yield when an excess of 3a (3.5 eq.) was employed. In fact, we had previously shown that the selectivity for mono- *vs.* bis-silylation pathways with trialkylsilyl-substituted diazenes can be controlled by tuning the steric hindrance of the silyl group (*e.g.* Me_3_Si *vs. t*BuMe_2_Si).^18^ A similar dichotomy was observed with alkoxysilyl-substituted diazenes (see Table S6 in the ESI†). For example, using diazene 3e as the silicon source, only one bulky Si(O*t*Bu)_3_ unit could be selectively introduced in product 16e, which was further isolated in 73% yield. In fact, the present silylation methodology is particularly efficient to assemble polysilylated compounds as witnessed by the per-silylation of 1,3,5-trifluorobenzene (17), which was readily converted into 17a_3_ (92% yield) with a slight excess of diazene 3a (4 eq.). Interestingly, this type of trifunctional arylsilane product has been employed as precursor of siloxane-based cyclophanes.^23^ The superb site selectivity for positions *ortho* to fluorine atom(s) was also retained for substrates bearing potentially competing methoxy (18–19) or phenoxy (20) directing groups as well as aryl (21) or methyl (22) substituents. In all instances, the corresponding mono- or bis-silylated products were isolated in high yields (>80%), though 22a was accompanied by 16% of the bis-silylated product 22a_2_, in which both the C(sp^2^)–H bond ortho to fluorine and one benzylic C(sp^3^)–H bond have been silylated.

Capitalizing on this observation as well as on our previous work that dealt with the undirected silylation of benzylic C(sp^3^)–H bonds,^19*b*^ the lateral silylation of various methylarenes was investigated (Scheme 3C). When toluene (23) was reacted with diazene 3a (3.0 eq.) in the presence of *t*BuOK (10 mol%), the corresponding benzylsilane 23a could be isolated in 51% yield after 4 h of reaction. With the bulkier diazene 3d, however, the SiMe(O*t*Bu)_2_ unit could be transferred to 23 in an improved 74% yield, thereby suggesting that increased steric hindrance (and Lewis acidity of the silicon center) minimizes pathways that shut down the catalytic activity. Nonetheless, other methylarenes bearing methoxy (24), phenoxy (26), diphenylphosphino (28) or phenyl (29) substituents reacted smoothly with 3a to afford the corresponding silylated products in good to excellent yields. In the cases of 24a and 26a, regioisomers arising from the silylation *ortho* to the MeO or PhO groups were barely observed, thus confirming that the high lateral selectivity we had previously demonstrated with Et_3_Si-substituted diazene (*t*Bu–N=N–SiEt_3_) also holds with 3a. However, we have found one major reactivity difference between 3a and *t*Bu–N=N–SiEt_3_ when substrates bearing (strongly) electron-donating groups at the para position are employed. As an illustration, while the silylation of 4-methylanisole 25 proceeds smoothly with *t*Bu–N=N–SiEt_3_ (84% isolated yield), only a small amount of 25a was formed with diazene 3a (<10% yield) under otherwise identical or more forcing conditions. A similar outcome was obtained with methylarenes bearing 4-OPh (27) or 4-NMe_2_ (not shown) substituents. Because meta-substituted isomers 24 and 26 are competent substrates, we ascribe the lack of reactivity of electron rich substrates to the relatively low acidity of the benzylic C(sp^3^)–H bonds, deprotonation of which would be potentially slower than side reactions, especially diazene self-decomposition. Despite these limitations, less aromatic scaffolds such as the naphthyl or pyridyl derivatives 30 and 31 as well as the secondary benzylic thioether 32 were also tolerated.

**Scheme 4:**
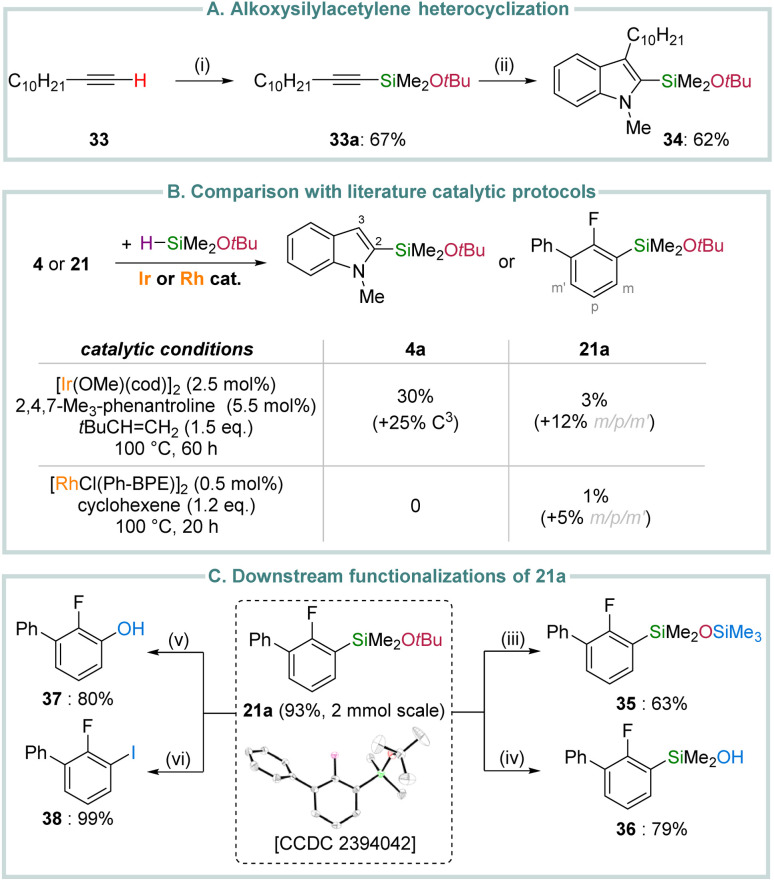
Applications and interest of the methodology. (i): KOH (10 mol%), 3a (1.5 eq.), RT, 3 h. (ii): Pd(OAc)_2_ (5 mol%), PPh_3_ (5 mol%), 2-iodo-*N*-methylaniline (1 eq.), 37a (2 eq.), LiCl (1 eq.), K_2_CO_3_ (5 eq.), DMF, 100 °C, 5 h. (iii): BiCl_3_ (5 mol%), Me_3_SiCl (3 eq.), MeCN : pentane (3 : 1), RT, 3 h. (iv): HCl_(aq)_ (4 mol%), MeCN, RT, 10 min. (v): *t*BuOOH (2 eq.), *t*BuOK (2.2 eq.), THF, 60 °C, 16 h. (vi): ICl (3 eq.), CH_2_Cl_2_, RT, 16 h.

Beyond C(sp^2^)–H or C(sp^3^)–H bonds, our alkoxysilylation protocol can also be applied to the silylation of more acidic C(sp)–H bonds.^24^ As an illustration, 1-dodecyne (33) smoothly reacted at room temperature with diazene 3a (1.5 eq.) upon simple treatment with KOH (10 mol%) rather than *t*BuOK, affording the silylated acetylene 33a in 67% isolated yield (Scheme 4A). Interestingly, 33a can be subjected to a Pd-catalysed Larock heterocyclization with 2-iodo-*N*-methylaniline according to a protocol developed by Denmark and Baird.^25^ Such a route gives access to the 3-substituted 2-alkoxysilylated indole product 34 (62% yield), which can be further derivatized by Hiyama–Denmark cross-couplings.^25^

Overall, the present catalytic methodology exhibits unique generality to assemble a range of structurally-diverse organosilanes bearing different alkoxysilyl groups from readily available hydrocarbon substrates. In particular, it provides a direct entry to (hetero)aryl(*tert*-butoxy)silanes, a class of compounds that remains largely inaccessible through state-of-the-art C(sp^2^)–H alkoxysilylation protocols. To illustrate this further, we reacted the model substrates 4 and 21 with HSiMe_2_O*t*Bu under Rh-^13^ or Ir-based^14*a*^ catalytic conditions (Scheme 4B and Section 3 in the ESI† for details), which have been previously developed to introduce alkoxysilyl units by (undirected) C(sp^2^)–H bond dehydrogenative silylation. Under such conditions, the desired products 4a and 21a were either not formed (Rh) or produced in low yields (Ir), despite the rather forcing conditions (100 °C), long reaction times (20–60 h) and the presence of hydrogen acceptors as additives. In addition, the Ir-based system displayed only moderate site-selectivity, as both 4a and 21a were accompanied by other regioisomers as well as bis-silylated products, as indicated by GC/MS analysis. In stark contrast, the potassium-catalysed silylation of 4 and 21 with 3a proceeds efficiently at room temperature with superb site-selectivity.

The limited availability of catalytic methods to access (hetero)aryl(*tert*-butoxy)silanes has resulted in a scarcity of data regarding their potential applications.^7*b*^ This is particularly noteworthy given their bench stability and thus ease of handling compared to the more prevalent methoxy- or ethoxysilyl derivatives.^26^ We thus embarked into an evaluation of the application potential of the fluorobiphenyl product 21a (Scheme 4C), which was prepared on a 2 mmol scale in 93% isolated yield (564 mg), further demonstrating the scalability of the methodology. Adopting a silicon chemistry point of view, we first demonstrated that despite its bulkiness, the *t*BuO residue can readily be replaced by other functional groups. For example, 21a smoothly underwent a bismuth-catalyzed cleavage of the O–C(CH_3_)_3_ in the presence of chlorotrimethylsilane to afford the heteroleptic disiloxane 35 in 63% yield.^27^ Alternatively, controlled hydrolysis of the O–Si bond in 21a led to the silanol 36.^25^ Finally, oxidative cleavage of the C(sp^2^)–Si bond could be achieved to assemble the functionalized phenol 37 and iodoarene 38 in good to excellent yields.

**Scheme 5:**
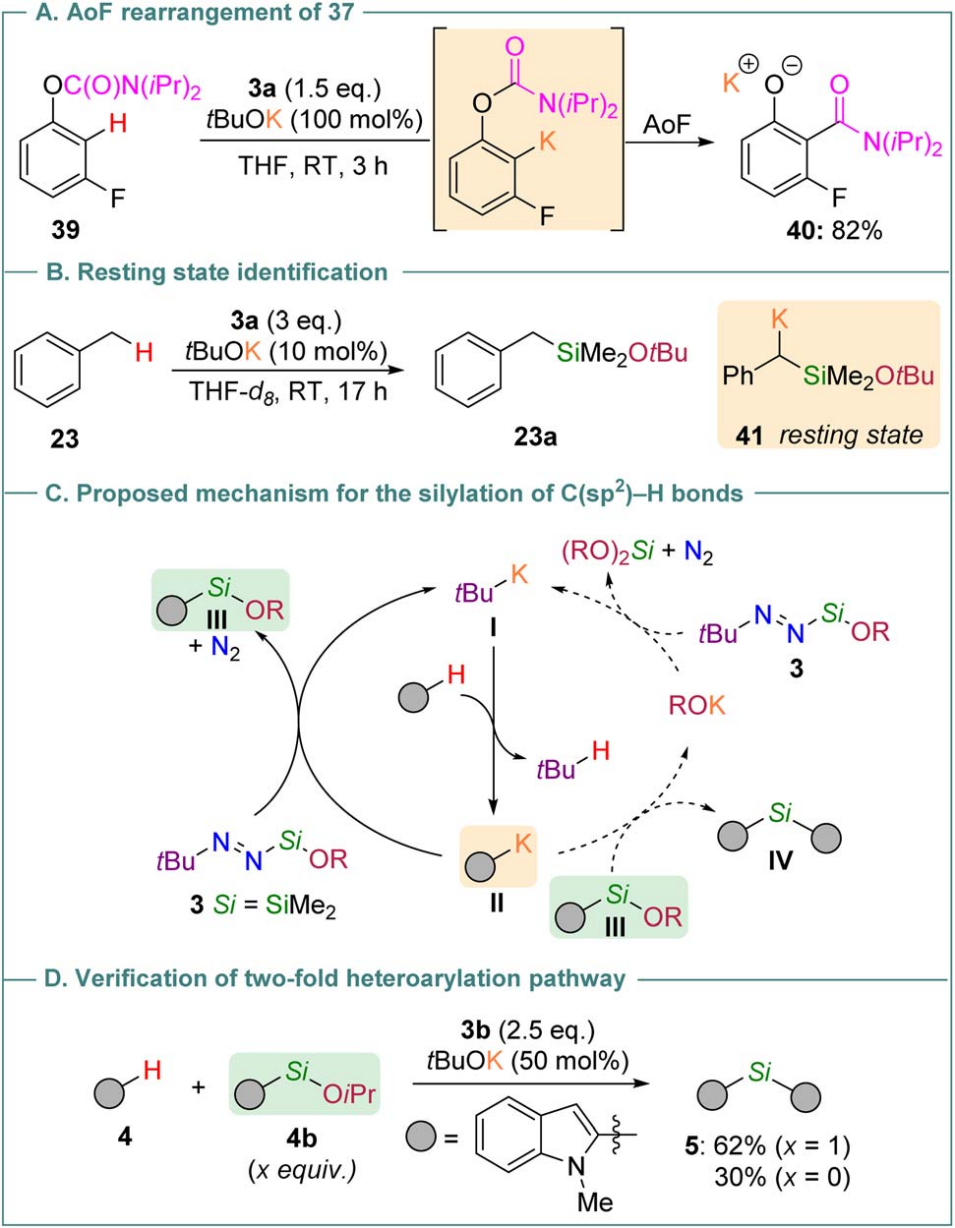
Mechanistic investigations.

To end this study, we sought to establish that the potassium-catalyzed C(sp^2^)–H and C(sp^3^)–H (alkoxy)silylation reactions proceed according to anionic mechanistic scenarios similar to those previously established with *tert*-butyl-substituted trialkylsilyldiazenes.^18,19,28^ To that end, we first verified that under the typical C(sp^2^)–H silylation conditions, the *O*-fluoroaryl carbamate 39 undergoes an anionic *ortho*-Fries (AoF) rearrangement diagnostic of the involvement of aryl potassium intermediates. When 39 was reacted with 3a (1.5 eq.) and a stoichiometric amount of *t*BuOK (100 mol%), the rearranged potassium phenoxide product 40 was formed in almost quantitative yield (Scheme 5A). Under catalytic conditions, such a rearrangement was also operative as witnessed by the formation of the corresponding *O*-silylated benzamide (*ca.* 7% yield after 16 h, see Table S11 in the ESI†), which presumably arises from the silylation of the hydroxide of 40 in the presence of 3a. Importantly, no reaction took place between 39 and *t*BuOK (1 eq.) in the absence of 3a, thereby indicating that the diazene decomposition is pivotal to induce the metalation and the subsequent rearrangement of 39. In a similar vein, the alkoxysilylation of benzylic C(sp^3^)–H bonds follows an anionic mechanism as confirmed by the direct observation of the α-silylbenzylpotassium species 41 that acts as a resting state in the *t*BuOK-promoted reaction between toluene (23) and 3a (Scheme 5B).

From this perspective, the (alkoxy)silylation of both C(sp^2^)–H and C(sp^3^)–H bonds proceeds through anionic chain reactions involving organopotassium intermediates as chain carriers (I and II in Scheme 5C). This reactivity manifold, comprising metalation (I → II) and silylation (II → III) events, does not radically differ from the one observed with trialkylsilyl-based diazenes, thereby demonstrating that the diazenyl unit (*t*Bu–N=N^−^) is a better leaving group than an alkoxide (RO^−^ with R = Et, iPr or *t*Bu). In some instances, however, products arising from the nucleophilic displacement of both units have been detected (*e.g.*, the bis-indole 5, see Table 1), especially when the alkoxide residue is sterically unhindered (R = Et or iPr). Although the exact pathway underpinning the formation of doubly-substituted products remains to be studied, the substitution of the alkoxy group in III by II may become kinetically accessible when the concentration of III is greater than that of the corresponding diazene (see Scheme 5C, dashed arrows). In line with this hypothesis, 5 was formed in 62% yield when the silylated indole 4b was introduced from the outset of a reaction between indole 4 and diazene 3b (Scheme 5D).

## Conclusions

To conclude, we have demonstrated that the preparation of various organo(alkoxy)silanes can be accomplished through the potassium-catalyzed alkoxysilylation of both arylic C(sp^2^)–H and benzylic C(sp^3^)–H bonds with newly-synthesized *tert*-butyl-substituted alkoxysilyldiazenes. These operationally-simple transformations proceed under ambient, transition metal-free conditions and involve organopotassium compounds as reactive intermediates, high nucleophilicity of which allows the introduction of silyl groups as bulky as the tris-*tert*-butoxysilyl (Si(O*t*Bu)_3_). Because organoalkoxysilanes are widely used in synthetic chemistry and in materials science, it is our hope that the present methodology will stimulate further applications, notably owing to the easy access to bench-stable organosilanes bearing mono-, bis- or tris-*tert*-butoxysilyl units from readily available hydrocarbon substrates.

## Data availability

The data supporting this article have been included as part of the ESI.† Crystallographic data for compounds 5 and 21a have been deposited at the Cambridge Crystallographic Data Centre under accession numbers 2394041 and 2394042 and can be obtained free of charge from http://www.ccdc.cam.ac.uk/structures.

## Author contributions

L. S. conducted all the experiments and analyzed the data. L. V. performed preliminary experiments. C. C. designed and directed the research and wrote the original draft. C. C. and L. S. wrote the ESI.†

## Conflicts of interest

There are no conflicts to declare.

## Supplementary Material

SC-016-D5SC02059J-s001

SC-016-D5SC02059J-s002
