# Supplementary material for: Catalytic alkoxysilylation of C–H bonds with tert-butyl-substituted alkoxysilyldiazenes
Source: Chem Sci. 2025 Apr 24;16(22):9794–801. doi: 10.1039/d5sc02059j (PMC12042343; doi:10.1039/d5sc02059j)
Supplement: SC-016-D5SC02059J-s001 [file SC-016-D5SC02059J-s001.pdf]

Supplementary Information (SI) for Chemical Science.
This journal is © The Royal Society of Chemistry 2025

Supporting Information

Catalytic Alkoxysilylation of C–H bonds with *tert*-Butyl-Substituted Alkoxysilyldiazenes

Lamine Saadi, Loïc Valade and Clément Chauvier*

Sorbonne Université, CNRS, Institut Parisien de Chimie Moléculaire, 4 Place Jussieu, 75005 Paris, France.

clement.chauvier@sorbonne-universite.fr

1. General information 2
2. Optimization studies 4
  2.1. Alkoxysilylation of N-methylindole (**4**) 4
  2.2. Alkoxysilation of fluoroarenes 6
  2.3. Alkoxysilylation of electron-rich methylarenes 8
3. Comparison with literature protocols 9
  3.1. Preparation of the required starting materials and catalysts. 9
  3.2. Silylation of **4** and **21** 10
4. Mechanistic studies: 14
  4.1. Anionic *ortho*-Fries rearrangement 14
  4.2. Benzylic $C(sp^3)$–H alkoxysilylation: resting state identification 17
5. Synthesis and characterization of *tert*-butyl-substituted alkoxysilyldiazenes 21
  5.1. Preparation of alkoxychlorosilanes 21
  5.2. Synthesis and characterization of monoalkoxysilyldiazenes **3a**, **3b** and **3c** 23
  5.3. Synthesis and characterization of bis(*tert*-butoxy)silyldiazene **3d** 25
  5.4. Synthesis and characterization of tris(*tert*-butoxy)silyldiazene **3e** 26
  5.5. Stability of diazenes **3a-d** under basic conditions 28
6. Synthesis and characterization of silylated products 30
  6.1. General procedures 30
  6.2. Characterization data for silylated (hetero)arenes 30
  6.3. Characterization data for silylated toluene derivatives 42
  6.4. Applications (Scheme 4A and 4C in the manuscript) 47
7. NMR spectra of new compounds 51
8. References 188

1. General information

Reactions were performed in flame-dried glassware using an MBraun glove box (O_2 < 0.5 ppm, H_2O < 0.5 ppm) or conventional Schlenk techniques under a static pressure of argon unless otherwise stated. Glassware for reactions was flame-dried under vacuum prior to use. Liquids and solutions were transferred with syringes. All stated temperatures refer to external temperatures.

Tetrahydrofuran (THF) was dried over sodium/benzophenone, thermally distilled, degassed with three freeze-pump-thaw cycles and stored in a glove box over thermally activated 4 Å molecular sieves (MS). *n*-Pentane was obtained from Aldrich and degassed by argon bubbling (> 30 min) prior to use. Unless otherwise stated, standard solvents and reagents were obtained from Doug Discovery, Acros, Alfa Aesar, Sigma-Aldrich,Tokyo Chemical Industry (TCI), or BLD Pharmatech Ltd. and used as received. Me_3SiOK (Aldrich) as well as *t*BuOLi, *t*BuONa (Aldrich) and *t*BuOK (Aldrich) were sublimed under high vacuum prior to use. *N*-methylindoles (**4** and **6**-**10**) and N-methyl(7-azaindole) (**11**) were prepared following reported procedure.[1] Benzyl potassium was prepared following reported procedure.[2]

Flash column chromatography was performed on Silica 60 M (40–63 µm, Macherey Nagel) silica gel. Technical grade solvents were distilled prior to use. TLC analyses were performed on Merck 60 F254 silica gel pre-coated aluminum-backed plates with a layer thickness of 200 µm. Product spots were visualized under UV light (λ_{max} = 254 nm) and/or by staining with a potassium permanganate or a phosphomolybdic acid solution.

^{1}H, ^{13}C, ^{29}Si and ^{19}F NMR spectra were recorded on Bruker AV300 and AV400 instruments. $CDCl_3$ was purchased from Eurisotop and used as received. THF-d_8 (Eurisotop or Sigma-Aldrich) was degassed by freeze-pump-thaw method and stored over activated 4 Å molecular sieves prior to use. Chemical shifts are reported in parts per million (ppm) and are referenced to the residual solvent signals as the internal standard (THF-d_8: δ = 3.58 ppm and 1.72 ppm, $CDCl_3$: δ = 7.26 ppm for ^{1}H NMR and THF-d_8: δ = 67.57 ppm and 25.37 ppm, $CDCl_3$: 77.16 ppm for ^{13}C NMR). Data are reported as follows: chemical shift, multiplicity (s = singlet, d = doublet, dd = doublet of doublets, dt = doublet of triplets, t = triplet, tt = triplet of triplets, q = quartet, hept = heptuplet, m = multiplet, b = broad), coupling constants (Hz) and integration. ^{29}Si and ^{19}F NMR spectra were calibrated according to the IUPAC recommendation using a unified chemical shift scale based on the proton resonance of Me_4Si as primary reference. Infrared (IR) spectra were recorded on Tensor 27 FT-IR spectrometer (Bruker) at 4 cm^{-1} resolution equipped with an ATR accessory. Melting points (M.p.) were determined with a Stuart Scientific SMP3 melting point apparatus and are not corrected.

High resolution mass spectrometry (HRMS) analyses were obtained using a S3 mass spectrometer MicroTOF from Bruker with an electron spray ion source (ESI) and a TOF detector at the Institut Parisien de Chimie Moléculaire (Sorbonne Université). Compound names were generated by the computer program ChemDraw according to the guidelines specified by the International Union of Pure and Applied Chemistry (IUPAC).

Gas liquid chromatography (GLC) was performed on an Agilent Technologies 7820A gas chromatograph equipped with a HP-5 capillary column (30 m × 0.32 mm, 0.25 μm film thickness) by Agilent Technologies/CS-Chromatographie Service using the following parameters: H_2 carrier gas, injection temperature 220 °C, detector temperature 300 °C, flow rate: 2.4 mL/min; temperature program: start temperature 35 °C during 10 min then heating rate of 10 °C/min, end temperature 200 °C for 10 min.

2. Optimization studies

2.1. Alkoxysilylation of N-methylindole (**4**)

<u>Procedure:</u> In a glove box, a 2-mL vial equipped with a magnetic stirring bar was charged with the catalyst (*x* mol%), THF (0.4 mL) and N-methylindole (12.5 µL, 0.1 mmol 1 eq.). To the resulting vigorously stirred mixture was then added dropwise a solution of the corresponding *N*-*tert*-butyl-*N*'-alkoxydimethylsilyldiazene (*y* eq.) in THF (0.3 mL) at room temperature. After stirring for 1 h, 1,3,5-trimethoxybenzene was added and the resulting crude mixture was concentrated by rotary evaporation. The crude residue was then analyzed by ^{1}H NMR spectroscopy to determine yield and conversion.

Table S1: Optimization of the C(sp^3)-H silylation of N-methylindole with diazene **3a**.

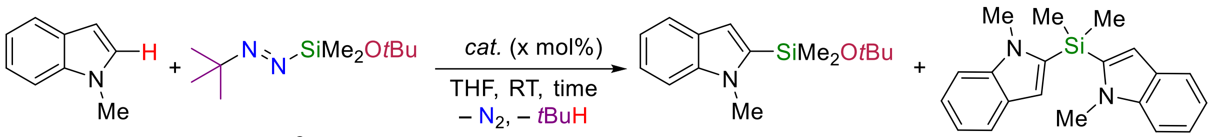

4 **3a** (*y* equiv.) **4a** **5**

Entry	Parameter	Cat. [*x*]	*y*	Solvent	time	Conversion	Yield **4a**[a]	Yield **5**[a]
1	Cation effect	*t*BuONa [10]	1.5	THF (0.14 M)	1 h	60%	50% (64%)[b]	5%
2		*t*BuOLi [10]	1.5	THF (0.14 M)	24 h	10%	3%	3%
3	Loading	*t*BuOK [5]	1.5	THF (0.14 M)	1 h	87%	78%	5%
4		*t*BuOK [20]	1.5	THF (0.14 M)	1 h	95%	70%	10%
5	Concentration	*t*BuOK [10]	1.5	THF (0.05 M)	1 h	86%	74%	11%
6		*t*BuOK [10]	1.5	THF (1 M)	1 h	75%	61%	8%
7	Equiv	*t*BuOK [10]	1.5	THF (0.14 M)	1 h	91%	78%	7%
8		*t*BuOK [10]	1.8	THF (0.14 M)	1 h	>95%	88%	7%
9		*t*BuOK [10]	2.0	THF (0.14 M)	1 h	>95%	89%	6%
10	Potassium salts	KOH [10]	1.8	THF (0.14 M)	1 h	NR	-	-
11		TMSOK [10]	1.8	THF (0.14 M)	1 h	95%	83%	7%

[a]Yields determined by ^{1}H NMR on the crude reaction mixture with 1,3,5-trimethoxybenzene as internal standard. NR: no reaction. [b]GC yield after 24h.

Table S2: Optimization of the C(sp^3)-H silylation of N-methylindole with diazene **3b**.

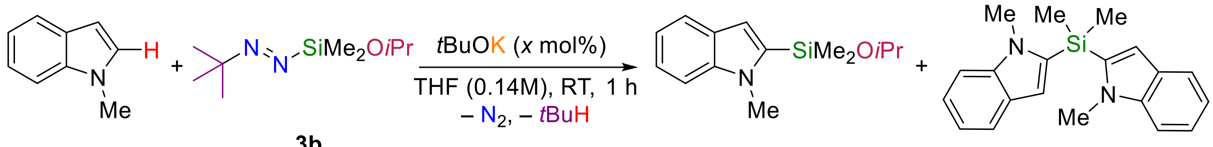

4 **3b** (*y* equiv.) **4b** **5**

Entry	*x*	y	Conversion	Yield **4b**^{a}	Yield **5**^{a}
1	10	1.2	59%	11%	32%
2	10	1.5	66%	40%	15%
3	10	3	86%	73%	7%
4	5	3	72%	59%	2%
5	50	2.5	73%	7%	40%

Table S3: Optimization of the C(sp^3)-H silylation of N-methylindole with diazene **3c**.

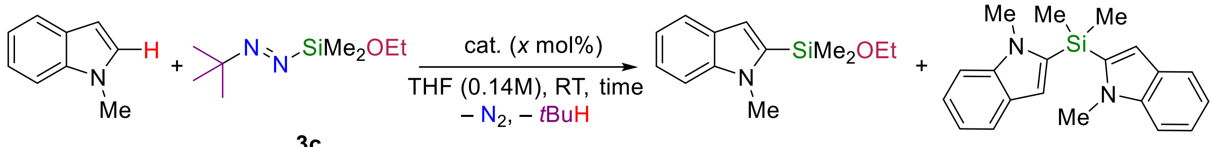

4 **3c** (*y* equiv.) **4c** **5**

Entry	Cat. [*x*]	*y*	time	Conversion	Yield **4c**^{a}	Yield **5**^{a}
1	*t*BuOLi [10]	1.2	22 h	38%	10%	-
2	*t*BuONa [10]	1.2	1 h	35%	15%	-
3	Me_3SiOK [10]	1.2	1 h	55%	16%	16%
4	*t*BuOK [10]	1.2	1 h	55%	10%	30%
5	*t*BuOK [10]	3	1 h	69%	63%	4%
6	*t*BuOK [5]	1.2	1 h	49%	17%	20%
7	*t*BuOK [20]	1.2	1 h	51%	3%	27%
8	*t*BuOK [50]	2.5	1 h	73%	3%	50% (48%)^b
9	*t*BuOK [100]	3	1 h	62%	10%	25%

^{a} NMR yields were determined using 1,3,5-trimethoxybenzene as internal standard. ^{b} Isolated yield.

Table S4: Influence of the silyl group on the silylation of N-methylindole.

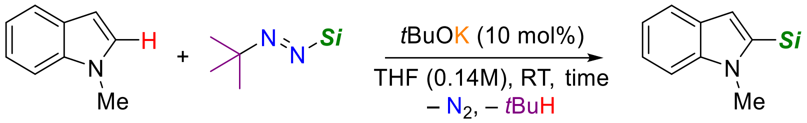

4 **3a-e** (1.8 equiv.) **4a-e**

Entry	*Si*	y	time	Conversion	Yield **4a-e**	Yield **5**
1	$SiMe_2OtBu$	1.8	1 h	>95%	88% (**4a**)	7%
2	$SiMe_2OiPr$	1.8	1 h	70%	51% (**4b**)	9%
3	$SiMe_2OEt$	1.8	1 h	65%	42% (**4c**)	10%
4	$SiMe(OtBu)_2$	1.8	16 h	88%	77% (**4d**)	–
5	$Si(OtBu)_3$	1.8	84 h	0%	–	–
6[b]	$Si(OtBu)_3$	2.2	24 h	75%	68% (**4e**)	–

[a]NMR yields were determined using 1,3,5-trimethoxybenzene as the internal standard. [b]*t*BuOK 40 mol%.

Table S5: Influence of the silyl group on the formation of bis-indole product **5**.

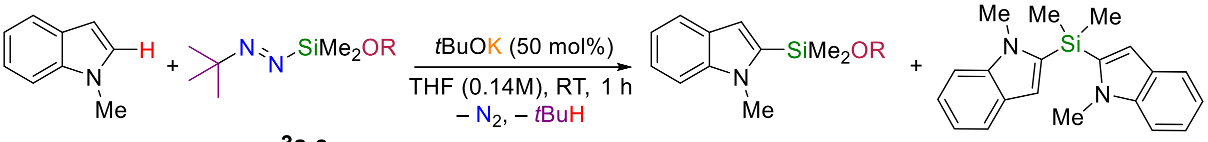

4 **3a-c** (2.5 equiv.) **4a-c** **5**

Entry	R	Conversion	Yield **4a-c**	Yield **5**
1	Et	73%	3% (**4c**)	50%
2	*i*Pr	67%	7% (**4b**)	40%
3	*t*Bu	91%	73% (**4a**)	3%

2.2. Alkoxysilation of fluoroarenes

<u>Procedure</u>: In a glove box, a 2-mL vial equipped with a magnetic stirring bar was charged with *t*BuOK (*x* mol%), THF (0.4 mL) and fluorobenzene (9.4 μL, 0.1 mmol 1 eq.). To the resulting vigorously stirred mixture was then added dropwise a solution of the corresponding *N-tert*-butyl-*N'*-alkoxydimethylsilyldiazene (*y* eq.) in THF (0.3 mL) at room temperature. After stirring for 1 h, bis(4-fluorophenyl)methanone was added and the resulting crude mixture was concentrated by rotary evaporation. The crude residue was then analyzed by ^{19}F NMR spectroscopy to determine the yield of monosilylation and bis-silylation products.

Table S6: Mono vs. bis-alkoxysilylation of fluorobenzene (**16**).

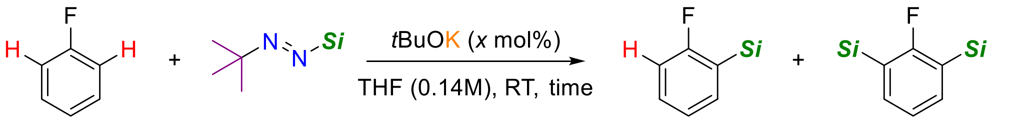

16 **3a**, **3d** or **3e** (*y* equiv.) **16a**, **16d** or **16e** $\mathbf{16a_2}$, $\mathbf{16d_2}$ or $\mathbf{16e_2}$

Entry	***Si***	*x*	*y*	time	Yield **16a/d/e**	Yield **16a$_2$/d$_2$/e$_2$**
1	$SiMe_2OtBu$ (**3a**)	10	1.2	1 h	**67%**	24%
2		10	3.0	30 min	35%	55%
3		20	3.0	1 h	22%	**75%**
4	SiMe(O*t*Bu)$_2$ (**3d**)	10	1.2	1 h	73%	6%
5	Si(O*t*Bu)$_3$ (**3e**)	10	1.2	21 h	17%	-
6		30	1.4	21 h	**71%**	2%

Yields were determined by $^{19}F\{^{1}H\}$ NMR spectroscopy using bis(4-fluorophenyl)methanone as an internal standard.

Table S7: Mono vs. bis-silylation of 2-fluorotoluene (**22**).

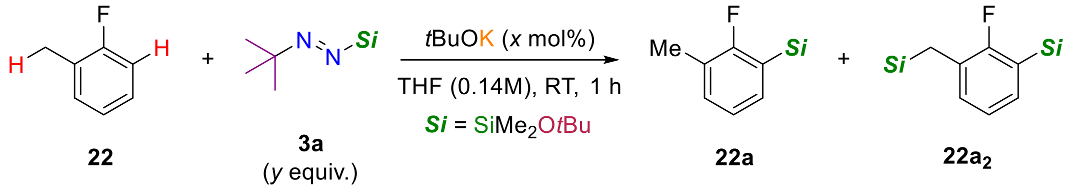

Entry	*x*	*y*	Conversion	Yield **22a**	Yield **22a$_2$**
1	10	1.8	97%	60%	16%
2	20	4.0	100%	25%	66%

Yields were determined by $^{19}F\{^{1}H\}$ NMR spectroscopy using (2-fluoro-1,3-phenylene)bis(trimethylsilane) as an internal standard.

2.3. Alkoxysilylation of electron-rich methylarenes

Procedure: In a glove box, a 2-mL vial equipped with a magnetic stirring bar was charged with the cat (*x* mol%), THF (0.1 mL) and the corresponding substrate (0.1 mmol). To the resulting vigorously stirred mixture was then added the corresponding silylated *tert*-butyldiazene (3 eq.) in one portion at room temperature at room temperature. After stirring for 16 h, 1,3,5-trimethoxybenzene was added and the resulting crude mixture was concentrated by rotary evaporation. The crude residue was then analyzed by ^{1}H NMR spectroscopy.

Table S8: Silylation of electron-rich *para*-substituted methylarenes.

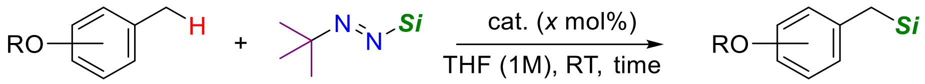

3a
(3 equiv.)

Entry	Substrate	Cat. [*x*]	*Si*	ρ	Yield[a]
1		*t*BuOK [10]	$SiMe_2OEt$	-	n.d.
2		*t*BuOK [10]	$SiMe_2O$*i*Pr	-	n.d.
3		*t*BuOK [10]	SiMe(O*t*Bu)$_2$	22%	9%
4	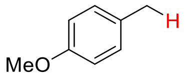 **25**	*t*BuOK [10]		21%	9%
5		*t*BuOK [30]		18%	4%
6		CsF [10]		-	n.d.
7		*t*BuONa [10]	$SiMe_2O$*t*Bu	-	n.d.
8		Me_3SiOK [10]		24%	12%
9	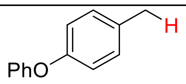 **27**	*t*BuOK [10]		23%	8%

[a]Yields were determined by ^{1}H NMR spectroscopy using 1,3,5-trimethoxybenzene as the internal standard. n.d.: not detected.

3. Comparison with literature protocols

To further assess the utility of our catalytic alkoxysilylation protocol, we set out to compare our results with those obtained by alternative alkoxysilylation methods from the literature. To that end, we first evaluated the outcome of the reaction between two model substrates (**4** and **21**) and H–$SiMe_2$O*t*Bu promoted by transition metal-based catalysts known to trigger the alkoxysilylation of C(sp^2)–H bonds. The first evaluated catalytic system, described by Lee and coworkers, is composed of the well-defined $[RhCl(Ph\text{-}BPE)]_2$ complex (Ph-BPE = (+)-1,2-bis((2S,5S)-2,5-diphenylphospholano)ethane).[3] Alternatively, we also evaluated Hartwig's Ir-based catalytic system ($[Ir(cod)(OMe)]_2$ + 2,4,7-trimethyl-1,10-phenanthroline) under Yorimitsu and Shimokawa's conditions.[4] The latter protocol offers more flexibility regarding the silyl groups tolerated in the silylation reaction compared to Hartwig's original conditions,[5] which were limited to the introduction of the $SiMe(OSiMe_3)_2$ group from the corresponding disiloxyhydrosilane. Finally, we also compared our methodology to a typical stoichiometric deprotonation/silylation strategy based on a metalation step with *n*BuLi followed by an electrophilic quench with Cl–$SiMe_2$O*t*Bu.

3.1. Preparation of the required starting materials and catalysts.

2,4,7-Trimethyl-1,10-phenanthroline[5] and $[RhCl(Ph\text{-}BPE)]_2$ (ref. 3) were prepared according to reported procedures and gave analytical data identical to those described.

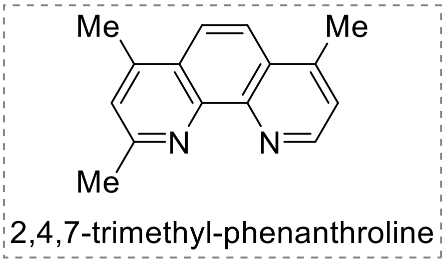

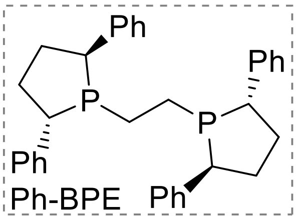

(Aldrich)

Tert-butoxydimethylsilane ($HSiMe_2$O*t*Bu) was prepared according to the following procedure:

A flame-dried, two-necked round-bottomed flask equipped with a stirring bar and a rubber septum was sequentially charged with urea (1.26 g, 21 mmol, 1.05 eq.) and chlorodimethylsilane (2.22 mL, 20 mmol, 1 eq.). The resulting mixture was cooled to 0 °C and *tert*-butanol (2 mL, 21 mmol, 1.05 eq.) was then added dropwise. The resulting mixture was allowed to warm to room temperature and stirred for 5 h. The volatiles were then separated from the solid byproduct (urea hydrochloride) by vacuum transfer under dynamic vacuum, resulting in a clear solution containing the volatile disiloxane side product $[(HSiMe_2)_2O]$ and the desired hydrosilane. The siloxane was then ultimately removed by heating the mixture at ca.

80 °C under argon, allowing the recovery of the analytically pure title hydrosilane (516.6 mg, 3.91 mmol, 20 %) as a colorless liquid.

H−SiMe$_2$O*t*Bu
$C_6H_{16}OSi$
mw: 132.28 g/mol

^{1}H NMR (300 MHz, C_6D_6) δ/ppm = 5.02 (hept, *J* = 2.8 Hz, 1H), 1.23 (s, 9H), 0.19 (d, *J* = 2.8 Hz, 6H). **^{13}C{^{1}H} NMR** (75 MHz, C_6D_6) δ/ppm = 72.4, 31.5, 1.0. **^{1}H/^{29}Si HMQC NMR** (300/60 MHz, C_6D_6): δ/ppm = 5.02/-8.8.

3.2. Silylation of **4** and **21**

The results obtained for the silylation of **4** are presented in Table S9 while those involving **21** are presented in Table S10.

Table S9: Comparison of various protocols for the C(sp^2)–H silylation of indole **4**.

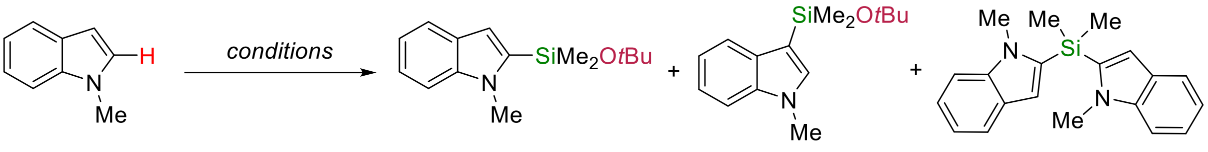

4 **4a** **4a'** **5**

Entry	Conditions	**4a**^{a} [%]	**4a'**^{a} [%]	**5**^{a} [%]
1	[Rh(Ph-BPE)Cl]$_2$ (0.5 mol%)<br>HSiMe$_2$O*t*Bu (1.0 eq)<br>cyclohexene (1.2 eq)<br>THF (1 M), 50 °C, 20 h.	0	0	0
2	[Ir(cod)OMe]$_2$ (2.5 mol%)<br>2,4,7-Me$_3$-phenantroline (5.5 mol%)<br>HSiMe$_2$O*t*Bu (1.5 eq)<br>*t*BuCHCH$_2$ (1.5 eq)<br>Neat, 100 °C, 60 h	30	25	0
3	*n*BuLi (1.1 eq), THF (0.2 M), 0 °C to RT, 1 h<br>*then* ClSiMe$_2$O*t*Bu (1.4 eq), RT, 3 h	60	0	4
4	**This work:**<br>*t*BuOK (10 mol%), **3a** (1.8 eq)<br>THF (0.14 M), RT, 1 h.	88	0	7

^{a} Yields were determined by ^{1}H NMR spectroscopy using 1,3,5-trimethoxybenzene as internal standard.

Experimental procedures for experiments described in Table S9:

- Entry 1: Following a reported procedure,[3] an oven-dried 10 mL microwave vial was charged in a glovebox with [RhCl(Ph-BPE)]$_2$ (1.9 mg, 1.5 µmol, 0.5 mol%), *N*-methylindole (0.3 mL, 2.4 mmol, 8.0 eq.) and THF (0.3 mL). The vial was sealed and the mixture was stirred at 50 °C for 1 h outside the glovebox. After cooling to room

temperature, the vial was brought back into the glovebox, opened and charged with cyclohexene (36.5 µL, 0.36 mmol, 1.2 eq.) and *tert*-butoxydimethylsilane (39.7 mg, 0.3 mmol, 1 eq). The vial was sealed again, removed from the glovebox and the reaction mixture was stirred at 50 °C for 20 h. After cooling to room temperature, 1,3,5-trimethoxybenzene was added as an internal standard and the crude mixture was concentrated by rotary evaporation. The crude residue was then analyzed by ^{1}H NMR spectroscopy to determine the yields.

- Entry 2: Following a reported procedure,[4] an oven-dried 10 mL microwave vial was charged in a glovebox with $[Ir(cod)(OMe)]_2$ (5 mg, 8 µmol, 2.5 mol%), 2,4,7-trimethyl-1,10-phenanthroline (3.7 mg, 17 µmol, 5.5 mol%), *N*-methylindole (37 µL, 0.3 mmol, 1.0 eq.), *tert*-butoxydimethylsilane (59.5 mg, 0.45 mmol, 1.5 eq) and 3,3-dimethyl-1-butene (58 µL, 0.45 mmol, 1.5 mmol). The vial was sealed, removed from the glovebox and the reaction mixture was stirred at 100 °C for 60 h. After cooling to room temperature, 1,3,5-trimethoxybenzene was added as an internal standard and the crude mixture was concentrated by rotary evaporation. The crude residue was then analyzed by ^{1}H NMR spectroscopy to determine the conversion and the yields.
- Entry 3: A flame-dried 25 mL Schlenk flask equipped with a stirring bar and a rubber septum was charged with *N*-methylindole (37 µL, 0.3 mmol, 1 eq.) and THF (1.5 mL, 0.2M). The resulting pale-yellow solution was cooled to 0 °C, and freshly titrated *n*BuLi (0.15 mL, 2.2 M in hexanes, 0.33 mmol, 1.1 eq.) was added dropwise. The mixture was allowed to warm to room temperature and stirred for 1 h. T*ert*-butoxychlorodimethylsilane (0.6 mL, 0.7M in THF, 0.42 mmol, 1.4 eq) was then added and the mixture was stirred for 3 h. After exposure of the crude reaction mixture to air, 1,3,5-trimethoxybenzene was added and the volatiles were removed by rotary evaporation. The resulting crude residue was then analyzed by ^{1}H NMR spectroscopy to determine the conversion and the yields.

Table S10: Comparison of various protocols for the C(sp^2)–H silylation of fluorobenzene **21**.

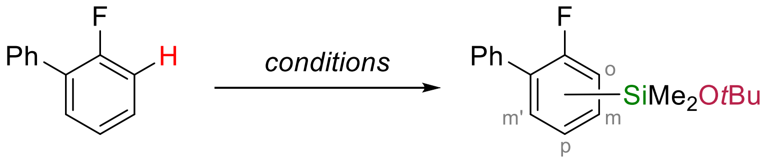

21 **21a (o) + other isomers**

Entry	Conditions	**21a**[a]	**mono-silylated isomers**		
1	[Rh(Ph-BPE)Cl]$_2$ (0.5 mol%), $HSiMe_2O$*t*Bu (1.0 eq) cyclohexene (1.2 eq) THF (1 M), 100 °C, 20 h	1 %	2 %	2 %	1 %
2	[Ir(cod)OMe]$_2$ (1.5 mol%) 2,4,7-Me$_3$-phenantroline (3.5 mol%) $HSiMe_2O$*t*Bu (1.5 eq) cyclohexene (1.0 eq) THF (0.9 M), 100 °C, 24 h	7 %	7 %	6 %	6 %
3	[Ir(cod)OMe]$_2$ (2.5 mol%) 2,4,7-Me$_3$-phenantroline (5.5 mol%) $HSiMe_2O$*t*Bu (1.5 eq) *t*BuCHCH$_2$ (1.5 eq) THF (1 M), 100 °C, 60 h	3 %	4 %	4 %	4 %
4	*n*BuLi (1.1 eq), THF (0.2 M), 0 °C to RT, 1 h *then* $ClSiMe_2O$*t*Bu (1.4 eq), RT, 3 h	0 %	–	–	–
5	**This work:** *t*BuOK (10 mol%), **3a** (1.5 eq) THF (0.14 M), RT, 1 h	95 %[b]	–	–	–

[a] Yields were determined by $^{19}F\{^{1}H\}$ NMR spectroscopy using 2-fluoro-1,3-dimethylbenzene as an internal standard. [b]Isolated yield.

Experimental procedures for experiments described in Table S10:

- Entry 1: Following a reported procedure,[3] an oven-dried 10 mL microwave vial was charged in a glovebox with [RhCl(Ph-BPE)]$_2$ (1.9 mg, 1.5 µmol, 0.5 mol%), 2-fluoro-1,1'-biphenyl (56.8 mg, 0.33 mmol, 1.1 eq) and THF (0.3 mL). The vial was sealed and the mixture was stirred at 100 °C for 1 h outside the glovebox. After cooling to room temperature, the vial was brought back into the glovebox, opened and charged with cyclohexene (36.5 µL, 0.36 mmol, 1.2 eq.) and *tert*-butoxydimethylsilane (39.7 mg, 0.3 mmol, 1 eq). The vial was sealed again, removed from the glovebox and the reaction mixture was stirred at 100 °C for 20 h. After cooling to room temperature and removal of the volatiles under reduced pressure, 2-fluoro-1,3-dimethylbenzene (internal

standard) was added to the crude residue, which was then analyzed by $^{19}F\{^{1}H\}$ NMR spectroscopy to determine the yields.

- Entry 2: Following a reported procedure,[5] an oven-dried 10 mL microwave vial was charged in a glovebox with $[Ir(cod)(OMe)]_2$ (3 mg, 4.5 µmol, 1.5 mol%), 2,4,7-trimethyl-1,10-phenanthroline (2.1 mg, 9.3 µmol, 3.1 mol%), 2-fluoro-1,1'-biphenyl (51.7 mg, 0.3 mmol, 1.0 eq.),THF (0.34 mL), *tert*-butoxydimethylsilane (59.5 mg, 0.45 mmol, 1.5 eq.) and cyclohexene (30.4 µL, 0.3 mmol, 1.0 eq.). The vial was sealed, removed from the glovebox and the reaction mixture was stirred at 100 °C for 24 h. After cooling to room temperature and removal of the volatiles under reduced pressure, 2-fluoro-1,3-dimethylbenzene (internal standard) was added to the crude residue, which was then analyzed by $^{19}F\{^{1}H\}$ NMR spectroscopy to determine the yields.
- Entry 3: Following a reported procedure,[4] an oven-dried 10 mL microwave vial was charged in a glovebox with $[Ir(cod)(OMe)]_2$ (5 mg, 8 µmol, 2.5 mol%), 2,4,7-trimethyl-1,10-phenanthroline (3.7 mg, 17 µmol, 5.5 mol%), 2-fluoro-1,1'-biphenyl (51.7 mg, 0.3 mmol, 1 eq) ,THF (0.3 mL), *tert*-butoxydimethylsilane (59.5 mg, 0.45 mmol, 1.5 eq) and 3,3-dimethyl-1-butene (58 µL, 0.45 mmol, 1.5 mmol). The vial was sealed, removed from the glovebox and the reaction mixture was stirred at 100 °C for 60 h. After cooling to room temperature and removal of the volatiles under reduced pressure, 2-fluoro-1,3-dimethylbenzene (internal standard) was added to the crude residue, which was then analyzed by $^{19}F\{^{1}H\}$ NMR spectroscopy to determine the yields.
- Entry 4: A flame-dried 25 mL Schlenk flask equipped with a stirring bar and a rubber septum was charged with 2-fluoro-1,1'-biphenyl (51.7 mg, 0.3 mmol, 1.0 eq.) and THF (1.5 mL, 0.2M). The resulting colorless solution was cooled to 0 °C, and freshly titrated *n*BuLi (0.15 mL, 2.2 M in hexanes, 0.33 mmol, 1.1 eq.) was added dropwise. The mixture was allowed to warm to room temperature and stirred for 1 h. *Tert*-butoxychlorodimethylsilane (0.6 mL, 0.7M in THF, 0.42 mmol, 1.4 eq) was then added and the mixture was stirred for 3 h. After exposure of the crude reaction mixture to air, the volatiles were removed by rotary evaporation and 2-fluoro-1,3-dimethylbenzene (internal standard) was added to the crude residue, which was then analyzed by $^{19}F\{^{1}H\}$ NMR spectroscopy to determine the yields.

4. Mechanistic studies:

4.1. Anionic *ortho*-Fries rearrangement

<u>Procedure for the catalytic run</u>: In a glove box, a 2-mL vial equipped with a magnetic stirring bar was charged with *t*BuOK (1.1 mg, 10 mol%, 0.1 eq.), 3-fluorocarbamate (24 mg, 0.1 mmol, 1 eq.) and THF (0.35 mL). To the resulting vigorously stirred mixture was then added dropwise a solution of **3a** (65 mg, 0.3 mmol, 3 eq.) in THF (0.35 mL) at room temperature. An aliquot of the mixture was removed at different reaction times, diluted with 0.6 mL of $CDCl_3$ and submitted to multinuclear NMR spectroscopy and GC/MS analyses. Yields were determined by $^{19}F\{^1H\}$ NMR using (2-fluoro-1,3-phenylene)bis(trimethylsilane) as an internal standard.

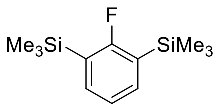

internal standard

Note: Structures of **39a** and **40*Si*** were further confirmed by GC/MS analysis.

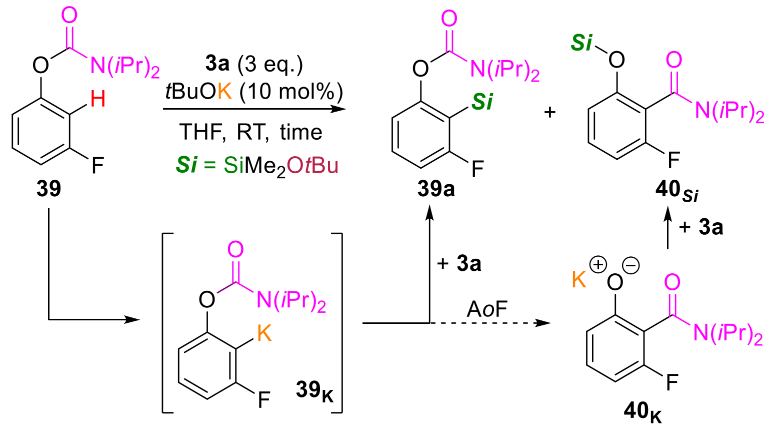

Table S11: Reaction evolution over time.

Entry	Time	Conversion	Yield of **40*Si***	Yield of **39a**
1	2 h	7%	1%	-
2	16 h	14%	7%	7%
3	40 h	33%	9%	13%
4	117 h	59%	15%	35%
5	166 h	74%	18%	47%

<u>Stoichiometric experiment</u>:

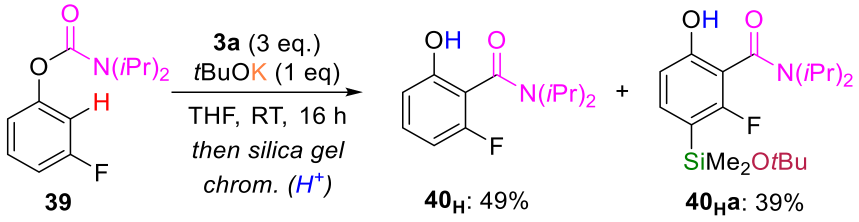

In a glove box, a 5-mL vial equipped with a magnetic stirring bar was charged with *t*BuOK (28.1 mg, 0.25 mmol, 1 eq.), 3-fluorophenyl diisopropylcarbamate (60 mg, 0.25 mmol, 1 eq.) and THF (1 mL). To the resulting solution was added dropwise a solution of diazene **3a** (162 mg, 0.75 mmol, 3 eq.) in THF (0.8 mL). The reaction mixture was stirred at RT overnight and then concentrated *in vacuo* by rotary evaporation. Purification by column chromatography on silica gel using PE/AcOEt (9:1 then 8:2 v/v) as eluent afforded the two rearranged products **40_H** (29.4 mg, 0,13 mmol, 49%) and **40_Ha** (36.4 mg, 0,1 mmol, 39%) as white solids.

40_H: ^{1}H NMR (400 MHz, $CDCl_3$): δ/ppm = 8.66 (b s, 1H), 7.05 (td, *J* = 8.3, 6.7 Hz, 1H), 6.64 (d, *J* = 8.3 Hz, 1H), 6.51 (t, *J* = 8.7 Hz, 1H), 3.71 (b s, 2H), 1.36 (b s, 12H). **^{13}C{^{1}H} NMR** (101 MHz, $CDCl_3$): δ/ppm = 165.8, 158.7 (d, *J* = 245.3 Hz), 156.9 (d, *J* = 6.7 Hz), 130.6 (d, *J* = 10.4 Hz), 113.2 (d, *J* = 2.9 Hz), 112.9 (d, *J* = 20.5 Hz), 106.3 (d, *J* = 22.1 Hz), 20.8.**^{19}F{^{1}H} NMR** (376 MHz, $CDCl_3$): δ/ppm = -114.80. The spectroscopic data match the reported literature.[6]

40_Ha: M.p (CH_2Cl_2) = 190-192 °C. **^{1}H NMR** (300 MHz, $CDCl_3$): δ/ppm = 8.02 (b s, 1H), 7.38 (dd, *J* = 8.1, 6.5 Hz, 1H), 6.76 (d, *J* = 8.2 Hz, 1H), 3.74 (s, 2H), 1.44 (b s, 12H), 1.26 (s, 9H), 0.38 (d, *J* = 1.3 Hz, 6H). **^{13}C{^{1}H} NMR** (75 MHz, CDCl3): δ/ppm = 166.2, 162.6 (d, J = 241.1 Hz), 158.4 (d, *J* = 7.1 Hz), 136.5 (d, J = 14.2 Hz), 116.06 (d, *J* = 31.1 Hz), 113.1 (d, *J* = 2.8 Hz), 112.1 (d, *J* = 24.9 Hz), 73.0, 32.1, 20.8, 2.2 (d, *J* = 1.6 Hz). **^{19}F{^{1}H} NMR** (282 MHz, $CDCl_3$): δ/ppm = -101.90. **^{1}H/^{29}Si HMQC NMR** (300/60 MHz, $CDCl_3$): δ/ppm = 7.38, 0.38/-4.3. **HRMS (ESI)** m/z: $[M+Na]^+$ Calcd for $C_{19}H_{32}FO_3SiNa$ 369.2028. Found 369.2027.

Potassium 2-(diisopropylcarbamoyl)-3-fluorophenolate

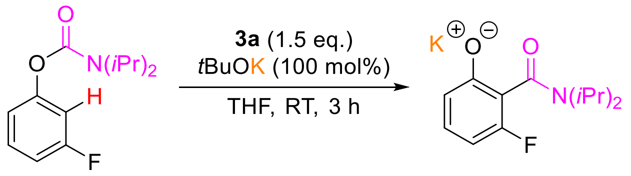

39 **40_K**: 82%

Following a reported procedure,[7] a 10-mL vial equipped with a magnetic stirring bar was charged in a glovebox with potassium *tert*-butoxide (56.1 mg, 0.5 mmol, 1 eq.), 3-fluorophenyl diisopropylcarbamate (120 mg, 0.50 mmol, 1.0 eq.) and THF (2 mL). To the resulting vigorously stirred mixture was then added dropwise a solution of diazene **3a** (162 mg, 0.75 mmol, 1.5 eq.) in THF (1.5 mL) at room temperature. The vial was capped with a rubber septum and vigorously stirred for 3 h. The THF was evaporated under reduced pressure affording a dark foamy solid residue, which was taken up in pentane (5 mL). The resulting suspension was sonicated for 3 min and then left settling for ca. 10 min upon which a yellow powder separated from the liquid layer. The supernatant was carefully removed and the solid residue was further dissolved in CH_2Cl_2, prior to being transferred to a round-bottomed flask. The solvent was

removed under reduced pressure to afford the title compound **40_K** (0.131 g, 0.41 mmol, 82%) as a yellow solid.

^{1}H NMR (300 MHz, $CDCl_3$): δ/ppm = 6.80 (q, *J* = 8.2 Hz, 1H), 6.15 (d, *J* = 8.3 Hz, 1H), 5.98 (t, *J* = 8.5 Hz, 1H), 3.93 (hept, *J* = 6.7 Hz, 1H), 3.46 (hept, *J* = 6.8 Hz, 1H), 1.41 (dd, *J* = 6.8, 2.4 Hz, 6H), 1.12 (d, *J* = 6.6 Hz, 3H), 1.04 (d, *J* = 6.6 Hz, 3H). **^{19}F {^{1}H} NMR** (282 MHz, $CDCl_3$): δ/ppm = -117.28. The spectroscopic data match the reported literature.[7]

4.2. Benzylic C(sp^3)–H alkoxysilylation: resting state identification

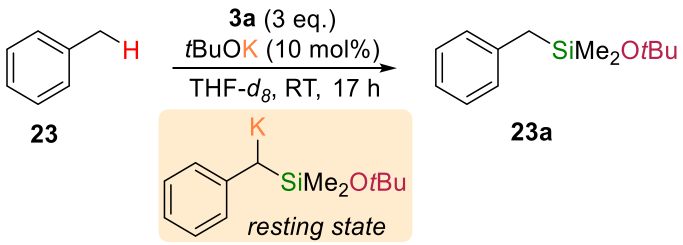

Procedure: In a glovebox, a 2-mL dram vial equipped with a stirring bar was charged with *t*BuOK (1.1 mg, 0.01 mmol,10 mol%), THF-d_8 (0.3 mL) and toluene (10.6 µL, 0.1 mmol, 1 eq.). To the vigorously stirred mixture was then added in one portion a solution of diazene **3a** (65 mg, 0.3 mmol, 3 eq.) in THF-d_8 (0.3 mL). After stirring for ca. 5 min, the resulting light brown mixture was transferred with a Pasteur pipet to an NMR tube equipped with a J-Young valve. The NMR tube was sealed under argon, brought out of the glovebox and the reaction mixture was analyzed by multinuclear NMR spectroscopy after 17 h.

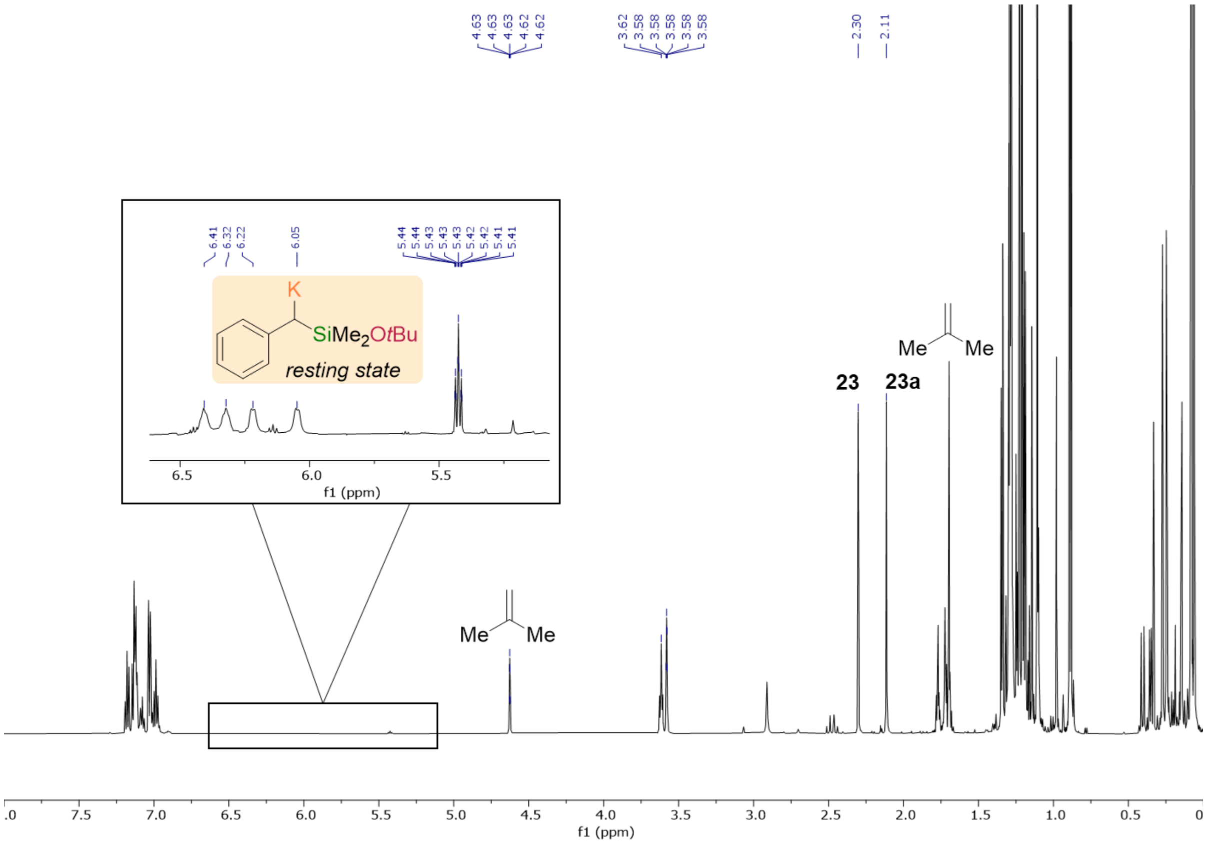

Figure S1: ^{1}H NMR (600 MHz, $CDCl_3$) of the crude mixture obtained after 17 h of reaction between toluene (**23**) diazene **3a** and *t*BuOK (10 mol%).

Careful analysis by NMR spectroscopy of the crude reaction mixture obtained upon reaction of toluene with diazene **3a** allowed to detect a small amount the α-silylbenzyl potassium species **41**. Beyond this organopotassium species, unreacted toluene, the silylation product

23a, isobutene, isobutane as well as $Me_2Si(OtBu)_2$ have also been detected among other unidentified side products. The formulation of **41** as an α-silylbenzyl potassium species was confirmed by its independent synthesis by deprotonation of **23a** with benzylpotassium (BnK):

<u>Independent synthesis of **41**</u>:

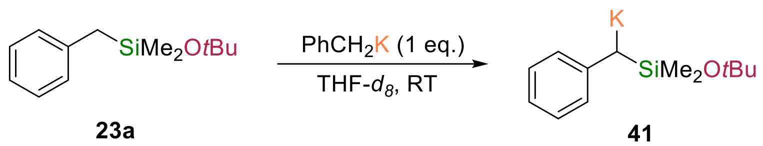

In a glovebox, a 2-mL dram vial equipped with a stirring bar was charged with benzyl(*tert*-butoxy)dimethylsilane **23a** (22.4 mg, 0.1 mmol, 1 eq.) followed by THF (0.3 mL). To the resulting clear solution was added in one portion and at room temperature a solution of benzyl potassium (13.0 mg, 0.1 mmol, 1 eq.) in THF (0.3 mL). The resulting light brown reaction mixture was stirred for 5 min and then transferred with a Pasteur pipet to an NMR tube equipped with a J-Young valve. The tube was sealed under argon, brought out the glovebox and the volatiles were removed under high vacuum. The crude residue was taken up in THF-d_8 and analyzed by multinuclear NMR spectroscopy.

The title compound **41** could be characterized by NMR spectroscopy:

^{1}H NMR (600 MHz, THF-d_8): δ/ppm = 6.40 (d, *J* = 7.6 Hz, 1H, *m*), 6.33 (d, *J* = 7.7 Hz, 1H, *m*'), 6.22 (d, *J* = 7.2 Hz, 1H, *o*), 6.03 (d, *J* = 7.4 Hz, 1H, *o*'), 5.42 (tt, *J* = 6.9, 1.2 Hz, 1H, *p*), 2.14 (s, 1H, PhC<u>H</u>KSi) 1.28 (s, 9H), 0.08 (s, 6H). **^{13}C{^{1}H} NMR** (151 MHz, THF): δ/ppm = 157.7 (C_{ipso}), 129.6 (br, C_m), 129.5 (br, $C_{m'}$), 120.1 (C_o), 114.9 ($C_{o'}$), 104.6 (C_p), 71.2 (SiO<u>C</u>(CH_3)$_3$), 54.4 (C_{benzyl}), 32.7(SiOC(<u>C</u>H_3)$_3$), 4.0 (Si<u>C</u>H_3). **^{1}H/^{29}Si HMQC NMR** (300/60 MHz, $CDCl_3$): δ/ppm 2.14, 0.08/-7.8.

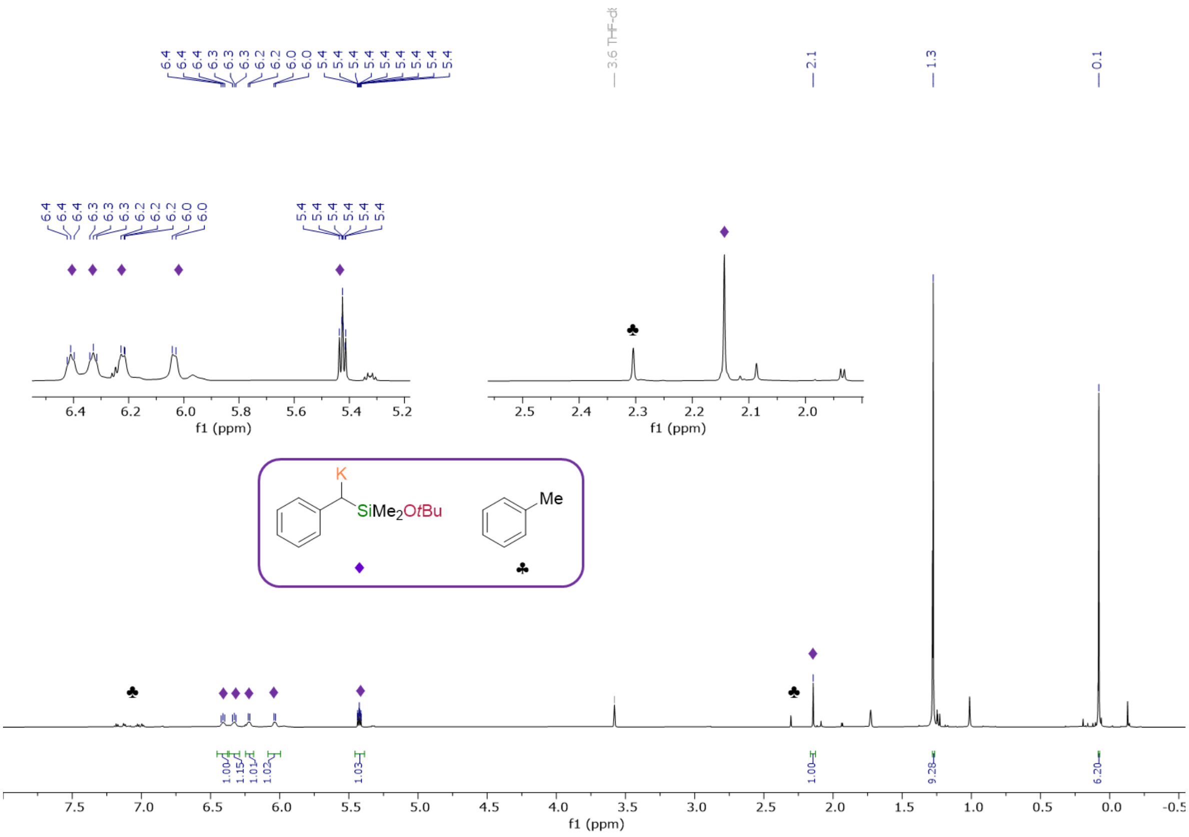

Figure S2: ^{1}H NMR (600 MHz, THF-d_8) of compound **41.**

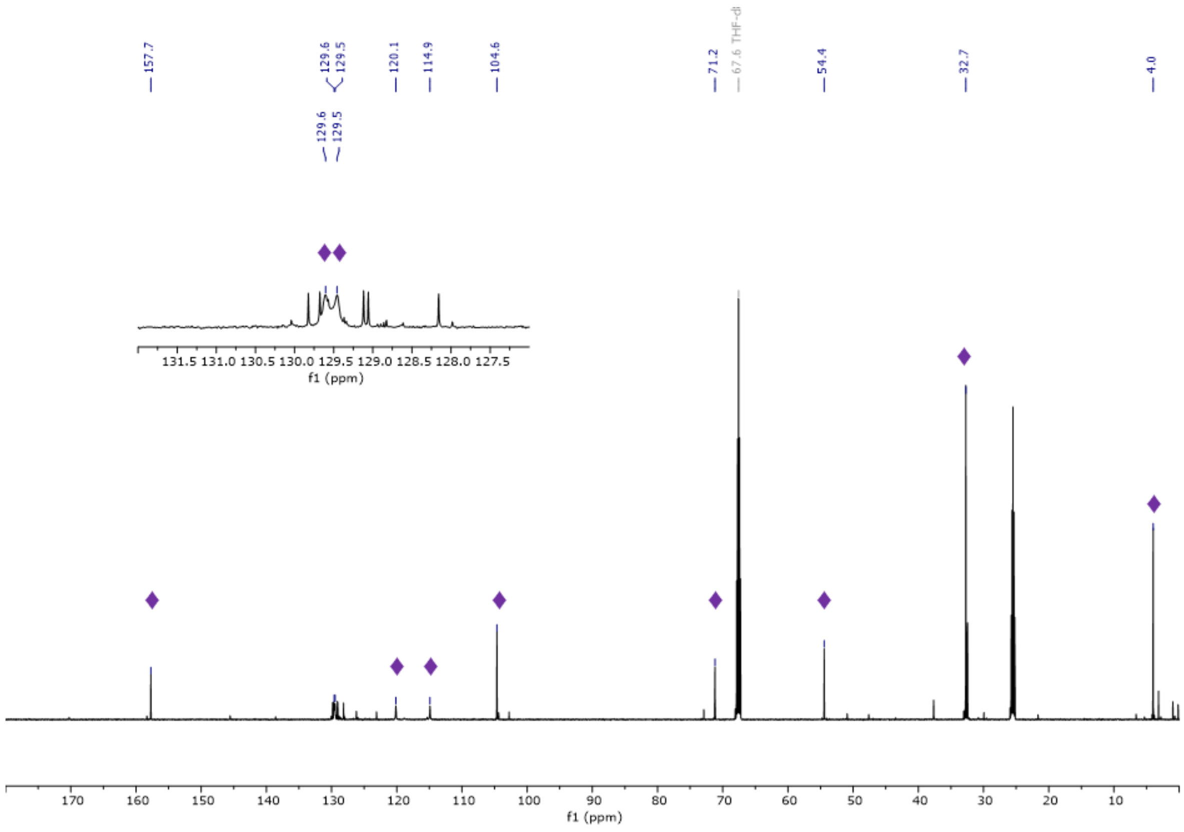

Figure S3: $^{13}C\{^{1}H\}$ NMR (151 MHz, THF-d_8) of compound **41**.

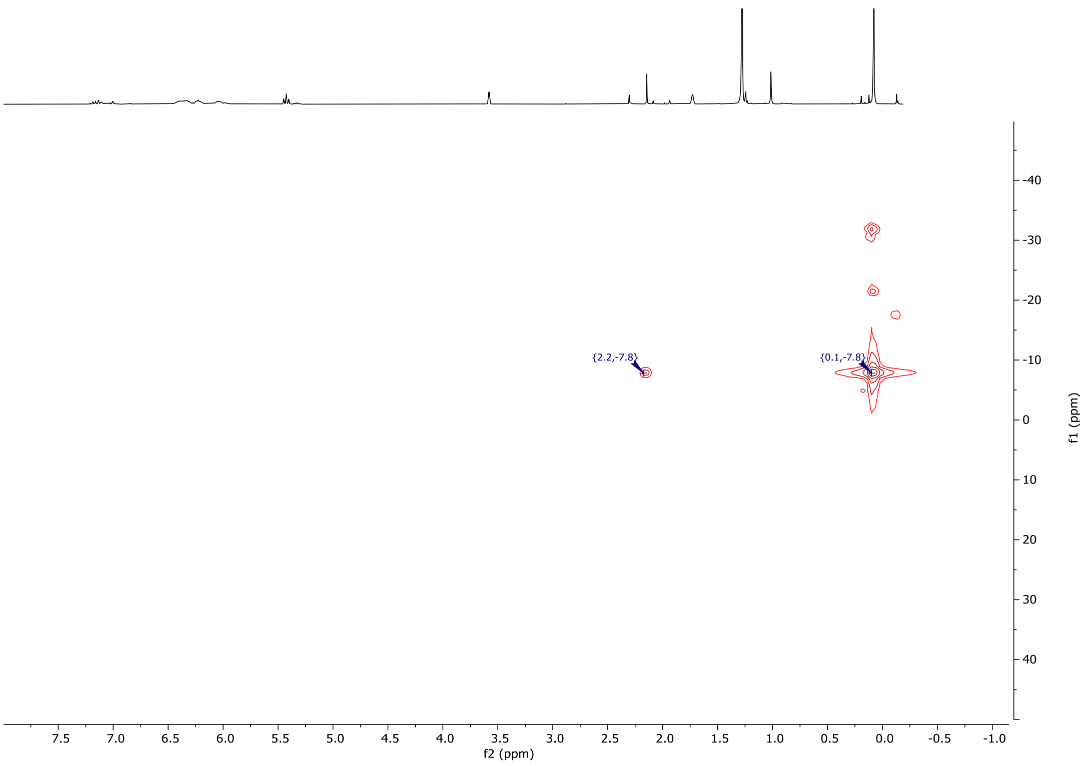

Figure S4: $^{1}H/^{29}Si$ HMQC NMR (300/60 MHz, THF-d_8) of compound **41**.

5. Synthesis and characterization of *tert*-butyl-substituted alkoxysilyldiazenes

5.1. Preparation of alkoxychlorosilanes

All alkoxychlorosilanes were synthesized from commercially available chlorosilanes Me_2SiCl_2, $MeSiCl_3$ and $SiCl_4$ and the corresponding absolute alcohols (EtOH, *i*PrOH and *t*BuOH dried over 4 Å MS) according to the procedures described below. Tri-*tert*-butoxychlorosilane ($ClSi(OtBu)_3$) was prepared according to reported procedure.[8]

Chloro(ethoxy)dimethylsilane

Cl—$SiMe_2OEt$

$C_4H_{11}ClOSi$

mw: 138.67 g/mol

A flame-dried, two-necked round-bottomed flask equipped with a stirring bar and a rubber septum was charged with urea (12.62 g, 210 mmol, 1.05 eq.) and dichlorodimethylsilane (6.45 mL, 200 mmol, 1 eq.). The resulting suspension was cooled to 0 °C and ethanol (12.25 mL, 210 mmol, 1.05 eq.) was then added dropwise. The reaction mixture was allowed to warm to room temperature and stirred for 2 h. The title compound was purified by simple distillation (vacuum transfer) and obtained as a colorless liquid (22.7 g, 164 mmol, 82 %).

^{1}H NMR (300 MHz, $CDCl_3$): δ/ppm = 3.81 (q, *J* = 7.0 Hz, 2H), 1.23 (t, *J* = 7.0 Hz, 3H), 0.46 (s, 6H). **^{1}H/^{29}Si HMQC NMR** (300/60 MHz, $CDCl_3$): δ/ppm = 0.46/13.7.

Chloro(isopropoxy)dimethylsilane

Cl—$SiMe_2O$*i*Pr

$C_5H_{13}ClOSi$

mw: 152.69 g/mol

A flame-dried, two-necked round-bottomed flask equipped with a stirring bar and a rubber septum was charged with urea (12.62 g, 210 mmol, 1.05 eq.) and dichlorodimethylsilane (6.45 mL, 200 mmol, 1 eq.). The resulting suspension was cooled to 0 °C and isopropanol (16.08 mL, 210 mmol, 1.05 eq.) was then added dropwise. The reaction mixture was allowed to warm to room temperature and stirred for 2 h. The title compound was purified by simple distillation (vacuum transfer) and obtained as a colorless liquid (27.2 g, 178 mmol, 89 %).

^{1}H NMR (300 MHz, $CDCl_3$): δ/ppm = 4.23 (hept, *J* = 6.1 Hz, 1H), 1.21 (d, *J* = 6.1 Hz, 6H), 0.46 (s, 6H). **^{1}H/^{29}Si HMQC NMR** (300/60 MHz, $CDCl_3$): δ/ppm = 0.46/11.1.

Tert-butoxychlorodimethylsilane

Cl—SiMe$_2$O*t*Bu

$C_6H_{15}ClOSi$
mw: 166.72 g/mol

A flame-dried, two-necked round-bottomed flask equipped with a stirring bar and a rubber septum was sequentially charged with urea (3.47 g, 57.8 mmol, 1.05 eq.), dichlorodimethylsilane (6.7 mL, 55 mmol, 1 eq.) and THF (78 mL, 0.7 M). The resulting mixture was cooled to 0 °C and *tert*-butanol (5.5 mL, 57.8 mmol, 1.05 eq.) was then added dropwise. The reaction mixture was allowed to warm to room temperature and stirred for 2 h. The title compound was isolated as a solution in THF (0.7 M) by simple distillation (vacuum transfer) and used without further processing in the hydrazine synthesis step (*vide infra*).

^{1}H NMR (300 MHz, $CDCl_3$): δ/ppm = 1.30 (s, 9H), 0.41 (s, 6H). **^{1}H/^{29}Si HMQC NMR** (300/60 MHz, $CDCl_3$): δ/ppm = 0.41/3.3. The spectroscopic data match the literature report.[9]

Di-tert-butoxychloro(methyl)silane

Cl—SiMe(O*t*Bu)$_2$

$C_9H_{21}ClO_2Si$
mw: 224.80 g/mol

A flame-dried, two-necked round-bottomed flask equipped with a stirring bar and a rubber septum was sequentially charged with urea (7.4 g, 123 mmol, 2.05 eq.), methyltrichlorosilane (7.1 mL, 60 mmol, 1 eq.) and THF (60 mL, 1M). The resulting mixture was cooled to -8 °C and *tert*-butanol (11.8 mL, 123 mmol, 2.05 eq.) was then added dropwise. The resulting mixture were allowed to warm to room temperature and stirred for ca. 16 h. The title compound was purified by simple distillation (vacuum transfer) and obtained as a colorless liquid (12.2 g, 54.6 mmol, 91 %).

^{1}H NMR (300 MHz, $CDCl_3$) δ/ppm 1.36 (s, 18H), 0.42 (s, 3H) **^{1}H/^{29}Si HMQC NMR** (300/60 MHz, $CDCl_3$): δ/ppm = 0.42/-43.2. The spectroscopic data match the literature report.[10]

5.2. Synthesis and characterization of monoalkoxysilyldiazenes **3a**, **3b** and **3c**

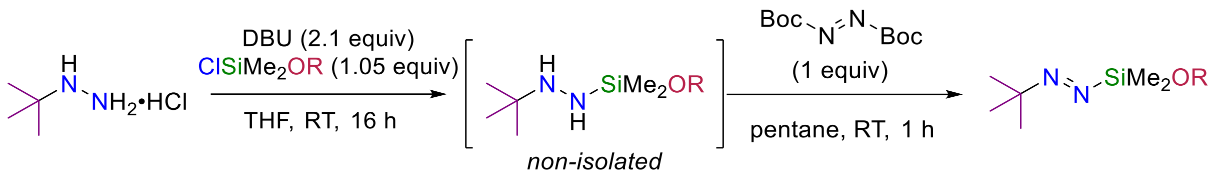

GP1: A flame-dried two-necked round-bottomed flask equipped with a stirring bar and a rubber septum was charged with finely ground and thoroughly dried *tert*-butylhydrazine hydrochloride (1 eq.), THF (20 mL) and DBU (2.1 eq). Under vigorous stirring, a solution of the chloro(alkoxy)dimethylsilane (1.1 eq) in THF (0.7 M) was then added dropwise at room temperature. The reaction mixture was further stirred at room temperature for 16 h, time after which full conversion of the chlorosilane was confirmed by $^{1}H/^{29}Si$ HMQC NMR. The crude suspension was cooled to 0 °C, then cold degassed pentane (40 mL) was added to precipitate most of the hydrochloride salt. The resulting suspension was then filtered under air and the solid washed with *n*-pentane (40 mL). The filtrate, collected in a Schlenk flask, was concentrated *in vacuo* by rotary evaporation to afford the crude silylated hydrazine, which was directly used in the oxidation step without further purification.

Note: the silylated hydrazines were of sufficient purity to be fully characterized by multinuclear NMR spectroscopy.

To the Schlenk flask containing the crude hydrazine was added *n*-pentane (98%, degassed by argon bubbling, 0.7 M). To the resulting solution was added di-*tert*-butylazodicarboxylate (DBAD, 1.0 eq. assuming 100 % yield from the previous reaction) in one portion by quickly opening the rubber septum under a positive pressure of argon. The resulting orange suspension was vigorously stirred at room temperature for 1 h, time after which the reaction mixture was deep red in color. The volatiles were removed *in vacuo* by rotary evaporation to afford a red slurry. The diazene was then separated from the solid residue ($[BocNH]_2$) by simple distillation (vacuum transfer) under dynamic vacuum.

(E)-1-(*tert*-butoxydimethylsilyl)-2-(*tert*-butyl)diazene (3a)

Prepared according to **GP1** from *tert*-butylhydrazine hydrochloride (6.23 g, 50.0 mmol, 1.0 eq.), DBU (15.7 mL, 105.0 mmol, 2.1 eq.) and $ClSiMe_2(OtBu)$ (9.17 g, 55.0 mmol, 1.1 eq.) followed by the addition of di-*tert*-butylazodicarboxylate (11.52 g, 50.0 mmol, 1 eq.). Careful removal of *n*-pentane by rotary evaporation followed by dynamic vacuum transfer afforded the title compound **3b** as a deep red liquid (9.14 g, 42.24 mmol, 84% over 2 steps).

Note: $Me_2Si(OtBu)_2$ (4 %) was identified as a side-product; corrected yield = 79% over 2 steps.

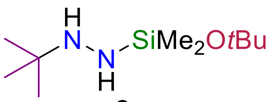

2a

$C_{10}H_{26}N_2OSi$

mw: 218.42 g/mol

^{1}H NMR (300 MHz, $CDCl_3$): δ/ppm = 1.26 (s, 9H), 0.98 (s, 9H), 0.07 (s, 6H). **^{13}C{^{1}H} NMR** (75 MHz, $CDCl_3$): δ/ppm = 71.7, 53.4, 32.2, 27.0, -0.3. **^{1}H/^{29}Si HMQC NMR** (300/60 MHz, $CDCl_3$): δ/ppm = 0.07/-11.4.

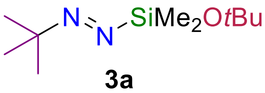

3a

$C_{10}H_{24}N_2OSi$

mw: 216.40 g/mol

^{1}H NMR (300 MHz, $CDCl_3$): δ/ppm = 1.32 (s, 9H), 1.17 (s, 9H), 0.18 (s, 6H). **^{13}C{^{1}H} NMR** (75 MHz, $CDCl_3$): δ/ppm = 73.92, 73.81, 32.24, 25.93, -1.69. **^{1}H/^{29}Si HMQC NMR** (300/60 MHz, $CDCl_3$): δ/ppm = 0.18/-8.4. *Note:* HRMS could not be obtained for this compound as ionization using APCI technique led to unidentified ions.

(E)-1-(*tert*-butyl)-2-(*iso*propoxydimethylsilyl)diazene (3b)

Prepared according to GP1 from *tert*-butylhydrazine hydrochloride (6.23 g, 50.0 mmol, 1.0 eq.), DBU (15.7 mL, 105.0 mmol, 2.1 eq.) and $ClSiMe_2(OiPr)$ (8.40 g, 55.0 mmol, 1.1 eq.) followed by the addition of di-*tert*-butylazodicarboxylate (11.52 g, 50.0 mmol, 1 eq.). Careful removal of *n*-pentane by rotary evaporation followed by dynamic vacuum transfer afforded the title compound **3b** as a deep red liquid (7.64 g, 35.75 mmol, 75% over 2 steps).

Note: $Me_2Si(OiPr)_2$ (7 %) was identified as a side-product; corrected yield = 68% over 2 steps.

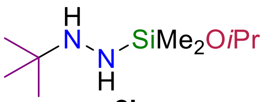

2b

$C_9H_{24}N_2OSi$

mw: 204.39 g/mol

^{1}H NMR (300 MHz, $CDCl_3$): δ/ppm = 4.07 (hept, *J* = 6.1 Hz, 1H), 1.14 (d, *J* = 6.1 Hz, 6H), 0.98 (s, 9H), 0.06 (s, 6H). **^{13}C{^{1}H} NMR** (75 MHz, $CDCl_3$): δ/ppm = 64.3, 53.3, 26.9, 25.9, -2.8. **^{1}H/^{29}Si HMQC NMR** (300/60 MHz, $CDCl_3$): δ/ppm = 0.06/-5.9.

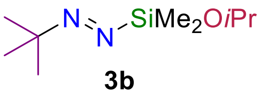

3b

$C_9H_{22}N_2OSi$

mw: 202.37 g/mol

^{1}H NMR (300 MHz, $CDCl_3$): δ/ppm = 4.27 (hept, *J* = 6.1 Hz, 1H), 1.21 (d, *J* = 6.1 Hz, 6H), 1.17 (s, 9H), 0.22 (s, 6H). **^{13}C{^{1}H} NMR** (75 MHz, $CDCl_3$): δ/ppm = 73.9, 65.8, 25.6, 25.4, -4.2. **^{1}H/^{29}Si HMQC NMR** (300/60 MHz, $CDCl_3$): δ/ppm = 0.22/-4.7. **HRMS (ESI)** m/z: $[M+H]^+$ Calcd for $C_9H_{22}N_2OSiH$ 203.1574. Found 203.1572.

(*E*)-1-(*tert*-butyl)-2-(ethoxydimethylsilyl)diazene (3c)

Prepared according to GP1 from *tert*-butylhydrazine hydrochloride (6.23 g, 50 mmol, 1.0 eq.), DBU (15.7 mL, 105 mmol, 2.1 eq.) and $ClSiMe_2OEt$ (7.63 g, 55 mmol, 1.1 eq.) followed by the addition of di-*tert*-butylazodicarboxylate (11.52 g, 50.0 mmol, 1 eq.). Careful removal of *n*-pentane by rotary evaporation followed by dynamic vacuum transfer afforded the title compound **3c** as a deep red liquid (5.50 g, 29.2 mmol, 58 % over 2 steps).

Note: $Me_2Si(OEt)_2$ (3 %) was identified as a side-product; corrected yield = 55 % over 2 steps.

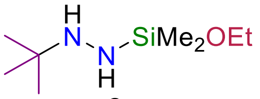

2c

$C_8H_{22}N_2OSi$

mw: 190.36 g/mol

^{1}H NMR (300 MHz, $CDCl_3$): δ/ppm = 3.71 (q, *J* = 7.0 Hz, 2H), 1.19 (t, *J* = 7.0 Hz, 3H), 1.00 (s, 9H), 0.08 (s, 6H). **^{13}C{^{1}H} NMR** (75 MHz, $CDCl_3$): δ/ppm = 58.0, 53.5, 26.9, 18.6, -3.4. **^{1}H/^{29}Si HMQC NMR** (300/60 MHz, $CDCl_3$): δ/ppm = 0.08/-4.3.

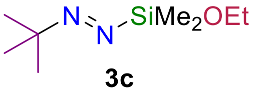

3c

$C_8H_{20}N_2OSi$

mw: 188.35 g/mol

^{1}H NMR (300 MHz, $CDCl_3$): δ/ppm = 3.89 (q, *J* = 7.0 Hz, 2H), 1.24 (t, *J* = 7.0 Hz, 3H), 1.17 (s, 9H), 0.23 (s, 6H). **^{13}C{^{1}H} NMR** (75 MHz, $CDCl_3$): δ/ppm = 74.5, 59.4, 26.0, 18.6, -4.3. **^{1}H/^{29}Si HMQC NMR** (300/60 MHz, $CDCl_3$): δ/ppm = 0.24/-3.0. **HRMS (ESI)** m/z: $[M+H]^+$ Calcd for $C_8H_{20}N_2OSiH$ 189.1418. Found 18.1415

5.3. Synthesis and characterization of bis(*tert*-butoxy)silyldiazene **3d**

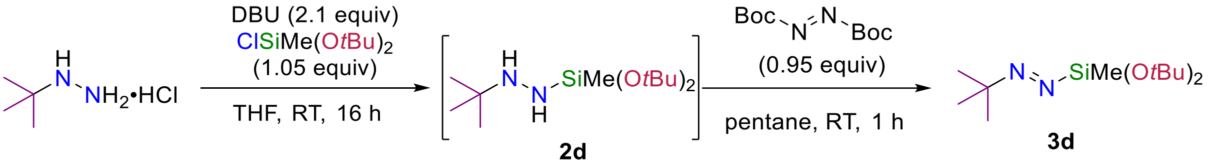

Hydrazine preparation: A flame-dried two-necked round-bottomed flask equipped with a stirring bar and a rubber septum was charged with finely ground and thoroughly dried *tert*-butylhydrazine hydrochloride (2.49 g, 20 mmol,1 eq.), THF (28 mL, 0.7 M) and DBU (6.4 mL, 42 mmol, 2.1 eq.). The resulting white suspension was vigorously stirred and neat ClSiMe(O*t*Bu)$_2$ (4.95 g, 22 mmol, 1.1 eq.) was then added dropwise at room temperature. The reaction mixture was stirred for 16 h, cooled to 0 °C, then cold degassed pentane (40 mL) was added to precipitate most of the hydrochloride salt. The resulting suspension was then filtered under air and the solid washed with *n*-pentane (40 mL). The filtrate, collected in a flame-dried Schlenk flask, was concentrated *in vacuo* by rotary evaporation to afford the crude silylated hydrazine **2d**, which was directly used in the oxidation step without further purification.

Oxidation: To the Schlenk flask containing the crude hydrazine **2d** was added *n*-pentane (28 mL, 0.7 M). The resulting solution was cooled to 0 °C and a solution of di-*tert*-butyl azodicarboxylate (3.68 g, 16 mmol, 0.8 eq.) in CH_2Cl_2 (5 mL) was then added dropwise. After 10 min of stirring at 0 °C, a second portion of di-*tert*-butyl azodicarboxylate (0.69 g, 3.0 mmol, 0.15 eq. in 1 mL CH_2Cl_2) was added to oxidize the remaining quantity of hydrazine **2d** (as judged by ^{1}H NMR spectroscopy). The resulting deep red reaction mixture was further stirred for 10 min and then concentrated *in vacuo* by rotary evaporation to remove ca. half of the pentane. The resulting suspension was cooled to -20 °C and filtered twice to get rid of the solid byproduct ([BocNH]$_2$), which was washed with cold pentane (-20 °C). The filtrate was concentrated by rotary evaporation and the crude red-colored residue was purified by flash

column chromatography on silica gel using PE/Et_3N (96:4 v/v) as eluent to afford the title compound **3d** as a deep red liquid (4.38 g, 16.0 mmol, 80 % yield over 2 steps).

Note: To facilitate the purification process, it is preferable to carefully adjust the amount of di-*tert*-butyl azodicarboxylate to match the exact quantity of the crude hydrazine, adding it portion-wise. We indeed observed that even a slight excess of di-*tert*-butyl azodicarboxylate can co-elute with the diazene during column chromatography.

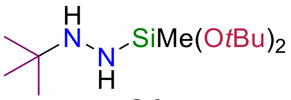

2d

$C_{13}H_{32}N_2O_2Si$

mw: 276.50 g/mol

^{1}H NMR (300 MHz, $CDCl_3$): δ/ppm = 1.29 (s, 18H), 1.01 (s, 9H), 0.16 (s, 3H). **^{13}C{^{1}H} NMR** (75 MHz, $CDCl_3$): δ/ppm = 71.8, 53.4, 31.8, 27.0, -1.0. **^{1}H/^{29}Si HMQC NMR** (300/60 MHz, $CDCl_3$): δ/ppm = 0.16/-43.8.

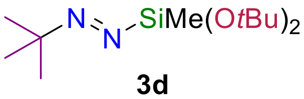

3d

$C_{13}H_{30}N_2O_2Si$

mw: 274.48 g/mol

^{1}H NMR (300 MHz, $CDCl_3$): δ/ppm = 1.31 (s, 18H), 1.18 (s, 9H), 0.12 (s, 3H). **^{13}C{^{1}H} NMR** (75 MHz, $CDCl_3$): δ/ppm = 73.9, 73.7, 32.1, 25.9, -3.6. **^{1}H/^{29}Si HMQC NMR** (300/60 MHz, $CDCl_3$): δ/ppm = 0.12/-49.6. **HRMS (ESI)** m/z: $[M+H]^+$ Calcd for $C_{13}H_{30}N_2O_2SiH$ 275.2149. Found 275.2150.

5.4. Synthesis and characterization of tris(*tert*-butoxy)silyldiazene **3e**

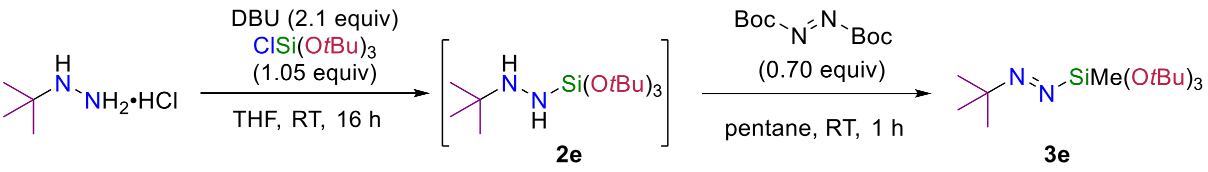

<u>Hydrazine preparation</u>: A flame-dried two-necked round-bottomed flask equipped with a stirring bar and a rubber septum was charged with finely ground and thoroughly dried *tert*-butylhydrazine hydrochloride (0.98 g, 7.9 mmol,1 eq.), THF (11 mL, 0.7 M) and DBU (2.5 mL, 16.6 mmol, 2.1 eq.). The resulting white suspension was vigorously stirred and neat $ClSi(OtBu)_3$ (2.46 g, 8.7 mmol, 1.1 eq.) was then added dropwise at room temperature. The reaction mixture was stirred for 43 h (conversion monitored by GC/MS), cooled to 0 °C, then cold degassed pentane (10 mL) was added to precipitate most of the hydrochloride salt. The resulting suspension was then filtered under air and the solid washed with cold *n*-pentane (20 mL). The filtrate, collected in a flame-dried Schlenk flask, was concentrated *in vacuo* by rotary evaporation to afford the crude silylated hydrazine **2e**, which was directly used in the oxidation step without further purification.

<u>Oxidation</u>: To the Schlenk flask containing the crude hydrazine **2e** was added *n*-pentane (11 mL, 0.7 M). The resulting solution was cooled to 0 °C and a solution of di-*tert*-butyl

azodicarboxylate (1.09 g, 4.74 mmol, 0.6 eq.) in CH_2Cl_2 (1.5 mL) was then added dropwise. After 10 min of stirring at 0 °C, a second portion of di-*tert*-butyl azodicarboxylate (0.18 g, 0.79 mmol, 0.1 eq. in 0.5 mL CH_2Cl_2) was added to oxidize the remaining quantity of hydrazine **2e** (as judged by ^{1}H NMR spectroscopy). The resulting deep red reaction mixture was further stirred for 10 min and then concentrated *in vacuo* by rotary evaporation to remove ca. half of the pentane. The resulting suspension was cooled to -20 °C and filtered twice to get rid of the solid byproduct ($[BocNH]_2$), which was washed with cold pentane (-20 °C). The filtrate was concentrated by rotary evaporation and the crude red-colored residue was purified by flash column chromatography on silica gel using PE/Et_3N (96:4 v/v) as eluent to afford the title compound **3e** as a deep red liquid (1.66 g, 5 mmol, 63% over 2 steps).

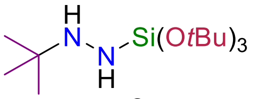

2e

$C_{16}H_{38}N_2O_3Si$

mw: 334.58 g/mol

^{1}H NMR (300 MHz, $CDCl_3$): δ/ppm = 1.30 (s, 27H), 1.03 (s, 9H). **^{13}C{^{1}H} NMR** (75 MHz, $CDCl_3$): δ/ppm = 72.4, 53.7, 31.7, 27.4.

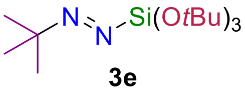

3e

$C_{16}H_{36}N_2O_3Si$

mw: 332.56 g/mol

^{1}H NMR (300 MHz, $CDCl_3$): δ/ppm = 1.29 (s, 27H), 1.19 (s, 9H). **^{13}C{^{1}H} NMR** (75 MHz, $CDCl_3$): δ/ppm = 74.0, 73.6, 31.7, 25.8. **^{29}Si NMR** (60 MHz, $CDCl_3$): δ/ppm = -95.2. **HRMS (ESI)** m/z: $[M+H]^+$ Calcd for $C_{16}H_{36}N_2O_3SiH$ 333.2568. Found 333.2569.

5.5. Stability of diazenes **3a-d** under basic conditions

<u>Procedure</u>*:* In a glove box, a 2-mL vial equipped with a magnetic stirring bar was charged with *t*BuOK (1.1 mg, 10 mol%) and THF (0.4 mL). Another 2-mL dram vial was charged with tetracosane, *t*BuN_2*Si* (0.1 mmol, *Si* as described in Figure S5) and THF (0.3 mL). An aliquot (ca. 10 µL) was withdrawn from the latter solution, diluted with 1 mL of *n*-heptane and submitted to GC/MS analysis to set the initial tetracosane/diazene ratio. The diazene solution was then added dropwise to the vigorously stirred solution of *t*BuOK at room temperature. Aliquot of the resulting reaction mixture were withdrawn at different times, diluted with 1 mL of *n*-heptane and submitted to GC/MS analysis.

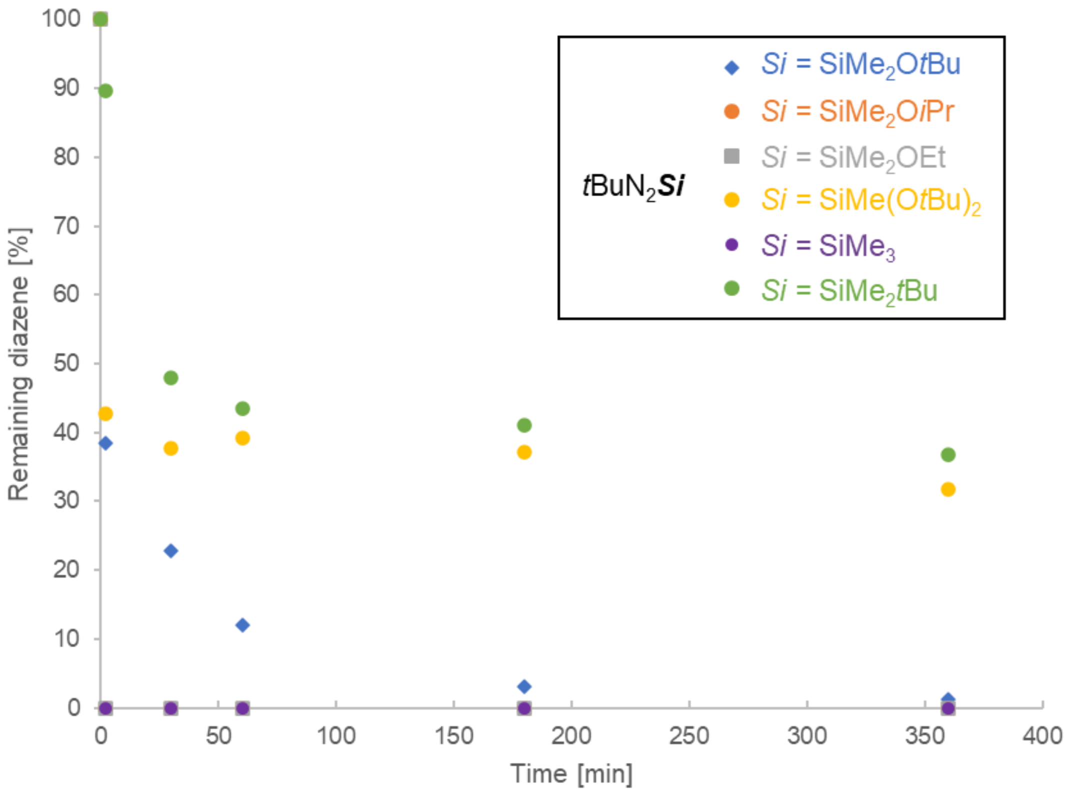

Figure S5: Stability of various alkoxysilyldiazenes under basic conditions in THF (tBuOK, 10 mol%). Note that the data points for **3b**, **3c** and *t*BuN_2SiMe_3 overlap and therefore cannot be distinguished.

As can be deduced from the plot in Figure S5, all the monoalkoxysilyldiazenes (**3a-c**) as well as the bis-*tert*-butoxysilyldiazenes (**3d**) were found reactive towards a catalytic amount of *t*BuOK (10 mol with respect to each diazene) in the absence of a hydrocarbon substrate. While the lightest congeners **3b** and **3c** were fully decomposed in less than 2 min under such basic conditions, slightly increased stability was observed for **3a** as its complete decomposition took ca. 3 h at room temperature. The behavior of the bulkiest diazene **3d** was more contrasted as ca. 58 % rapidly decomposed upon mixing, but the remaining quantity was found to decrease

only slowly (only 6 % decomposed between 30 min and 360 min of reaction). As far as the monoalkoxysilyldiazenes are concerned, the major decomposition products identified by GC/MS include the following compounds (R = *t*Bu, *i*Pr or Et):

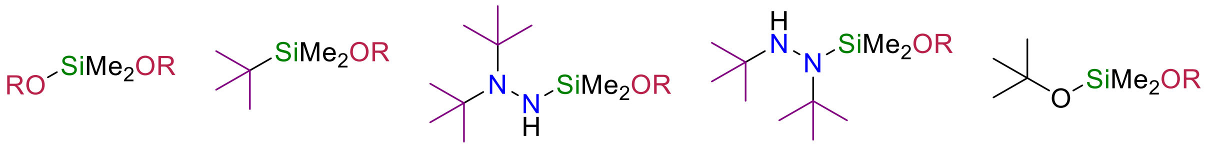

The stability trends discussed above are similar to those observed for the trialkylsilyl-substituted diazenes bearing Me_3Si or bulkier $tBuMe_2Si$ groups, stability of which under basic conditions primarily depends on the steric bulkiness of the silyl group.

6. Synthesis and characterization of silylated products

6.1. General procedures

GP2 (silylation of C(sp^2)–H bonds): A 10-mL vial equipped with a magnetic stirring bar was charged with potassium *tert*-butoxide (10 to 40 mol%), THF (2.0 mL) and the corresponding substrate (0.5 mmol, prior to adding the solvent when solid). To the resulting vigorously stirred mixture was then added dropwise a solution of the corresponding silylated *tert*-butyldiazene in THF (1.5 mL). The reaction mixture was stirred at room temperature for the indicated time, concentrated by rotary evaporation and the resulting residue was directly purified by flash column chromatography on silica gel.

GP3 (silylation of benzylic C(sp^3)–H bonds): A 5-mL microwave vial equipped with a magnetic stirring bar was charged with potassium *tert*-butoxide, THF (0.5 mL, 1 M) and the corresponding substrate (0.5 mmol, prior to adding the solvent). To the resulting vigorously stirred mixture was then added neat the corresponding silylated *tert*-butyldiazene in one portion. After stirring at room temperature for the indicated time, the reaction mixture was concentrated by rotary evaporation and the resulting crude residue was directly purified by column chromatography on silica gel.

Caution: the silylation reaction with *tert*-butyl silyldiazenes rapidly generates gaseous byproducts, including N_2. Reactions must therefore be carried out in well-vented open systems or using pressure-proof glassware while applying the appropriate safety procedures.

6.2. Characterization data for silylated (hetero)arenes

2-(*tert*-butoxydimethylsilyl)-1-methyl-1*H*-indole

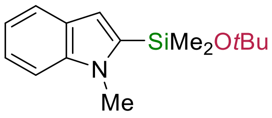

4a
$C_{15}H_{23}NOSi$
mw: 261.44 g/mol

Prepared according to **GP2** from the corresponding diazene **3a** (195 mg, 0.90 mmol, 1.8 eq.), 1-methyl-1*H*-indole (62.5 µL, 0.50 mmol, 1.0 eq.) and potassium *tert*-butoxide (5.6 mg, 10 mol%, 0.1 eq). The reaction mixture was stirred for 1 h at room temperature. Purification by flash column chromatography on silica gel using PE then PE/EtOAc 99:1 as eluent afforded the title compound **4a** (119.1 mg, 0.46 mmol, 91 %) as a yellowish solid.

M.p (CH_2Cl_2) = 46-48 °C. **^{1}H NMR** (300 MHz, $CDCl_3$): δ/ppm = 7.62 (d, J = 1.2 Hz, 1H), 7.34 (d, J = 8.4 Hz, 1H), 7.28 – 7.20 (m, 1H), 7.08 (dd, J = 8.1, 6.9 Hz, 1H), 6.71 (s, 1H), 3.95 (s,

3H), 1.26 (s, 9H), 0.50 (s, 6H). **^{13}C{^{1}H} NMR** (75 MHz, $CDCl_3$): δ/ppm = 141.5, 140.2, 122.2, 121.0, 119.2, 111.2, 109.3, 73.5, 33.1, 32.0, 2.0. **^{1}H/^{29}Si HMQC NMR** (400/79 MHz, $CDCl_3$): δ/ppm = 0.50/-8.4. **HRMS (ESI)** m/z: $[M+H]^+$ Calcd for $C_{15}H_{23}NOSiH$ 262.1622. Found 262.1623.

2-(*iso*propoxydimethylsilyl)-1-methyl-1*H*-indole

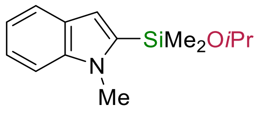

4b
$C_{14}H_{21}NOSi$
mw: 247.41 g/mol

Prepared according to **GP2** from the corresponding diazene **3b** (394 mg, 1.5 mmol, 3.0 eq.), 1-methyl-1*H*-indole (62.5 µL, 0.50 mmol, 1.0 eq.) and potassium *tert*-butoxide (5.6 mg, 10 mol%). The reaction mixture was stirred for 2 h. Purification by flash column chromatography on silica gel using PE/CH_2Cl_2 (100:0 → 95:5 → 90:10→ 80:20 → 70:30 v/v) as eluent afforded the title compound **4b** (60.7 mg, 0.25 mmol, 49 %) as a yellowish oil.

Note: **4b** slightly decomposed on silica gel (NMR yield: 73 %).

^{1}H NMR (300 MHz, $CDCl_3$): δ/ppm = 7.64 (dd, J = 7.9, 1.0 Hz, 1H), 7.36 (dt, J = 8.3, 1.0 Hz, 1H), 7.32 – 7.20 (m, 1H), 7.10 (ddt, J = 7.9, 6.9, 1.0 Hz, 1H), 6.75 (s, 1H), 4.04 (hept, J = 0.9 Hz, 1H), 3.94 (s, 3H), 1.15 (dd, J = 6.1, 0.9 Hz, 6H), 0.51 (s, 6H). **^{13}C{^{1}H} NMR** (75 MHz, $CDCl_3$): δ/ppm = 140.3, 139.4, 128.4, 122.5, 121.1, 119.3, 112.5, 109.4, 65.8, 33.1, 25.7, -0.4. **^{1}H/^{29}Si HMQC NMR** (300/60 MHz, $CDCl_3$): δ/ppm = 0.50/-1.4. **HRMS (ESI)** m/z: $[M+H]^+$ Calcd for $C_{14}H_{21}NOSiH$ 248.1465. Found 248.1466.

2-(ethoxydimethylsilyl)-1-methyl-1*H*-indole

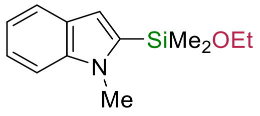

4c
$C_{13}H_{19}NOSi$
mw: 233.39 g/mol

Prepared according to **GP2** from the corresponding diazene **3c** (57 mg, 0.30 mmol, 3.0 eq.), 1-methyl-1*H*-indole (12.5 µL, 0.10 mmol, 1.0 eq.) and potassium *tert*-butoxide (1.1 mg, 10 mol%). The reaction mixture was stirred for 1 h at room temperature. 1,3,5- trimethoxybenzene was then added to the crude mixture, which was then concentrated by rotary evaporation and analyzed by ^{1}H NMR spectroscopy. NMR yield = 63 %.

The title compound could be *in-situ* characterized using NMR spectroscopy.

^{1}H NMR (300 MHz, $CDCl_3$): δ/ppm = 7.64 (d, *J* = 8.1 Hz, 1H), 7.36 (d, *J* = 8.2 Hz, 1H), 7.26 (d, *J* = 7.9 Hz, 1H), 7.11 (d, *J* = 7.0 Hz, 1H), 6.77 (s, 1H), 3.93 (s, 3H), 3.69 (q, *J* = 7.1 Hz, 2H), 1.20 (t, *J* = 7.2 Hz, 3H), 0.50 (s, 6H). **^{13}C{^{1}H} NMR** (75 MHz, $CDCl_3$): δ/ppm = 140.4, 138.9, 128.3, 122.6, 121.6, 121.1, 119.3, 112.8, 58.9, 30.3, 18.5, -0.9. **^{1}H/^{29}Si HMQC NMR** (300/60 MHz, $CDCl_3$): δ/ppm = 0.50/1.4. The spectroscopic data match the literature report.[3]

2-(di-*tert*-butoxy(methyl)silyl)-1-methyl-1*H*-indole

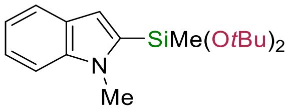

4d
$C_{18}H_{29}NO_2Si$
mw: 319.52 g/mol

Prepared according to **GP2** from the corresponding diazene **3d** (302 mg, 1.1 mmol, 2.2 eq.), 1-methyl-1*H*-indole (62.5 µL, 0.50 mmol, 1.0 eq.) and potassium *tert*-butoxide (5.6 mg, 10 mol%). The reaction mixture was stirred overnight. Purification by flash column chromatography on deactivated silica gel (1% Et_3N) using PE/EtOAc (100:0 → 99:1 v/v) as eluent afforded the title compound **4d** (165 mg, 0.50 mmol, >99 %) as a yellow oil.

^{1}H NMR (300 MHz, $CDCl_3$): δ/ppm = 7.64 (d, *J* = 7.8 Hz, 1H), 7.36 (d, *J* = 8.3 Hz, 1H), 7.25 (t, *J* = 8.2 Hz, 1H), 7.09 (t, *J* = 7.3 Hz, 1H), 6.79 (s, 1H), 3.98 (s, 3H), 1.34 (s, 18H), 0.58 (s, 3H). **^{13}C{^{1}H} NMR** (75 MHz, $CDCl_3$): δ/ppm = 140.6, 140.1, 128.4, 122.1, 121.0, 119.0, 112.1, 109.4, 73.6, 33.1, 32.0, 2.4. **^{1}H/^{29}Si HMQC NMR** (300/60 MHz, $CDCl_3$): δ/ppm = 0.58/-89.0. **HRMS (ESI)** m/z: $[M+H]^+$ Calcd for $C_{18}H_{29}NO_2SiH$ 320.2040. Found 320.2041.

1-methyl-2-(tri-*tert*-butoxysilyl)-1*H*-indole

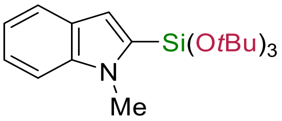

4e
$C_{21}H_{35}NO_3Si$
mw: 377.60 g/mol

Prepared according to **GP2** from the corresponding diazene **3e** (293 mg, 0.88 mmol, 2.2 eq.), 1-methyl-1*H*-indole (50 µL, 0.40 mmol, 1.0 eq.) and potassium *tert*-butoxide (18 mg, 40 mol%, 0.4 eq.). The reaction mixture was stirred for 24 h. Purification by flash column chromatography on silica gel using PE/CH_2Cl_2 (95:5 → 90:10, v/v) as eluent afforded the title compound **4e** (68.9 mg, 0.19 mmol, 46 %) as a light-yellow solid.

Note: The same reaction carried out on a smaller scale (0.1 mmol) gave **4e** in 68 % yield as determined by ^{1}H NMR spectroscopy using 1,3,5-trimethoxybenzene as internal standard.

M.p (CH_2Cl_2) = 71-73 °C. **^{1}H NMR** (300 MHz, $CDCl_3$): δ/ppm = 7.64 (d, *J* = 7.7 Hz, 1H), 7.35 (d, *J* = 8.1 Hz, 1H), 7.23 (ddd, *J* = 8.2, 7.1, 1.4 Hz, 1H), 7.08 (ddd, *J* = 7.9, 6.9, 1.1 Hz, 1H), 6.91 (s, 1H), 3.97 (s, 3H), 1.42 (s, 27H). **^{13}C{^{1}H} NMR** (75 MHz, $CDCl_3$): δ/ppm = 139.9, 139.1, 128.2, 122.0, 121.0, 118.9, 113.2, 109.4, 74.1, 33.2, 32.0. **^{1}H/^{29}Si HMQC NMR** (300/60 MHz, $CDCl_3$): δ/ppm = 6.91/-182.5. **HRMS (ESI)** m/z: $[M+H]^+$ Calcd for $C_{21}H_{35}NO_3SiH$ 378.2459. Found 378.2460.

Dimethylbis(1-methyl-1*H*-indol-2-yl)silane

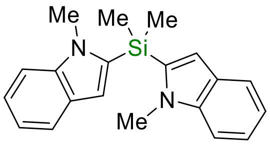

5
$C_{20}H_{22}N_2Si$
mw: 318.50 g/mol

Prepared according to **GP2** from diazene **3c** (236 mg, 1.25 mmol, 2.5 eq.), 1-methyl-1*H*-indole (62.5 µL, 0.50 mmol, 1.0 eq.) and potassium *tert*-butoxide (28.1 mg, 50 mol%). The reaction mixture was stirred for 1 h. Purification by flash column chromatography on silica gel using PE/CH_2Cl_2 (95:5 → 90:10, v/v) as eluent afforded the title compound **5** (37.9 mg, 0.12 mmol, 48 %) as a yellow solid. Crystals suitable for X-ray diffraction were obtained by slow evaporation of a concentrated solution of **5** in $CDCl_3$.

M.p (CH_2Cl_2) = 103-105°C. **^{1}H NMR** (300 MHz, $CDCl_3$): δ/ppm = 7.68 (dt, *J* = 7.9, 1.0 Hz, 2H), 7.31 (m, 2H), 7.26 (ddd, *J* = 8.3, 6.9, 1.2 Hz, 2H), 7.13 (ddd, *J* = 7.9, 6.7, 1.3 Hz, 2H), 6.90 (s, 2H), 3.64 (s, 6H), 0.78 (s, 6H). **^{13}C{^{1}H} NMR** (75 MHz, CDCl3): δ/ppm = 140.6, 138.1, 128.5, 122.5, 121.0, 119.5, 113.0, 109.4, 32.7, - 1.4. **^{1}H/^{29}Si HMQC NMR** (300/60 MHz, $CDCl_3$): δ/ppm = 0.78/-23.3. **HRMS (ESI)** m/z: $[M+H]^+$ Calcd for $C_{20}H_{22}N_2SiH$ 319.1625. Found 319.1619.

2-(*tert*-butoxydimethylsilyl)-1-methyl-4-methoxy-1*H*-indole

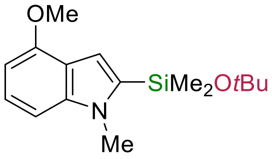

6a
$C_{16}H_{25}NO_2Si$
mw: 291.47 g/mol

Prepared according to **GP2** from the corresponding diazene **3a** (195 mg, 0.90 mmol, 1.8 eq.), 4-methoxy-1-methyl-1*H*-indole (80.6 mg, 0.50 mmol, 1.0 eq.) and potassium *tert*-butoxide (5.6 mg, 10 mol%). The reaction mixture was stirred for 2 h. Purification by flash column

chromatography on silica gel using PE/EtOAc (100:0 → 99:1 v/v) as eluent afforded the title compound **6a** (148.7 mg, 0.50 mmol, >99 %) as a white solid.

M.p (CH_2Cl_2) = 65-67 °C**.** ^{1}**H NMR** (400 MHz, $CDCl_3$): δ/ppm = 7.17 (dd, *J* = 8.3, 7.7 Hz, 1H), 6.98 (d, *J* = 8.3 Hz, 1H), 6.82 (s, 1H), 6.50 (d, *J* = 7.6 Hz, 1H), 3.98 (s, 3H), 3.94 (s, 3H), 1.25 (s, 9H), 0.49 (s, 6H). ^{13}**C{**^{1}**H} NMR** (101 MHz, $CDCl_3$): δ/ppm = 153.5, 141.8, 140.0, 123.12, 119.1, 108.3, 102.9, 73.5, 55.4, 33.4, 32.0, 1.9. ^{1}**H/**^{29}**Si HMQC NMR** (400/79 MHz, $CDCl_3$): δ/ppm = 0.49/-8.5. **HRMS (ESI)** m/z: $[M+H]^+$ Calcd for $C_{16}H_{25}NO_2SiH$ 292.1727. Found 292.1728.

2-(*tert*-butoxydimethylsilyl)-6-methoxy-1-methyl-1*H*-indole

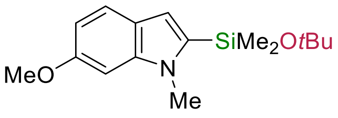

7a
$C_{16}H_{25}NO_2Si$
mw: 291.47 g/mol

Prepared according to **GP2** from the corresponding diazene **3a** (195 mg, 0.90 mmol, 1.8 eq.) and 1-methyl-6-methoxy-1*H*-indole (80.6 mg, 0.50 mmol, 1.0 eq.) and potassium *tert*-butoxide (5.6 mg, 10 mol%). The reaction mixture was stirred for 2 h. Purification by flash column chromatography on silica gel using PE/EtOAc (100:0 → 99:1 v/v) as eluent afforded the title compound **7a** (114.8 mg, 0.40 mmol, 79 %) as a white solid.

M.p (CH_2Cl_2) = 81-83 °C. ^{1}**H NMR** (400 MHz, $CDCl_3$): δ/ppm = 7.52 – 7.48 (m, 2H), 6.78 (d, *J* = 7.9 Hz, 1H), 6.65 (d, *J* = 0.8 Hz, 1H), 3.91 (s, 6H), 1.27 (s, 9H), 0.49 (s, 6H). ^{13}**C{**^{1}**H} NMR** (101 MHz, $CDCl_3$): δ/ppm = 156.9, 141.0, 140.3, 122.9, 121.6, 111.3, 109.6, 92.5, 73.4, 55.8, 33.2, 32.0, 1.9. ^{1}**H/**^{29}**Si HMQC NMR** (400/79 MHz, $CDCl_3$): δ/ppm = 0.49/-8.8. **HRMS (ESI)** m/z: $[M+H]^+$ Calcd for $C_{16}H_{25}NO_2SiH$ 292.1727. Found 292.1726.

2-(*tert*-butoxydimethylsilyl)-1,4-dimethyl-1*H*-indole

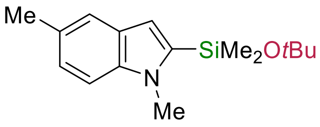

8a
$C_{16}H_{25}NOSi$
mw: 275.47 g/mol

Prepared according to **GP2** from the corresponding diazene **3a** (195 mg, 0.90 mmol, 1.8 eq.) and 1,5-dimethyl-1*H*-indole (72.6 mg, 0.50 mmol, 1.0 eq.) and potassium *tert*-butoxide (5.6 mg, 10 mol%). The reaction mixture was stirred for 2 h. Purification by flash column

chromatography on silica gel using PE/EtOAc (100:0 → 99:1 v/v) as eluent afforded the title compound **8a** (113.8 mg, 0.41 mmol, 83 %) as pinkish solid.

M.p (CH_2Cl_2) = 63-65 °C. **^{1}H NMR** (400 MHz, $CDCl_3$): δ/ppm = 7.30 – 7.25 (m, 1H), 7.11 (d, *J* = 8.1 Hz, 1H), 6.94 (dd, *J* = 8.4, 1.7 Hz, 1H), 6.50 (s, 1H), 3.80 (s, 3H), 2.33 (s, 3H), 1.13 (s, 9H), 0.37 (s, 6H). **^{13}C{^{1}H} NMR** (101 MHz, $CDCl_3$): δ/ppm = 141.5, 138.7, 128.7, 128.3, 124.0, 120.5, 110.6, 109.0, 73.5, 33.1, 32.0, 21.5, 1.9. **^{1}H/^{29}Si HMQC NMR** (400/79 MHz, $CDCl_3$): δ/ppm = 0.37/-8.4. **HRMS (ESI)** m/z: $[M+H]^+$ Calcd for $C_{16}H_{25}NOSiH$ 276.1778. Found 276.1779.

2-(*tert*-butoxydimethylsilyl)-1-methyl-5-phenyl-1*H*-indole

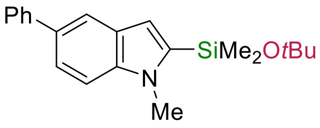

9a
$C_{21}H_{27}NOSi$
mw: 337.54 g/mol

Prepared according to **GP2** from the corresponding diazene **3a** (195 mg, 0.90 mmol, 1.8 eq.) and 1-methyl-5-phenyl-1*H*-indole (103.7 mg, 0.50 mmol, 1.0 eq.) and potassium *tert*-butoxide (5.6 mg, 10 mol%). The reaction mixture was stirred for 2 h. Purification by flash column chromatography on silica gel using PE/EtOAc (100:0 → 99:1 v/v) as eluent afforded the title compound **9a** (171.6 mg, 0.50 mmol, >99 %) as a yellow solid.

M.p (CH_2Cl_2) = 68-70 °C. **^{1}H NMR** (300 MHz, $CDCl_3$): δ/ppm = 7.86 (s, 1H), 7.74 – 7.63 (m, 2H), 7.58 – 7.39 (m, 4H), 7.38 – 7.27 (m, 1H), 6.78 (s, 1H), 4.00 (s, 3H), 1.30 (s, 9H), 0.54 (s, 6H). **^{13}C{^{1}H} NMR** (75 MHz, $CDCl_3$): δ/ppm = 142.8, 142.4, 139.8, 132.8, 129.0, 128.8, 127.5, 126.3, 122.2, 119.5, 111.6, 109.5, 73.6, 33.3, 32.0, 1.9. **^{1}H/^{29}Si HMQC NMR** (300/60 MHz, $CDCl_3$): δ/ppm = 0.54/-8.4. **HRMS (ESI)** m/z: [M+H]+ Calcd for $C_{21}H_{27}NOSiH$ 338.1935. Found 338.1933.

2-(*tert*-butoxydimethylsilyl)-5-chloro-1-methyl-1*H*-indole

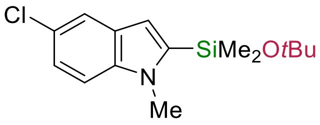

10a
$C_{15}H_{22}ClNOSi$
mw: 295.88 g/mol

Prepared according to **GP2** from the corresponding diazene **3a** (275 mg, 1.0 mmol, 2.0 eq.) and 5-chloro-1-methyl-1*H*-indole (82.7 mg, 0.50 mmol, 1.0 eq.) and potassium *tert*-butoxide (11.2 mg, 20 mol%, 0.2 eq.). The reaction mixture was stirred for 2 h. Purification by flash

column chromatography on silica gel using PE/EtOAc (100:0 → 95:5 v/v) as eluent afforded the title compound **10a** (97.8 mg, 0.33 mmol, 66 %) as a yellow oil.

^{1}H NMR (300 MHz, $CDCl_3$): δ/ppm = 7.57 (d, *J* = 2.0 Hz, 1H), 7.25 (d, *J* = 8.8 Hz, 1H), 7.17 (dd, *J* = 8.8, 2.0 Hz, 1H), 6.63 (s, 1H), 3.93 (s, 3H), 1.26 (s, 9H), 0.50 (s, 6H). **^{13}C{^{1}H} NMR** (75 MHz, $CDCl_3$): δ/ppm = 143.1, 138.6, 129.4, 125.0, 122.5, 120.2, 110.6, 110.2, 73.6, 33.3, 32.0, 1.9. **^{1}H/^{29}Si HMQC NMR** (300/60 MHz, $CDCl_3$): δ/ppm = 0.50/-8.4. **HRMS (ESI)** m/z: $[M+H]^+$ Calcd for $C_{15}H_{22}ClNOSiH$ 296.1232. Found 296.1233.

2-(*tert*-butoxydimethylsilyl)-1-methyl-1*H*-pyrrolo[2,3-b]pyridine

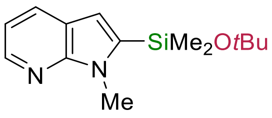

11a
$C_{14}H_{22}N_2OSi$
mw: 262.43 g/mol

Prepared according to **GP2** from the corresponding diazene **3a** (195 mg, 0.90 mmol, 1.8 eq.) and 1-methyl-1*H*-pyrrolo[2,3-*b*]pyridine (66.1 mg, 0.50 mmol, 1.0 eq.) and potassium *tert*-butoxide (5.6 mg, 10 mol%). The reaction mixture was stirred for 2 h. Purification by flash column chromatography on silica gel using PE/EtOAc (100:0 → 99:1 v/v) as eluent afforded the title compound **11a** (104.8 mg, 0.40 mmol, 80 %) as an orange oil.

^{1}H NMR (300 MHz, $CDCl_3$): δ/ppm = 8.35 (dd, J = 4.7, 1.6 Hz, 1H), 7.88 (dd, J = 7.8, 1.6 Hz, 1H), 7.01 (dd, J = 7.8, 4.7 Hz, 1H), 6.65 (s, 1H), 4.05 (s, 3H), 1.26 (s, 10H), 0.51 (s, 6H). **^{13}C{^{1}H} NMR** (75 MHz, $CDCl_3$): δ/ppm = 150.8, 143.6, 142.1, 128.8, 120.5, 115.4, 109.1, 73.7, 32.0, 31.5, 1.8. **^{1}H/^{29}Si HMQC NMR** (300/60 MHz, $CDCl_3$): δ/ppm = 0.51/-8.2. **HRMS (ESI)** m/z: $[M+H]^+$ Calcd for $C_{14}H_{22}N_2OSiH$ 263.1574. Found 263.1574.

benzo[b]thiophen-2-yl(*tert*-butoxy)dimethylsilane

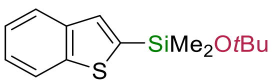

13a
$C_{14}H_{20}OSSi$
mw: 264.46 g/mol

Prepared according to **GP2** from the corresponding diazene **3a** (195 mg, 0.90 mmol, 1.8 eq.) and benzo[*b*]thiophene (67.1 mg, 0.50 mmol, 1.0 eq.) and potassium *tert*-butoxide (5.6 mg, 10 mol%). The reaction mixture was stirred for 2 h. Purification by flash column chromatography on silica gel using PE/EtOAc (100:0 → 99:1 v/v) as eluent afforded the title compound **13a** (119.5 mg, 0.45 mmol, 90 %) as a light-yellow oil.

^{1}H NMR (400 MHz, $CDCl_3$): δ/ppm = 7.94 – 7.88 (m, 1H), 7.86 – 7.79 (m, 1H), 7.54 (s, 1H), 7.40 – 7.30 (m, 2H), 1.30 (s, 9H), 0.49 (s, 6H). **^{13}C{^{1}H} NMR** (101 MHz, $CDCl_3$): δ/ppm = 143.8, 142.6, 141.1, 131.3, 124.4, 124.1, 123.8, 122.4, 73.6, 32.1, 2.4. **^{1}H/^{29}Si HMQC NMR** (400/79 MHz, $CDCl_3$): δ/ppm = 0.49/-6.5. **HRMS (ESI)** m/z: $[M+H]^+$ Calcd for $C_{14}H_{20}OSSiH$ 265.1077. Found 265.1078.

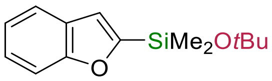

12a
$C_{14}H_{20}O_2Si$
mw: 248.40 g/mol

Prepared according to **GP2** from the corresponding diazene **3a** (195 mg, 0.90 mmol, 1.8 eq.) and benzo[*b*]thiophene (58.1 µL, 59 mg, 0.50 mmol, 1.0 eq.) and potassium *tert*-butoxide (5.6 mg, 10 mol%). The reaction mixture was stirred for 2 h. Purification by flash column chromatography on silica gel using PE/EtOAc (100:0 → 99:1 v/v) as eluent afforded the title compound **12a** (109.7 mg, 0.44 mmol, 88 %) as a light-yellow oil.

^{1}H NMR (400 MHz, $CDCl_3$): δ/ppm = 7.60 (ddd, *J* = 7.7, 1.4, 0.7 Hz, 1H), 7.54 (dd, *J* = 8.1, 1.1 Hz, 1H), 7.30 (ddd, *J* = 8.3, 7.2, 1.4 Hz, 1H), 7.21 (d, *J* = 1.1 Hz, 1H), 7.05 (s, 1H), 1.28 (s, 9H), 0.47 (s, 6H). **^{13}C{^{1}H} NMR** (101 MHz, $CDCl_3$): δ/ppm = 204.1, 199.1, 169.2, 165.8, 163.6, 162.5, 157.8, 152.7, 114.7, 73.1, 42.0. **^{1}H/^{29}Si HMQC NMR** (400/79 MHz, $CDCl_3$): δ/ppm = 0.47/-10.8. **HRMS (ESI)** m/z: $[M+H]^+$ Calcd for $C_{14}H_{20}O_2SiH$ 249.1305. Found 249.1306.

***tert*-butoxydimethyl(5-pentylfuran-2-yl)silane**

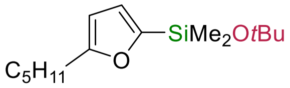

14a
$C_{15}H_{28}O_2Si$
mw: 268.47 g/mol

Prepared according to **GP2** from the corresponding diazene **3a** (195 mg, 0.90 mmol, 1.8 eq.), 2-pentylfuran (78 µL, 0.50 mmol, 1.0 eq.) and potassium *tert*-butoxide (5.6 mg, 10 mol%, 0.1 eq.). The reaction mixture was stirred for 1 h. Purification by flash column chromatography on silica gel using PE/NEt_3 (99:1 v/v) as eluent afforded the title compound **14a** (86 mg, 0.32 mmol, 64 %) as a colorless oil.

^{1}H NMR (300 MHz, $CDCl_3$): δ/ppm = 6.58 (d, *J* = 3.1 Hz, 1H), 5.96 (d, *J* = 3.1 Hz, 1H), 2.65 (t, *J* = 7.5 Hz, 2H), 1.72 – 1.59 (m, 2H), 1.39 – 1.28 (m, 4H), 1.21 (s, 9H), 0.95 – 0.84 (m, 3H), 0.34 (s, 6H). **^{13}C{^{1}H} NMR** (75 MHz, $CDCl_3$): δ/ppm = 161.0, 158.0, 121.2, 104.9, 73.0, 31.9, 31.5, 28.3, 28.0, 22.6, 14.2, 0.8. **^{1}H/^{29}Si HMQC NMR** (300/60 MHz, $CDCl_3$): δ/ppm = 0.34/-12.3. **HRMS (ESI)** m/z: $[M+H]^+$ Calcd for $C_{15}H_{28}O_2SiH$ 269.1931. Found 269.1933.

2,5-bis(di-*tert*-butoxy(methyl)silyl)thiophene

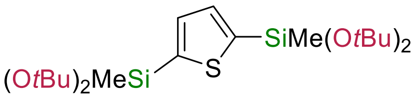

15d$_2$
$C_{22}H_{44}O_4SSi_2$
mw: 460.82 g/mol

Prepared according to **GP2** from the corresponding diazene **3d** (439 mg, 1.6 mmol, 3.2 eq.), thiophene (40 µL, 0.50 mmol, 1.0 eq.) and potassium *tert*-butoxide (5.6 mg, 10 mol%, 0.1 eq.). The reaction mixture was stirred for 16 h. Purification by flash column chromatography on silica gel using PE/EtOAc (100:0 → 99:1 v/v) as eluent afforded the title compound **15d$_2$** (223.5 mg, 0.49 mmol, 97 %) as a colorless oil.

Note: The non-volatile disiloxane side product [(*t*BuO)$_2$MeSi]$_2$O proved inseparable by column chromatography (ca. 13%).

^{1}H NMR (300 MHz, $CDCl_3$): δ/ppm = 7.38 (s, 2H), 1.30 (s, 36H), 0.42 (s, 6H). **^{13}C{^{1}H} NMR** (75 MHz, $CDCl_3$): δ/ppm = 145.5, 135.7, 73.5, 32.0, 2.8. **^{1}H/^{29}Si HMQC NMR** (300/60 MHz, $CDCl_3$): δ/ppm = 7.38, 0.42/-37.1. *Note*: HRMS could not be obtained for this compound as ionization using ESI or APCI techniques coupled with MeCN elution did not occur.

(2-fluoro-1,3-phenylene)bis(*tert*-butoxydimethylsilane)

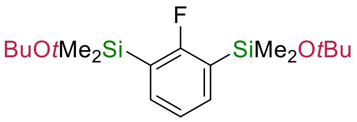

16a$_2$
$C_{18}H_{33}FO_2Si_2$
mw: 356.63 g/mol

Prepared according to **GP2** from the corresponding diazene **3a** (379 mg, 1.75 mmol, 3.5 eq.), fluorobenzene (48.1 mg, 0.50 mmol, 1.0 eq.) and potassium *tert*-butoxide (11.2 mg, 20 mol%). The reaction mixture was stirred for 2 h. Purification by flash column chromatography on silica gel using PE/NEt_3 (99:1 v/v) as eluent afforded the title compound **16a$_2$** (155.5 mg, 0.44 mmol, 87 %) as a colorless oil.

^{1}H NMR (300 MHz, $CDCl_3$): δ/ppm = 7.59 (dd, *J* = 7.2, 5.8 Hz, 2H), 7.15 (td, *J* = 7.2, 1.7 Hz, 1H), 1.27 (s, 18H), 0.43 (d, *J* = 1.3 Hz, 12H). **^{13}C{^{1}H} NMR** (75 MHz, $CDCl_3$): δ/ppm = 171.3 (d, *J* = 237.6 Hz), 137.5 (d, *J* = 11.4 Hz), 125.5 (d, *J* = 34.2 Hz), 123.5 (d, *J* = 2.8 Hz), 73.1, 32.1, 2.2 (d, *J* = 1.9 Hz).**^{19}F{^{1}H} NMR** (282 MHz, $CDCl_3$): δ/ppm = -87.38. **^{1}H/^{29}Si HMQC NMR** (300/60 MHz, $CDCl_3$): δ/ppm = 0.4, 7.59, 0.4/-3.9. **HRMS (ESI)** m/z: $[M+Na]^+$ Calcd for $C_{18}H_{33}FO_2Si_2Na$ 379.1895. Found 379.1895.

Tri-*tert*-butoxy(2-fluorophenyl)silane

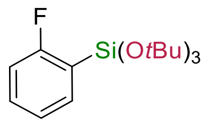

16e
$C_{18}H_{31}FO_3Si$
mw: 342.53 g/mol

Prepared according to **GP2** from the corresponding diazene **3e** (291 mg, 0.85 mmol, 1.7 eq.), fluorobenzene (48.1 mg, 0.50 mmol, 1.0 eq.) and potassium *tert*-butoxide (16.8 mg, 30 mol%). The reaction mixture was stirred for 21 h. Purification by flash column chromatography on silica gel using PE/NEt_3 (99:1 v/v) as eluent afforded the title compound **16e** (125.8 mg, 0.39 mmol, 73 %) as a colorless oil.

^{1}H NMR (300 MHz, $CDCl_3$): δ/ppm = 7.69 (ddd, *J* = 7.5, 5.9, 1.9 Hz, 1H), 7.39 – 7.29 (m, 1H), 7.09 (tt, *J* = 7.3, 0.9 Hz, 1H), 6.94 (ddd, *J* = 9.1, 8.2, 1.0 Hz, 1H), 1.35 (s, 27H). **^{13}C{^{1}H} NMR** (75 MHz, $CDCl_3$): δ/ppm = 166.8 (d, *J* = 244.0 Hz), 137.7 (d, *J* = 10.1 Hz), 131.5 (d, *J* = 8.4 Hz), 124.8 (d, *J* = 27.3 Hz), 123.5 (d, *J* = 3.0 Hz), 114.9 (d, *J* = 25.6 Hz), 73.8, 31.9. **^{19}F{^{1}H NMR** (282 MHz, $CDCl_3$): δ/ppm = -97.46. **^{1}H/^{29}Si HMQC NMR** (300/60 MHz, $CDCl_3$): δ/ppm = 7,69/-180,6. **HRMS (ESI)** m/z: $[M+Na]^+$ Calcd for $C_{18}H_{31}FO_3SiNa$ 365.1919. Found 365.1921.

(2,4,6-trifluorobenzene-1,3,5-triyl)tris(*tert*-butoxydimethylsilane)

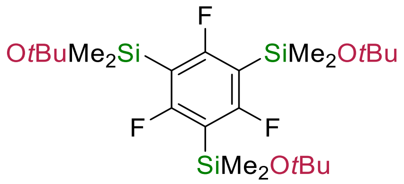

17a$_3$
$C_{24}H_{45}F_3O_3Si_3$
mw: 522.87 g/mol

Prepared according to **GP2** from the corresponding diazene **3a** (433 mg, 2 mmol, 4.0 eq.), 1,3,5-trifluorobenzene (51.7 µL, 0.50 mmol, 1.0 eq.) and potassium *tert*-butoxide (11.2 mg, 20 mol%). The reaction mixture was stirred for 2 h. Purification by flash column chromatography on silica gel using PE/NEt_3 (99:1 v/v) then PE/AcOEt (98:2 → 95:5 v/v) as eluents afforded the title compound **17a$_2$** (240.1 mg, 0.46 mmol, 92 %) as a light-yellow oil.

^{1}H NMR (300 MHz, $CDCl_3$): δ/ppm = 1.22 (s, 27H), 0.44 (s, 18H). **^{13}C{^{1}H} NMR** (75 MHz, $CDCl_3$): δ/ppm = 173.0 (dt, *J* = 244.9, 20.0 Hz), 109.3 (t), 73.3, 31.8, 3.7. **^{19}F{^{1}H} NMR** (282 MHz, $CDCl_3$) δ -78.44. **^{1}H/^{29}Si HMQC NMR** (300/60 MHz, $CDCl_3$): δ/ppm = 0.44/-5.7. **HRMS (ESI)** m/z: $[M+Na]^+$ Calcd for $C_{24}H_{45}F_3O_3Si_3Na$ 545.2521. Found 545.2519.

(2-fluoro-4-methoxy-1,3-phenylene)bis(*tert*-butoxydimethylsilane)

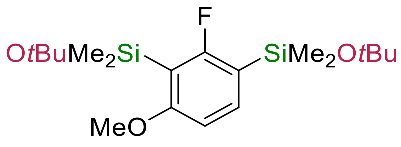

18a$_2$
$C_{19}H_{35}FO_3Si_2$
mw: 386.65 g/mol

Prepared according to **GP2** from the corresponding diazene **3a** (433 mg, 2 mmol, 4 eq.), 3-fluoroanisole (63.1 mg, 0.50 mmol, 1.0 eq.) and potassium *tert*-butoxide (11.2 mg, 20 mol%). The reaction mixture was stirred for 1 h. Purification by flash column chromatography on silica gel using PE/Et_2O (100:0 → 99:1 → 98:2 v/v) as eluent afforded the title compound **18a$_2$** (154.4 mg, 0.40 mmol, 80 %) as a light-yellow oil.

^{1}H NMR (300 MHz, $CDCl_3$): δ/ppm = 7.52 (dd, *J* = 8.1, 6.8 Hz, 1H), 6.64 (d, *J* = 8.1 Hz, 1H), 3.80 (s, 3H), 1.26 (s, 9H), 1.21 (s, 9H), 0.43 (d, *J* = 2.4 Hz, 6H), 0.39 (d, *J* = 1.3 Hz, 6H). **^{13}C{^{1}H} NMR** (75 MHz, $CDCl_3$): δ/ppm = 171.9 (d, *J* = 238.7 Hz), 167.0 (d, *J* = 15.9 Hz), 138.5 (d, *J* = 14.7 Hz), 117.8 (d, *J* = 36.4 Hz), 113.4 (d, *J* = 35.5 Hz), 105.9 (d, *J* = 2.7 Hz), 72.9, 72.9, 55.4, 32.1, 31.8, 4.2 (d, *J* = 4.1 Hz), 2.3 (d, *J* = 1.9 Hz). **^{19}F{^{1}H} NMR** (282 MHz, $CDCl_3$): δ/ppm = -84.24. **^{1}H/^{29}Si HMQC NMR** (300/60 MHz, $CDCl_3$): δ/ppm = 0.4/-4.9, 0.4/-4.1. **HRMS (ESI)** m/z: $[M+Na]^+$ Calcd for $C_{19}H_{35}FO_3Si_2Na$ 409.2001. Found 409.2000.

***tert*-butoxy(2-fluoro-3-methoxyphenyl)dimethylsilane**

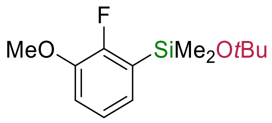

19a
$C_{13}H_{21}FO_2Si$
mwt: 256.39 g/mol

Prepared according to **GP2** from the corresponding diazene **3a** (162 mg, 0.75 mmol, 1.5 eq.), 1-fluoro-2-methoxybenzene (63.1 mg, 0.50 mmol, 1.0 eq.) and potassium *tert*-butoxide (5.6 mg, 10 mol%). The reaction mixture was stirred for 1 h. Purification by flash column chromatography on silica gel using PE/EtOAc (100:0 → 99:1 v/v) as eluent afforded the title compound **19a** (131 mg, 0.50 mmol, >99 %) as a colorless oil.

^{1}H NMR (300 MHz, $CDCl_3$): δ/ppm = 7.15 – 7.04 (m, 2H), 6.98 (ddd, *J* = 9.4, 7.2, 2.6 Hz, 1H), 3.88 (s, 3H), 1.29 (s, 9H), 0.44 (s, 6H). **^{13}C{^{1}H} NMR** (75 MHz, $CDCl_3$): δ/ppm = 156.1 (d, *J* = 240.4 Hz), 147.0 (d, *J* = 14.0 Hz), 127.2 (d, *J* = 27.0 Hz), 126.3 (d, *J* = 10.5 Hz), 124.0 (d, *J* = 3.6 Hz), 114.6 (d, *J* = 2.2 Hz), 73.1, 56.1, 31.9, 2.1 (d, *J* = 1.7 Hz). **^{19}F{^{1}H} NMR** (282 MHz, $CDCl_3$): δ/ppm = -124.11. **^{1}H/^{29}Si HMQC NMR** (300/60 MHz, $CDCl_3$): δ/ppm = 0.44/-4.1. **HRMS (ESI)** m/z: $[M+H]^+$ Calcd for $C_{13}H_{21}FO_2SiH$ 257.1368. Found 257.1368.

(2-fluoro-5-phenoxy-1,3-phenylene)bis(*tert*-butoxydimethylsilane)

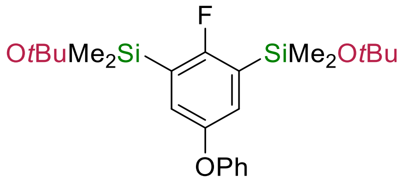

20a$_2$
$C_{24}H_{37}FO_3Si_2$
mw: 448,73 g/mol

Prepared according to **GP2** from the corresponding diazene **3a** (357 mg, 1.65 mmol, 3.3 eq.), 1-fluoro-4-phenoxybenzene (94.1 mg, 0.50 mmol, 1.0 eq.) and potassium *tert*-butoxide (11.2 mg, 20 mol%). The reaction mixture was stirred for 1 h. Purification by flash column chromatography on silica gel using PE/NEt_3 (99:1 v/v) as eluent afforded the title compound **20a$_2$** (180.1 mg, 0.40 mmol, 80 %) as a light-yellow oil.

^{1}H NMR (300 MHz, $CDCl_3$): δ/ppm = 7.37 – 7.28 (m, 2H), 7.19 (d, *J* = 4.3 Hz, 2H), 7.11 – 7.03 (m, 1H), 7.01 – 6.95 (m, 2H), 1.23 (s, 18H), 0.40 (d, *J* = 1.2 Hz, 12H). **^{13}C{^{1}H} NMR** (101 MHz, $CDCl_3$): δ/ppm = 166.8 (d, *J* = 233.5 Hz), 158.2, 152.4 (d, *J* = 2.3 Hz), 129.8, 127.7 (d, *J* = 12.4 Hz), 127.5 (d, *J* = 37.1 Hz), 122.8, 118.2, 73.2, 32.0, 2.1 (d, *J* = 1.8 Hz). **^{19}F{^{1}H} NMR** (282 MHz, $CDCl_3$): δ/ppm = -95.73. **^{1}H/^{29}Si HMQC NMR** (300/60 MHz, $CDCl_3$): δ/ppm = 0.40/-4.3. **HRMS (ESI)** m/z: $[M+Na]^+$ Calcd for $C_{24}H_{37}FO_3Si_2Na$ 471.2157. Found 471.2155.

***tert*-butoxy(2-fluoro-[1,1'-biphenyl]-3-yl)dimethylsilane**

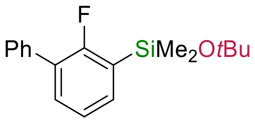

21a
$C_{18}H_{23}FOSi$
mw: 302.46 g/mol

Prepared according to **GP2** from the corresponding diazene **3a** (162 mg, 0.75 mmol, 1.5 eq.), 2-fluoro-1,1'-biphenyl (86.1 mg, 0.50 mmol, 1.0 eq.) and potassium *tert*-butoxide (5.6 mg, 10 mol%, 0.1 eq.). The reaction mixture was stirred for 1 h. Purification by flash column chromatography on silica gel using PE/EtOAc (100:0 → 99:1 v/v) as eluent afforded the title compound **21a** (143 mg, 0.47 mmol, 95 %) as a white solid.

Note: The same reaction carried out on a larger scale (2 mmol) gave a similar yield (**21a**: 564 mg, 1.86 mmol, 93 %).

M.p (CH_2Cl_2) = 41-43 °C. **^{1}H NMR** (400 MHz, $CDCl_3$): δ/ppm = 7.60 – 7.53 (m, 3H), 7.50 – 7.42 (m, 3H), 7.41 – 7.34 (m, 1H), 7.26 – 7.20 (m, 1H), 1.33 (s, 9H), 0.48 (s, 6H). **^{13}C{^{1}H} NMR** (101

MHz, $CDCl_3$): δ/ppm = 163.7 (d, *J* = 243.0 Hz), 136.5, 135.1 (d, *J* = 11.6 Hz), 132.5 (d, *J* = 3.7 Hz), 129.3 (d, *J* = 2.7 Hz), 128.5, 128.5 (d, *J* = 17.8 Hz), 127.6, 127.2 (d, *J* = 31.8 Hz), 124.2 (d, *J* = 3.3 Hz), 73.2, 32.2, 2.4 (d, *J* = 1.9 Hz). **$^{19}F\{^1H\}$ NMR** (376 MHz, $CDCl_3$): δ/ppm = -105.98. **$^1H/^{29}Si$ HMQC NMR** (400/79 MHz, $CDCl_3$): δ/ppm = 0.48/-3.9. **HRMS (ESI)** m/z: $[M+Na]^+$ Calcd for $C_{18}H_{23}FOSiNa$ 325.1394. Found 325.1390.

***tert*-butoxy(2-fluoro-3-methylphenyl)dimethylsilane**

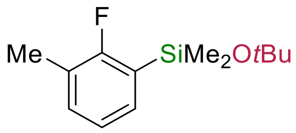

22a
$C_{13}H_{21}FOSi$
mw: 240.39 g/mol

Prepared according to **GP2** from the corresponding diazene **3a** (195 mg, 0.9 mmol, 1.8 eq.), 2-fluorotoluene (55 µL, 0.50 mmol, 1.0 eq.) and potassium *tert*-butoxide (5.6 mg, 10 mol%). The reaction mixture was stirred for 1 h. The yield of **22a** was determined by $^{19}F\{^1H\}$ NMR spectroscopy (see Table S7). Purification by flash column chromatography on silica gel using PE as eluent allowed to isolate pure fractions containing the title compound (colorless oil) for analytical purpose.

^1H NMR (300 MHz, $CDCl_3$): δ/ppm = 7.39 (t, *J* = 5.3 Hz, 1H), 7.20 (t, *J* = 7.3 Hz, 1H), 7.04 (t, *J* = 7.3 Hz, 1H), 2.27 (s, 3H), 1.29 (s, 9H), 0.44 (s, 6H). **$^{13}C\{^1H\}$ NMR** (75 MHz, $CDCl_3$): δ/ppm = 165.5 (d, *J* = 240.4 Hz), 133.2 (d, *J* = 15.2 Hz), 133.2 (d, *J* = 1.3 Hz), 125.9 (d, *J* = 30.7 Hz), 124.1 (d, *J* = 21.0 Hz), 123.6 (d, *J* = 3.4 Hz), 73.1, 32.1, 14.8 (d, *J* = 3.9 Hz), 2.3 (d, *J* = 1.8 Hz). **$^{19}F\{^1H\}$ NMR** (282 MHz, $CDCl_3$): δ/ppm = -105.42. **$^1H/^{29}Si$ HMQC NMR** (300/60 MHz, $CDCl_3$): δ/ppm = 7.39, 0.44/-3.9.

6.3. Characterization data for silylated toluene derivatives

benzyl(*tert*-butoxy)dimethylsilane

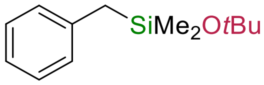

23a
$C_{13}H_{22}OSi$
mw: 222.40 g/mol

Prepared according to **GP3** from the corresponding diazene **3a** (325 mg, 1.50 mmol, 3.0 eq.), toluene (53.2 µL, 0.50 mmol, 1.0 eq.) and potassium *tert*-butoxide (5.6 mg, 10 mol%). The reaction mixture was stirred for 4 h at room temperature. Purification by column

chromatography on silica gel using PE as eluent afforded the title compound **23a** (57.2 mg, 0.25 mmol, 51 %) as a colorless oil.

^{1}H NMR (300 MHz, $CDCl_3$): δ/ppm = 7.20 (dd, *J* = 8.1, 7.0 Hz, 1H), 7.12 – 7.01 (m, 3H), 2.14 (s, 2H), 1.24 (s, 9H), 0.09 (s, 6H). **^{13}C{^{1}H} NMR** (75 MHz, $CDCl_3$): δ/ppm = 140.1, 128.6, 128.1, 124.1, 72.5, 32.2, 29.0, 0.7. **^{1}H/^{29}Si HMQC NMR** (300/60, $CDCl_3$): δ/ppm = 0.09/4.7.

benzyldi-*tert*-butoxy(methyl)silane

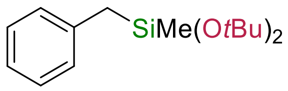

23d
$C_{16}H_{28}O_2Si$
mw: 280.48 g/mol

Prepared according to **GP3** from the corresponding diazene **3d** (412 mg, 1.50 mmol, 3.0 eq.), toluene (53.2 µL, 0.50 mmol, 1.0 eq.) and potassium *tert*-butoxide (11.2 mg, 20 mol%). The reaction mixture was stirred overnight at room temperature. Purification by column chromatography on silica gel using PE/NEt_3 (99:1 v/v) afforded the title compound **23d** as a colorless oil.

Note: because of the presence of the siloxane and its coelution with the title compound, a pure fraction was isolated for the full characterization.

^{1}H NMR (300 MHz, $CDCl_3$): δ/ppm = 7.24 – 7.16 (m, 2H), 7.16 – 7.10 (m, 2H), 7.10 – 7.02 (m, 1H), 2.12 (s, 2H), 1.27 (s, 18H), 0.07 (s, 3H). **^{13}C{^{1}H} NMR** (75 MHz, $CDCl_3$): δ/ppm = 139.8, 128.9, 127.8, 123.9, 72.5, 31.9, 28.8, 0.5. **^{1}H/^{29}Si HMQC NMR** (300/60 MHz, $CDCl_3$): δ/ppm = 2.12, 0.07/- 24.6, 0.07/- 24.6. **HRMS (ESI)** m/z: $[M+Na]^+$ Calcd for $C_{16}H_{28}O_2SiNa$ 303.1751. Found 303.1751.

***tert*-butoxy(3-methoxybenzyl)dimethylsilane**

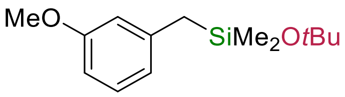

24a
$C_{14}H_{24}O_2Si$
mw: 252.43 g/mol

Prepared according to **GP3** from the corresponding diazene **3a** (325 mg, 1.50 mmol, 3.0 eq.), 3-methylanisole (61.1 mg, 0.50 mmol, 1.0 eq.) and potassium *tert*-butoxide (5.6 mg, 10 mol%). The reaction mixture was stirred overnight at room temperature. Purification by flash column chromatography on silica gel using PE/NEt_3 (99:1 v/v) as eluent afforded the title compound **24a** (87.1 mg, 0.35 mmol, 69 %) as a yellowish oil.

^{1}H NMR (300 MHz, $CDCl_3$): δ/ppm = 7.17 – 7.09 (m, 1H), 6.69 – 6.60 (m, 3H), 3.79 (s, 3H), 2.13 (s, 2H), 1.25 (s, 9H), 0.10 (s, 6H). **^{13}C{^{1}H} NMR** (75 MHz, $CDCl_3$): δ/ppm = 159.6, 141.7, 129.0, 121.3, 114.3, 109.6, 72.5, 55.2, 32.2, 29.1, 0.8. **^{1}H/^{29}Si HMQC NMR** (300/60, $CDCl_3$): δ/ppm = 2.13, 0.10/4.9. **HRMS (ESI)** m/z: $[M+Na]^+$ Calcd for $C_{14}H_{24}O_2SiNa$ 275.1438. Found 275.1439.

***tert*-butoxydimethyl(3-phenoxybenzyl)silane**

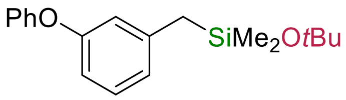

26a

$C_{19}H_{26}O_2Si$

mw: 314.50 g/mol

Prepared according to **GP3** from the corresponding diazene **3a** (325 mg, 1.50 mmol, 3.0 eq.), 3-phenoxytoluene (92.1 mg, 0.50 mmol, 1.0 eq.) and potassium *tert*-butoxide (5.6 mg, 10 mol%). The reaction mixture was stirred overnight at room temperature. Purification by flash column chromatography on silica gel using petroleum ether then PE/CH_2Cl_2 (95:5 → 90:10 v/v) as eluent afforded the title compound **26a** (110.4 mg, 0.35 mmol, 70 %) as a colorless oil.

^{1}H NMR (300 MHz, $CDCl_3$): δ/ppm = 7.32 (dd, J = 8.5, 7.3 Hz, 2H), 7.17 (dd, J = 8.8, 7.6 Hz, 1H), 7.07 (t, J = 7.4 Hz, 1H), 6.99 (d, J = 8.2 Hz, 2H), 6.82 (d, J = 7.6 Hz, 2H), 6.76 – 6.71 (m, 2H), 2.12 (s, 2H), 1.22 (s, 9H), 0.09 (s, 6H). **^{13}C{^{1}H} NMR** (75 MHz, $CDCl_3$): δ/ppm = 157.8, 157.1, 142.3, 129.8, 129.3, 123.8, 123.0, 119.3, 118.8, 114.9, 72.6, 32.2, 29.0, 0.8. **^{1}H/^{29}Si HMQC NMR** (300/60, $CDCl_3$): δ/ppm = 2.12, 0.09/4.7. **HRMS (APCI)** m/z: $[M-H]^-$ Calcd for $C_{19}H_{25}O_2Si$ 313.1629. Found 313.1629.

(4-((*tert*-butoxydimethylsilyl)methyl)phenyl)diphenylphosphane

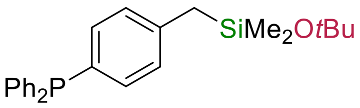

28a

$C_{25}H_{31}OPSi$

mw: 406.58 g/mol

Prepared according to **GP3** from the corresponding diazene **3a** (325 mg, 1.50 mmol, 3.0 eq.), diphenyl(*p*-tolyl)phosphane (138.2 mg, 0.50 mmol, 1.0 eq.) and potassium *tert*-butoxide (11.2 mg, 20 mol%). The reaction mixture was stirred overnight at room temperature. The crude was then quenched under argon with 6.5 mL of a degassed 0.5 M HCl solution. The aqueous layer was extracted with Et_2O (3 × 10 mL), the combined organic phases were dried over $MgSO_4$, filtrated and evaporated. Purification by column chromatography on silica gel using PE/EtOAc

(100:0 → 98:2 v/v) as eluent afforded the title compound **28a** (166.2 mg, 0.41 mmol, 82 %) as a white solid.

M.p (CH_2Cl_2) = 48-50 °C. **^{1}H NMR** (400 MHz, $CDCl_3$): δ/ppm = 7.36 – 7.28 (m, 10H), 7.20 – 7.15 (m, 2H), 7.08 – 7.03 (m, 2H), 2.15 (s, 2H), 1.22 (s, 9H), 0.10 (s, 6H). **^{13}C{^{1}H} NMR** (101 MHz, $CDCl_3$): δ/ppm = 141.3, 138.0 (d, *J* = 10.8 Hz), 133.9 (d, *J* = 20.0 Hz), 133.7 (d, *J* = 19.2 Hz), 131.6 (d, *J* = 8.5 Hz), 128.9 (d, *J* = 7.5 Hz), 128.6, 128.5 (d, *J* = 6.9 Hz), 72.6, 32.2, 29.1, 0.9. **^{31}P{^{1}H} NMR** (162 MHz, $CDCl_3$): δ/ppm = -6.34. **^{1}H/^{29}Si HMQC NMR** (400/79 MHz, $CDCl_3$): δ/ppm = 0.10/4.5. **HRMS (ESI)** m/z: $[M+H]^+$ Calcd for $C_{25}H_{31}POSiH$ 407.1955. Found 407.1960.

([1,1'-biphenyl]-4-ylmethyl)(*tert*-butoxy)dimethylsilane

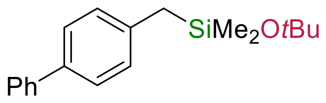

29a
$C_{19}H_{26}OSi$
mw: 298.50 g/mol

Prepared according to **GP3** from the corresponding diazene **3a** (325 mg, 1.50 mmol, 3.0 eq.), 4-methyl-1,1'-biphenyl (84.1 mg, 0.50 mmol, 1.0 eq.) and potassium *tert*-butoxide (10 mol%, 5.6 mg, 0.1 eq.). The reaction mixture was stirred overnight at room temperature. Purification by column chromatography on silica gel using PE/EtOAc (100:0 → 95:5 v/v) as eluent afforded the title compound **29a** (125.8 mg, 0.42 mmol, 84 %) as a light yellow solid.

M.p (CH_2Cl_2) = 41-43 °C. **^{1}H NMR** (400 MHz, $CDCl_3$): δ/ppm = 7.62 – 7.55 (m, 2H), 7.47 (d, *J* = 8.2 Hz, 2H), 7.42 (t, *J* = 7.6 Hz, 2H), 7.34 – 7.28 (m, 1H), 7.14 (d, *J* = 8.2 Hz, 2H), 2.19 (s, 2H), 1.26 (s, 9H), 0.13 (s, 6H). **^{13}C{^{1}H} NMR** (101 MHz, CDCl3): δ/ppm = 141.4, 139.3, 136.9, 129.0, 128.8, 126.9, 126.9, 126.8, 72.6, 32.2, 28.7, 0.8. **^{1}H/^{29}Si HMQC NMR** (400/79 MHz, $CDCl_3$): δ/ppm = 0.13/4.7. **HRMS (ESI)** m/z: $[M+Na]^+$ Calcd for $C_{19}H_{26}OSiNa$ 321.1645. Found 321.1646

***tert*-butoxydimethyl(naphthalen-2-ylmethyl)silane**

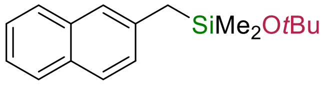

30a
$C_{17}H_{24}OSi$
mw: 272.46 g/mol

Prepared according to **GP3** from the corresponding diazene **3a** (325 mg, 1.50 mmol, 3.0 eq.), 2-methylnaphthalene (71.1 mg, 0.50 mmol, 1.0 eq.) and potassium *tert*-butoxide (11.2 mg, 20 mol%). The reaction mixture was stirred overnight at room temperature. Purification by column

chromatography on silica gel using PE/EtOAc (100:0 → 98:2 v/v) as eluent afforded the title compound **30a** (107.4 mg, 0.40 mmol, 79%) as a pale-yellow solid.

M.p (CH_2Cl_2) = 46-48 °C. **^{1}H NMR** (400 MHz, $CDCl_3$): δ/ppm = 7.77 (d, *J* = 7.5 Hz, 1H), 7.72 (dd, 2H), 7.50 (s, 1H), 7.42 (ddt, *J* = 8.1, 6.8, 1.3 Hz, 1H), 7.37 (ddd, *J* = 8.1, 6.8, 1.3 Hz, 1H), 7.25 (d, *J* = 8.3 Hz, 1H), 2.32 (s, 2H), 1.26 (s, 9H), 0.12 (d, *J* = 1.4 Hz, 6H). **^{13}C{^{1}H} NMR** (101 MHz, $CDCl_3$): δ/ppm = 137.8, 134.0, 131.2, 128.4, 127.7, 127.5, 127.1, 125.8, 125.8, 124.4, 72.6, 32.2, 29.3, 0.9. **^{1}H/^{29}Si HMQC NMR** (400/79 MHz, $CDCl_3$): δ/ppm = 0.12/4.9. **HRMS (ESI)** m/z: $[M+H]^+$ Calcd for $C_{17}H_{24}OSiH$ 273.1669. Found 273.1671.

4-((*tert*-butoxydimethylsilyl)methyl)pyridine

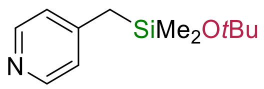

31a
$C_{12}H_{21}NOSi$
mw: 223.39 g/mol

Prepared according to **GP3** from the corresponding diazene **3a** (325 mg, 1.50 mmol, 3.0 eq.), 4-picoline (46.6 mg, 0.50 mmol, 1.0 eq.) and potassium *tert*-butoxide (5.6 mg, 10 mol%). The reaction mixture was stirred overnight at room temperature. Purification by column chromatography on silica gel using PE/EtOAc (90:10 → 80:20 v/v) as eluent afforded the title compound **31a** (68.3 mg, 0.31 mmol, 61%) as a light-yellow oil.

^{1}H NMR (300 MHz, $CDCl_3$): δ/ppm = 8.37 (d, *J* = 6.1 Hz, 1H), 6.97 (d, *J* = 5.8 Hz, 1H), 2.12 (s, 1H), 1.21 (s, 6H), 0.10 (s, 4H). **^{13}C{^{1}H} NMR** (75 MHz, $CDCl_3$): δ/ppm = 149.9, 149.3, 124.1, 72.8, 32.1, 29.2, 0.8. **^{1}H/^{29}Si HMQC NMR** (300/60 MHz, $CDCl_3$): δ/ppm = 2.12, 0.10/3.9. **HRMS (ESI)** m/z: $[M+H]^+$ Calcd for $C_{12}H_{21}NOSiH$ 224.1465. Found 224.1464.

***tert*-butoxydimethyl((methylthio)(phenyl)methyl)silane**

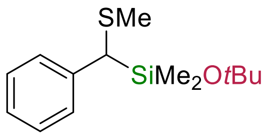

32a
$C_{14}H_{24}OSSi$
mw: 268.49 g/mol

Prepared according to **GP3** from the corresponding diazene **3a** (325 mg, 1.50 mmol, 3.0 eq.), benzyl(methyl)sulfane (69.1 mg, 0.50 mmol, 1.0 eq.) and potassium *tert*-butoxide (11.2 mg, 20 mol%). The reaction mixture was stirred for 4 h at room temperature. Purification by flash column chromatography on silica gel using PE/EtOAc (100:0 → 99:1 v/v) as eluent afforded the title compound **32a** (113.1 mg, 0.42 mmol, 84 %) as a light-yellow oil.

^{1}H NMR (300 MHz, $CDCl_3$): δ/ppm = 7.34 – 7.22 (m, 4H), 7.18 – 7.10 (m, 1H), 3.14 (s, 1H), 1.21 (s, 9H), 0.15 (d, *J* = 5.4 Hz, 6H). **^{13}C{^{1}H} NMR** (75 MHz, $CDCl_3$): δ/ppm = 141.0, 128.7, 128.1, 125.5, 73.0, 43.3, 32.0, 16.2, 0.2 (d, J = 5.4 Hz). **^{1}H/^{29}Si HMQC NMR** (300/60 MHz, $CDCl_3$): δ/ppm = 3.14, 0.18/2.1. *Note:* HRMS could not be obtained for this compound as ionization using APCI technique led to unidentified ions.

6.4. Applications (Scheme 4A and 4C in the manuscript)

Tert-butoxy(dodec-1-yn-1-yl)dimethylsilane

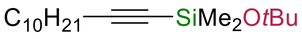

33a
$C_{18}H_{36}OSi$
mw: 296.57 g/mol

Prepared according to **GP2** from the corresponding diazene **3a** (162.1 mg, 0.6 mmol, 1.5 eq.), potassium hydroxide (2.8 mg, 0.05 mmol, 10 mol%) and 1-dodecyne (83.1 mg, 0.50 mmol, 1.0 eq.). The reaction mixture was stirred for 3 h at room temperature. Purification by column chromatography on silica gel using petroleum ether as eluent afforded the title compound **33a** (99.0 mg, 0.34 mmol, 67 %) as a colorless liquid.

R_f (petroleum ether) = 0.38. **^{1}H NMR** (300 MHz, $CDCl_3$) δ/ppm = 2.22 (t, *J* = 7.0 Hz, 2H), 1.51 (q, *J* = 7.1 Hz, 2H), 1.32-1.27 (m, 23H), 0.93 – 0.83 (m, 3H), 0.22 (s, 6H). **^{13}C{^{1}H} NMR** (101 MHz, $CDCl_3$) δ/ppm = 107.2, 85.1, 73.3, 32.1, 31.8 (3C), 29.7, 29.7, 29.5, 29.2, 29.0, 28.6, 22.8, 19.9, 14.2, 3.1 (2C). **^{1}H/^{29}Si HMQC NMR** (300/60 MHz, $CDCl_3$): δ/ppm = 0.20/-20.30. **HRMS (APCI)** m/z: $[M+H]^+$ Calcd for $C_{18}H_{36}OSiH$ 297.2608. Found 297.2610.

Tert-butoxy(3-decyl-1-methyl-1H-inden-2-yl)dimethylsilane

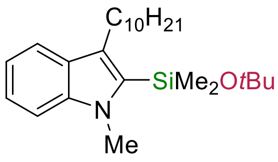

34
$C_{25}H_{43}NOSi$
Mw: 401.71 g/mol

Following a reported procedure,[11] a flame-dried, 25-mL microwave vial equipped with a magnetic stirring bar was charged under argon with potassium carbonate (138.1 mg, 1.0 mmol, 5.0 eq.), lithium chloride (8.4 mg, 0.2 mmol, 1.0 equiv), palladium acetate (2.3 mg, 0.01 mmol, 5 mol%), triphenylphosphine (2.6 mg, 0.01 mmol, 5 mol%) and DMF (1.0 mL). Another oven-dried 8-mL dram vial was charged with 2-iodo-N-methylaniline (46.4 mg, 0.2 mmol, 1.0 eq.), the silylated alkyne **33a** (118.4 mg, 0.4 mmol, 2.0 equiv) and DMF (0.5 mL). The resulting

solution was transferred to the palladium-containing 25-mL microwave vial, and the vial was further rinsed with additional DMF (0.5 mL). The reaction flask was then immerged in a pre-heated oil bath (100 °C) and stirred (900 rpm) for 5 h. After cooling to room temperature, the crude reaction mixture was passed through a plug of silica gel (0.5 g) eluted with ethyl acetate (20 mL). To the resulting solution, collected in a 100-mL round-bottomed, was added a solution of saturated NH_4Cl (5 mL). The phases were separated, the organic layer was then washed with H_2O (2 × 10 mL), and the combined organic layer was dried over $MgSO_4$, filtered and concentrated by rotary evaporation. Purification by column chromatography on silica gel using PE/AcOEt (50:1 v/v) as eluent afforded the title compound **34** (49.6 mg, 0.12 mmol, 62 %) as a brownish liquid.

R_f (PE/AcOEt = 50:1) = 0.53. **^{1}H NMR (300 MHz, $CDCl_3$)** δ/ppm = 7.61 (d, *J* = 7.9 Hz, 1H), 7.31 (d, *J* = 8.2 Hz, 1H), 7.25 (t, 1H), 7.08 (t, 1H), 3.94 (s, 3H), 2.92 – 2.81 (m, 2H), 1.71 – 1.57 (m, 2H), 1.49 – 1.21 (m, 23H), 0.91 (t, *J* = 6.6 Hz, 3H), 0.55 (s, 6H). **^{13}C{^{1}H} NMR** (101 MHz, $CDCl_3$) δ/ppm = 139.9, 135.4, 128.6, 125.7, 122.3, 119.4, 118.4, 109.1, 73.6, 33.2, 33.1, 32.1, 30.3, 29.8, 29.8, 29.8, 29.5, 25.9, 22.8, 14.3, 4.1. **^{1}H/^{29}Si HMQC NMR** (300/60 MHz, $CDCl_3$): δ/ppm = 0.61/-6.82. **HRMS (ESI)** m/z: $[M+H]^+$ Calcd for $C_{25}H_{43}NOSiH$ 402.3187. Found 402.3186.

Alkyl group modification :

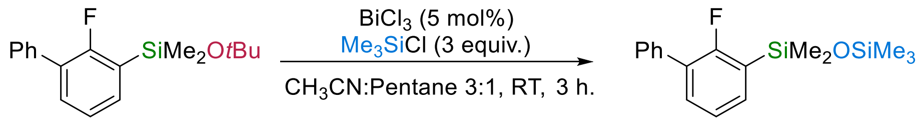

21a **35**: 63%

Adapting a reported procedure,[12] a flame-dried Schlenk equipped with a magnetic stirring bar was charged with **21a** (105.9 mg, 0.35 mmol, 1 eq.) and degassed pentane (0.4 mL). To the resulting solution was then added at room temperature a solution of bismuth trichloride (5.5 mg, 17.5 µmol, 5 mol%) in dry acetonitrile (1.2 mL) and chlorotrimethylsilane (133.3 µL, 1.05 mmol, 3eq.). The resulting mixture was then stirred at room temperature for 3 h. The volatiles were removed *in vacuo* by rotary evaporation. Purification of the crude residue by column chromatography on silica gel using PE then PE:CH_2Cl_2 (98:2 → 95:5 v/v) as eluent afforded compound **35** (70.4 mg, 0.22 mmol, 63 %) as a white solid.

M.p (CH_2Cl_2) = 56-58 °C. **^{1}H NMR** (300 MHz, $CDCl_3$): δ/ppm = 7.57 (d, *J* = 1.5 Hz, 2H), 7.52 – 7.42 (m, 4H), 7.42 – 7.33 (m, 1H), 7.24 (t, *J* = 7.4 Hz, 1H), 0.42 (d, *J* = 1.2 Hz, 6H), 0.15 (d, *J* = 0.9 Hz, 9H). **^{13}C{^{1}H} NMR** (75 MHz, $CDCl_3$): δ/ppm = 163.5 (d, *J* = 242.6 Hz), 136.2 (d, *J* =

1.3 Hz), 134.4 (d, J = 11.8 Hz), 132.5 (d, J = 3.8 Hz), 129.2 (d, J = 2.9 Hz), 128.4, 128.3 (d, J = 17.3 Hz), 127.5, 126.8 (d, J = 32.3 Hz), 124.1 (d, J = 3.4 Hz), 1.9, 1.5 (d, J = 1.6 Hz). **^{19}F{^{1}H} NMR** (282 MHz, $CDCl_3$): δ/ppm = -106.40. **^{1}H/^{29}Si HMQC NMR** (300/60 MHz, $CDCl_3$): δ/ppm = 7.5, 0.42/-3.5, 0.15/9.2. **HRMS (ESI)** m/z: $[M+H]^+$ Calcd for $C_{17}H_{23}FOSi_2H$ 319.1344. Found 319.1345.

<u>Silanol synthesis:</u>

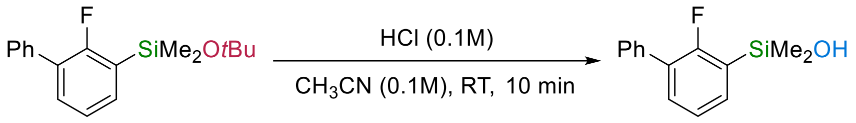

21a **36**: 79%

Adapting a reported procedure,[11] an aqueous solution of HCl (0.1M, 0.4 mL, 4 mol%) was added at room temperature to a solution of **21a** (302.5 mg, 1 mmol, 1 eq.) in acetonitrile (10 mL). The reaction mixture was stirred at room temperature for 10 min and the medium neutralized by adding a saturated aqueous solution of $NaHCO_3$ until pH = 8 (ca. 5 mL). The aqueous layer was extracted with toluene (3 × 20 mL). The combined organic phases were dried over $MgSO_4$, filtrated and concentrated by rotary evaporation. Purification on a pad of silica gel using PE/CH_2Cl_2 (90:10 v/v) then AcOEt (200 mL) afforded **36** (195.1 mg, 0.79 mmol, 79%) as a colorless oil.

^{1}H NMR (300 MHz, $CDCl_3$): δ/ppm = 7.58 – 7.53 (m, 2H), 7.52 – 7.42 (m, 4H), 7.41 – 7.34 (m, 1H), 7.25 (t, J = 7.4 Hz, 1H), 2.12 (br s, 1H), 0.48 (d, J = 1.1 Hz, 6H). **^{13}C{^{1}H} NMR** (75 MHz, $CDCl_3$): δ/ppm = 163.8 (d, J = 241.6 Hz), 136.1, 134.2 (d, J = 11.5 Hz), 133.0 (d, J = 4.0 Hz), 129.3 (d, J = 2.9 Hz), 128.8, 128.6, 127.8, 126.1 (d, J = 31.8 Hz), 124.6 (d, J = 3.3 Hz), 0.7 (d, J = 1.5 Hz). **^{19}F{^{1}H} NMR** (282 MHz, $CDCl_3$): δ/ppm = -106.91. **^{1}H/^{29}Si HMQC NMR** (300/60 MHz, $CDCl_3$): δ/ppm = 7.5, 0.48/7.2. **HRMS (ESI)** m/z: $[M+H]^+$ Calcd for $C_{14}H_{15}FOSiH$ 247.0949. Found 247.0947.

<u>Tamao-Fleming oxidation:</u>

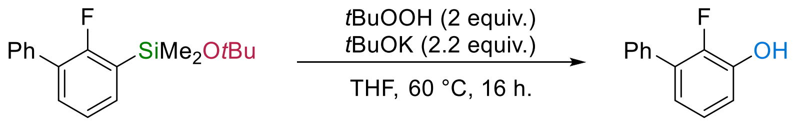

21a **37**: 80%

Adapting a reported procedure,[4] a flame-dried Schlenk equipped with a magnetic stirring bar was charged with potassium *tert*-butoxide (98.7 mg, 0.88 mmol, 2.2 eq.) and THF (1.4 mL). *Tert*-butyl hydroperoxide (5.5 M in decane, 146 µL, 0.8 mmol, 2 eq.) and **21a** (121 mg, 0.4 mmol, 1 eq.) in THF (1 mL) were then added at 0 °C and the resulting mixture was stirred at 60 °C for ca. 16 h. To the mixture were then added a saturated aqueous solution of $NaHSO_3$

(5 mL), HCl (2 M, 5 mL) and water (10 mL). The aqueous layer was extracted with AcOEt (3 × 10 mL), the combined organic phases were dried over $MgSO_4$, filtrated and evaporated. Purification by column chromatography on silica gel using PE/AcOEt (95:5 → 90:10 v/v) as eluent afforded compound **37** (60.5 mg, 0.32 mmol, 80%) as a white solid.

^{1}H NMR (400 MHz, $CDCl_3$): δ/ppm = 7.59 – 7.51 (m, 2H), 7.49 – 7.43 (m, 2H), 7.42 – 7.36 (m, 1H), 7.10 (td, *J* = 7.9, 1.3 Hz, 1H), 7.05 – 6.93 (m, 2H), 5.26 (br s, 1H). **^{13}C{^{1}H} NMR** (101 MHz, $CDCl_3$): δ/ppm = 148.2 (d, *J* = 238.0 Hz), 144.2 (d, *J* = 15.3 Hz), 135.5, 129.7 (d, *J* = 11.3 Hz), 129.1 (d, *J* = 2.9 Hz), 128.6, 128.0, 124.7 (d, *J* = 4.7 Hz), 122.0, 116.2 (d, *J* = 1.8 Hz). **^{19}F{^{1}H} NMR** (376 MHz, $CDCl_3$): δ/ppm = -147.09. The spectroscopic data match the reported literature.[13]

<u>Ipso-iodination:</u>

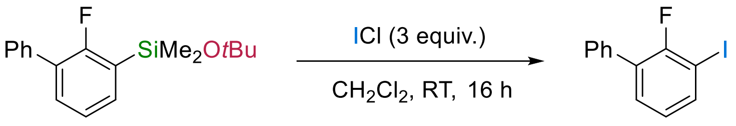

21a → **38**: 99%

A flame-dried and argon flushed round-bottomed flask, equipped with a magnetic stirring bar and a rubber septum, was charged with **21a** (106 mg, 0.35 mmol, 1 eq.) and CH_2Cl_2 (3.5 mL). To the resulting solution was then dropwise iodide monochloride (1.05 mL (1M in CH_2Cl_2),1.05 mmol, 3 eq.). After stirring for ca. 16 h, a solution of saturated aqueous $NaHSO_3$ (3 mL) was added and the aqueous layer was extracted with CH_2Cl_2 (3 × 5 mL). The combined organic layers were dried over MgSO4, filtered and concentrated under *vacuo*. Purification by column chromatography on silica gel using petroleum ether as eluent afforded compound **38** (103.1 mg, 0.35 mmol, 99%) as a white solid.

M.p ($CHCl_3$) = 73-75 °C. **^{1}H NMR** (300 MHz, $CDCl_3$): δ/ppm = 7.74 (ddd, J = 7.7, 5.7, 1.7 Hz, 1H), 7.56 – 7.50 (m, 2H), 7.49 – 7.43 (m, 2H), 7.43 – 7.36 (m, 2H), 6.97 (td, J = 7.8, 0.7 Hz, 1H). **^{13}C{^{1}H} NMR** (75 MHz, $CDCl_3$): δ/ppm = 158.5 (d, *J* = 246.8 Hz), 138.6 (d, *J* = 1.6 Hz), 135.3 (d, *J* = 1.7 Hz), 131.2 (d, *J* = 3.1 Hz), 130.1 (d, *J* = 16.2 Hz), 129.1 (d, *J* = 2.9 Hz), 128.6, 128.2, 126.0 (d, *J* = 4.3 Hz), 82.6 (d, *J* = 27.1 Hz). **^{19}F{^{1}H} NMR** (282 MHz, $CDCl_3$): δ/ppm = -96.72. *Note*: HRMS could not be obtained for this compound as ionization using ESI or APCI techniques coupled with MeCN elution did not occur.

7. NMR spectra of new compounds

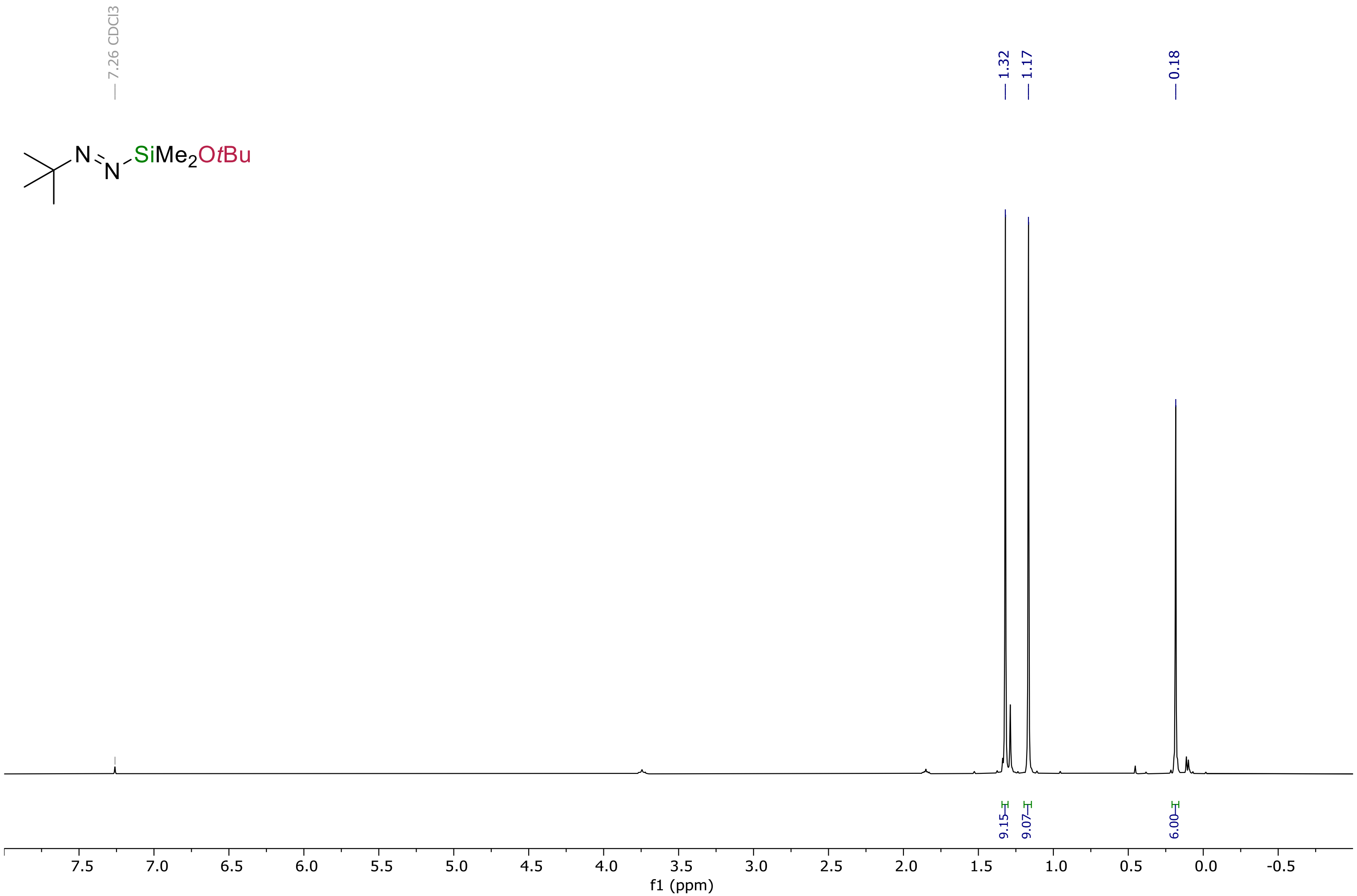

Figure S6: ^{1}H NMR (300 MHz, $CDCl_3$) of compound **3a**.

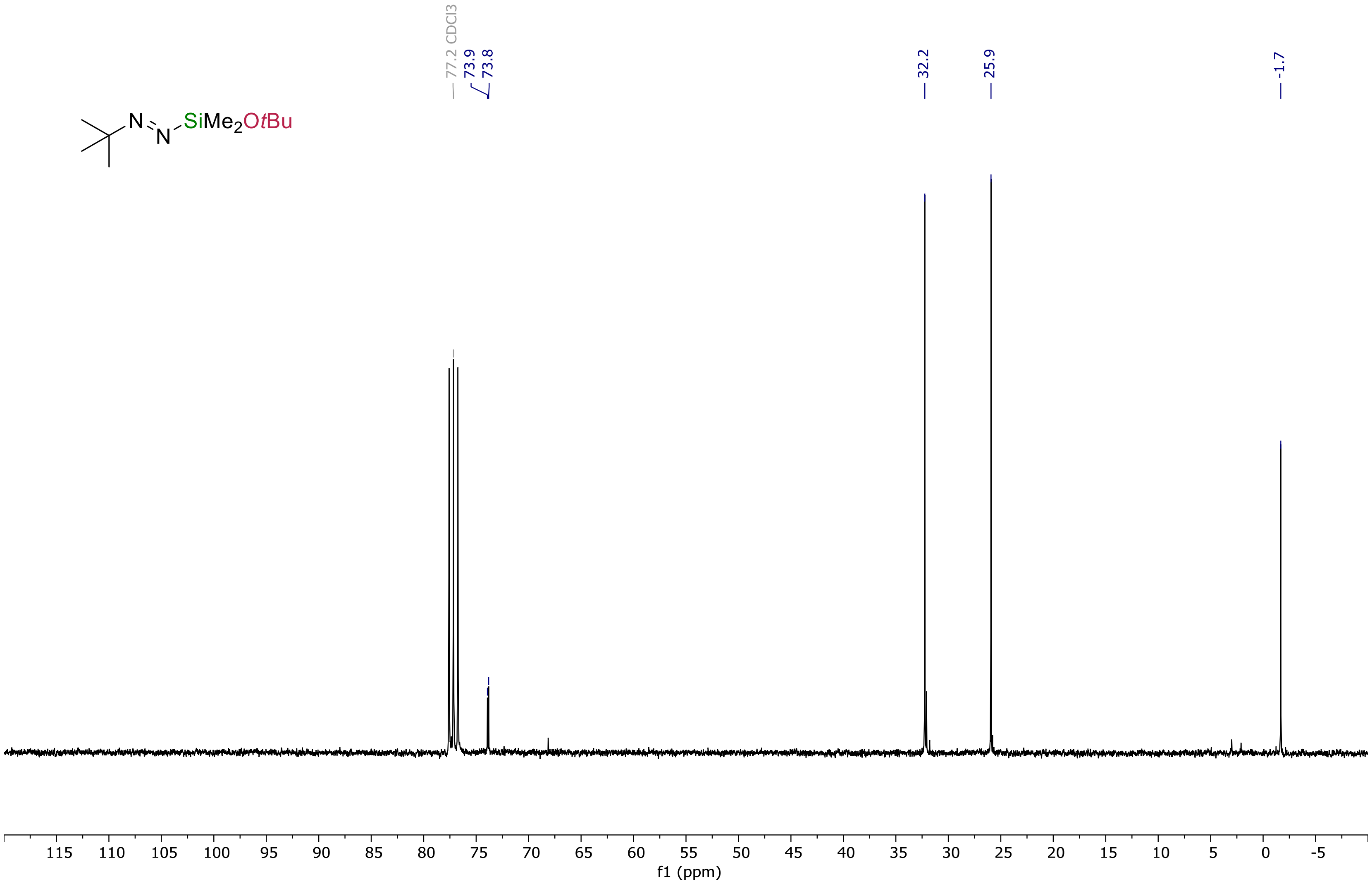

Figure S7: $^{13}C\{^{1}H\}$ NMR (75 MHz, $CDCl_3$) of compound **3a**.

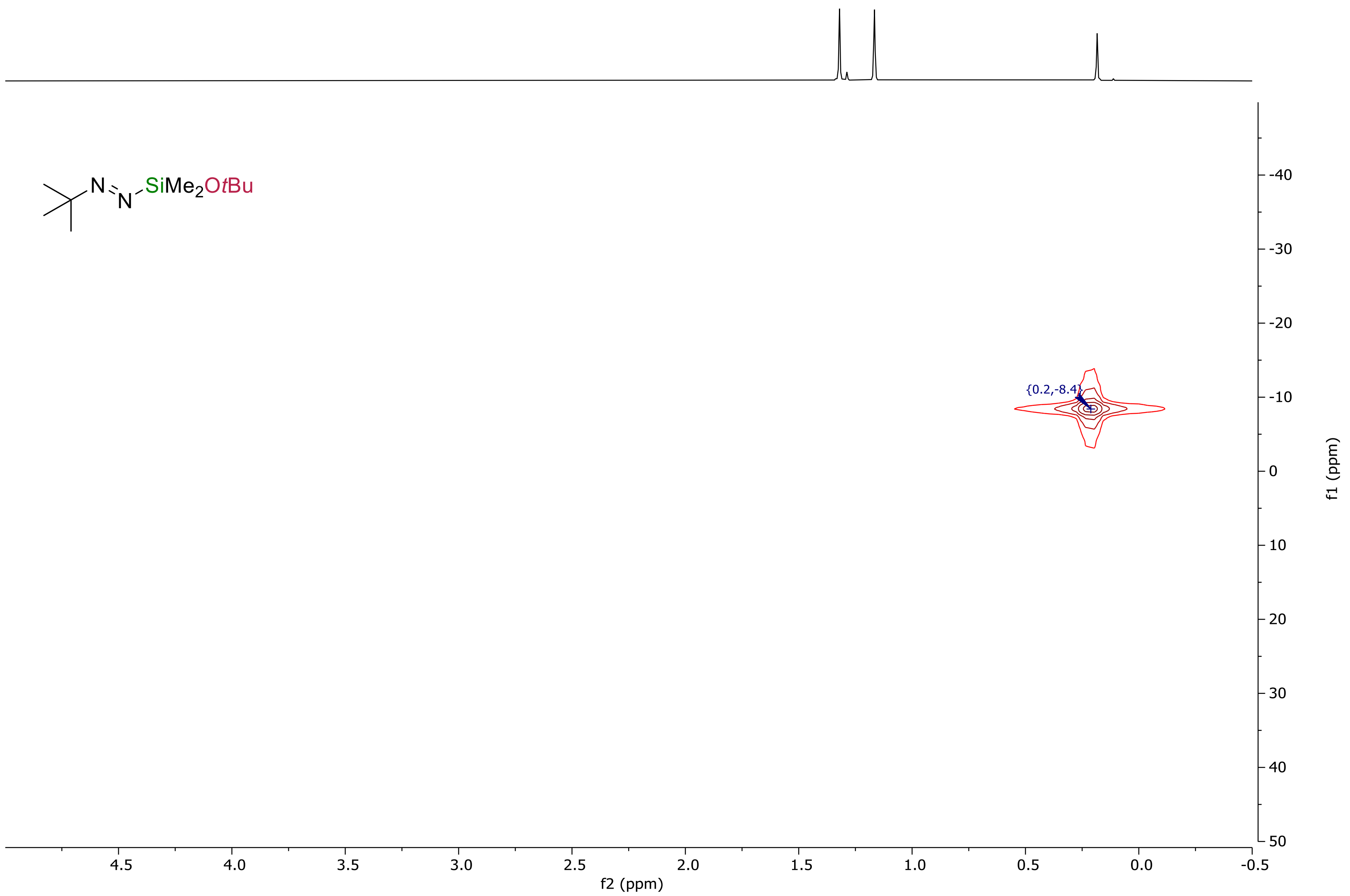

Figure S8: $^{1}H/^{29}Si$ HMQC NMR (300/60 MHz, $CDCl_3$) of compound **3a**.

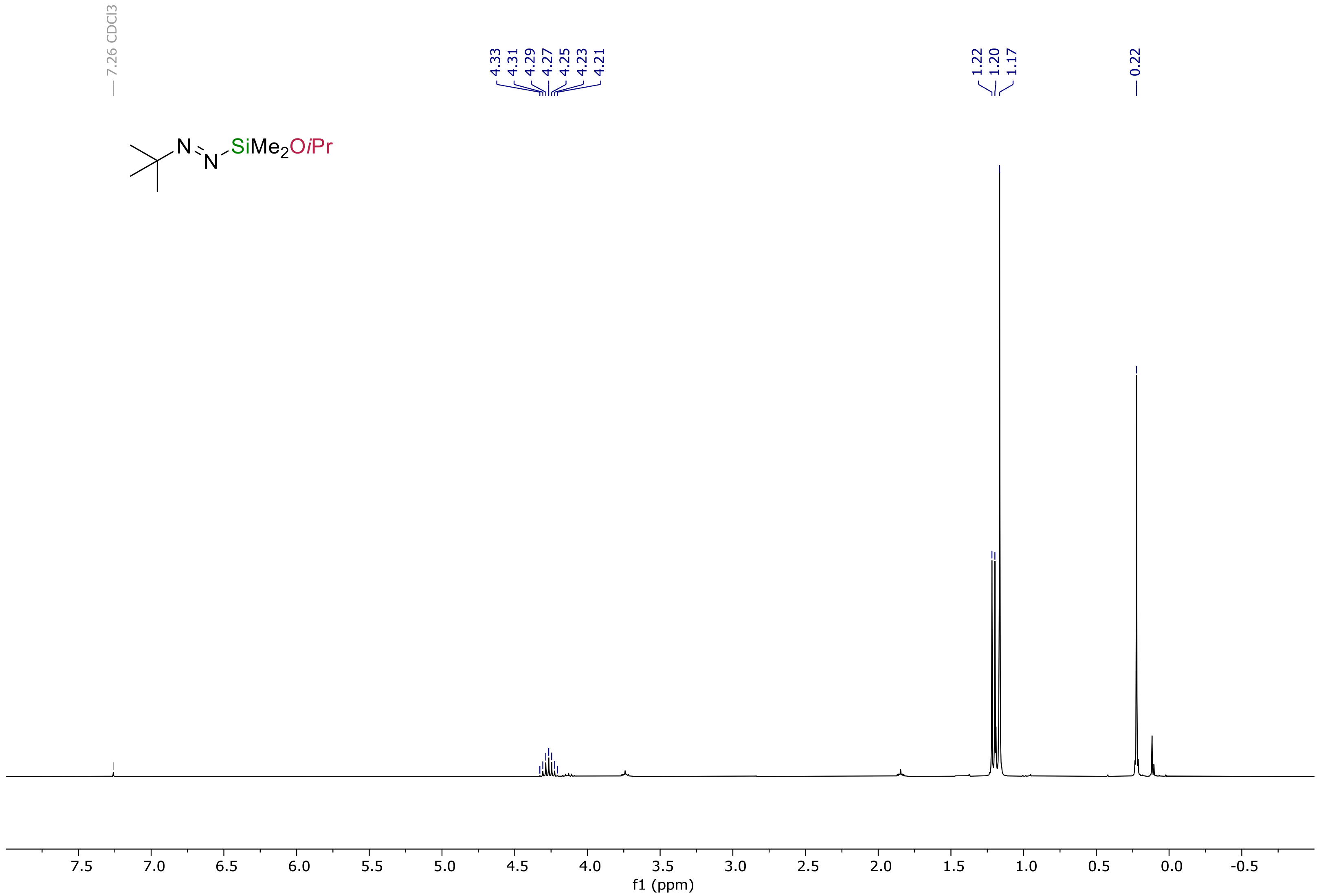

Figure S9: ^1H NMR (300 MHz, $CDCl_3$) of compound **3b**.

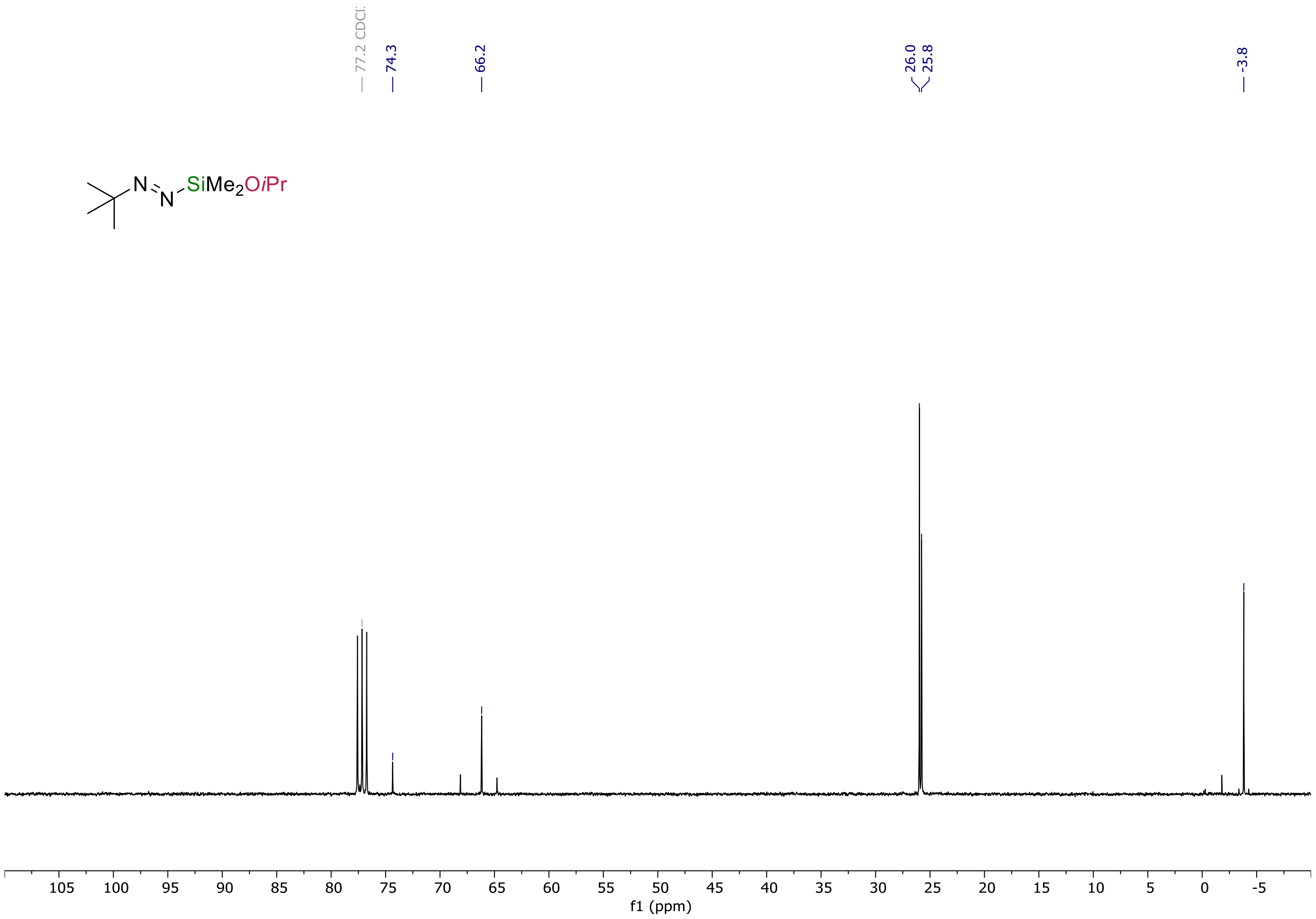

Figure S10: $^{13}C\{^{1}H\}$ NMR (75 MHz, $CDCl_3$) of compound **3b**.

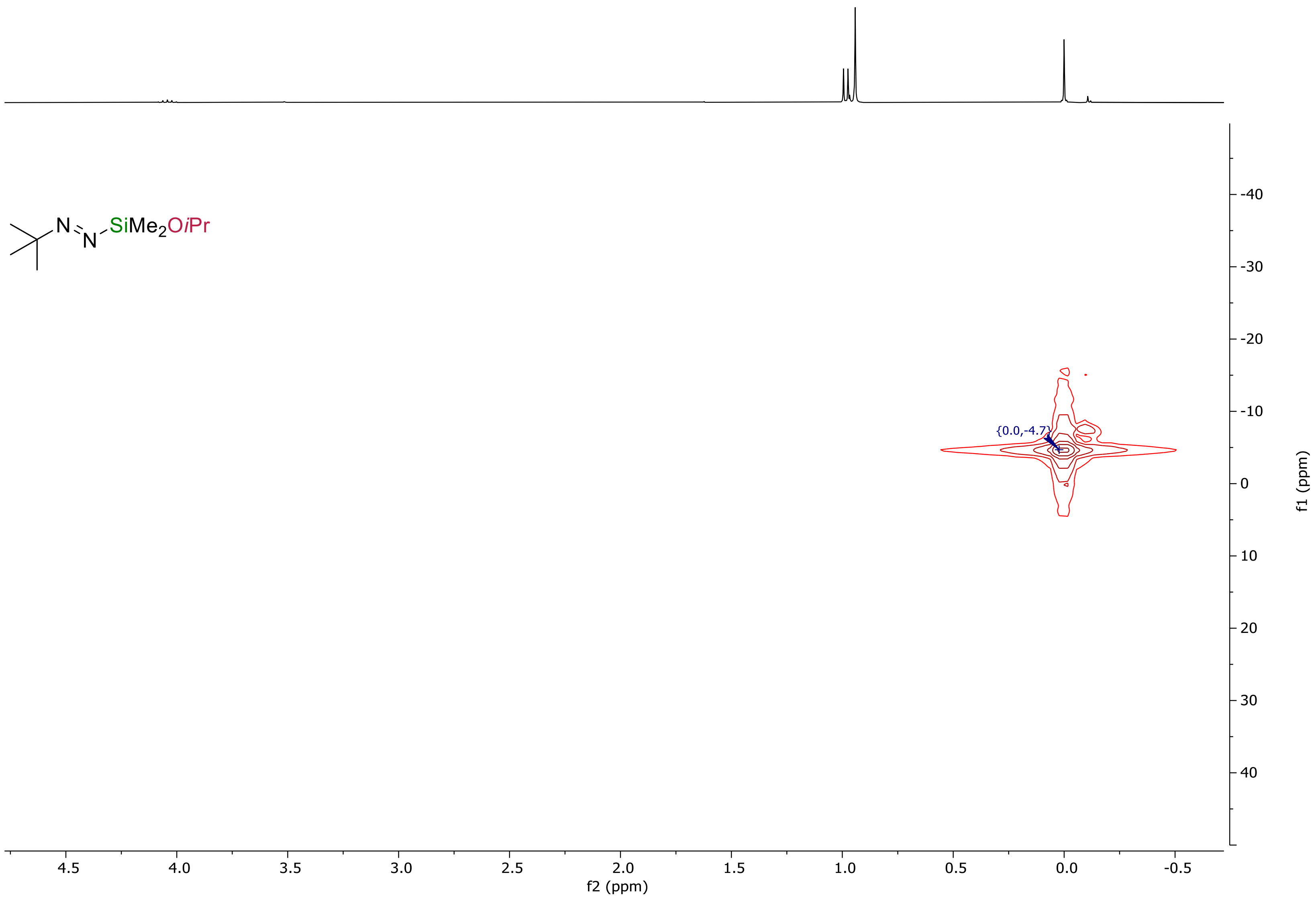

Figure S11: $^{1}H/^{29}Si$ HMQC NMR (300/60 MHz, $CDCl_3$) of compound **3b**.

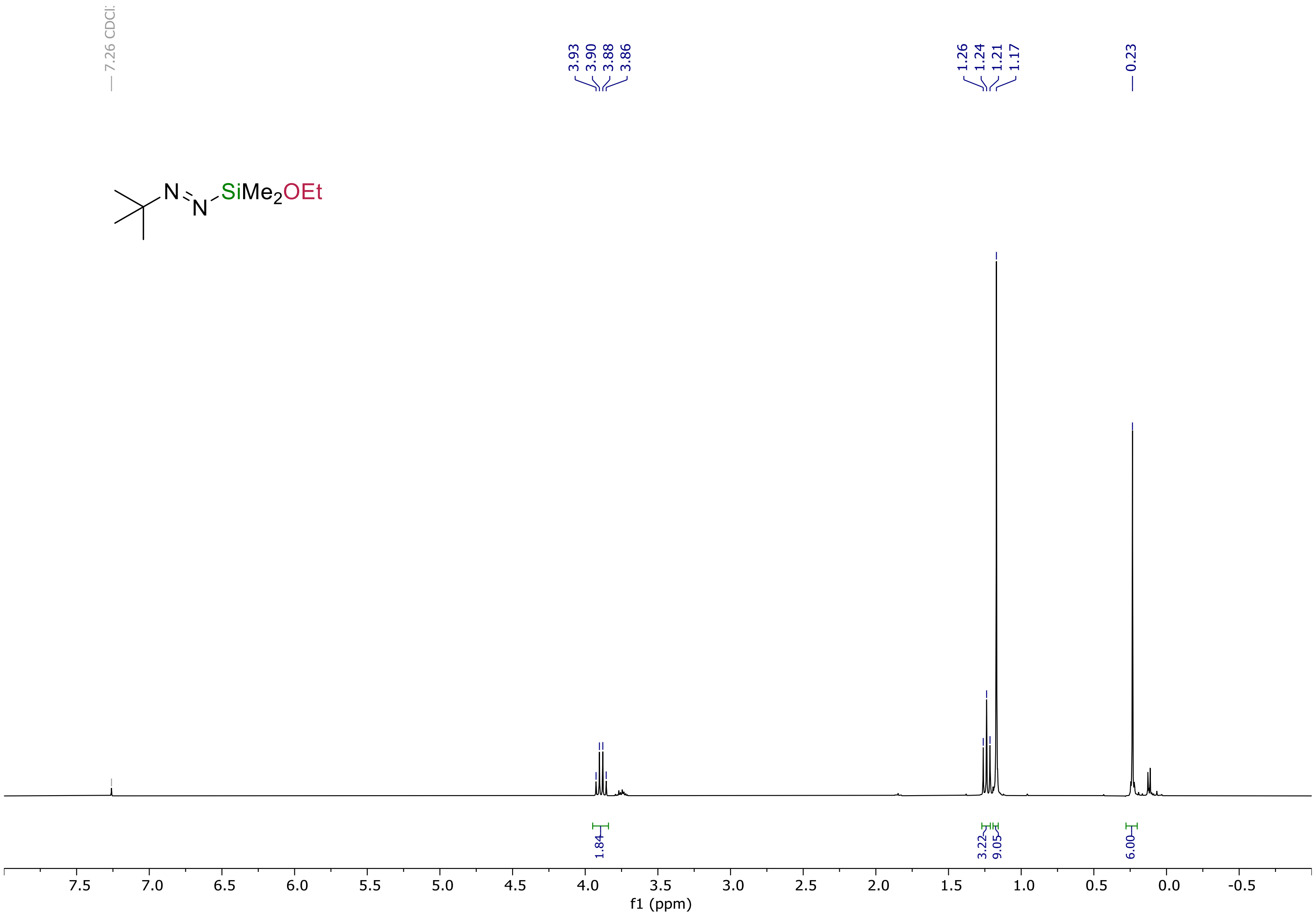

Figure S12: ^{1}H NMR (300 MHz, $CDCl_3$) of compound **3c**.

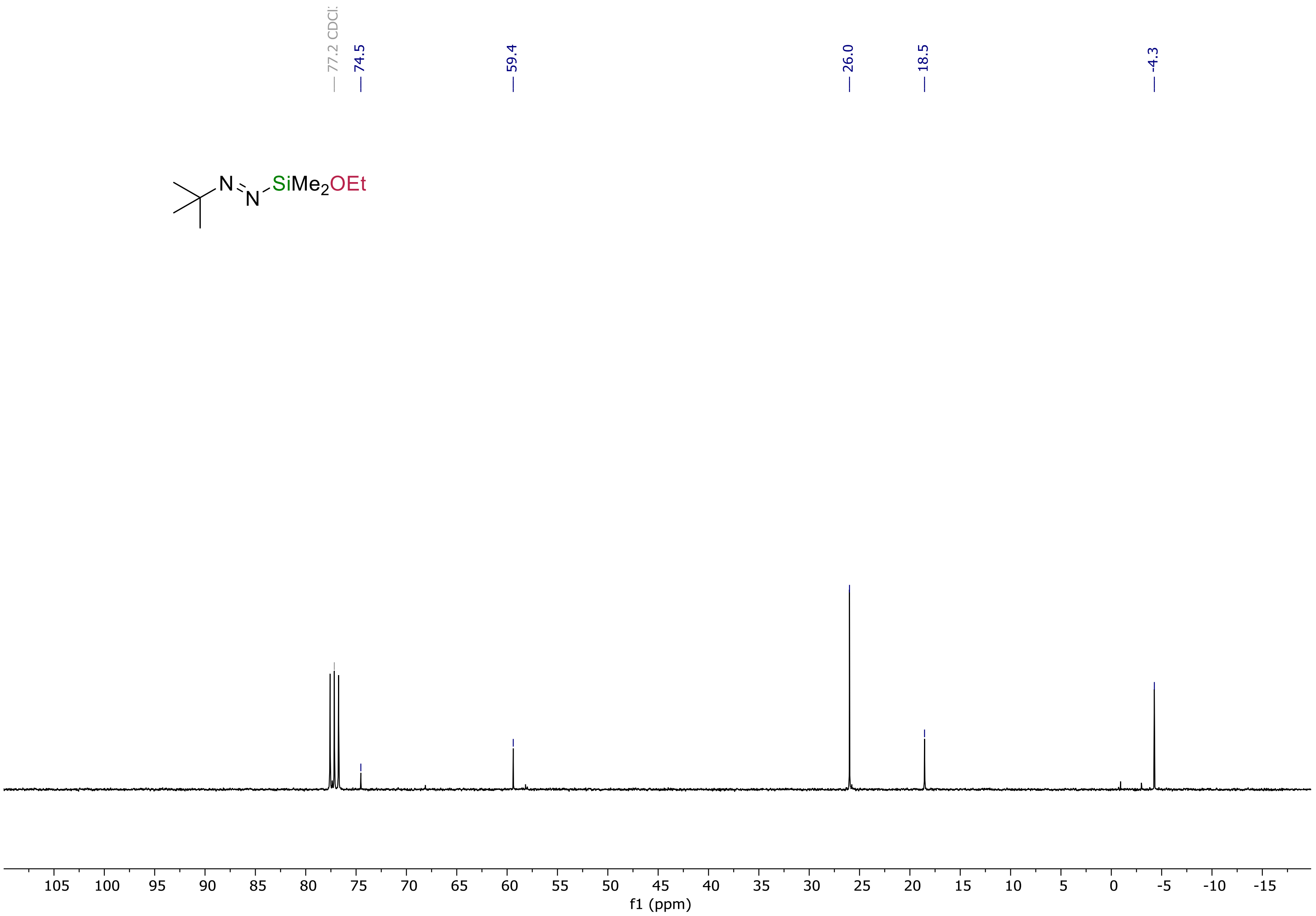

Figure S13: $^{13}C\{^1H\}$ NMR (75 MHz, $CDCl_3$) of compound **3c**.

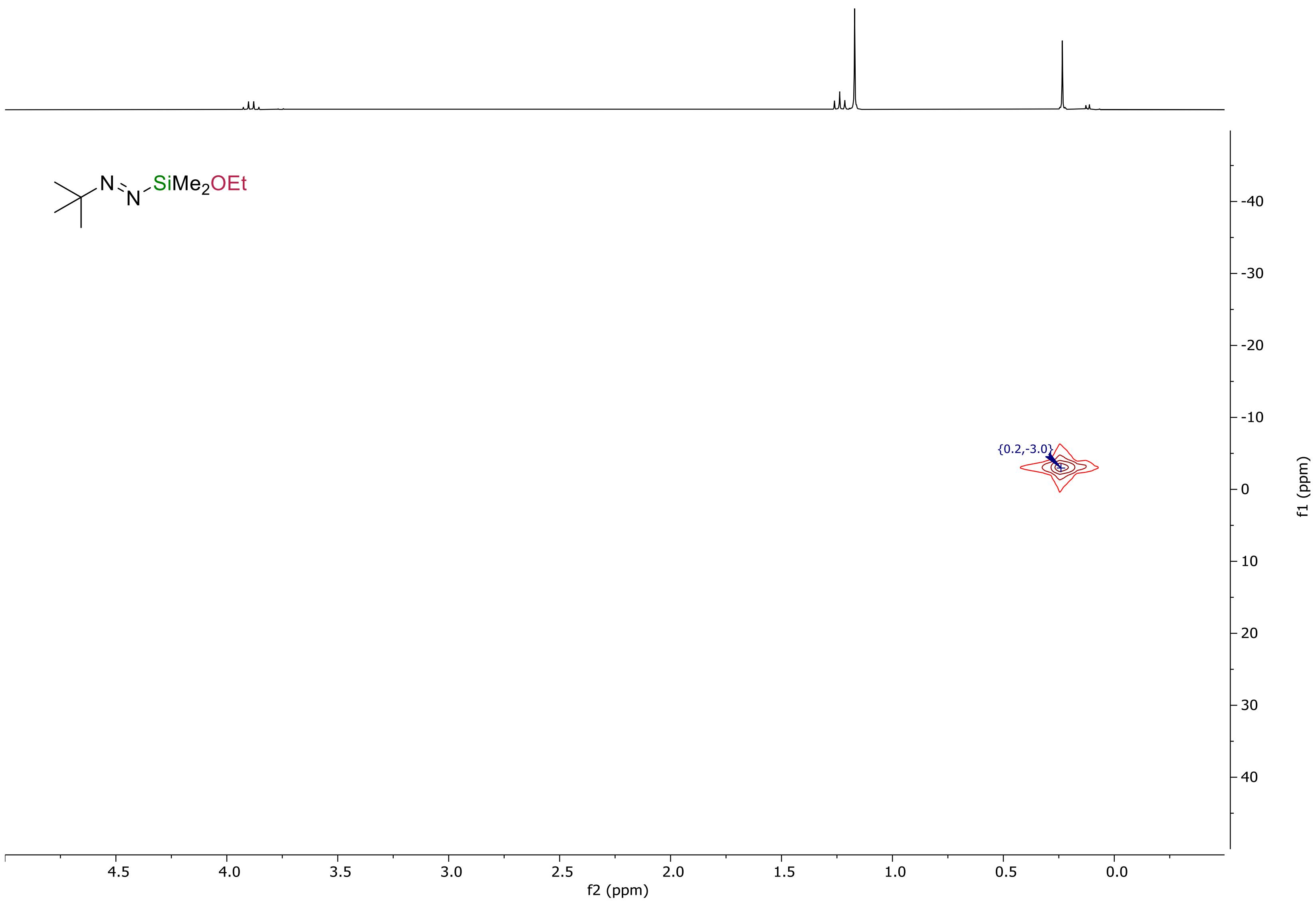

Figure S14: $^{1}H/^{29}Si$ HMQC NMR (300/60 MHz, $CDCl_3$) of compound **3c**.

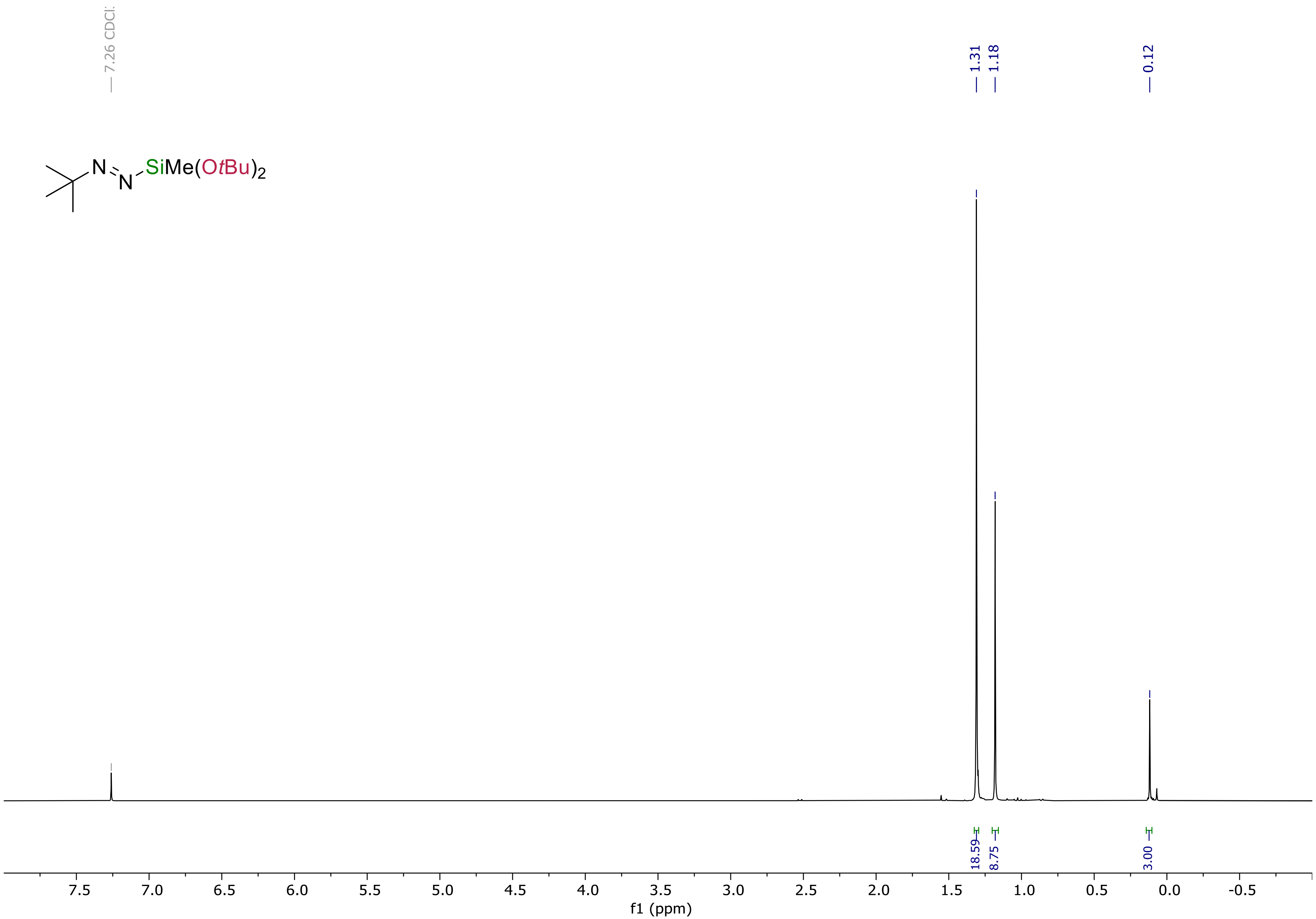

Figure S15: ^1H NMR (300 MHz, $CDCl_3$) of compound **3d**.

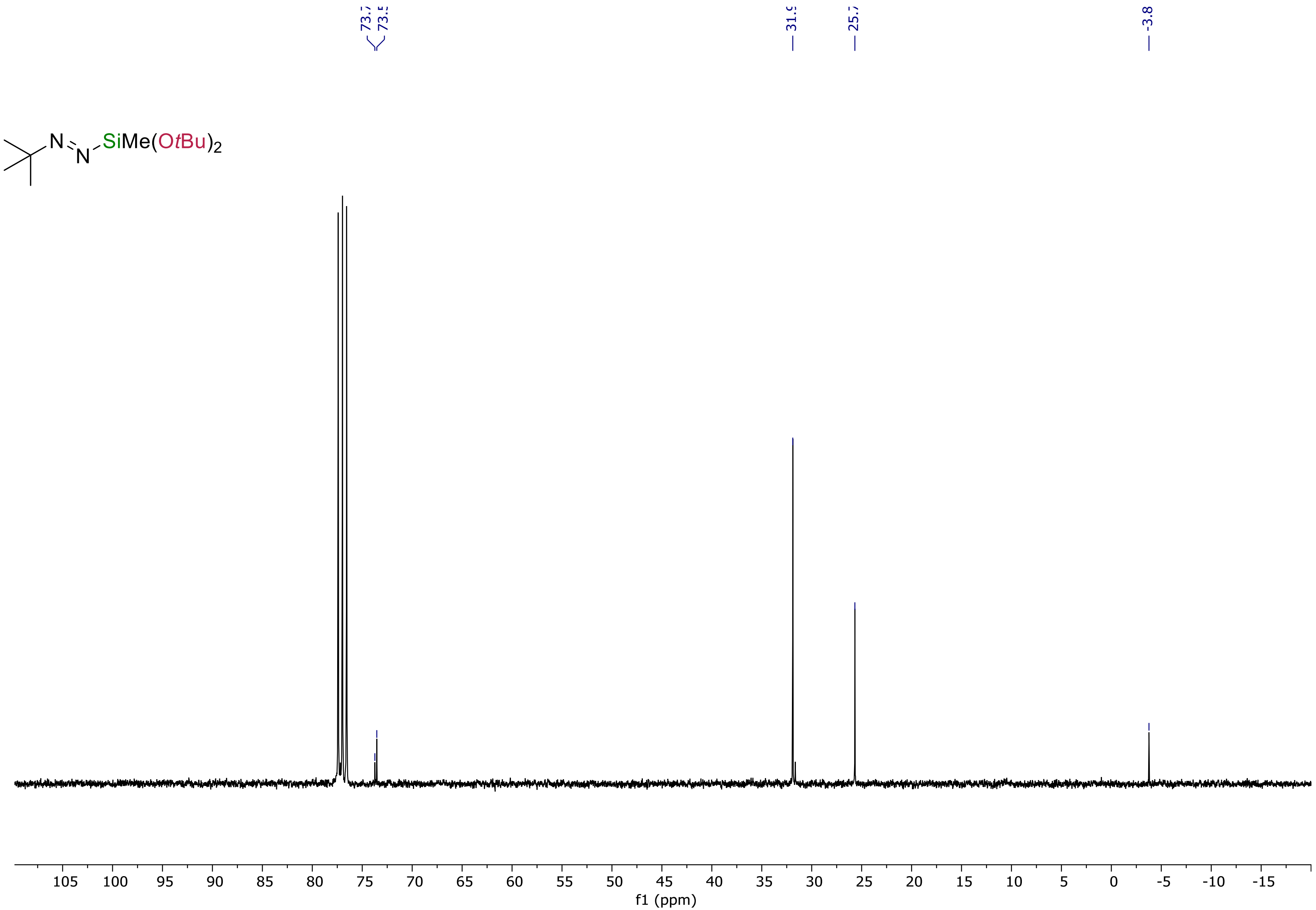

Figure S16: $^{13}C\{^1H\}$ NMR (75 MHz, $CDCl_3$) of compound **3d**.

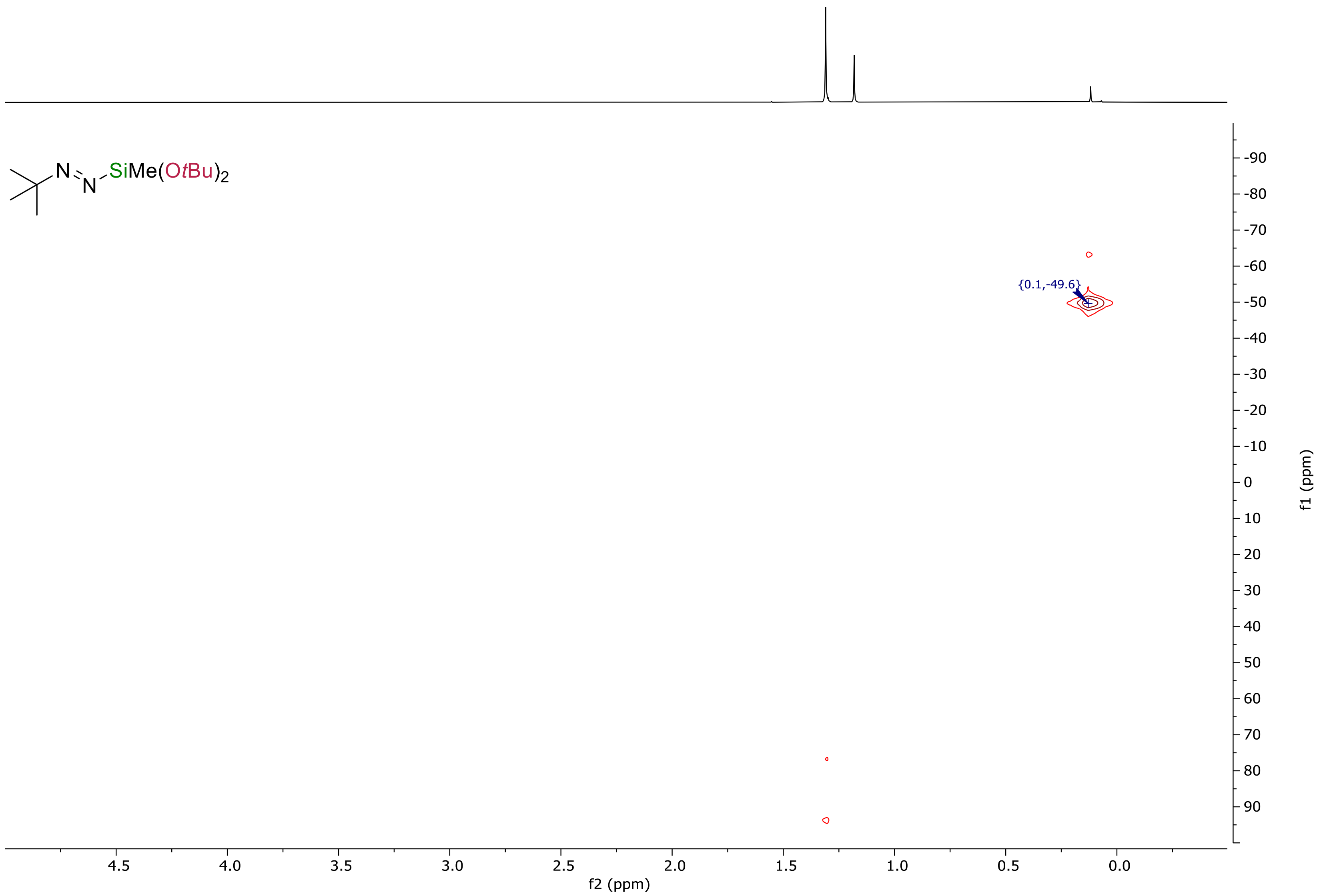

Figure S17: $^{1}H/^{29}Si$ HMQC NMR (300/60 MHz, $CDCl_3$) of compound **3d**.

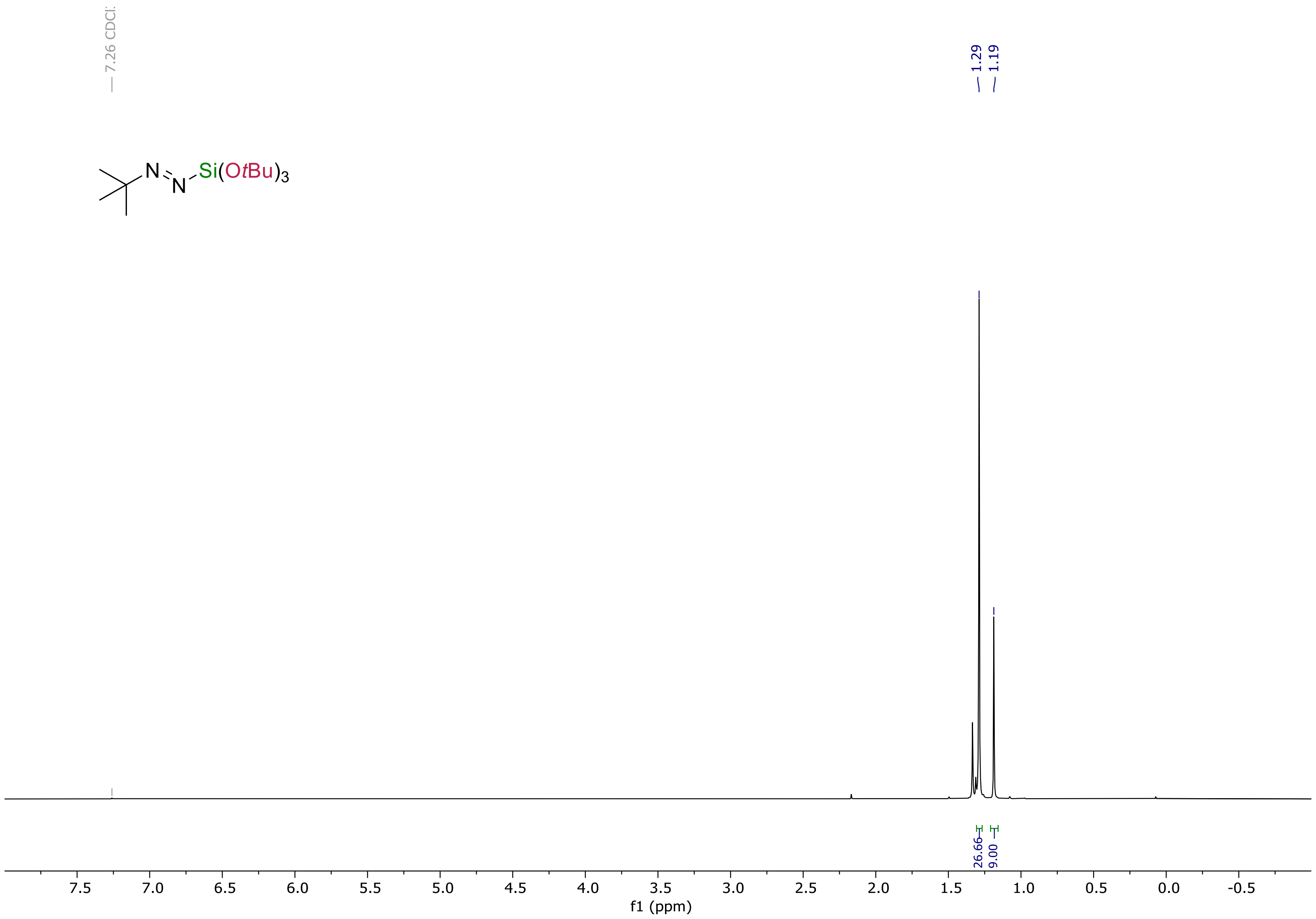

Figure S18: ^{1}H NMR (300 MHz, $CDCl_3$) of compound **3e**.

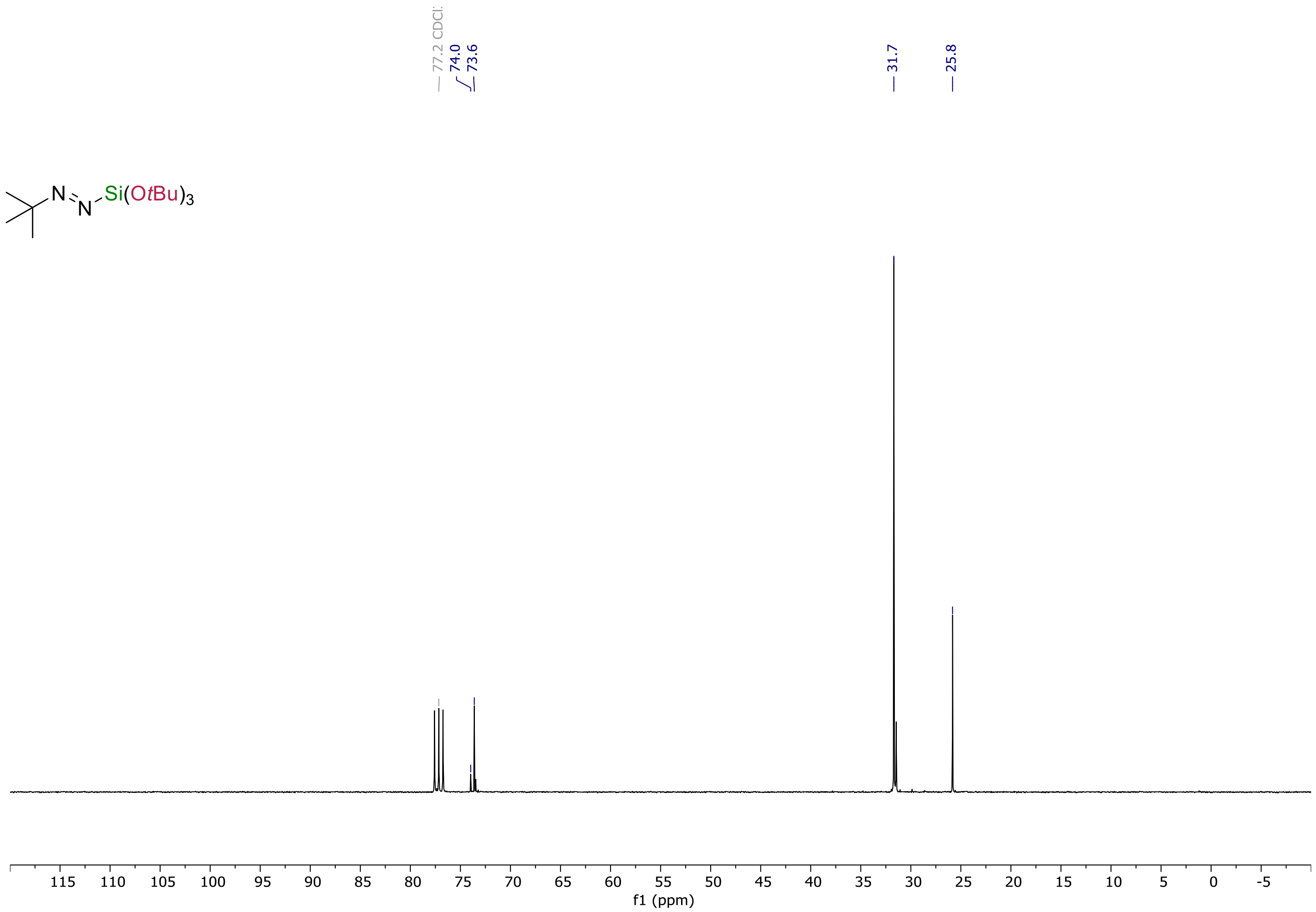

Figure S19: $^{13}C\{^{1}H\}$ NMR (75 MHz, $CDCl_3$) of compound **3e**.

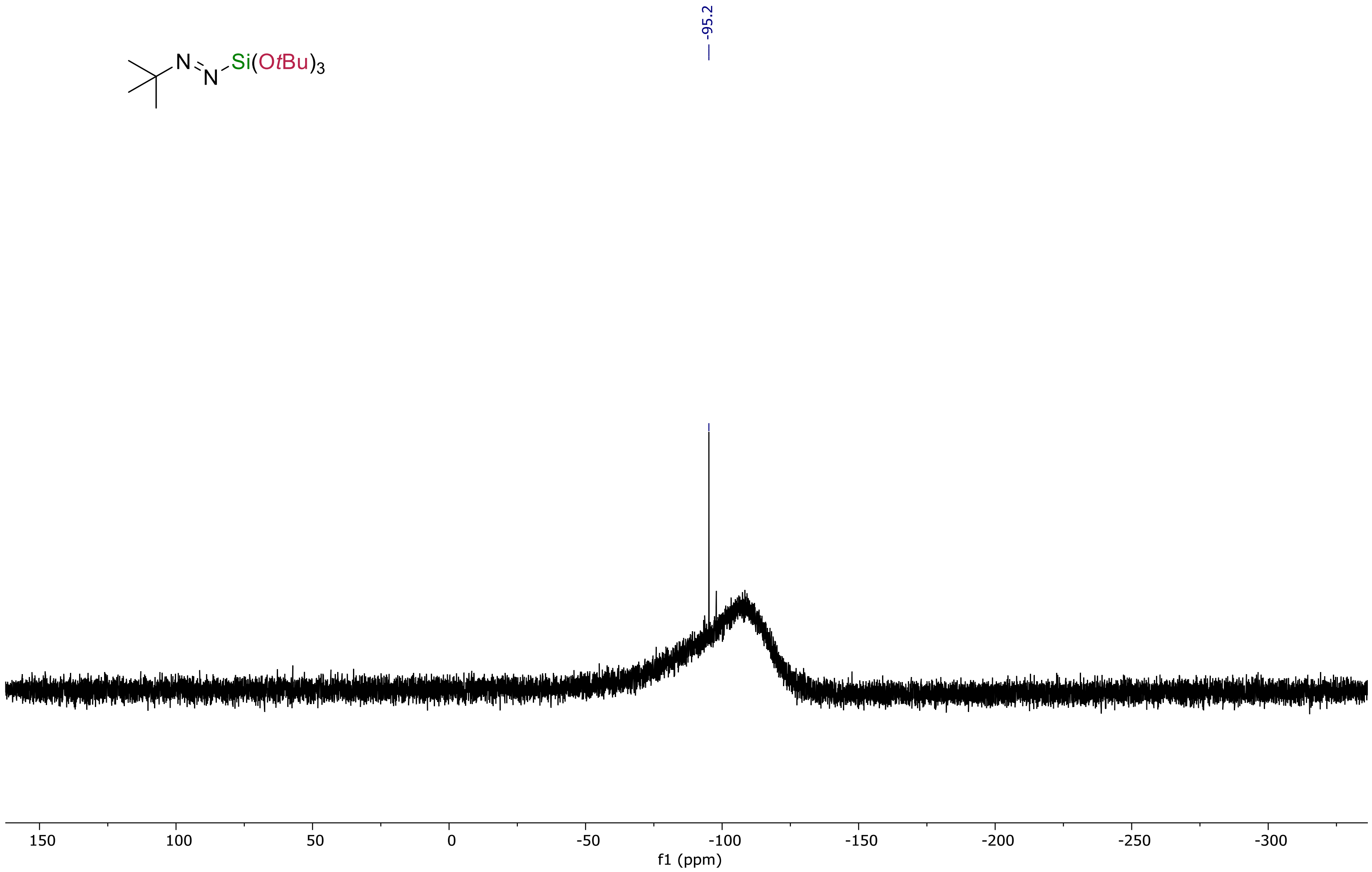

Figure S20: $^{29}Si\{^1H\}$ NMR (60 MHz, $CDCl_3$) of compound **3e**.

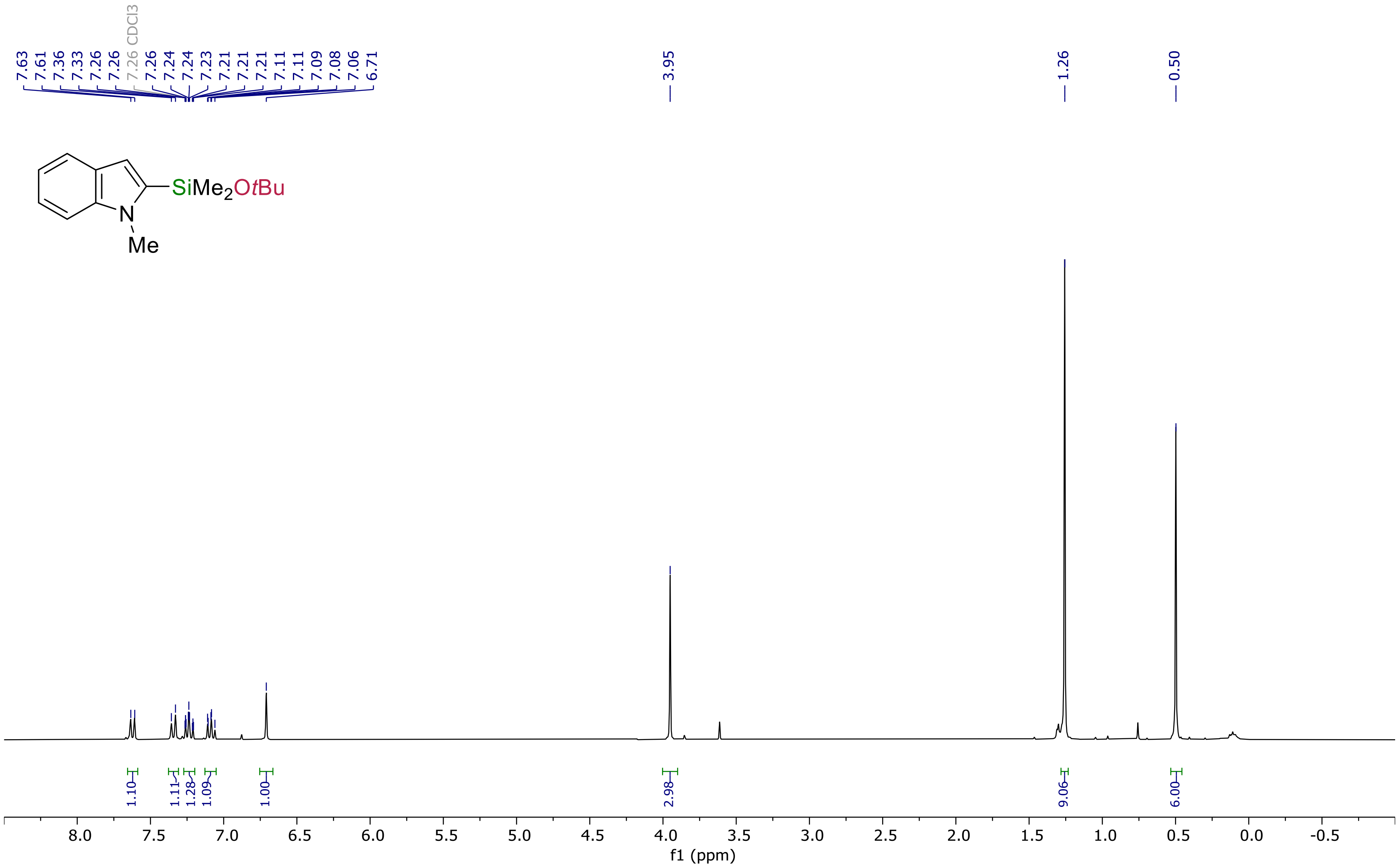

Figure S21: ^{1}H NMR (300 MHz, $CDCl_3$) of compound **4a**.

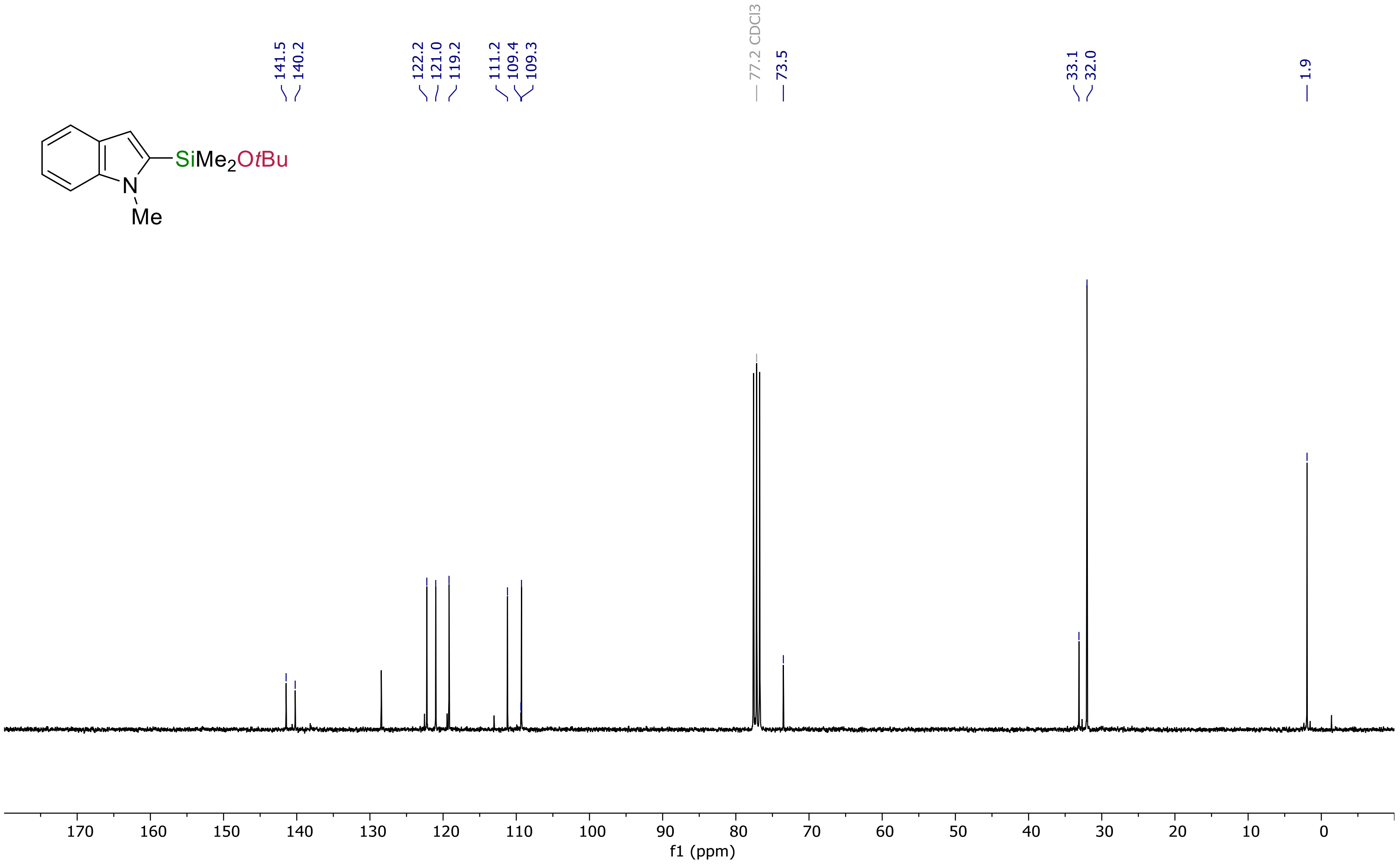

Figure S22: $^{13}C\{^{1}H\}$ NMR (75 MHz, $CDCl_3$) of compound **4a**.

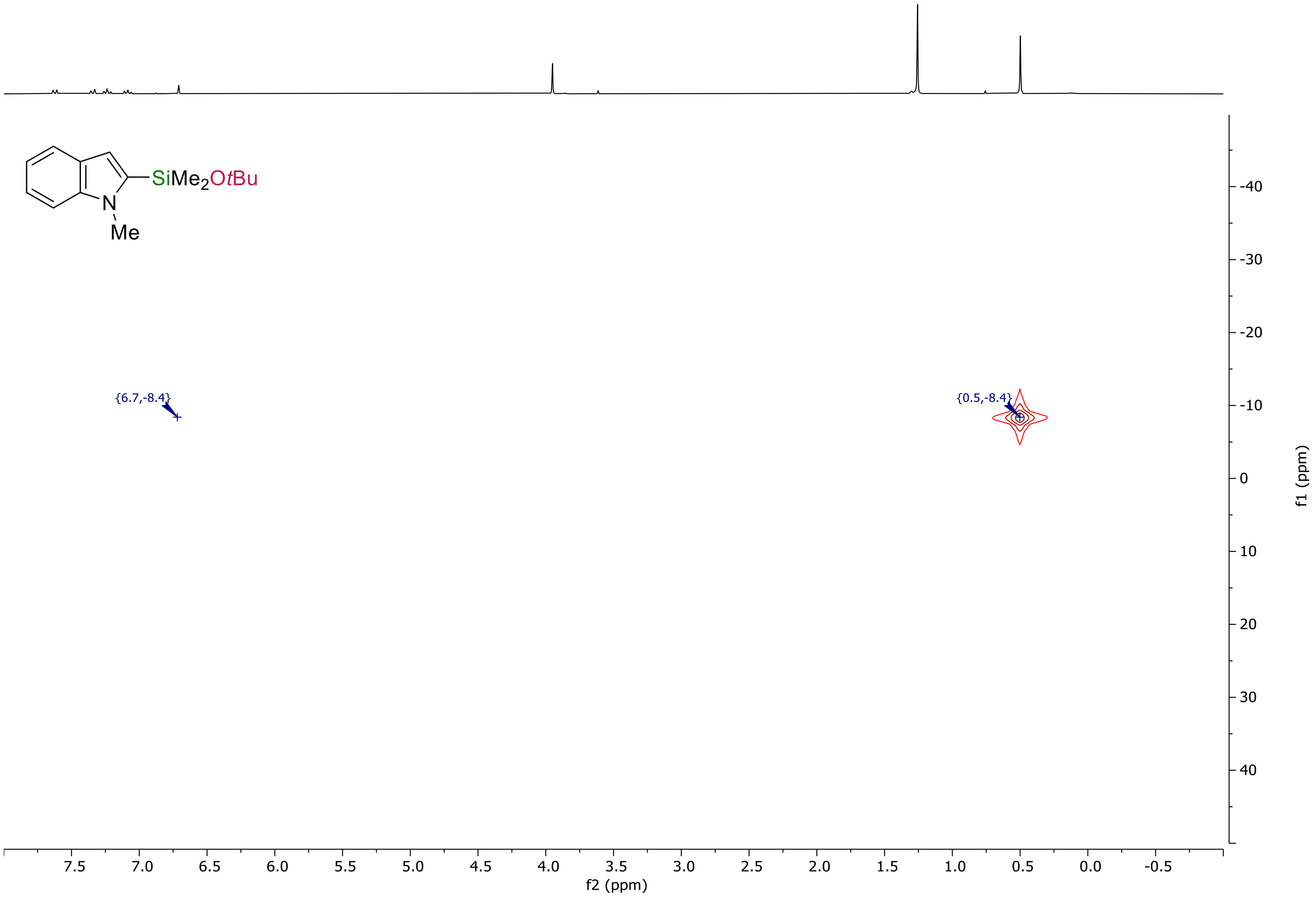

Figure S23: $^{1}H/^{29}Si$ HMQC NMR (300/60 MHz, $CDCl_3$) of compound **4a**.

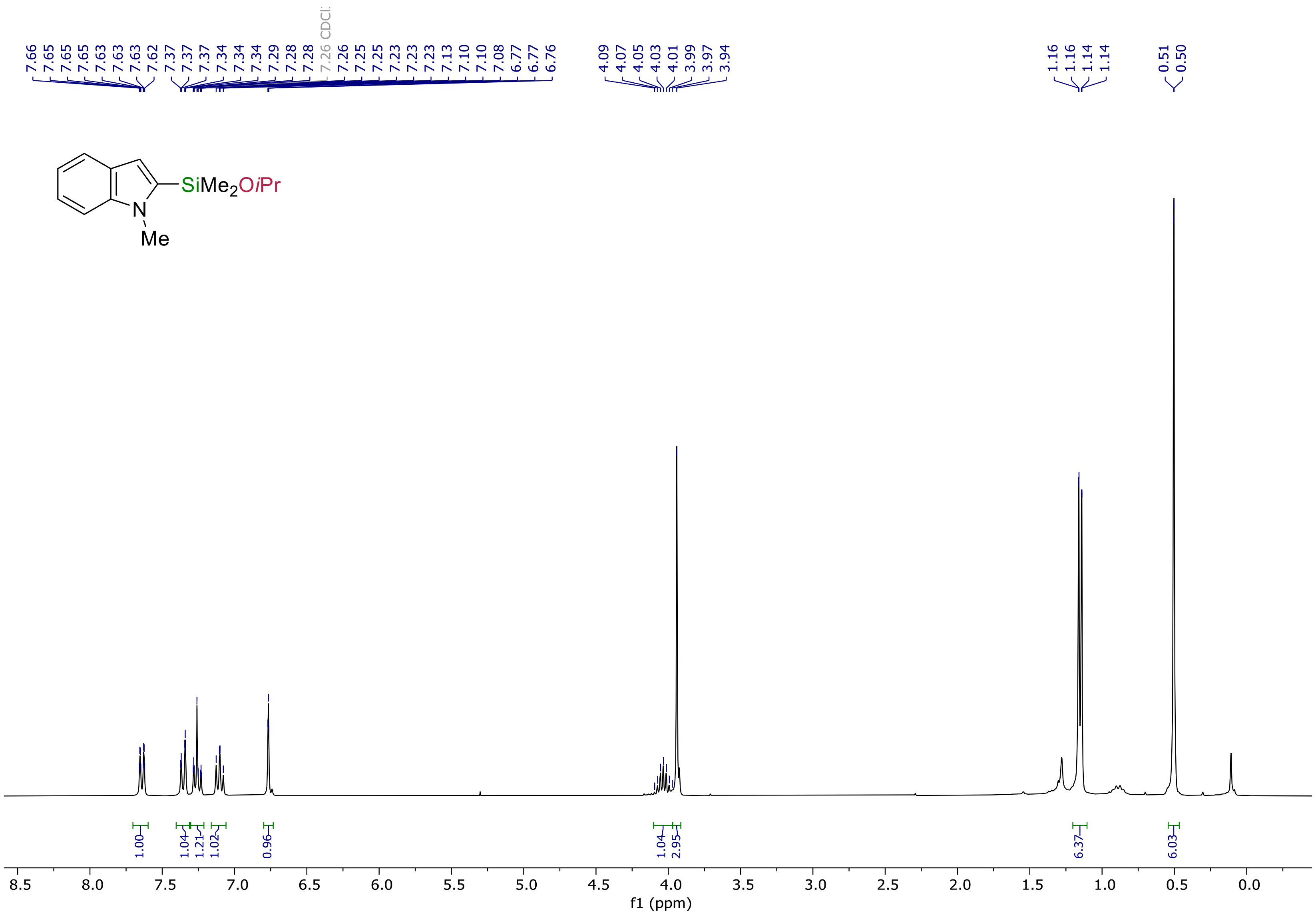

Figure S24: ^{1}H NMR (300 MHz, $CDCl_3$) of compound **4b**.

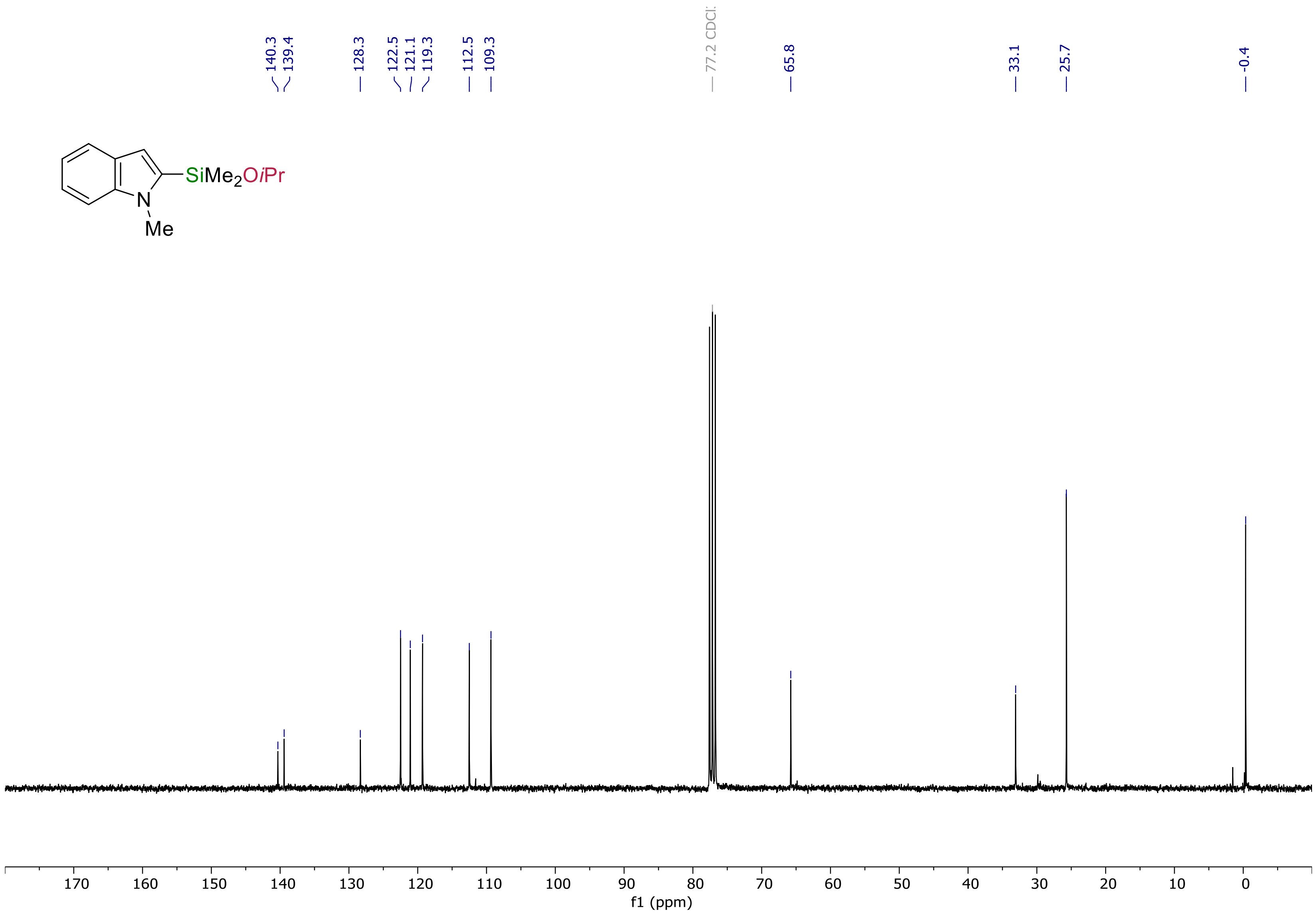

Figure S25: $^{13}C\{^{1}H\}$ NMR (75 MHz, $CDCl_3$) of compound **4b.**

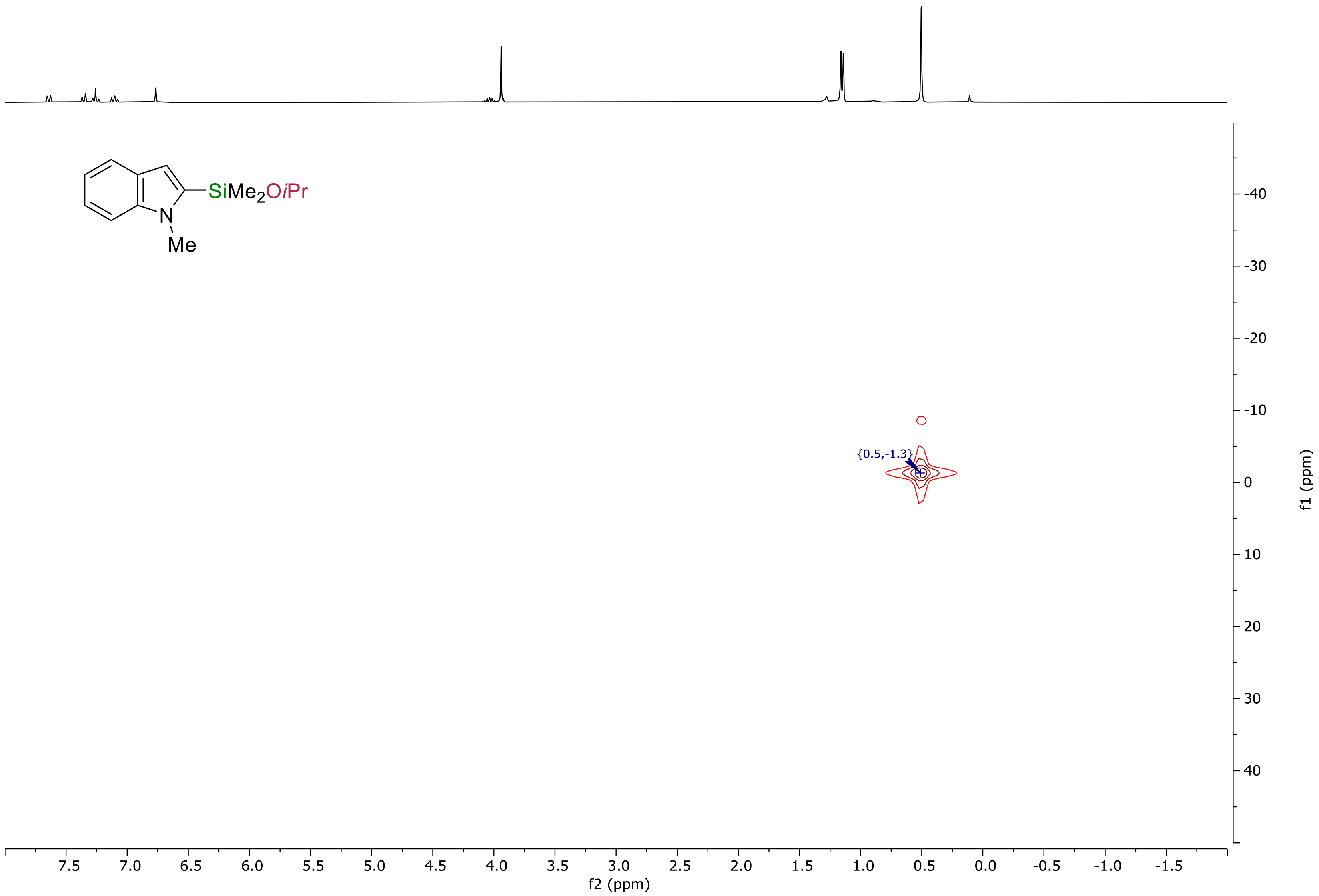

Figure S26: $^{1}H/^{29}Si$ HMQC NMR (300/60 MHz, $CDCl_3$) of compound **4b**.

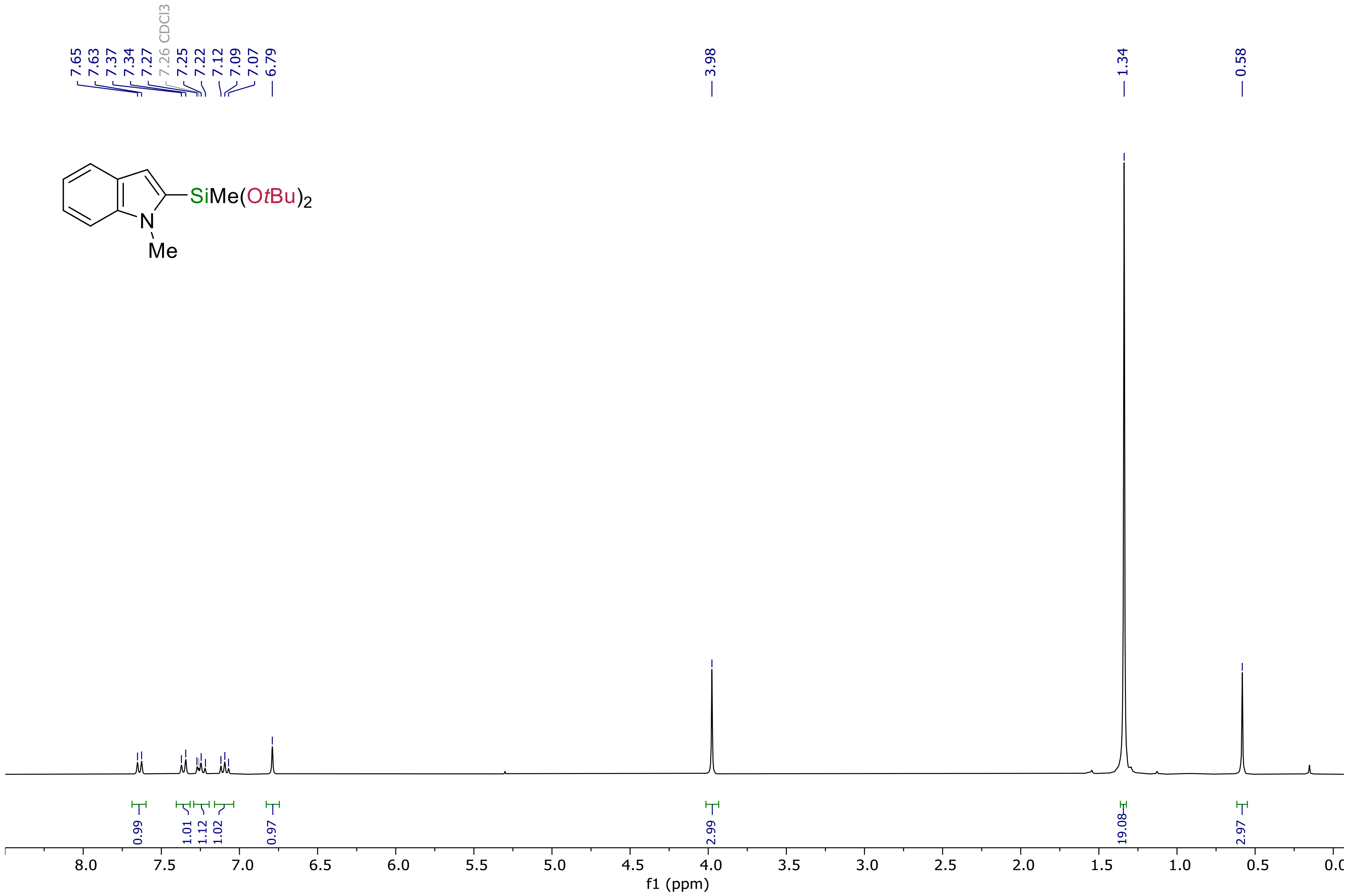

Figure S27: ^{1}H NMR (300 MHz, $CDCl_3$) of compound **4d**.

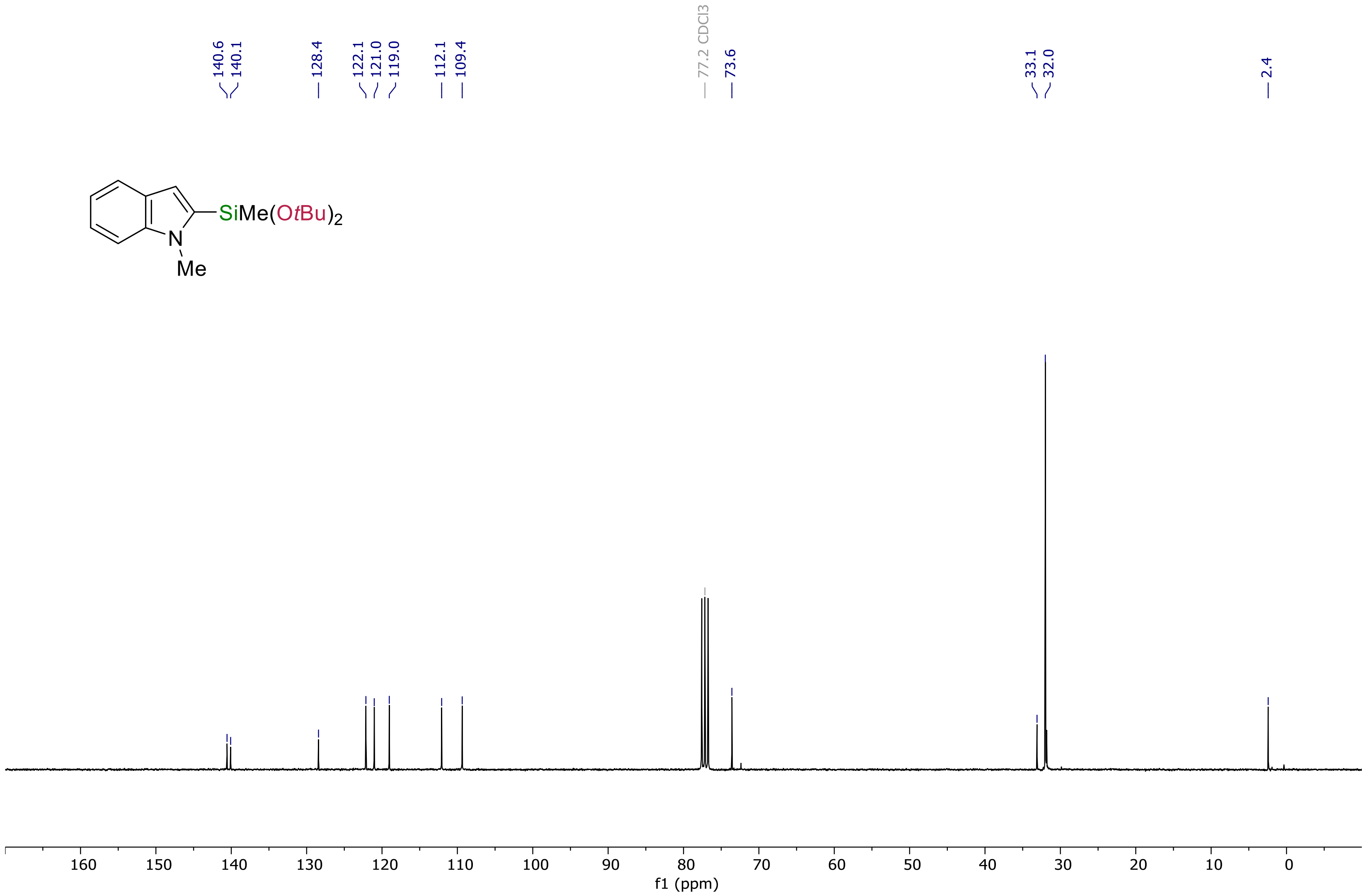

Figure S28: $^{13}C\{^1H\}$ NMR (75 MHz, $CDCl_3$) of compound **4d**.

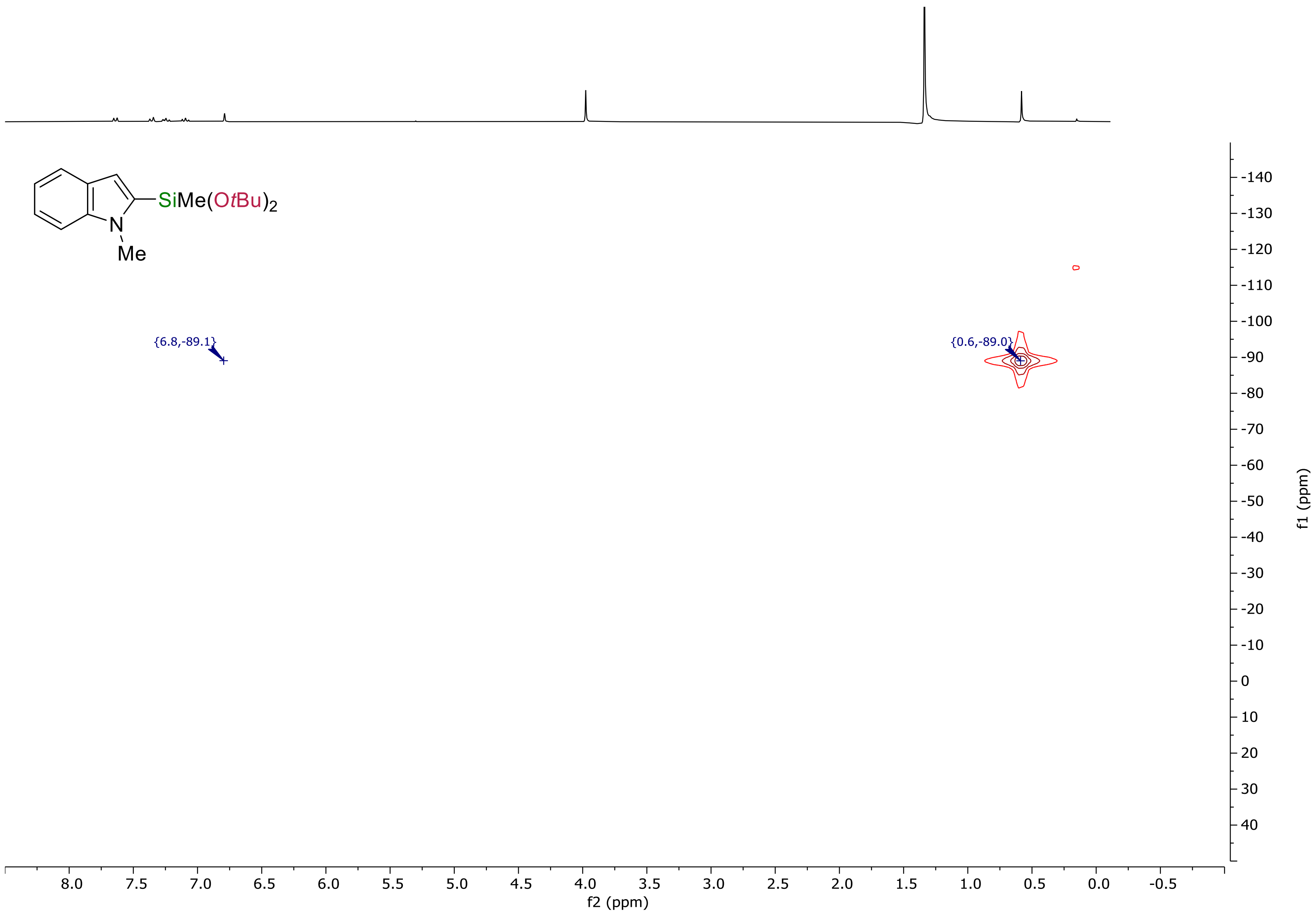

Figure S29: $^{1}H/^{29}Si$ HMQC NMR (300/60 MHz, $CDCl_3$) of compound **4d**.

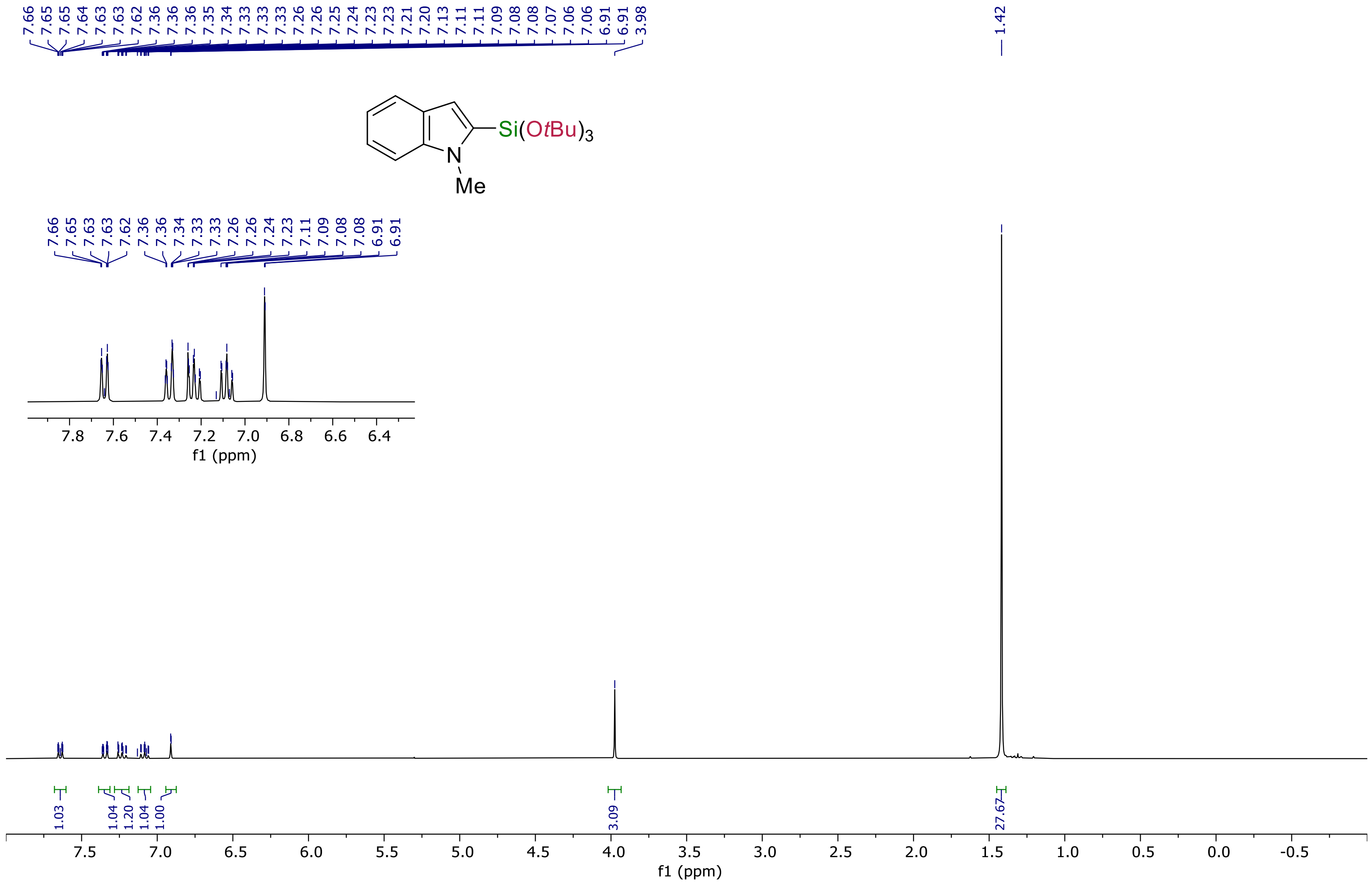

Figure S30: ^{1}H NMR (300 MHz, $CDCl_3$) of compound **4e**.

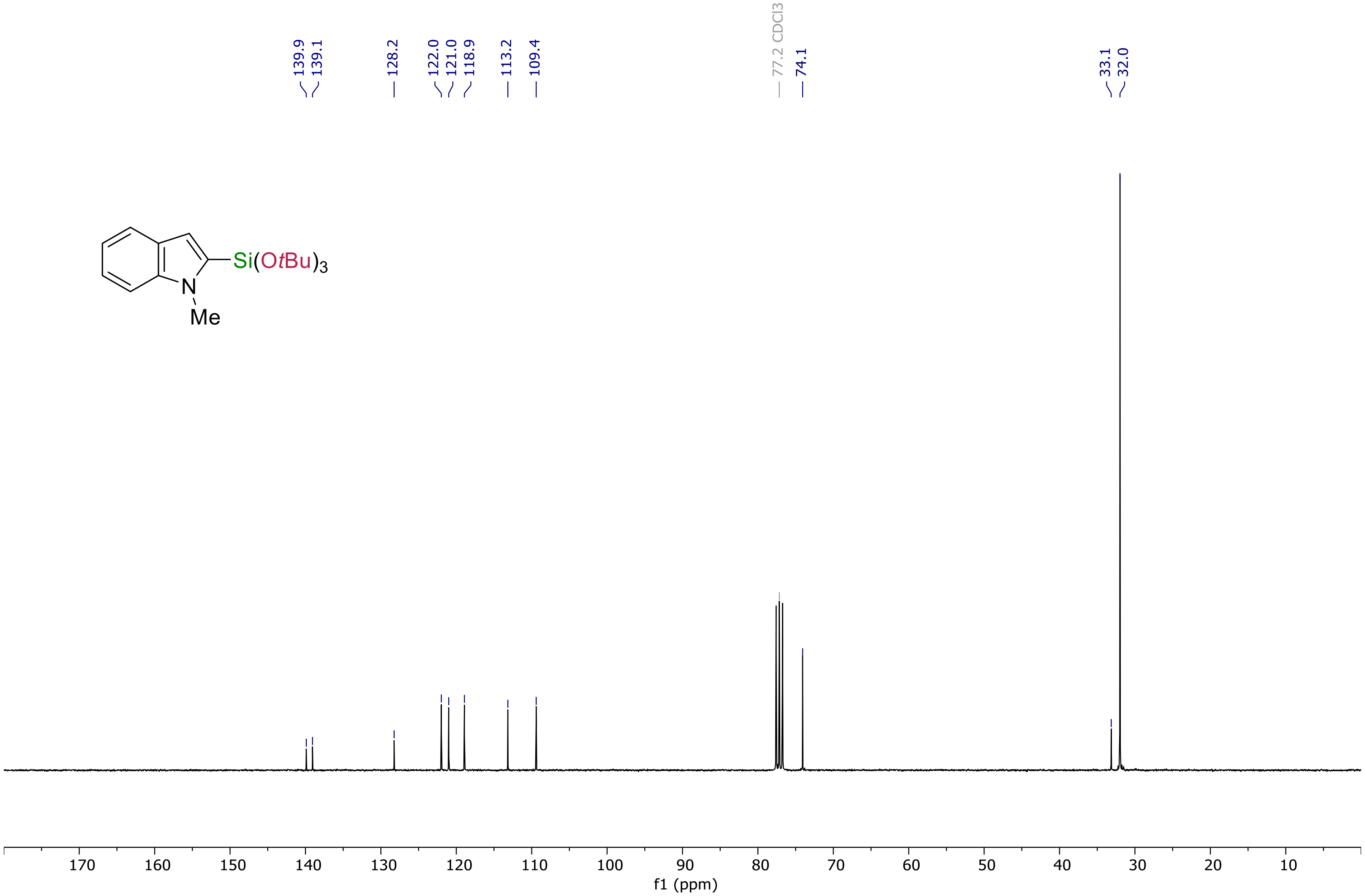

Figure S31: $^{13}C\{^{1}H\}$ NMR (75 MHz, $CDCl_3$) of compound **4e**.

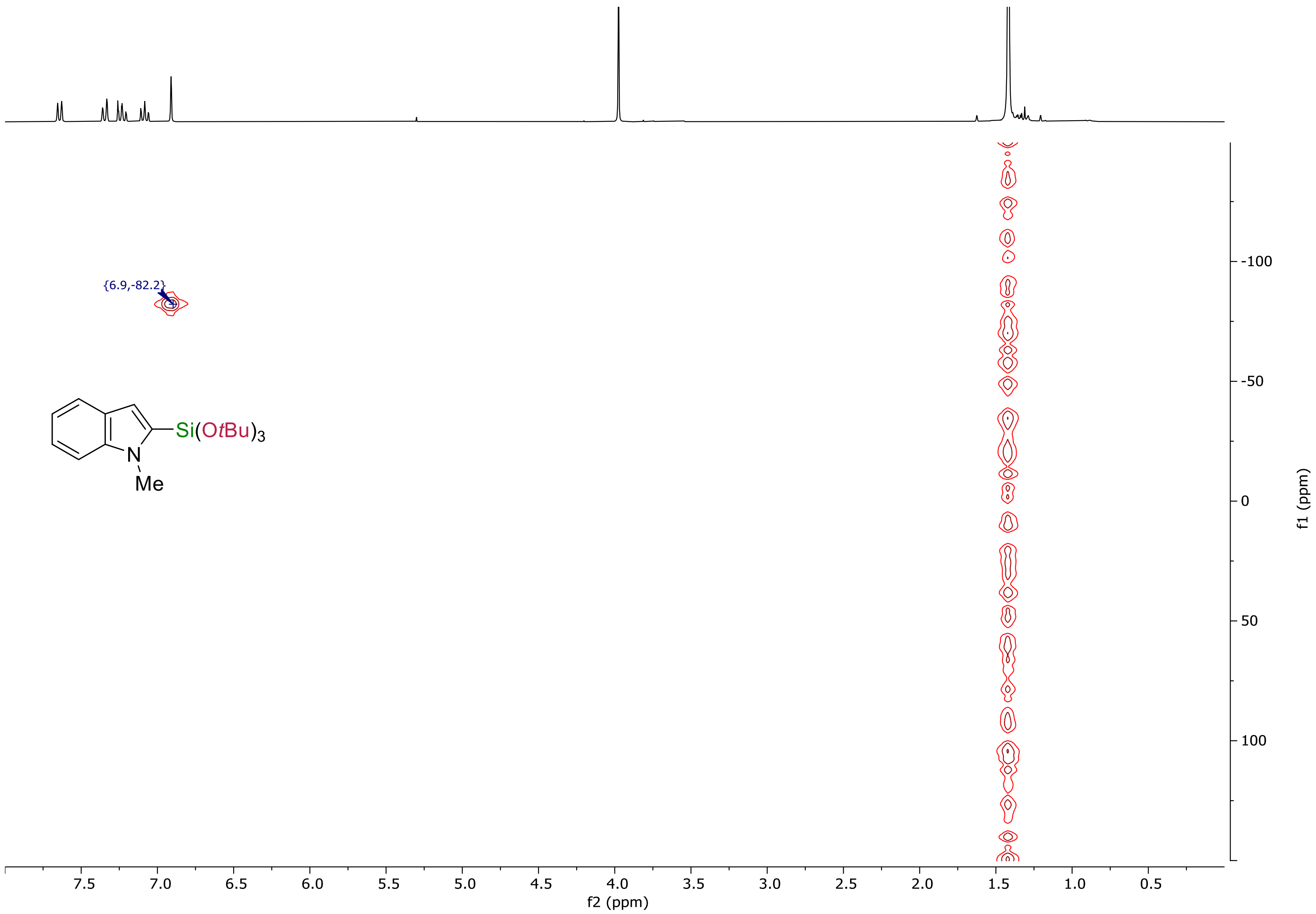

Figure S32: $^{1}H/^{29}Si$ HMQC NMR (300/60 MHz, $CDCl_3$) of compound **4e**.

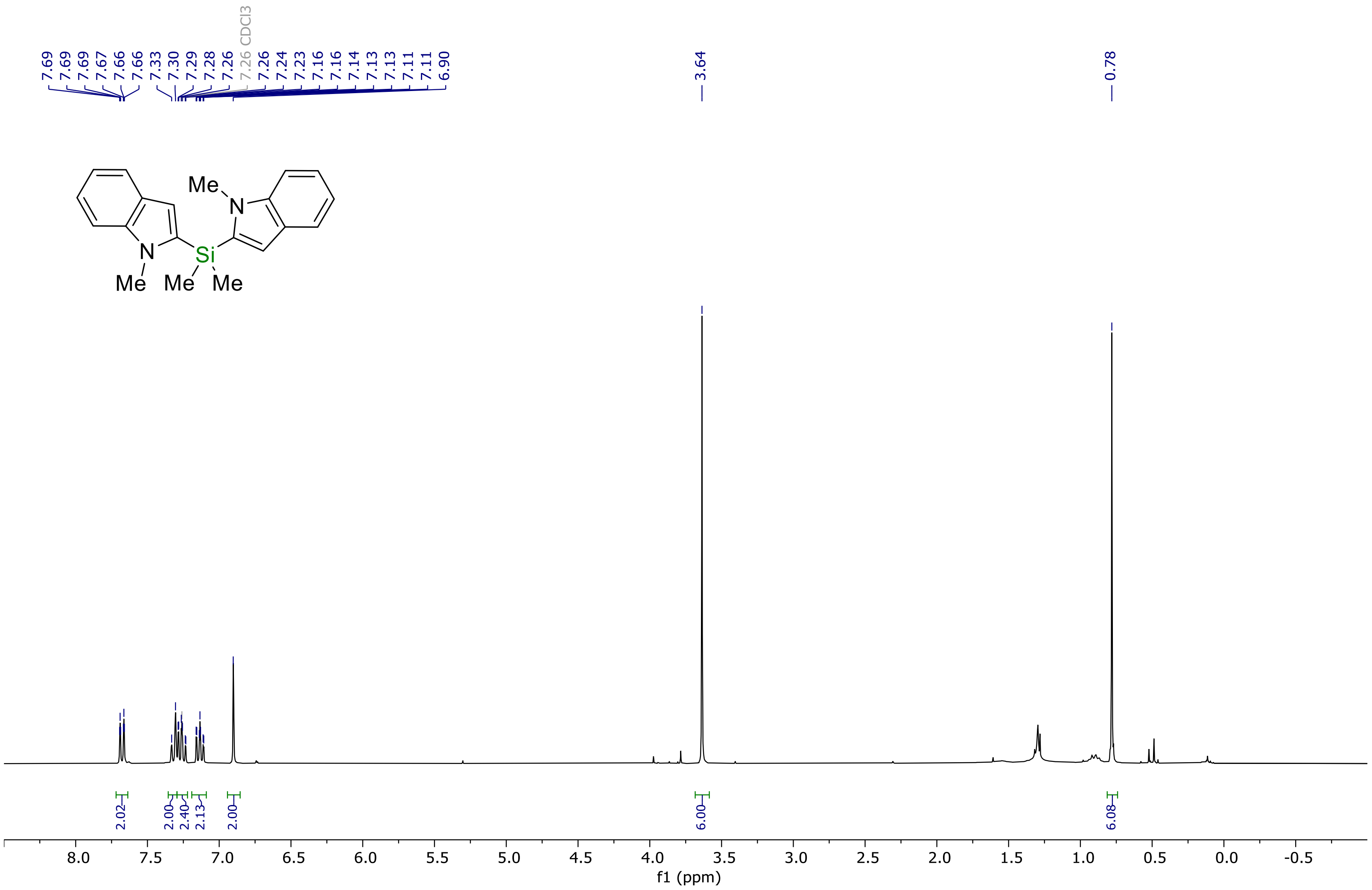

Figure S33: ^{1}H NMR (300 MHz, $CDCl_3$) of compound **5**.

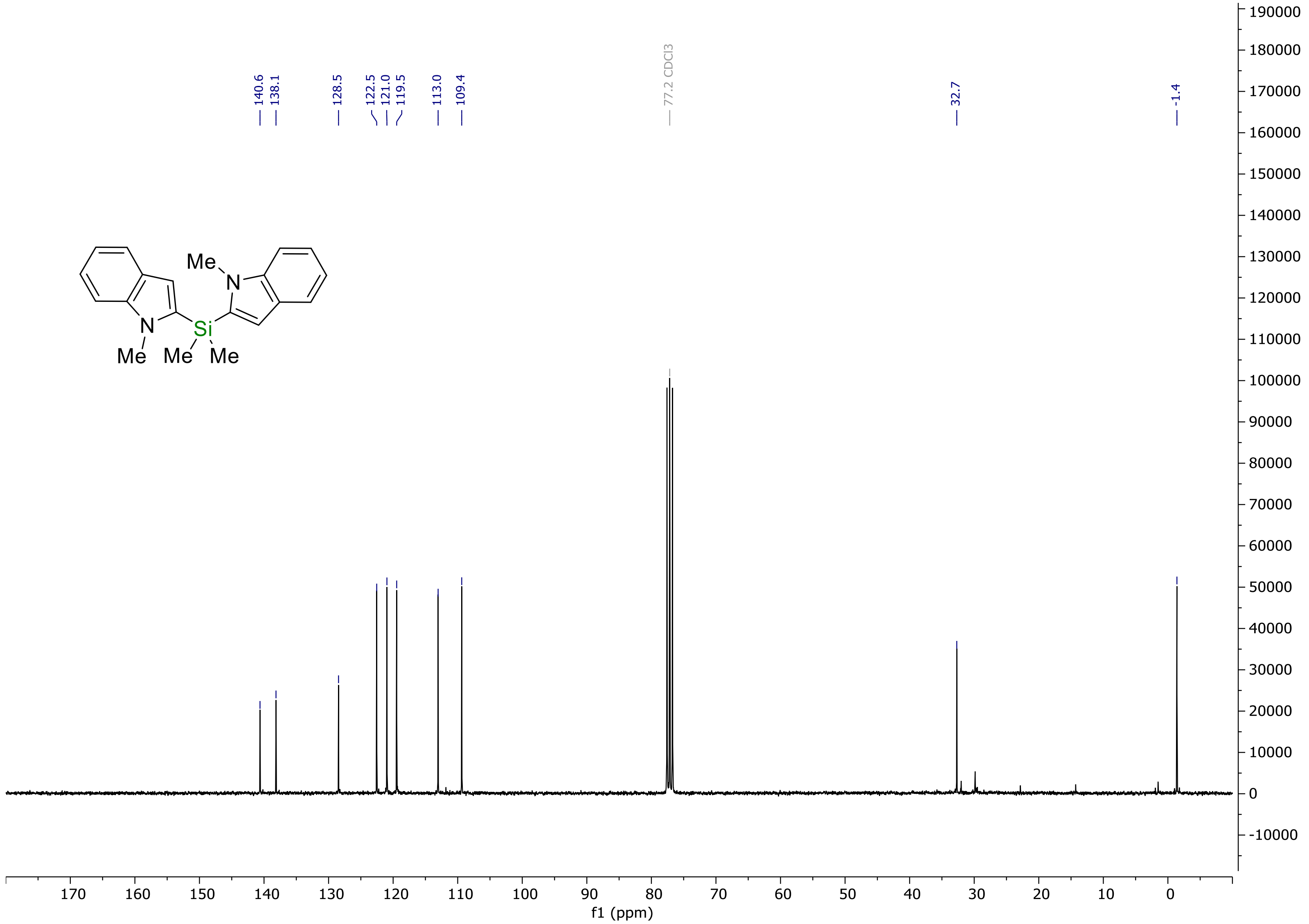

Figure S34: $^{13}C\{^{1}H\}$ NMR (75 MHz, $CDCl_3$) of compound **5**.

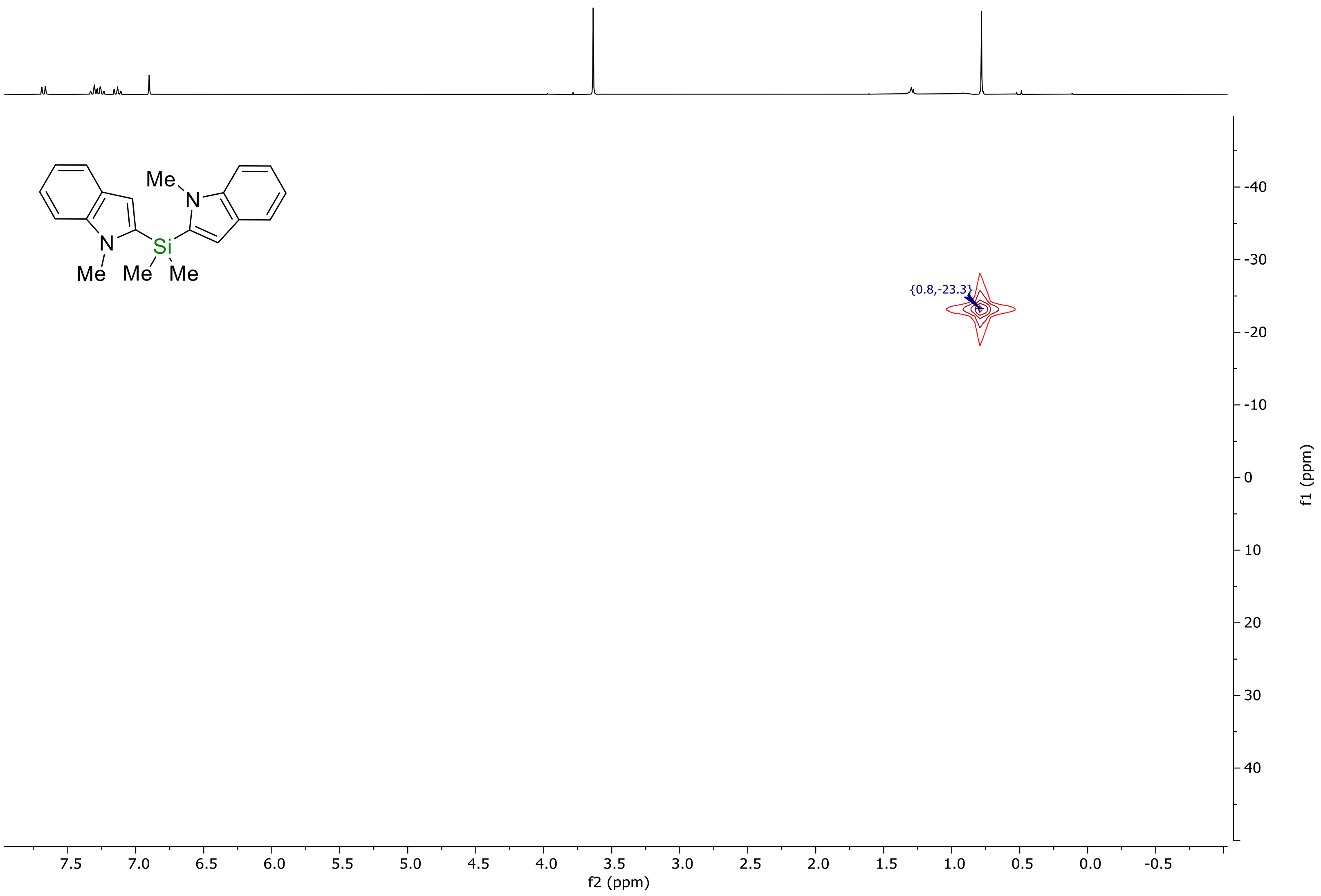

Figure S35: $^{1}H/^{29}Si$ HMQC NMR (300/60 MHz, $CDCl_3$) of compound **5**.

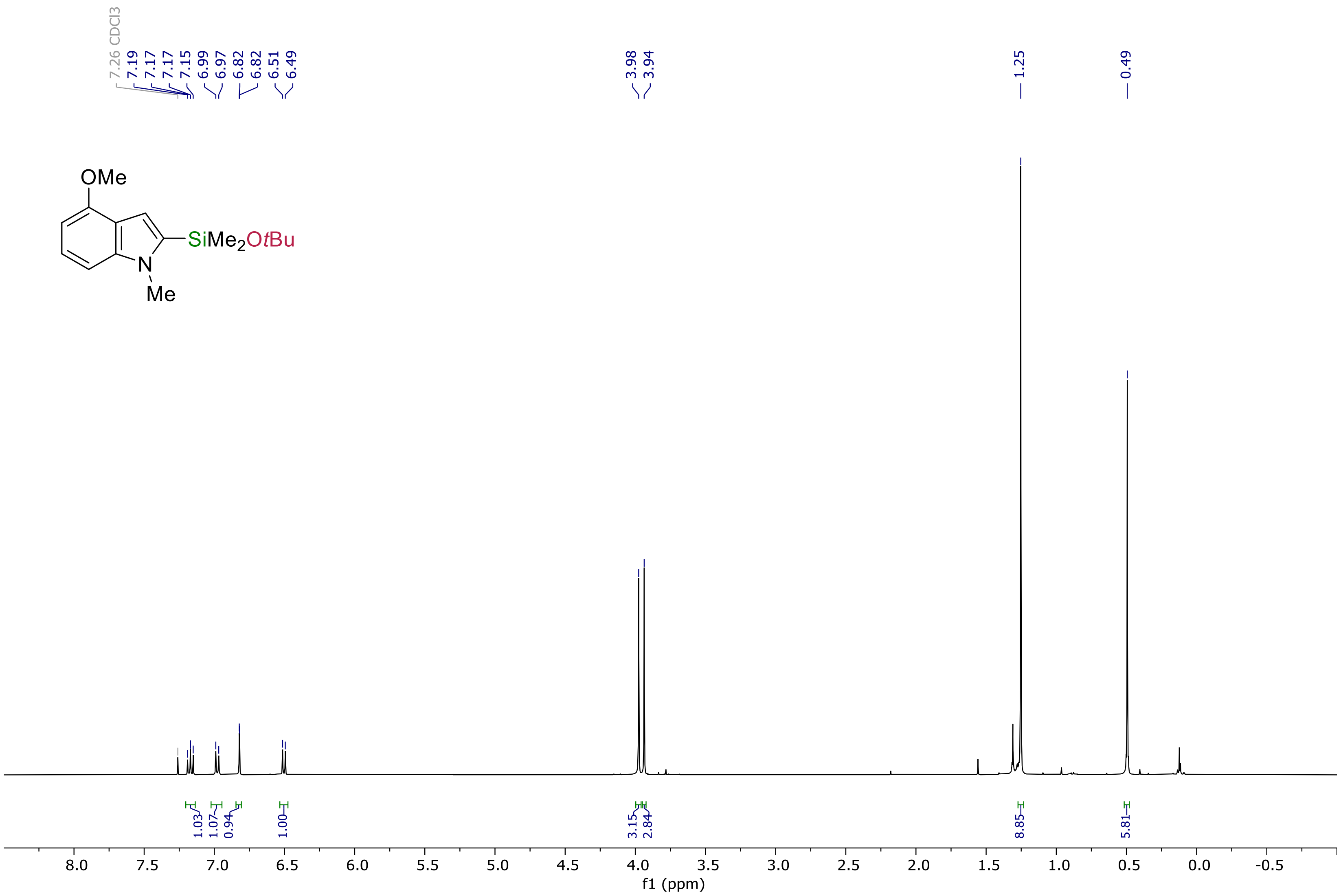

Figure S36: ^{1}H NMR (400 MHz, $CDCl_3$) of compound **6a**.

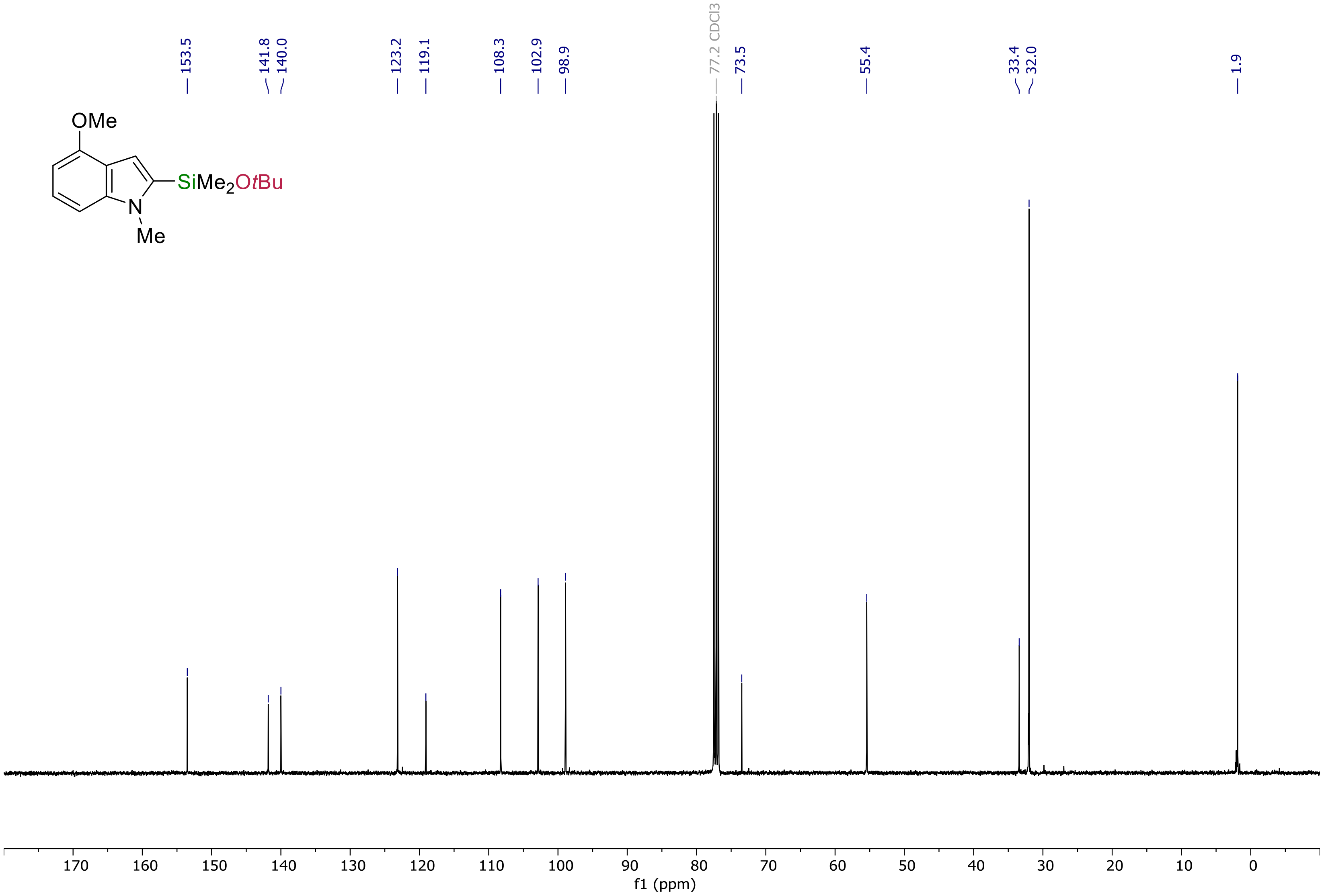

Figure S37: $^{13}C\{^{1}H\}$ NMR (101 MHz, $CDCl_3$) of compound **6a**.

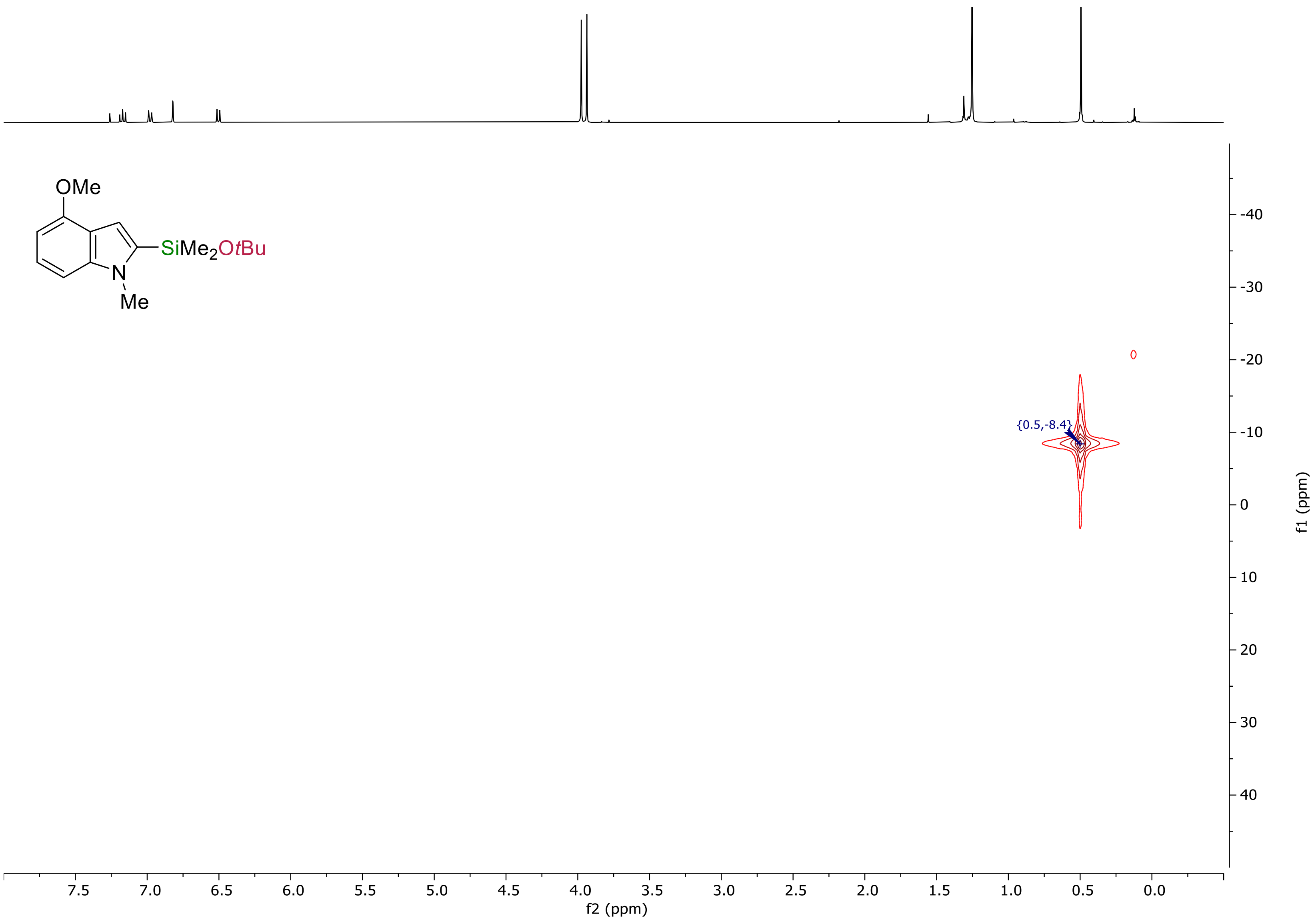

Figure S38: $^{1}H/^{29}Si$ HMQC NMR (400/75 MHz, $CDCl_3$) of compound **6a**.

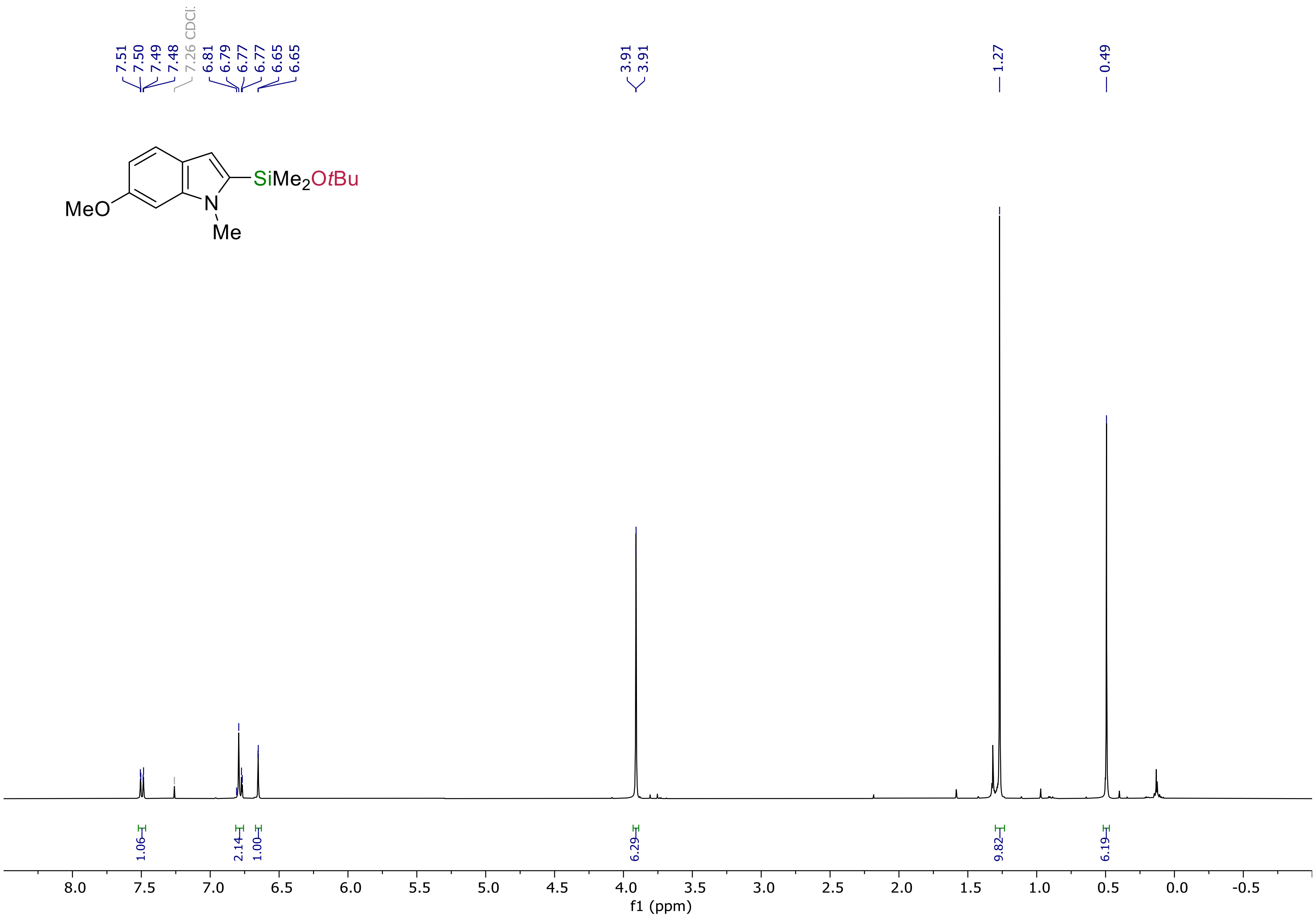

Figure S39: ^{1}H NMR (400 MHz, $CDCl_3$) of compound **7a**.

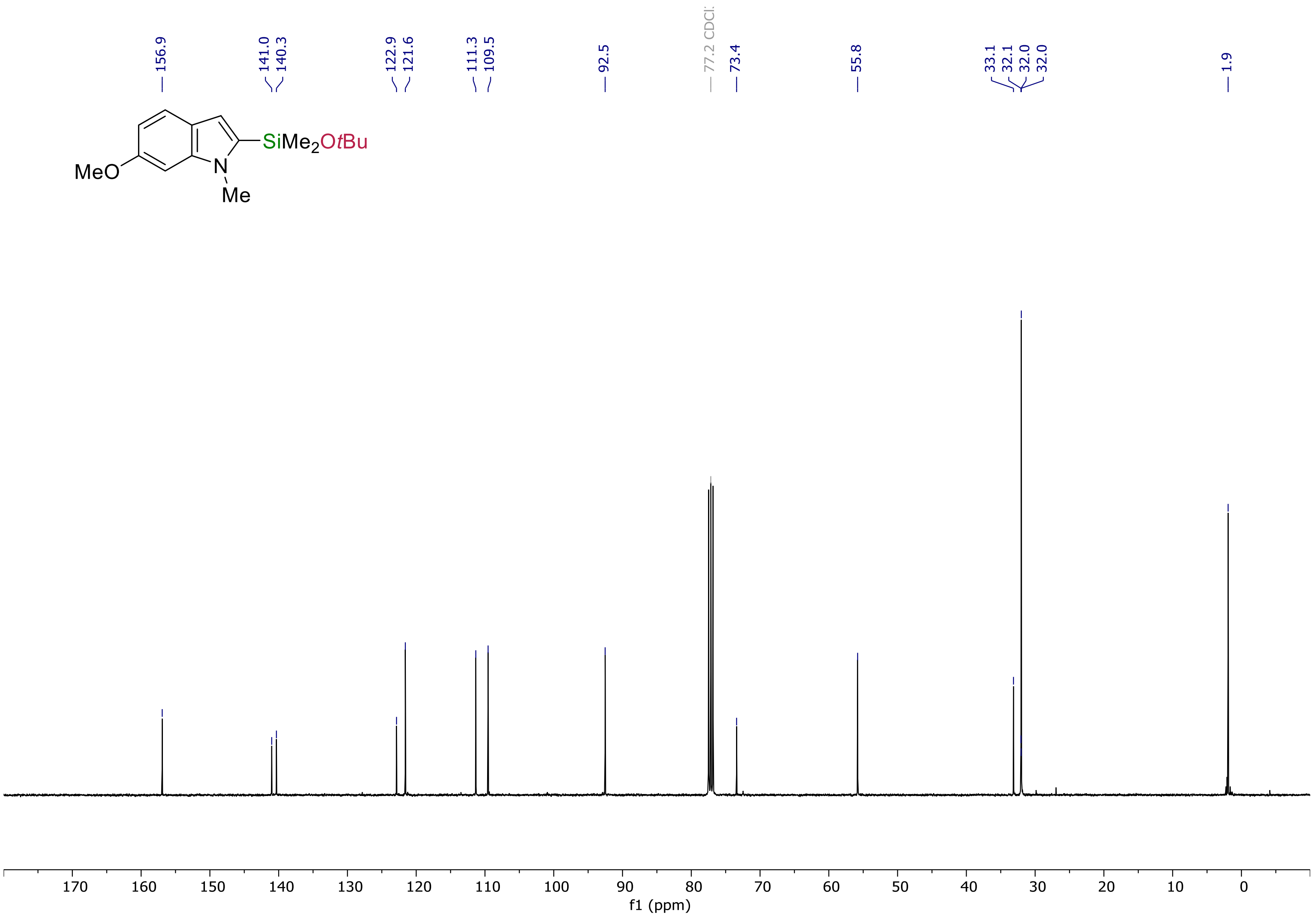

Figure S40: $^{13}C\{^1H\}$ NMR (101 MHz, $CDCl_3$) of compound **7a**.

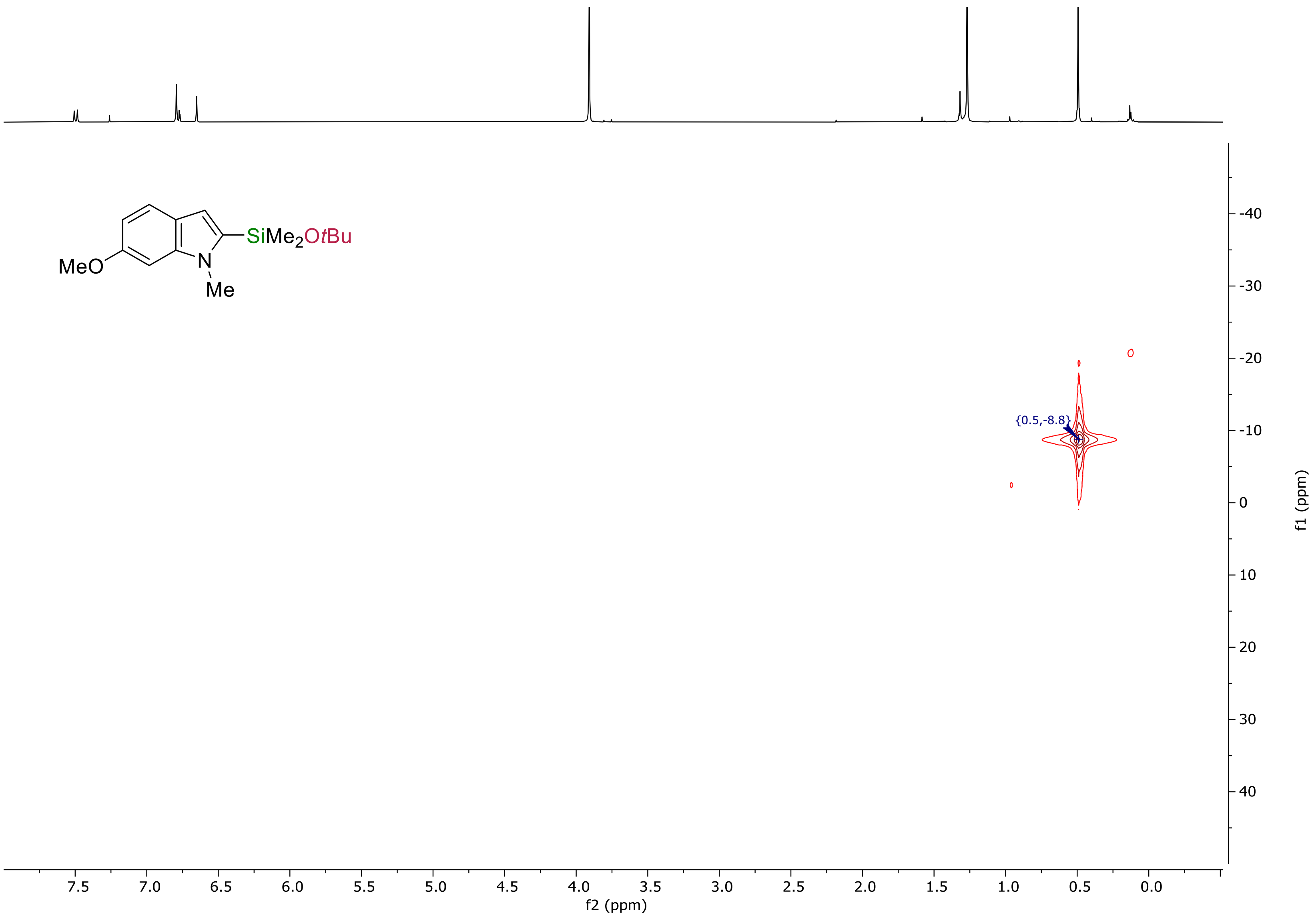

Figure S41: $^{1}H/^{29}Si$ HMQC NMR (400/75 MHz, $CDCl_3$) of compound **7a**.

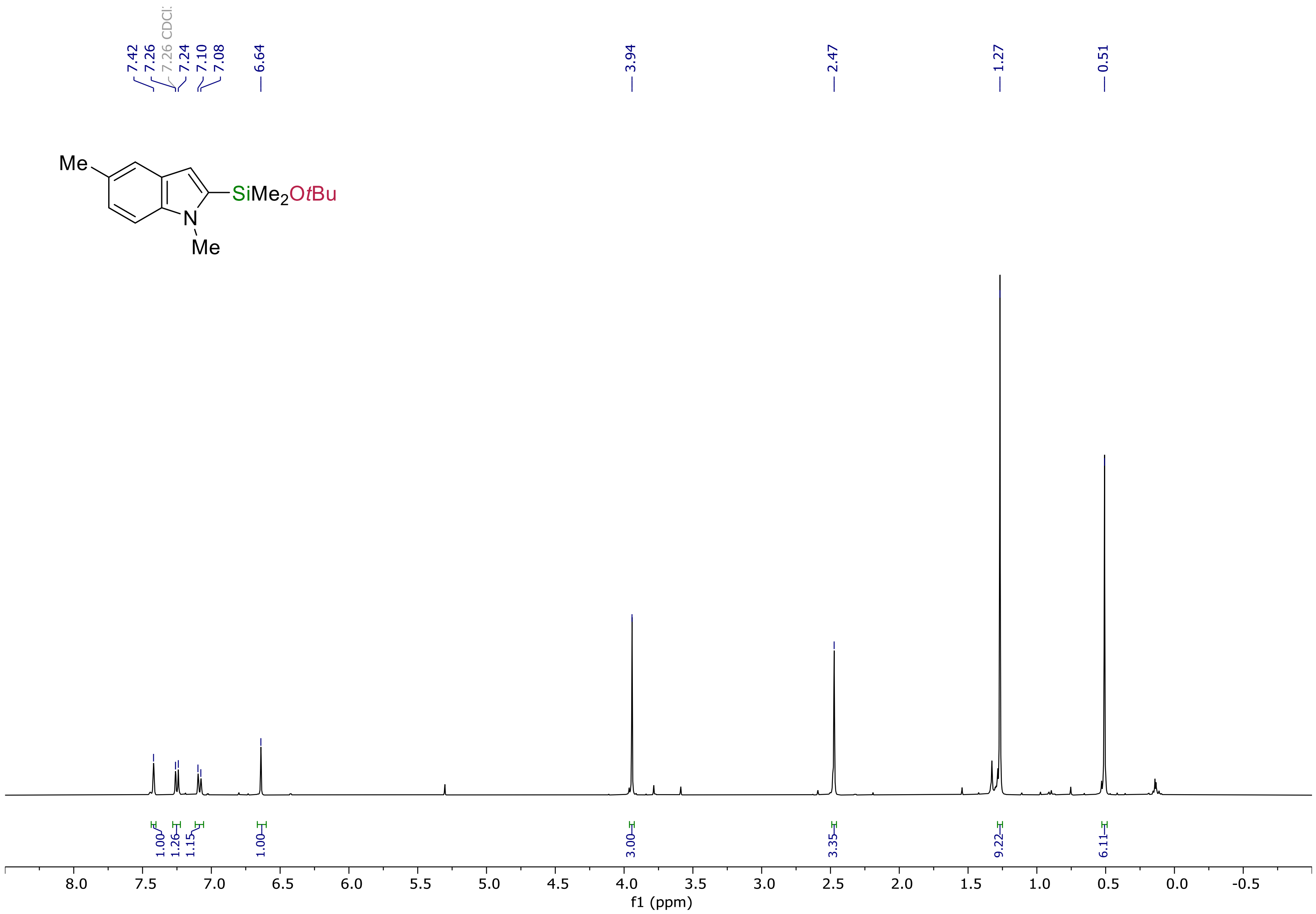

Figure S42: ^{1}H NMR (400 MHz, $CDCl_3$) of compound **8a**.

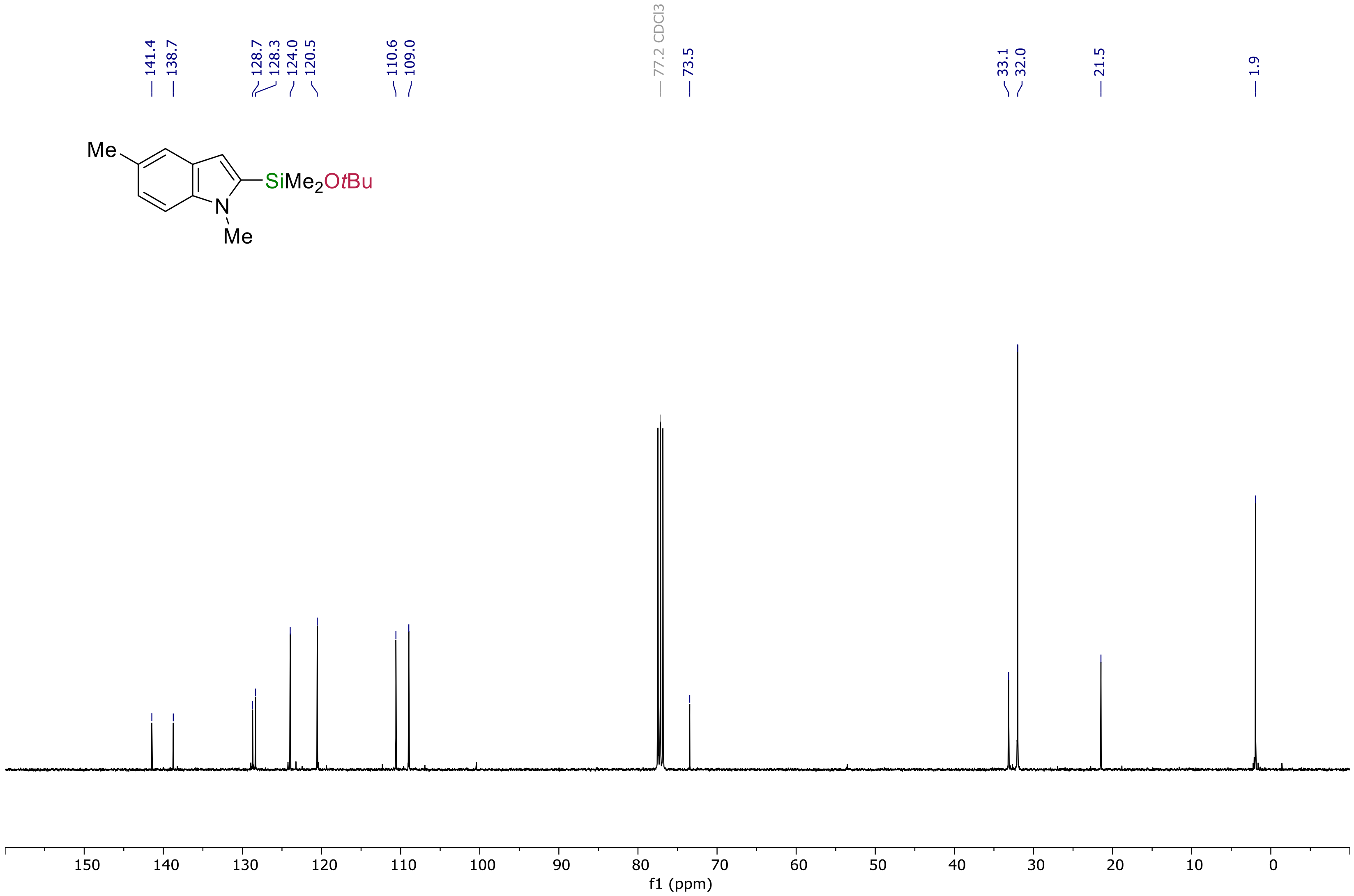

Figure S43: $^{13}C\{^1H\}$ NMR (101 MHz, $CDCl_3$) of compound **8a**.

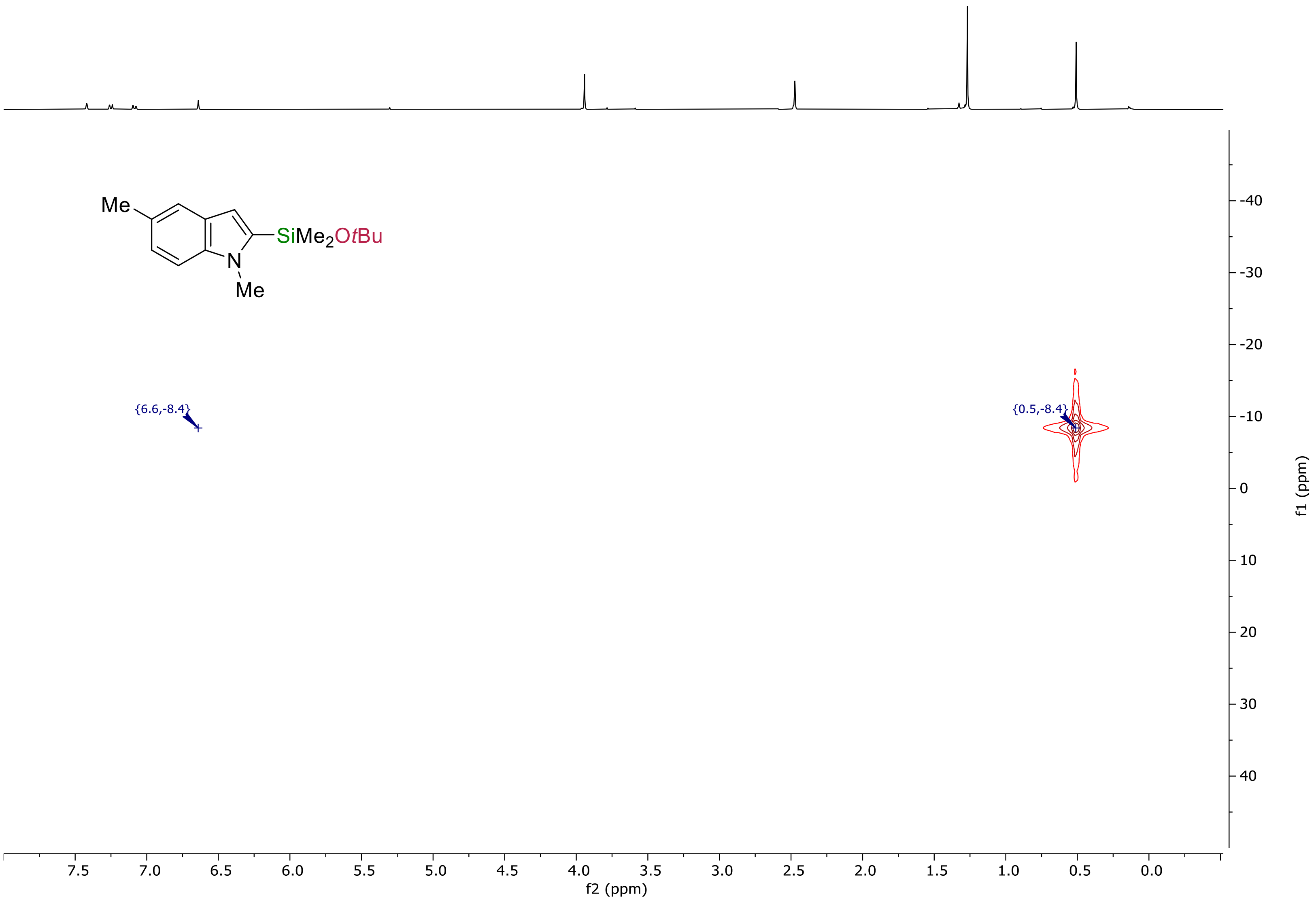

Figure S44: $^{1}H/^{29}Si$ HMQC NMR (400/75 MHz, $CDCl_3$) of compound **8a**.

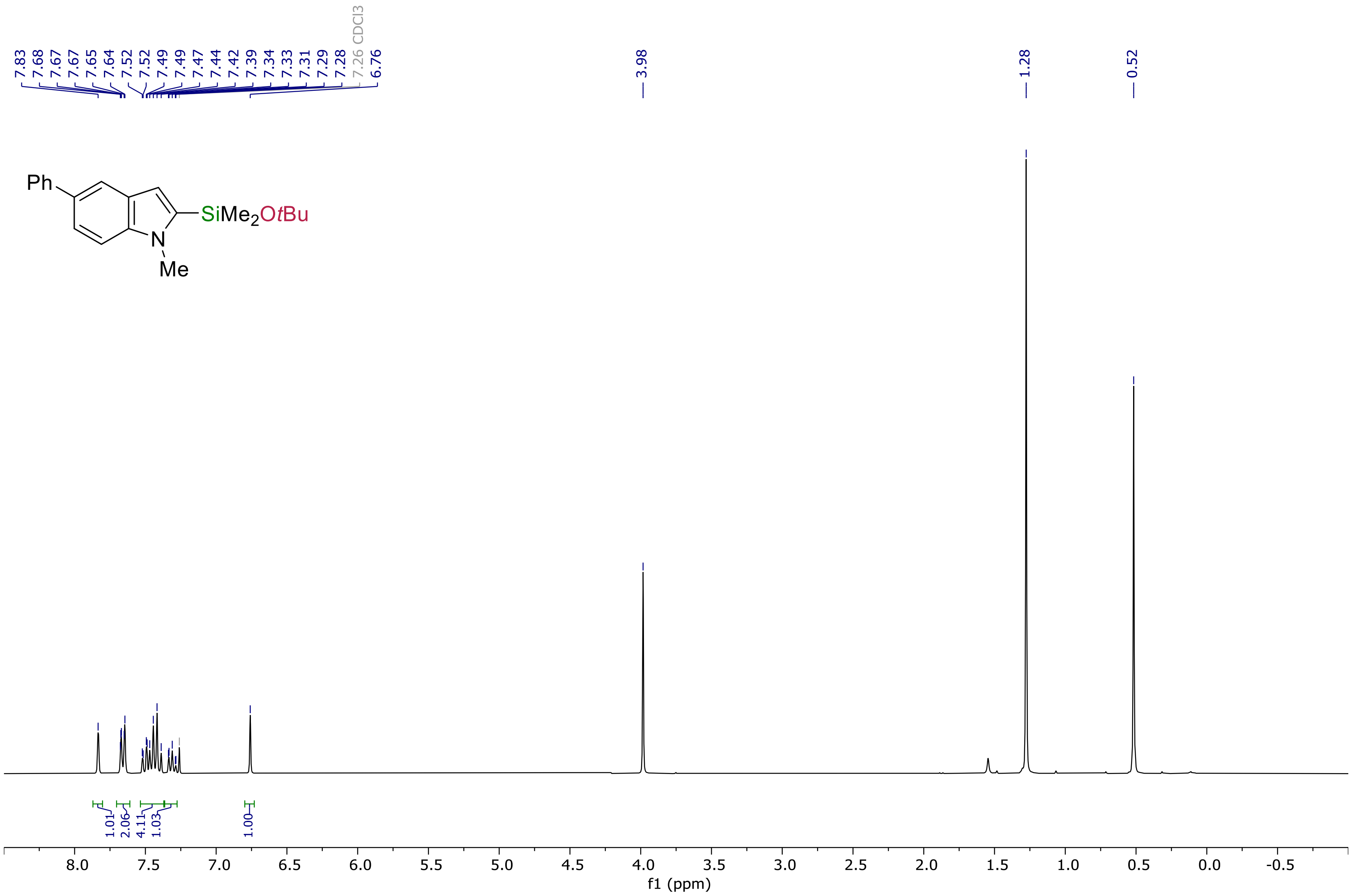

Figure S45: ^{1}H NMR (300 MHz, $CDCl_3$) of compound **9a**.

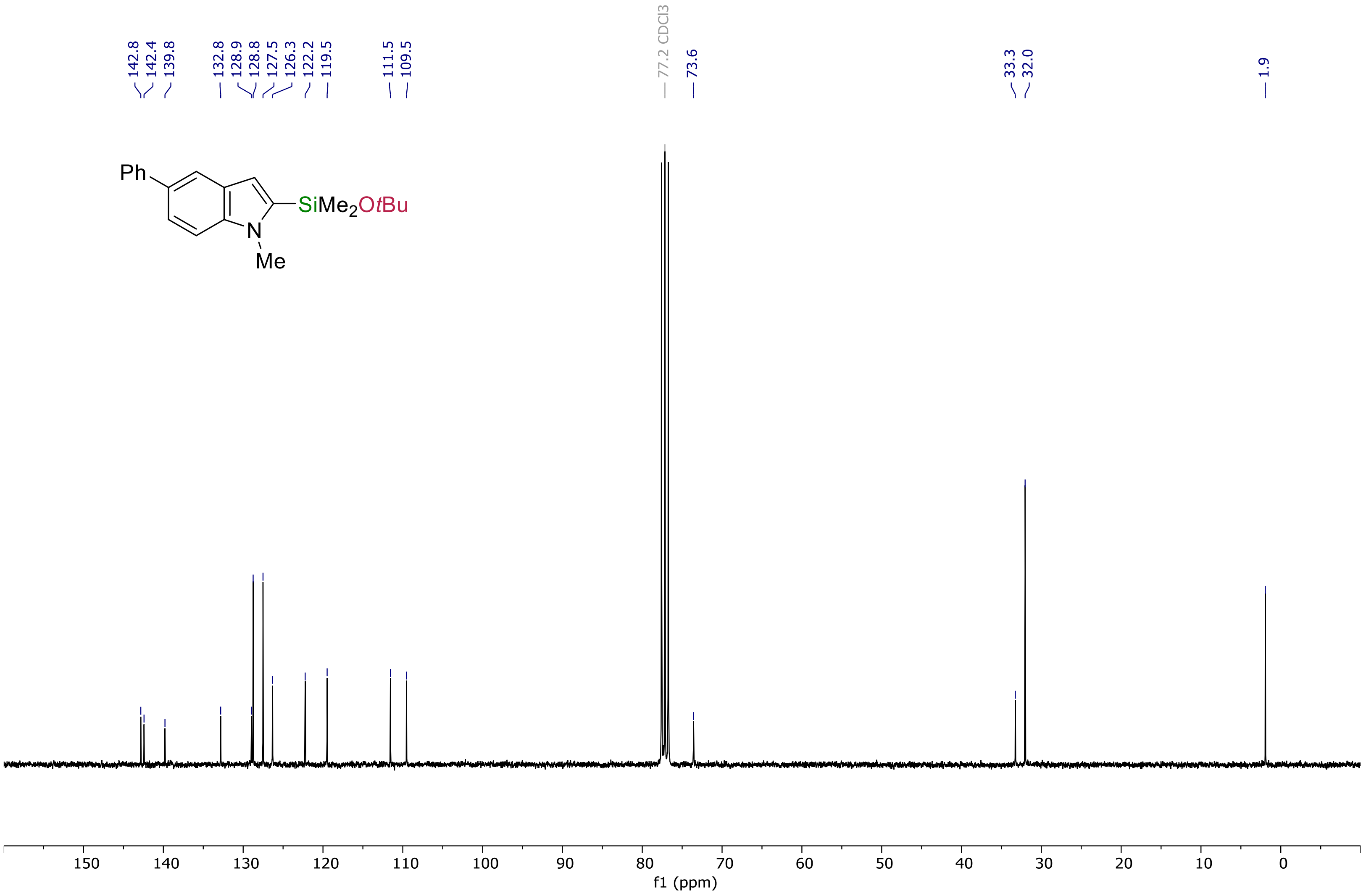

Figure S46: $^{13}C\{^1H\}$ NMR (75 MHz, $CDCl_3$) of compound **9a**.

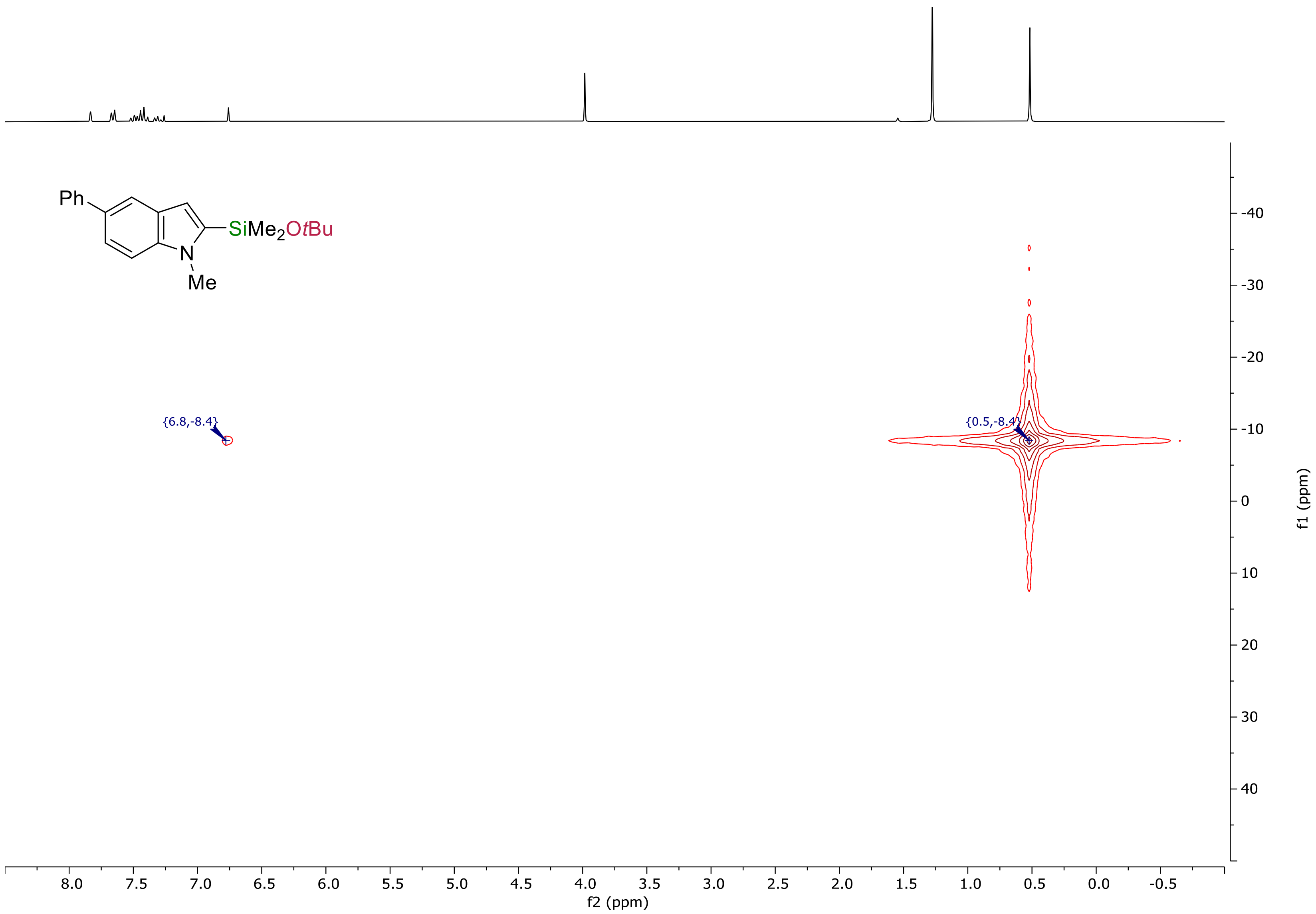

Figure S47: $^{1}H/^{29}Si$ HMQC NMR (300/60 MHz, $CDCl_3$) of compound **9a**.

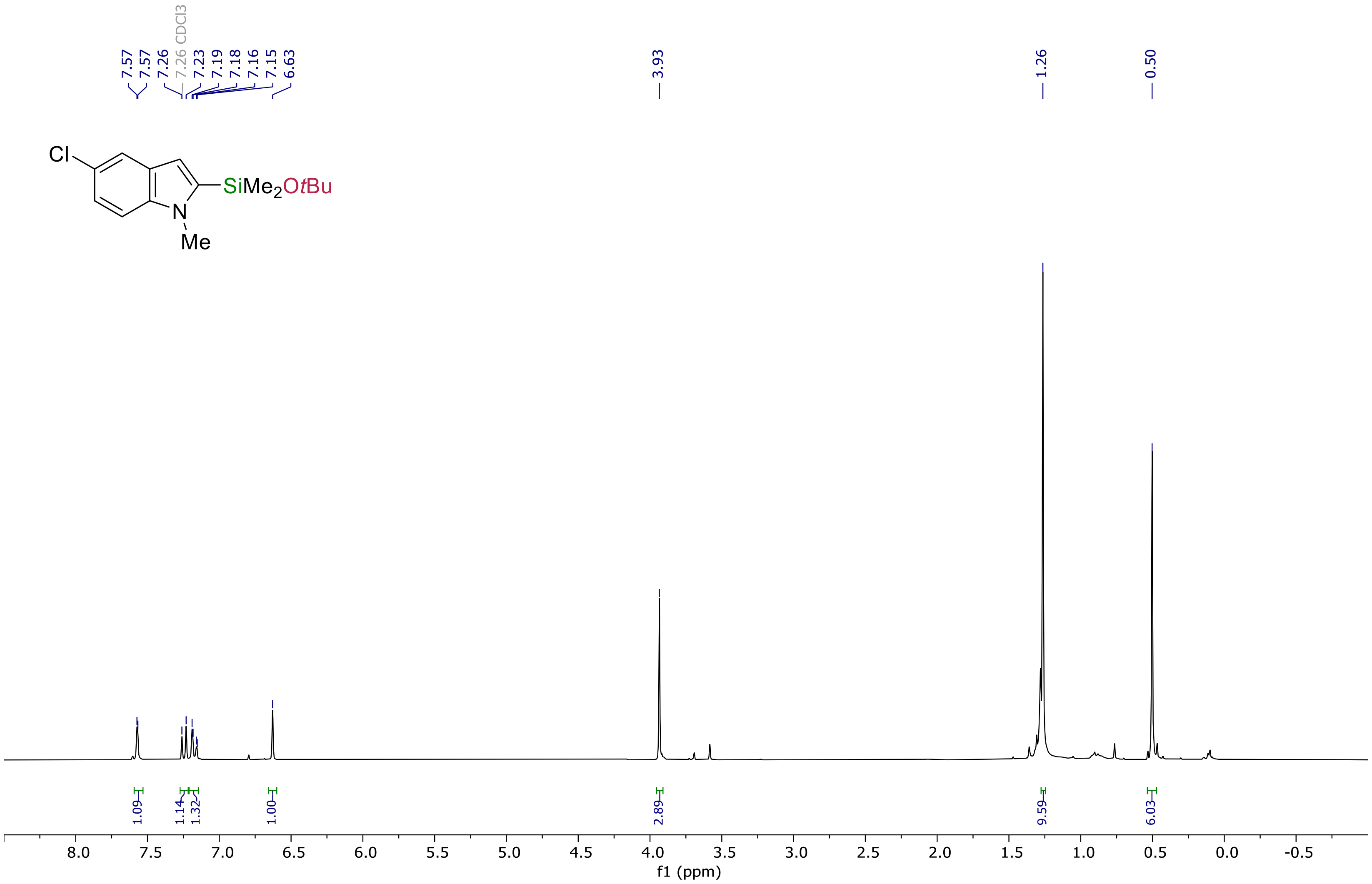

Figure S48: ^{1}H NMR (300 MHz, $CDCl_3$) of compound **10a.**

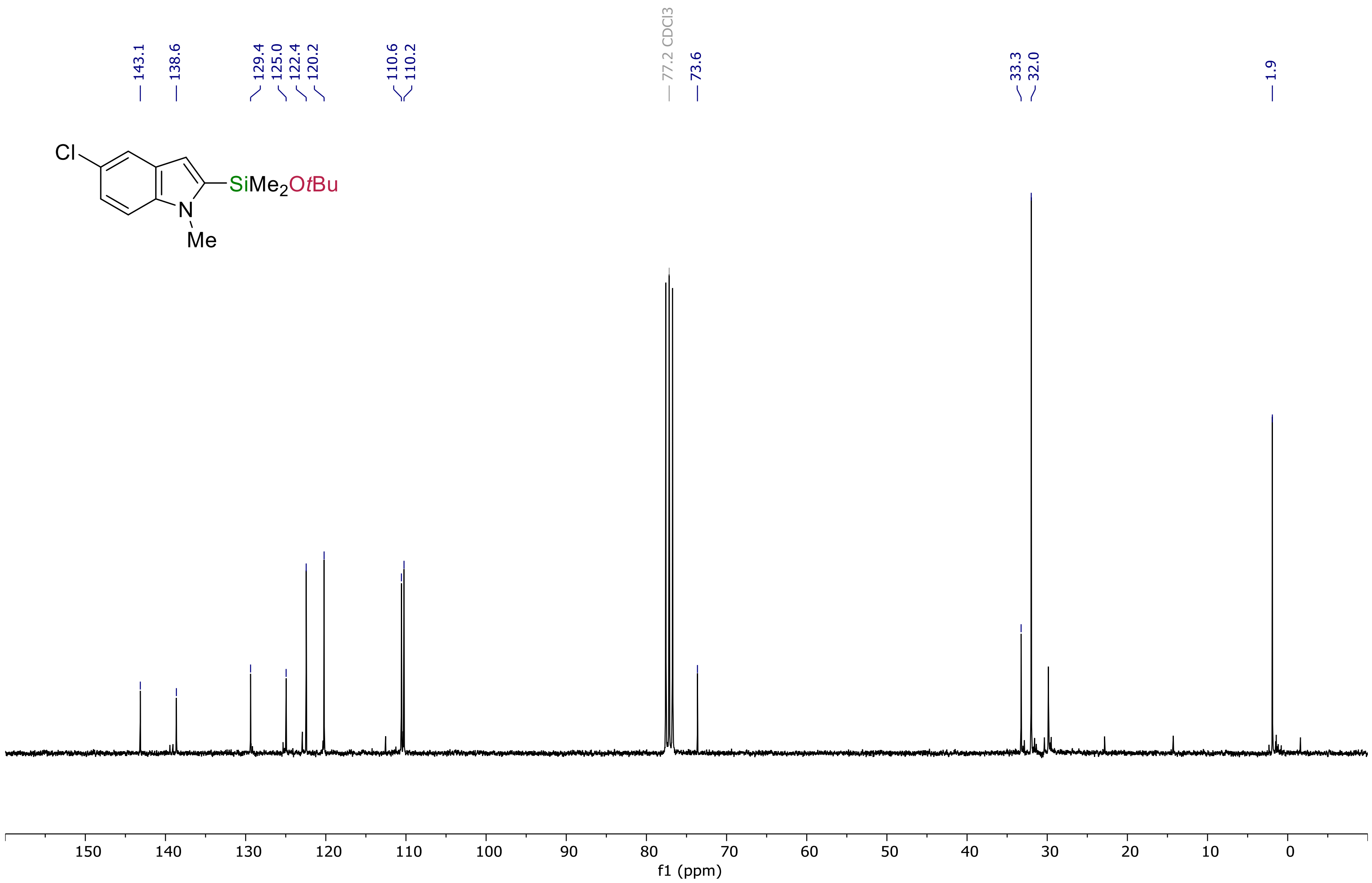

Figure S49: $^{13}C\{^{1}H\}$ NMR (75 MHz, $CDCl_3$) of compound **10a.**

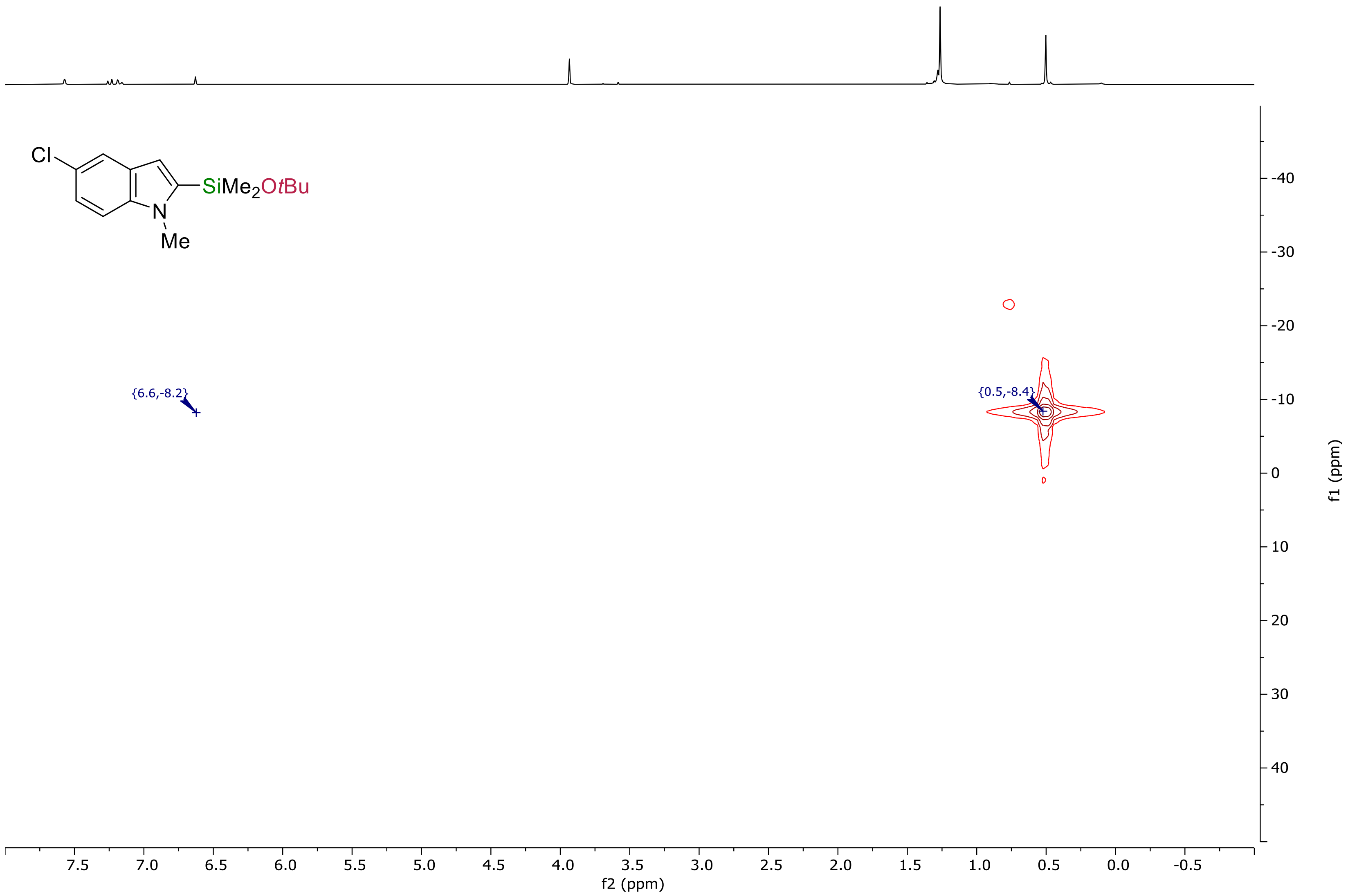

Figure S50: $^{1}H/^{29}Si$ HMQC NMR (300/60 MHz, $CDCl_3$) of compound **10a.**

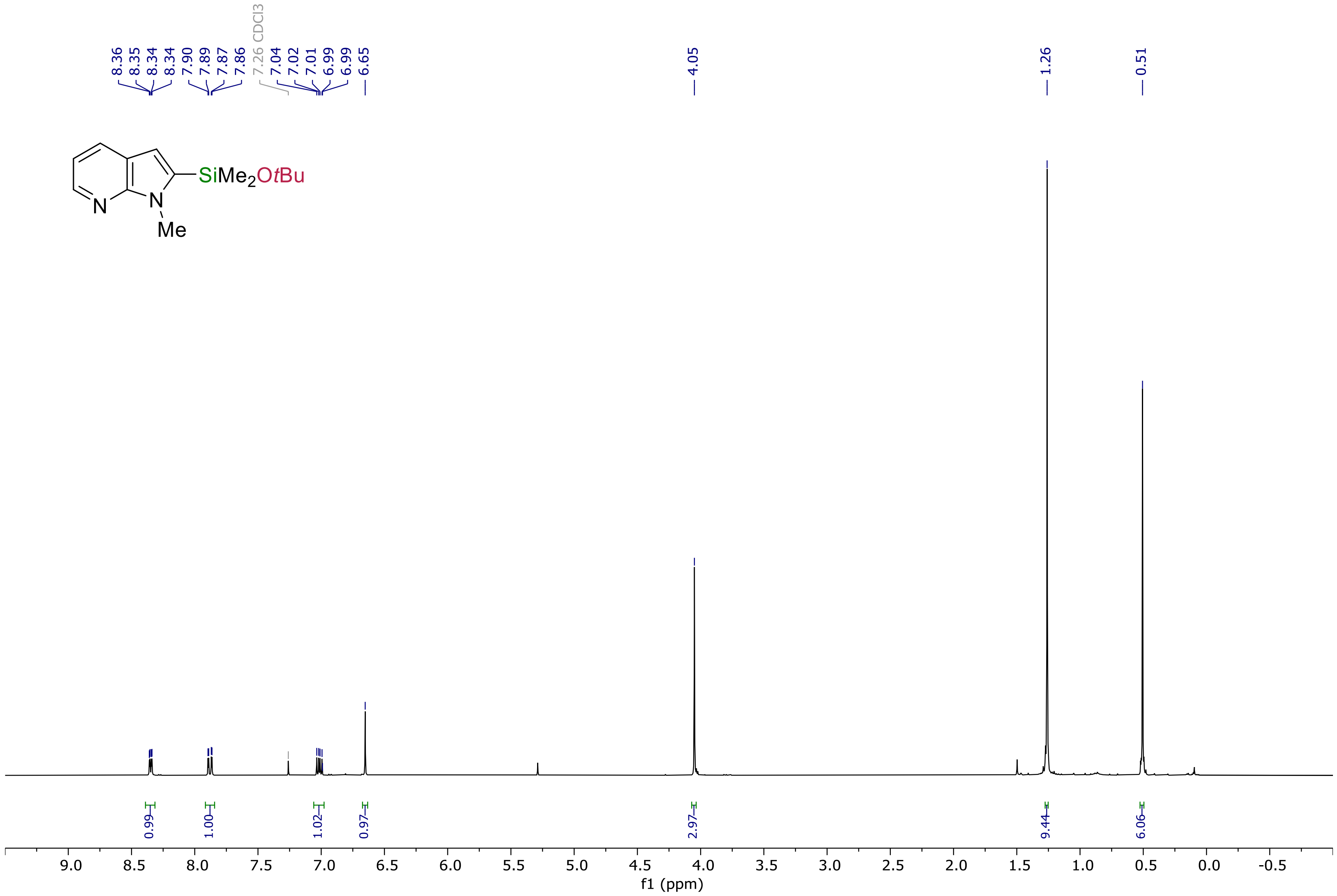

Figure S51: ^{1}H NMR (300 MHz, $CDCl_3$) of compound **11a.**

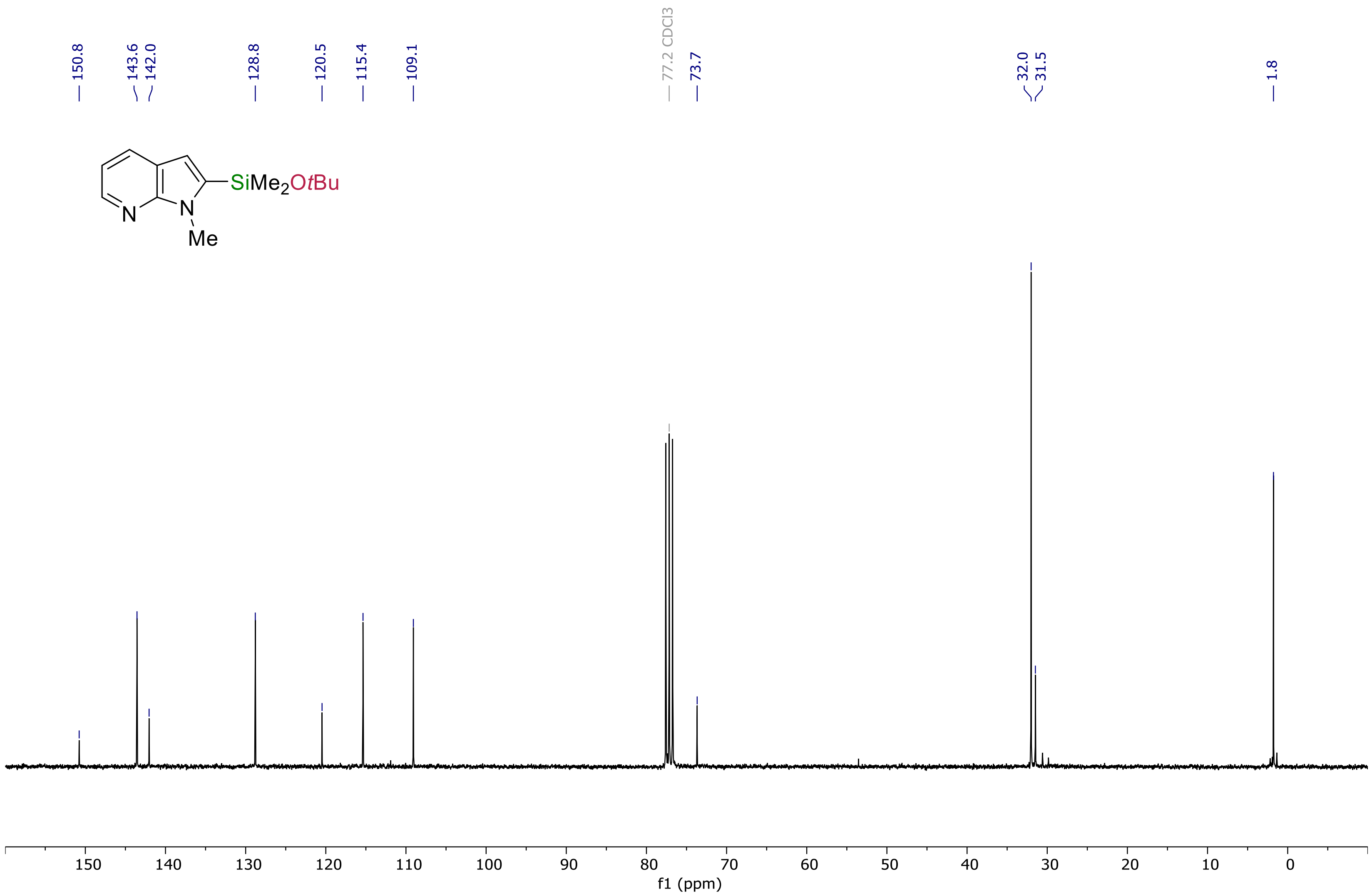

Figure S52: $^{13}C\{^{1}H\}$ NMR (75 MHz, $CDCl_3$) of compound **11a.**

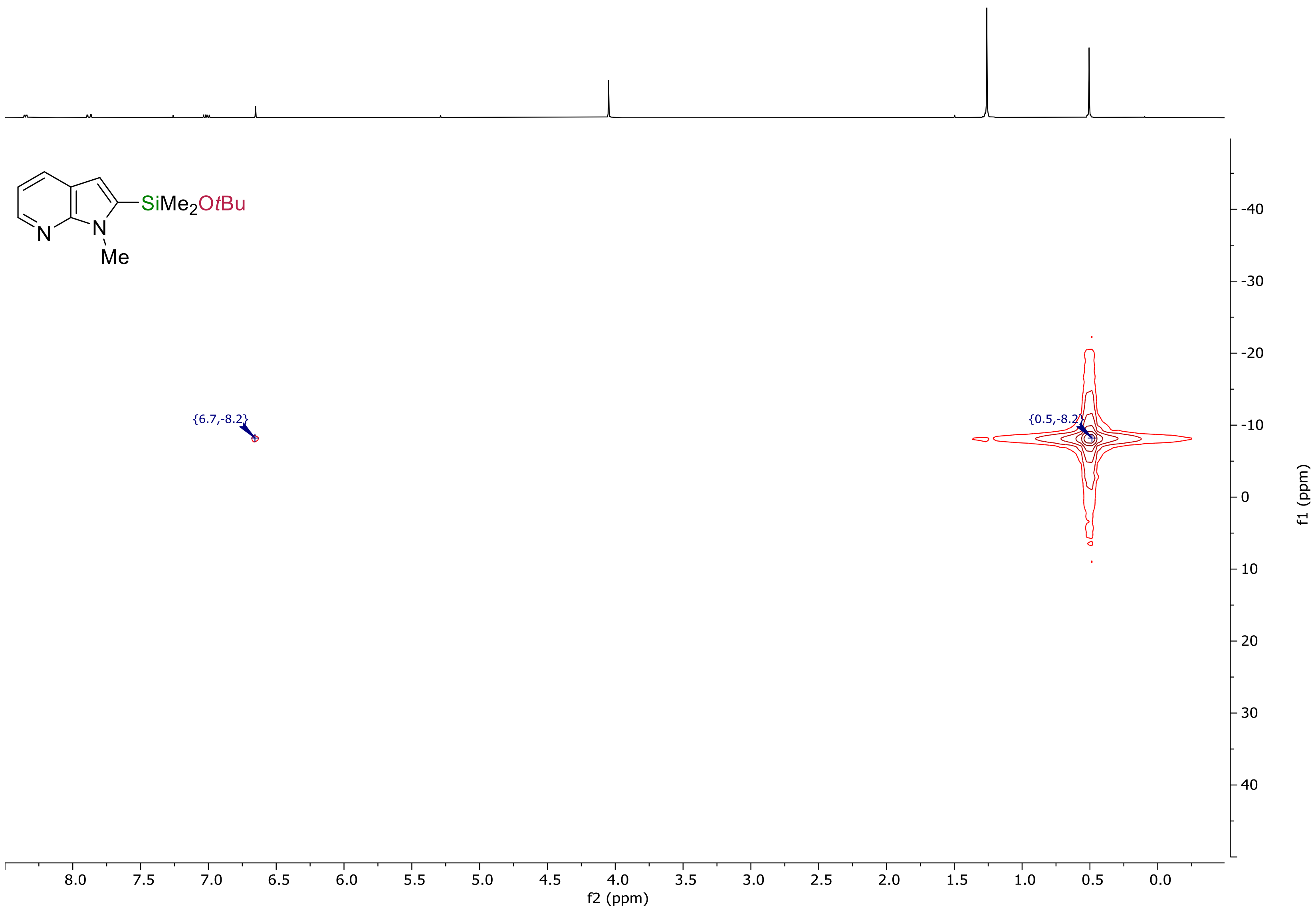

Figure S53: $^{1}H/^{29}Si$ HMQC NMR (300/60 MHz, $CDCl_3$) of compound **11a.**

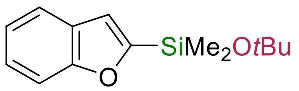

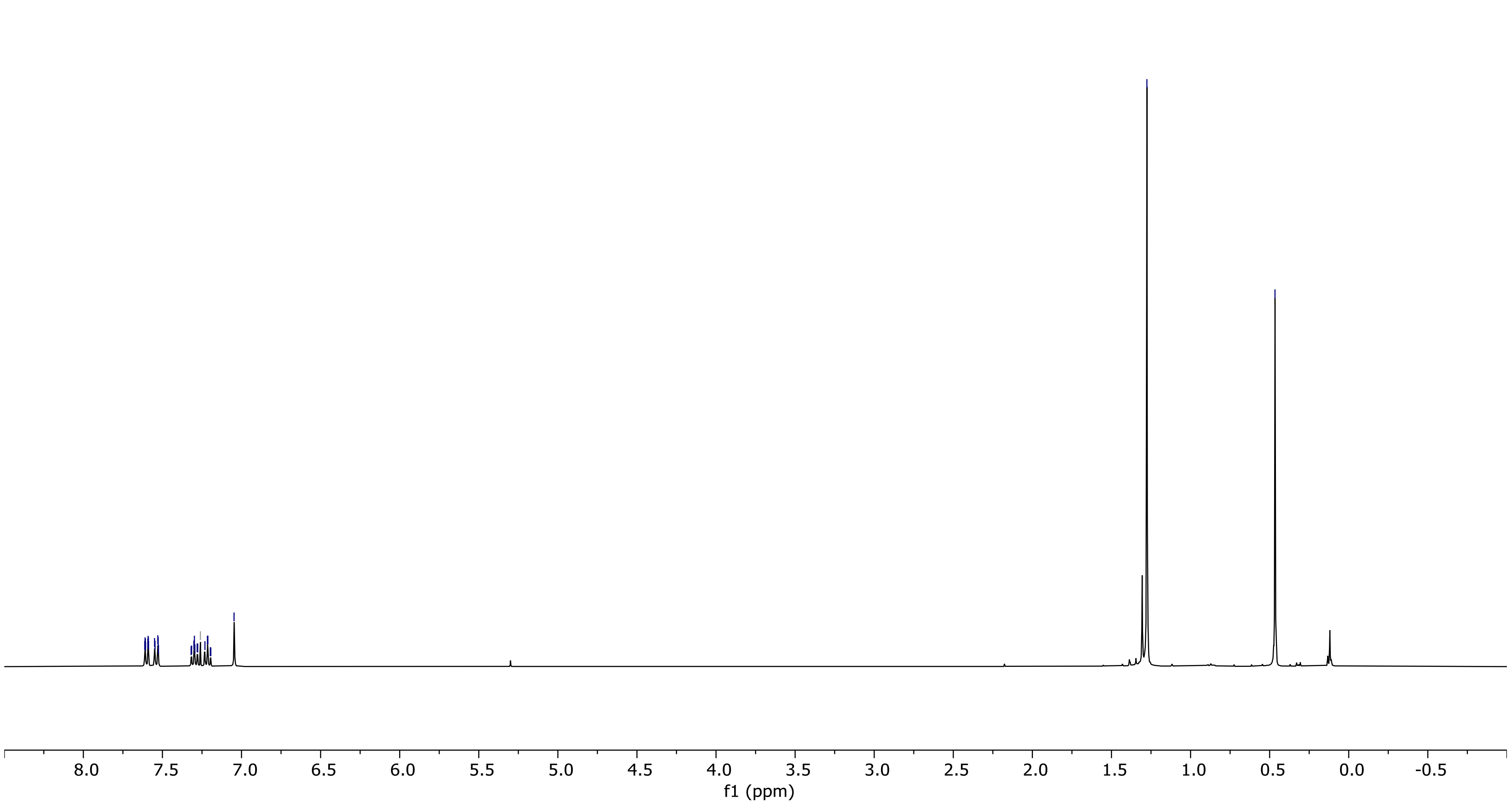

Figure S54: ^{1}H NMR (400 MHz, $CDCl_3$) of compound **12a.**

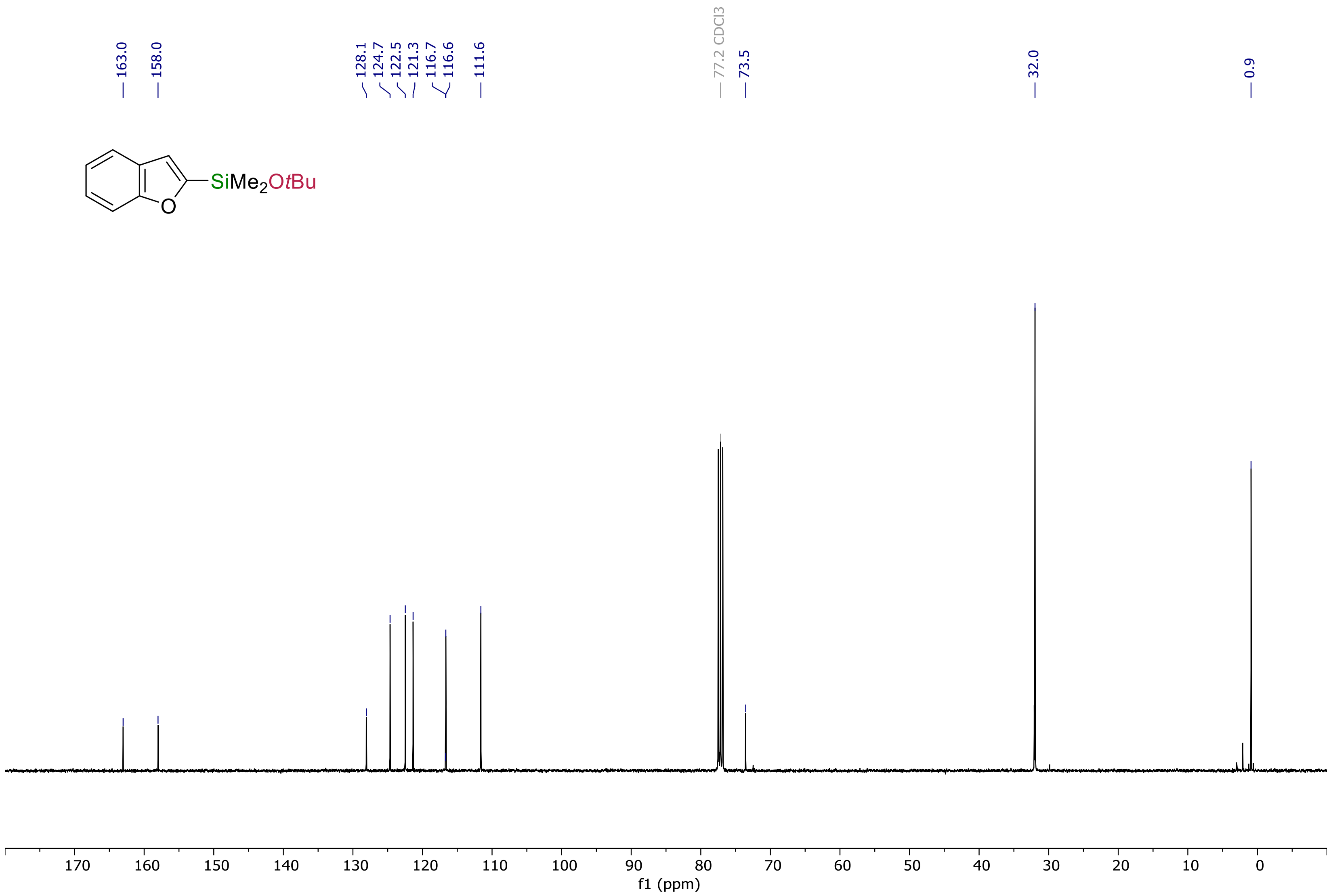

Figure S55: $^{13}C\{^{1}H\}$ NMR (101 MHz, $CDCl_3$) of compound **12a.**

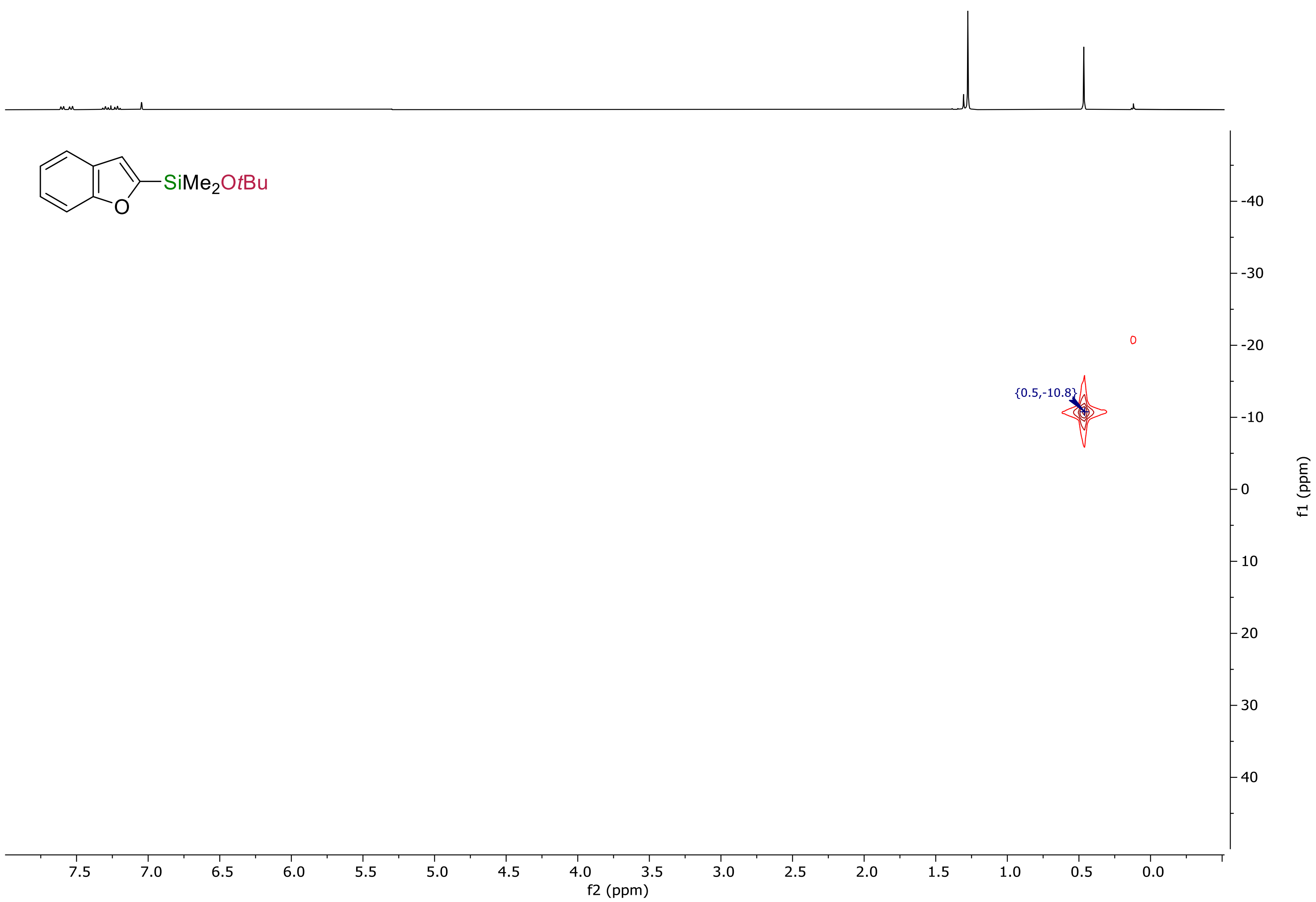

Figure S56: $^{1}H/^{29}Si$ HMQC NMR (400/75 MHz, $CDCl_3$) of compound **12a.**

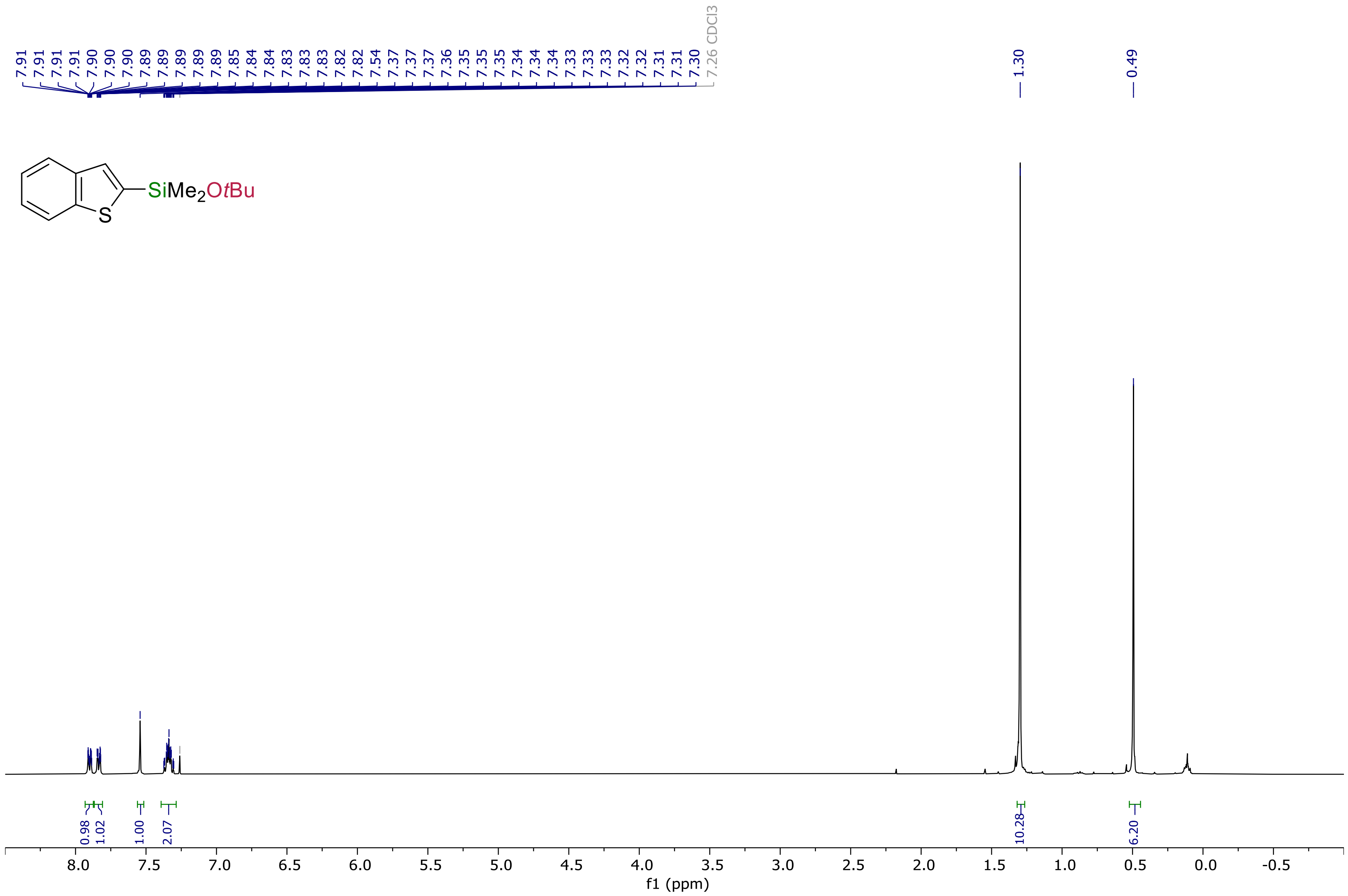

Figure S57: ^{1}H NMR (400 MHz, $CDCl_3$) of compound **13a.**

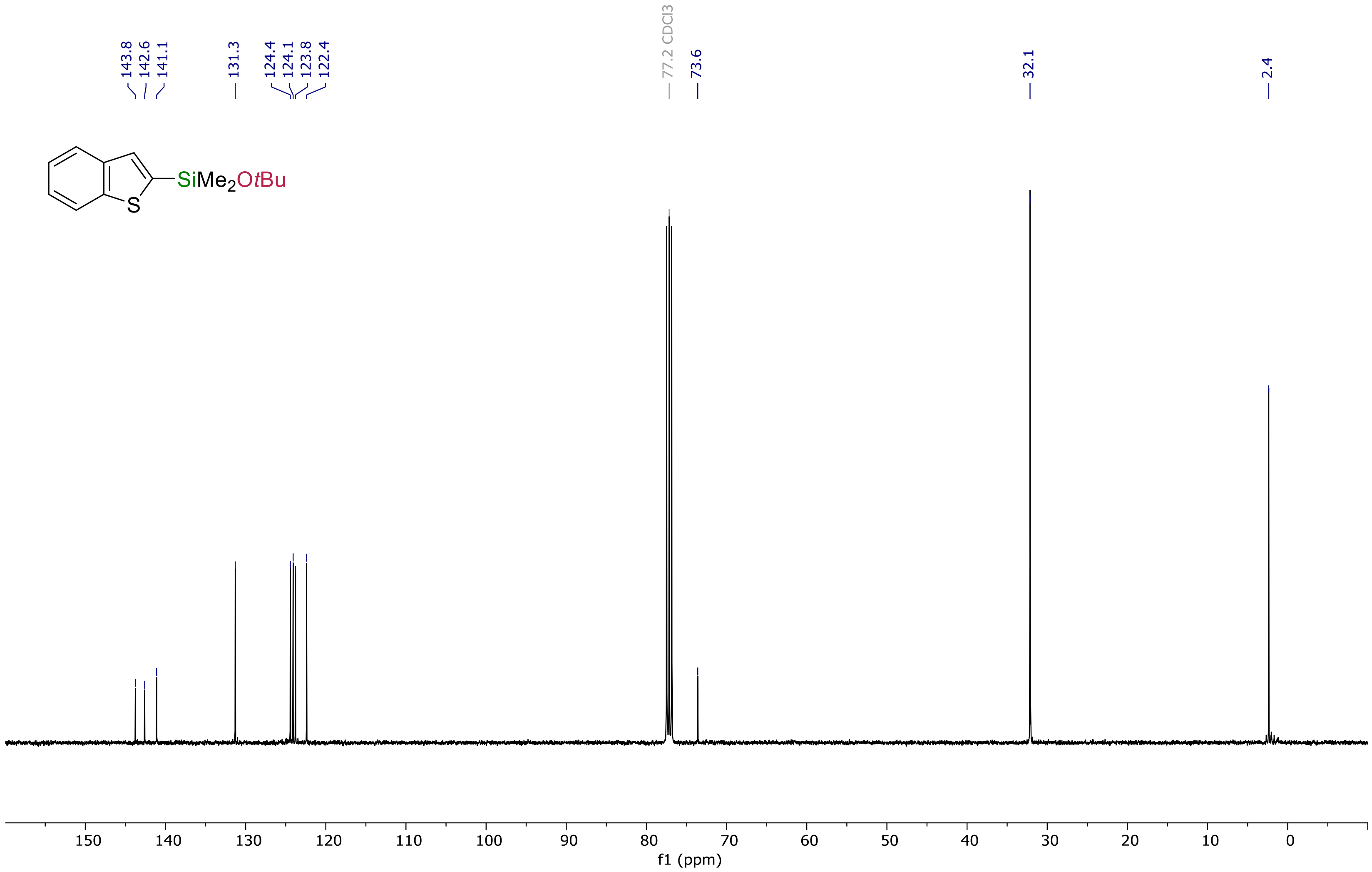

Figure S58: $^{13}C\{^{1}H\}$ NMR (101 MHz, $CDCl_3$) of compound **13a.**

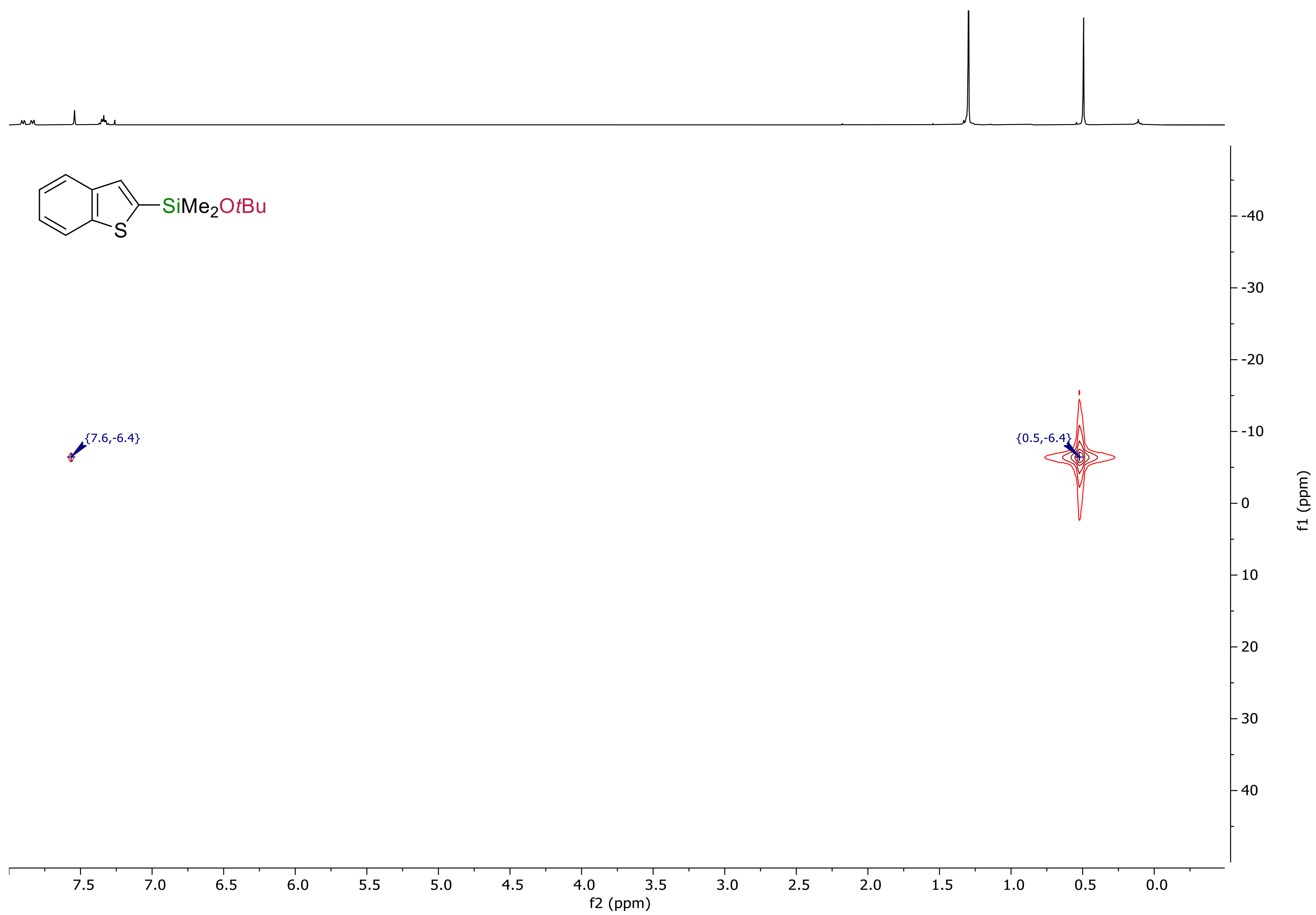

Figure S59: $^{1}H/^{29}Si$ HMQC NMR (400/75 MHz, $CDCl_3$) of compound **13a.**

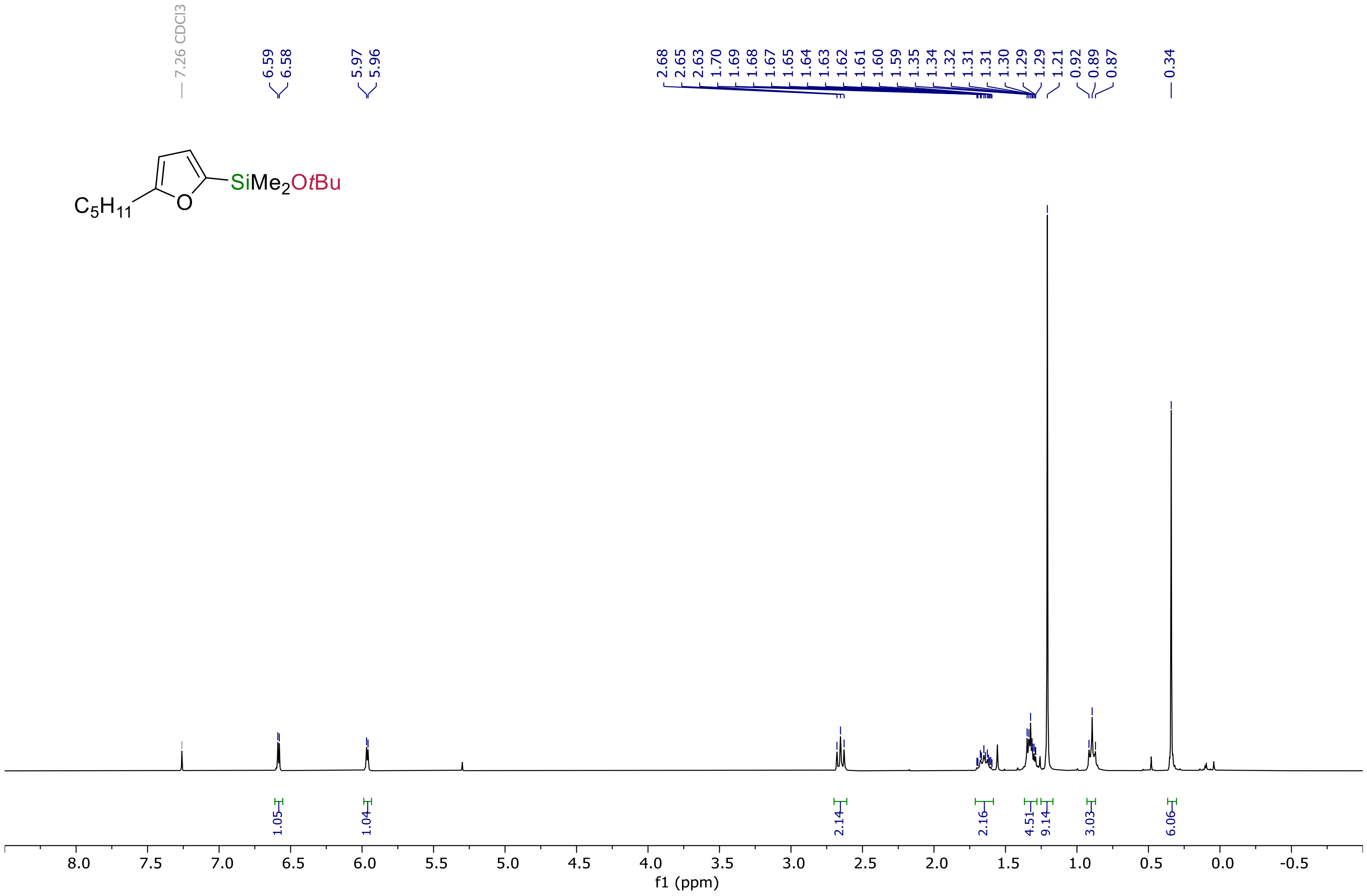

Figure S60: ^1H NMR (300 MHz, $CDCl_3$) of compound **14a.**

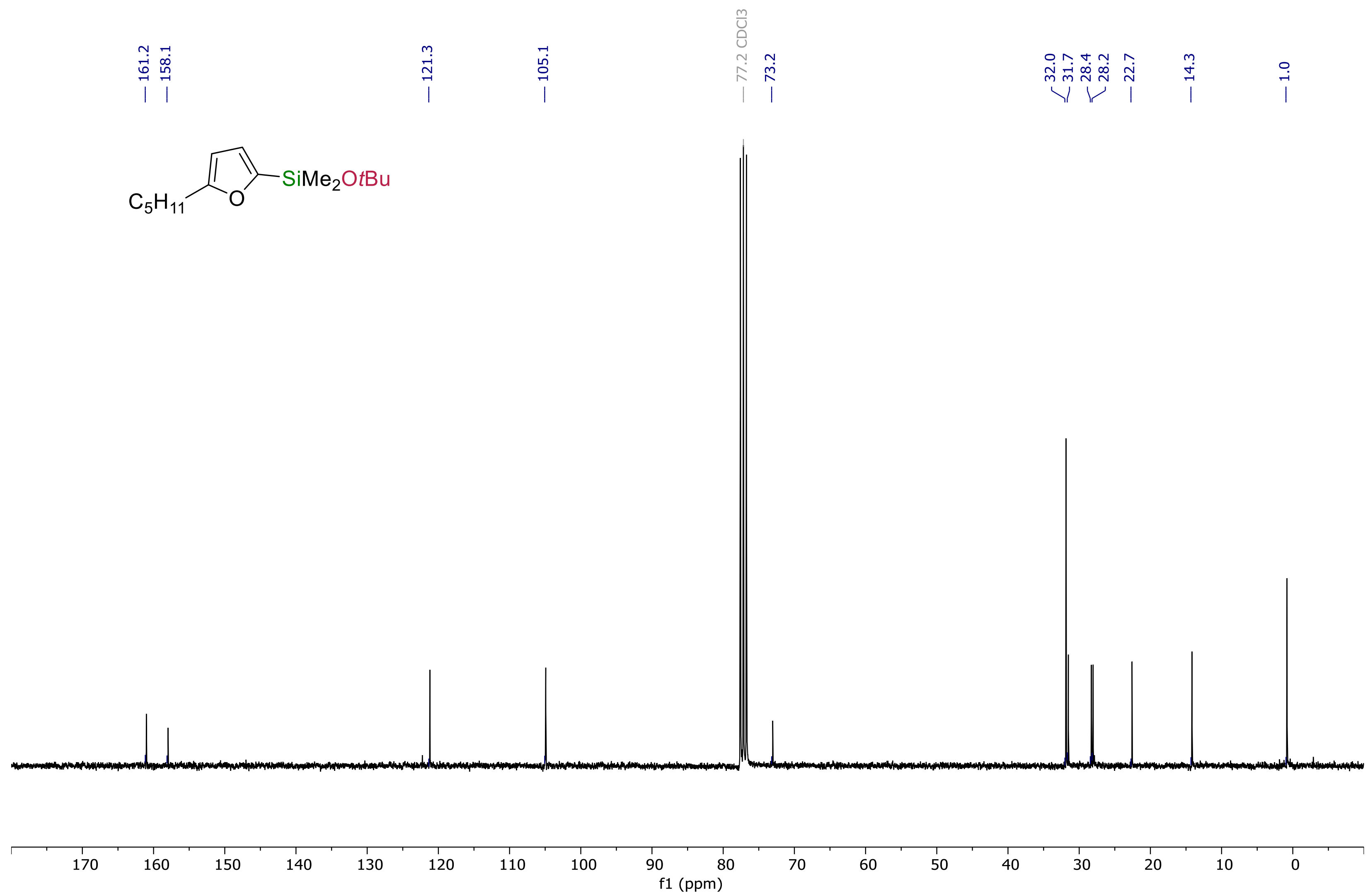

Figure S61: $^{13}C\{^{1}H\}$ NMR (75 MHz, $CDCl_3$) of compound **14a.**

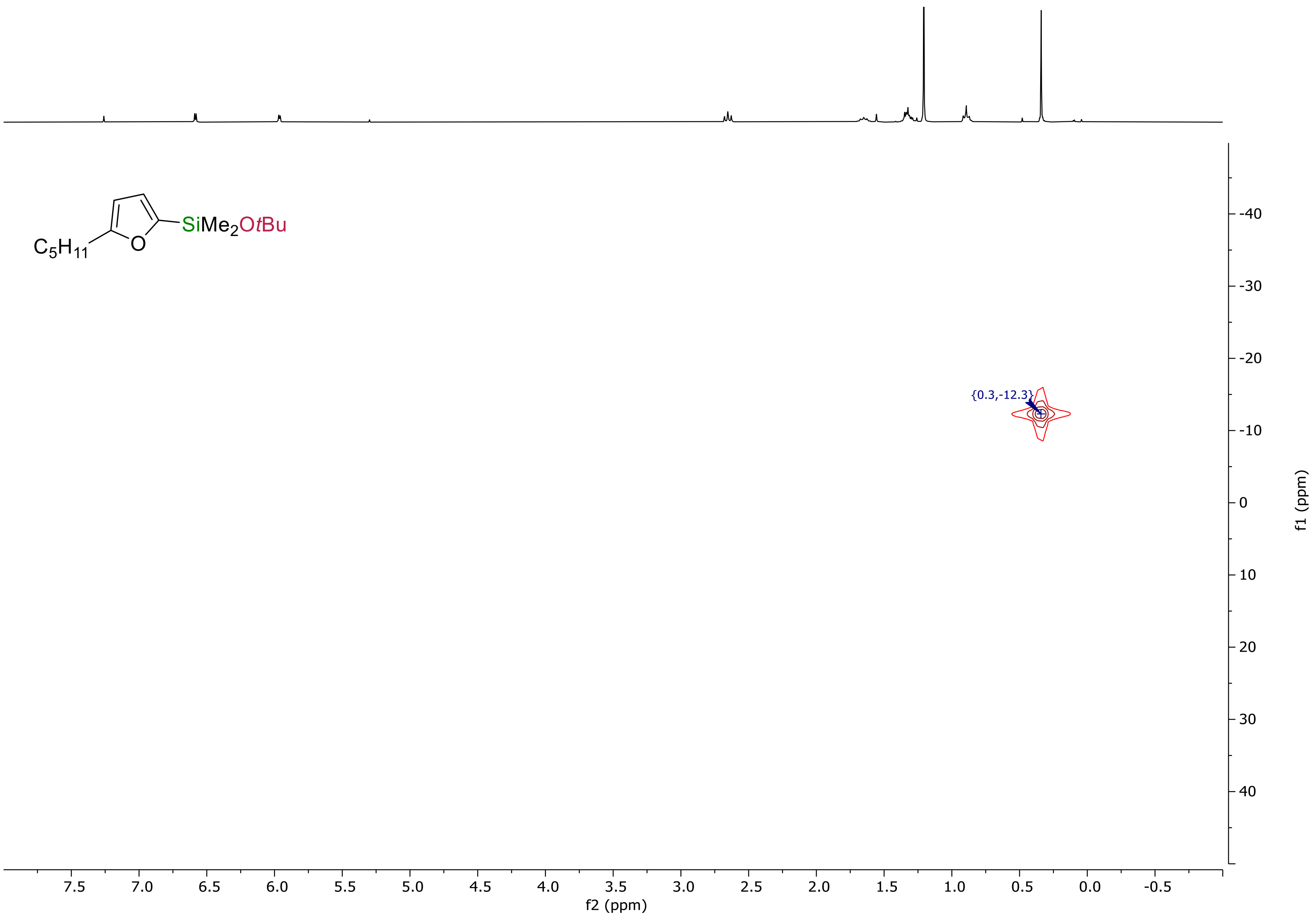

Figure S62: $^{1}H/^{29}Si$ HMQC NMR (300/60 MHz, $CDCl_3$) of compound **14a.**

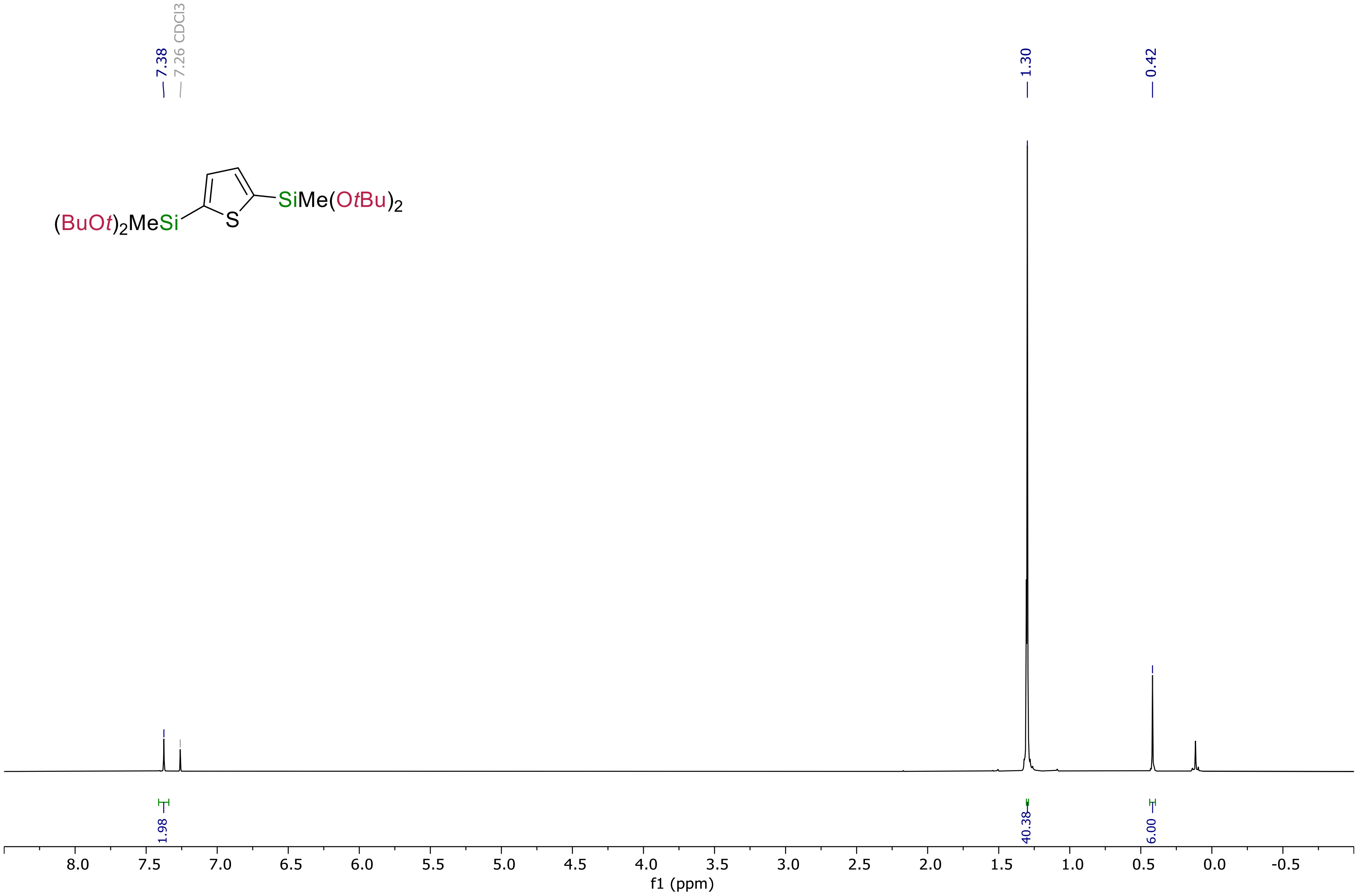

Figure S63: ^{1}H NMR (300 MHz, $CDCl_3$) of compound **15d₂**.

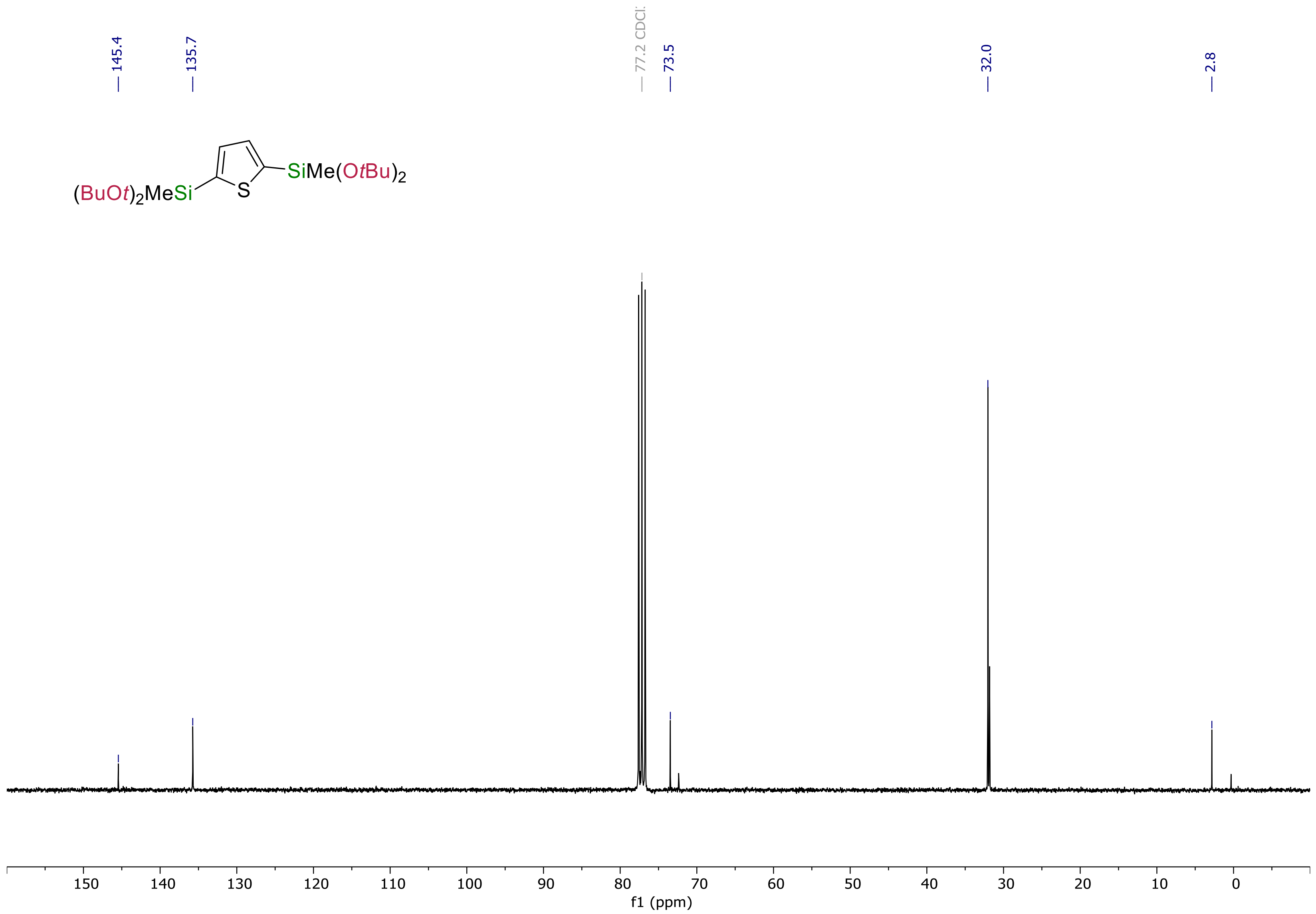

Figure S64: $^{13}C\{^{1}H\}$ NMR (75 MHz, $CDCl_3$) of compound **15d2.**

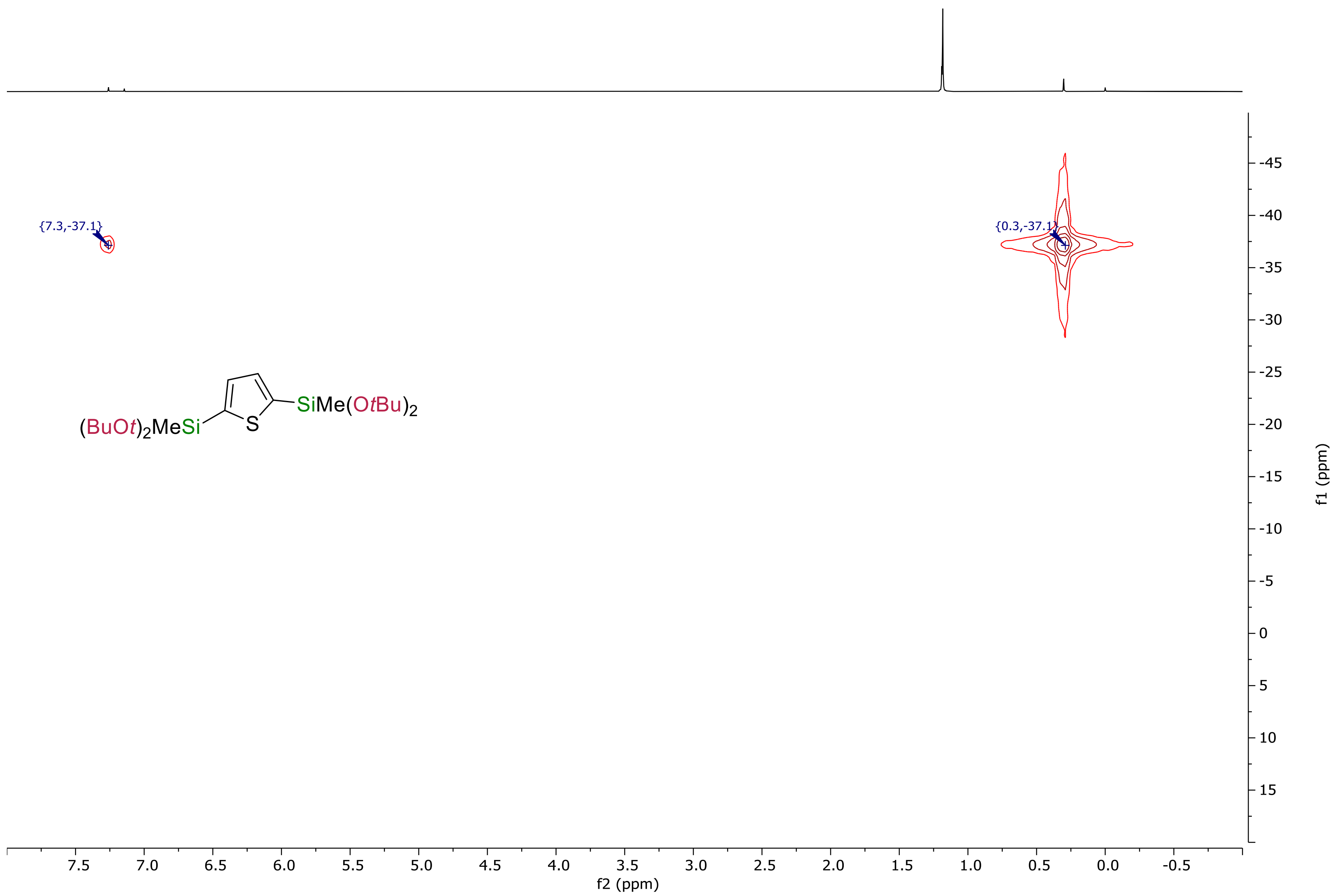

Figure S65: $^{1}H/^{29}Si$ HMQC NMR (300/60 MHz, $CDCl_3$) of compound **15d2.**

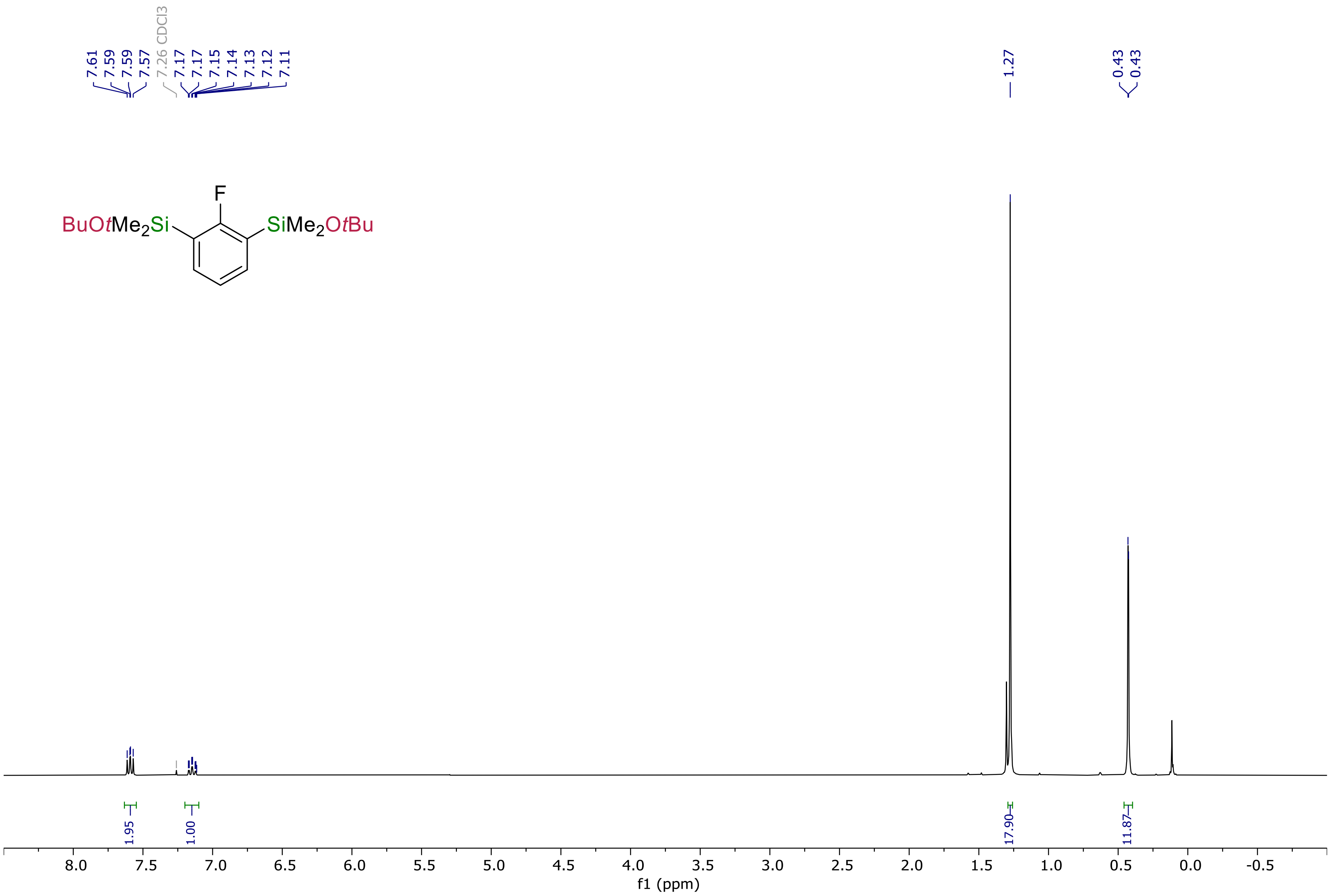

Figure S66: ^{1}H NMR (300 MHz, $CDCl_3$) of compound **16a2**.

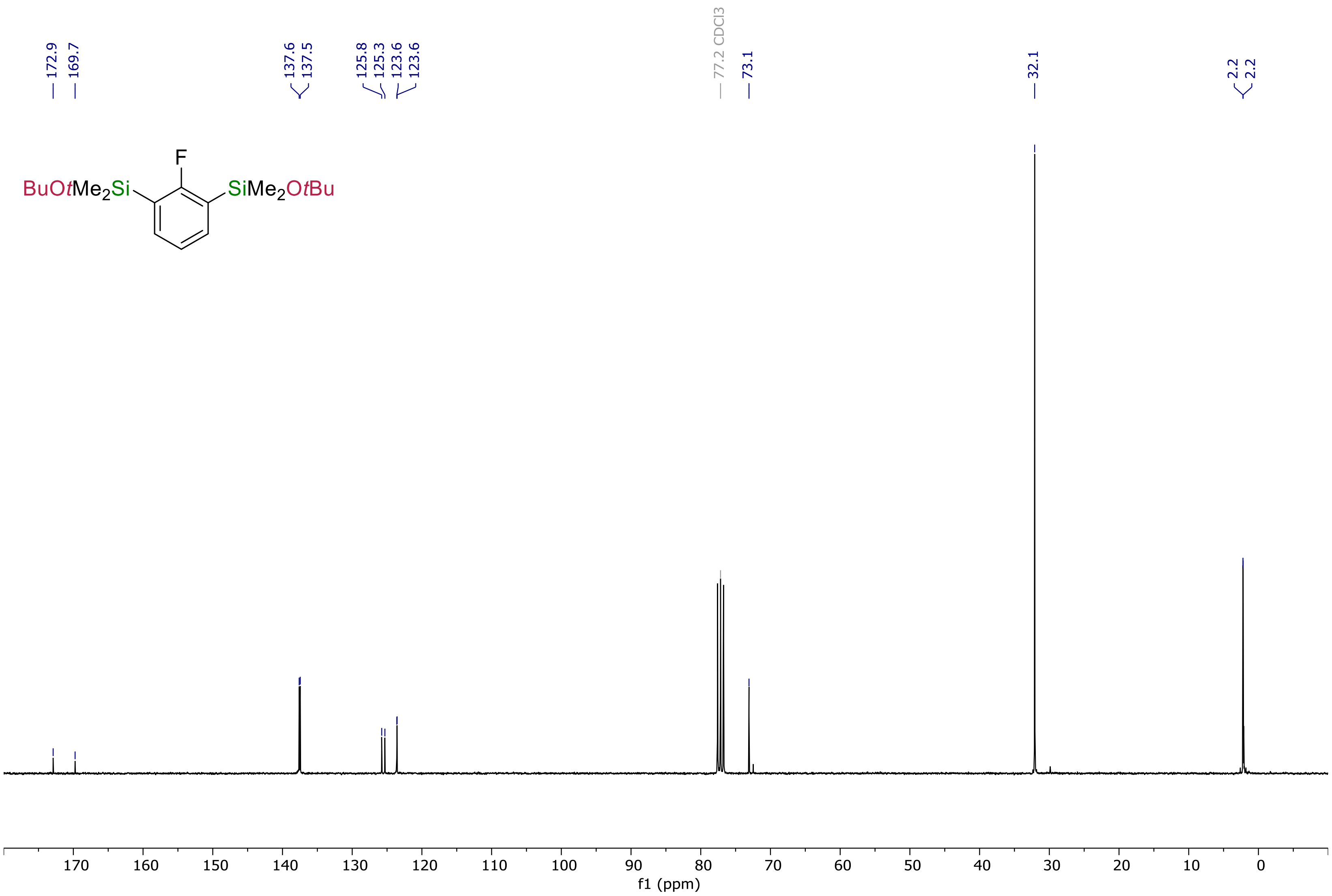

Figure S67: $^{13}C\{^1H\}$ NMR (75 MHz, $CDCl_3$) of compound **16a₂**.

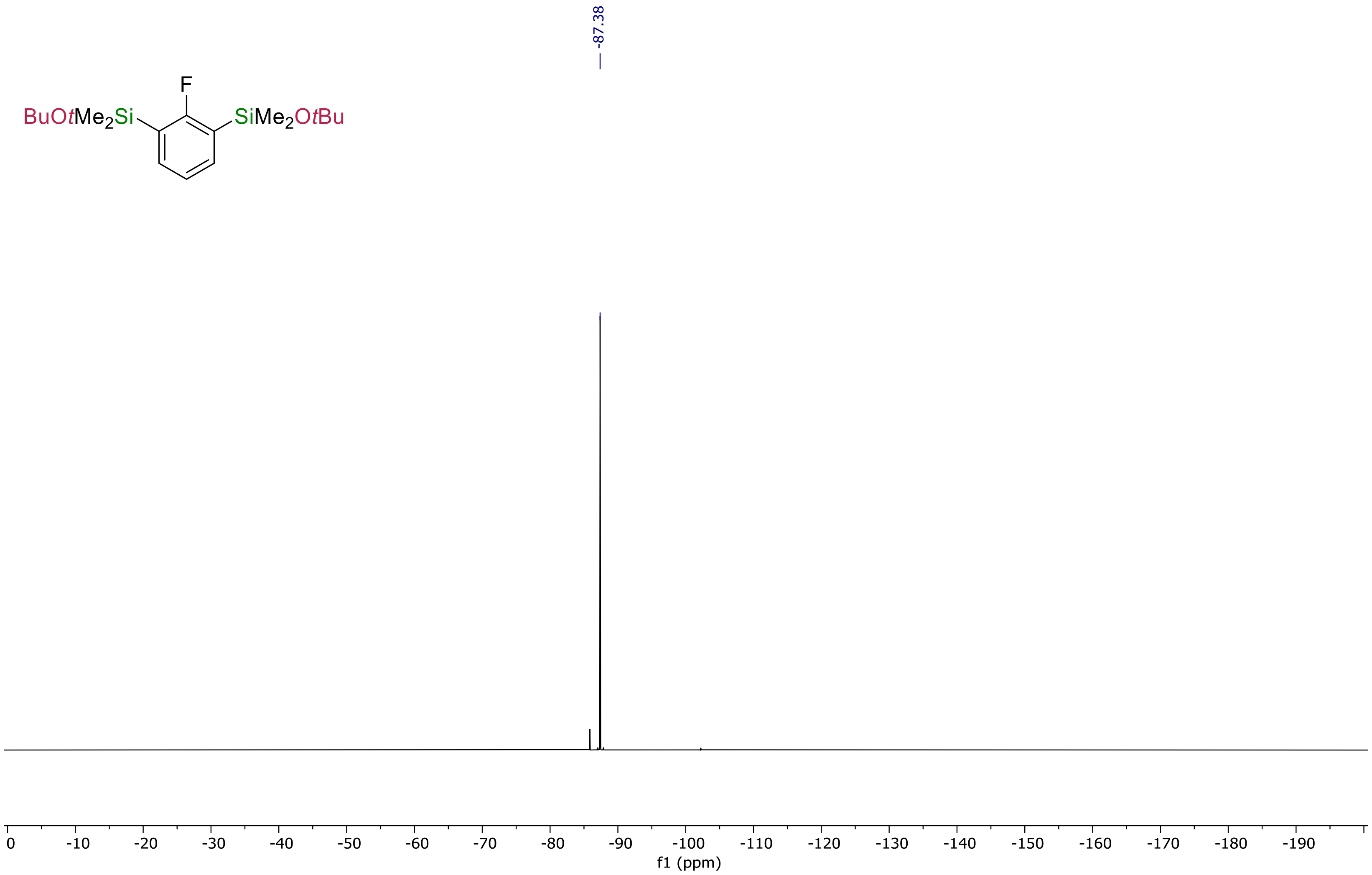

Figure S68: ^{19}F{^{1}H} NMR (282 MHz, $CDCl_3$) of compound **16a₂**.

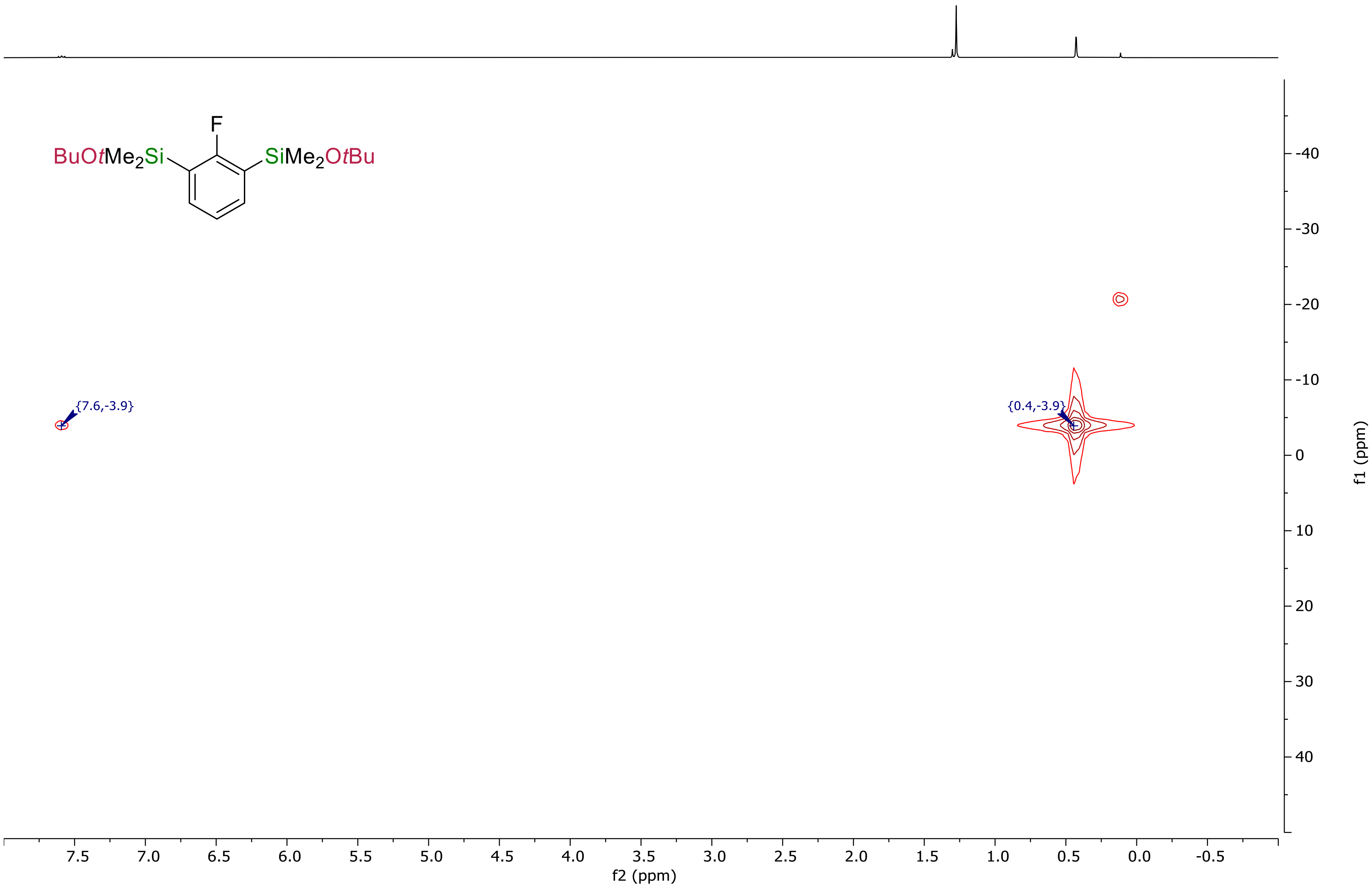

Figure S69: $^{1}H/^{29}Si$ HMQC NMR (300/60 MHz, $CDCl_3$) of compound **16a₂**.

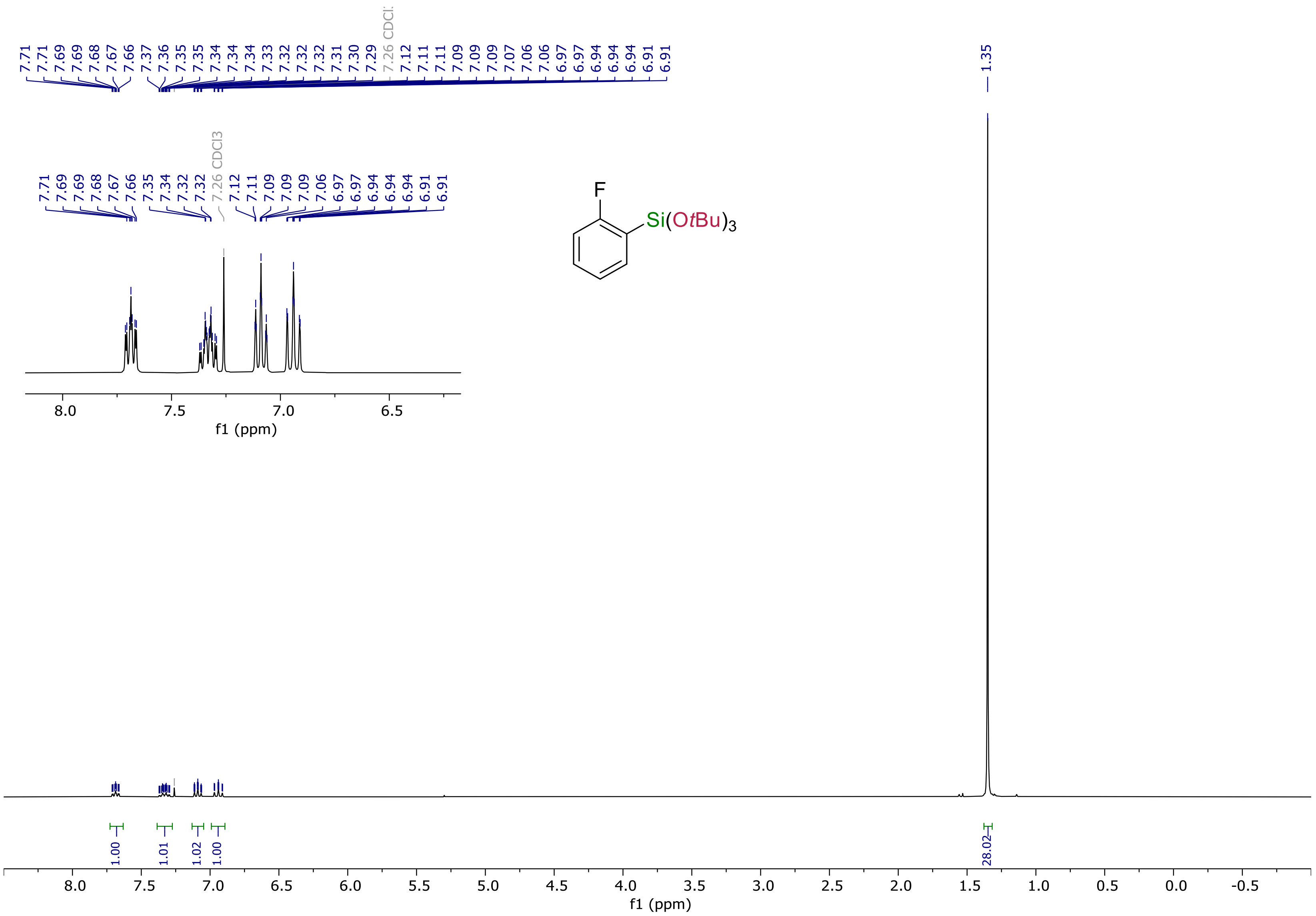

Figure S70: ^{1}H NMR (300 MHz, $CDCl_3$) of compound **16e**.

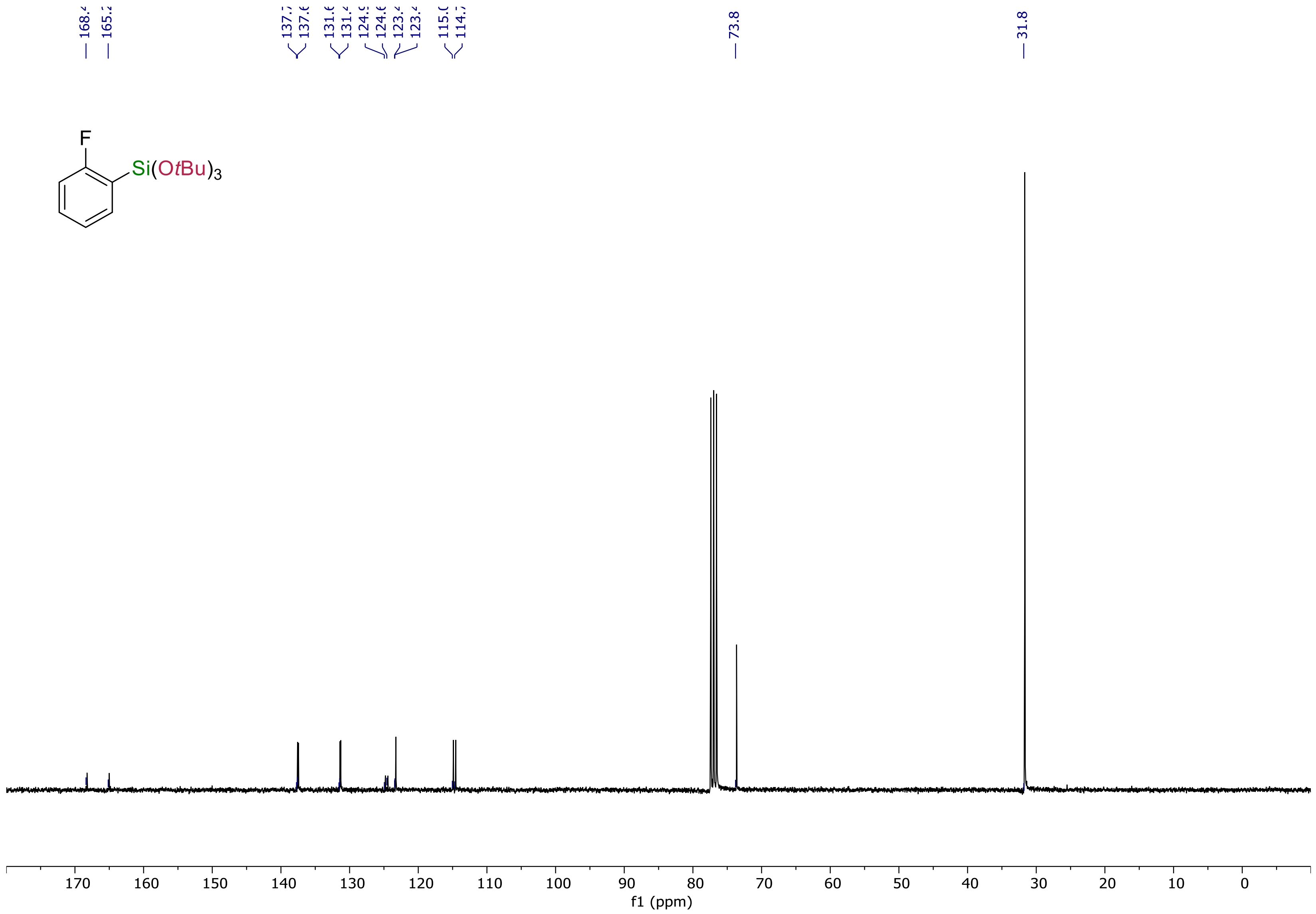

Figure S71: $^{13}C\{^{1}H\}$ NMR (75 MHz, $CDCl_3$) of compound **16e**.

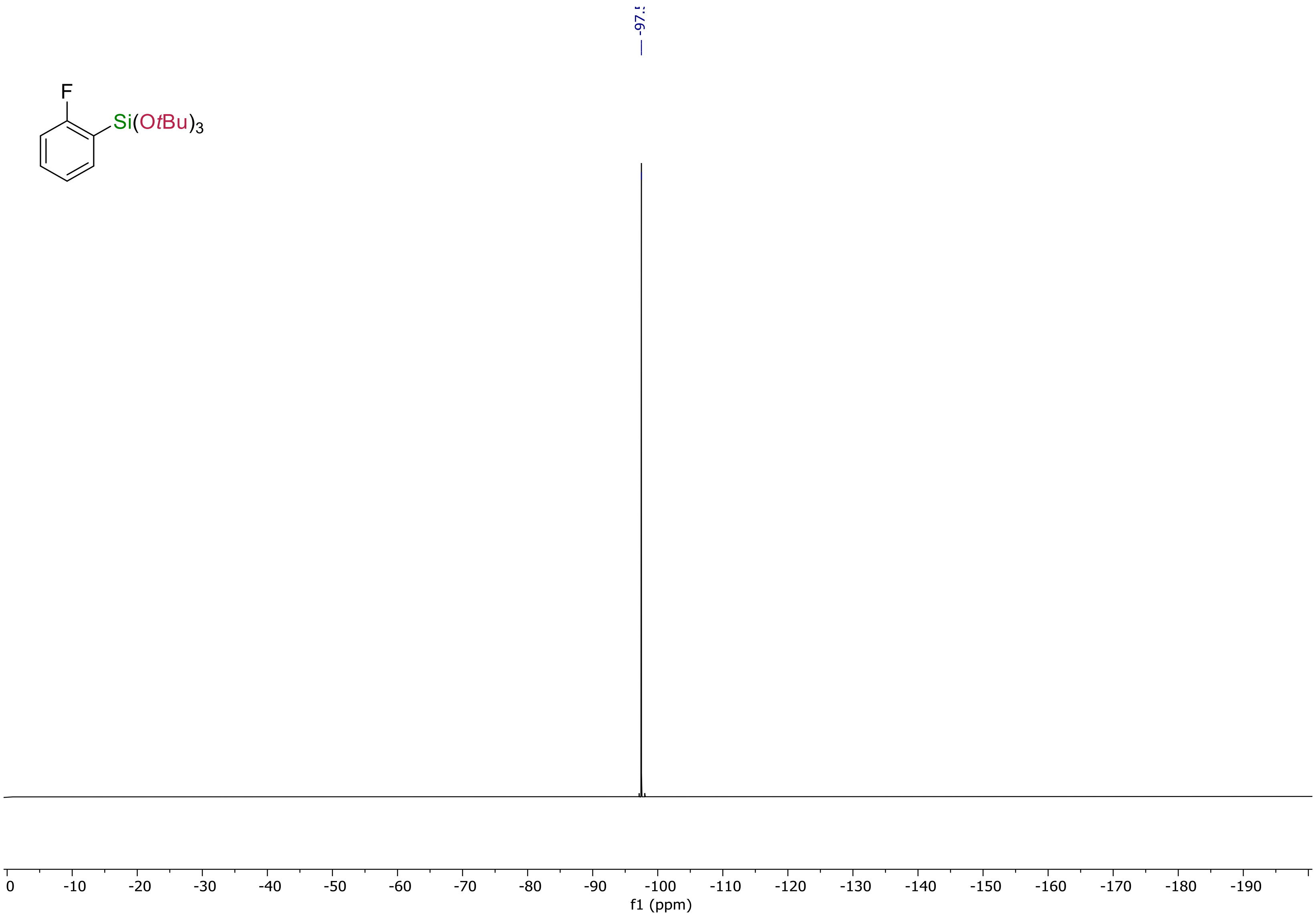

Figure S72: $^{19}F\{^{1}H\}$ NMR (282 MHz, $CDCl_3$) of compound **16e**.

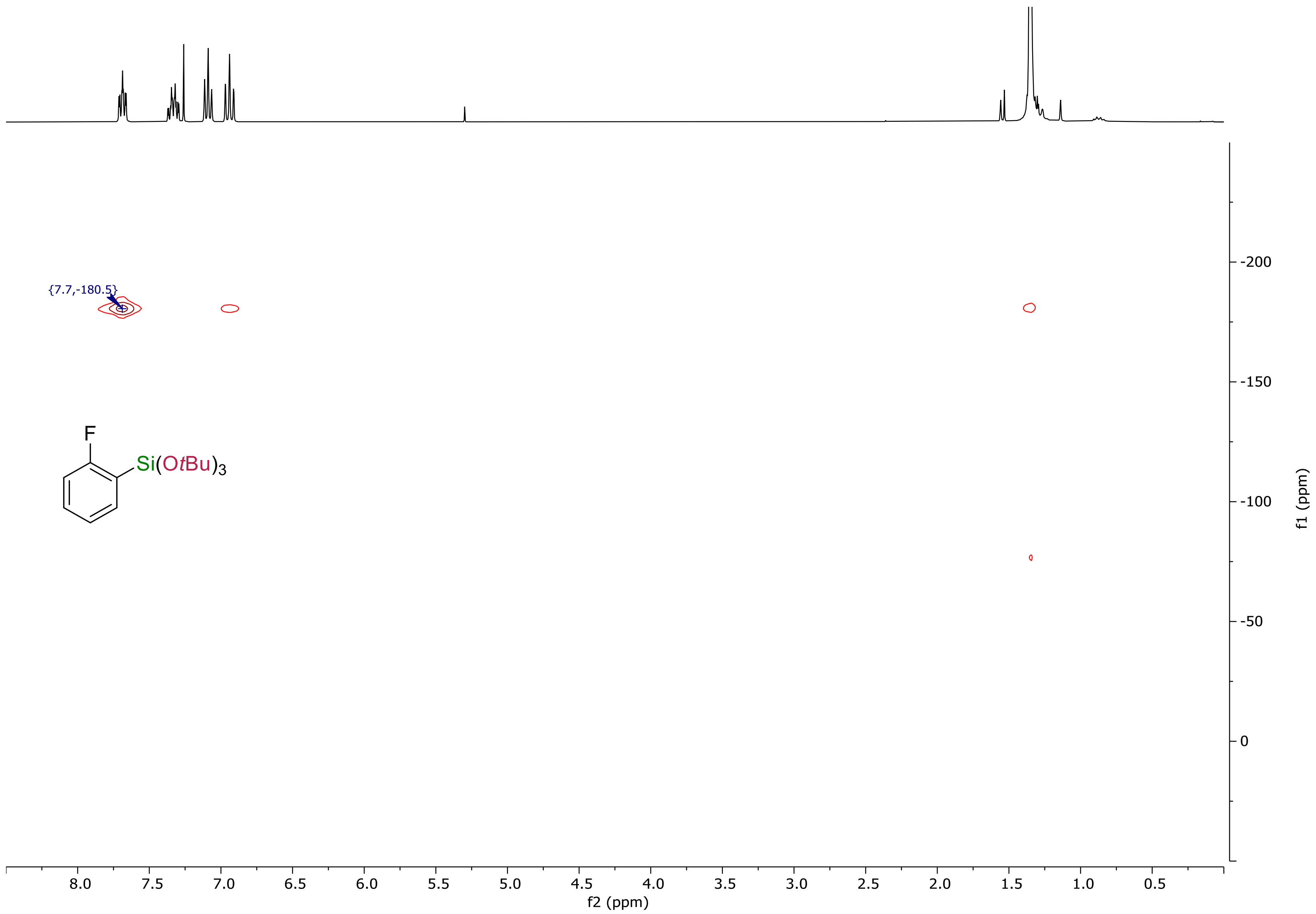

Figure S73: $^{1}H/^{29}Si$ HMQC NMR (300/60 MHz, $CDCl_3$) of compound **16e**.

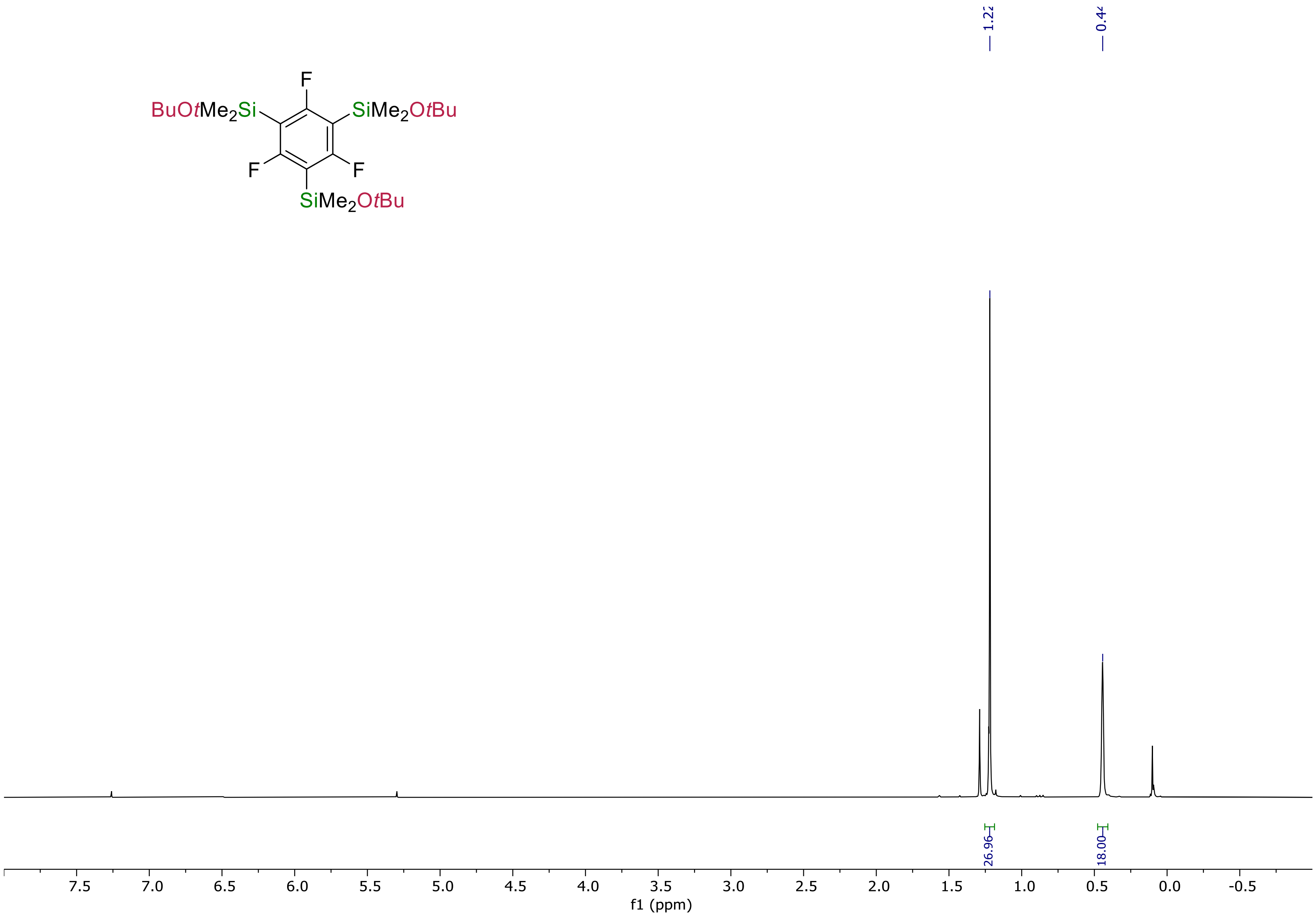

Figure S74: ^{1}H NMR (300 MHz, $CDCl_3$) of compound **17a**3.

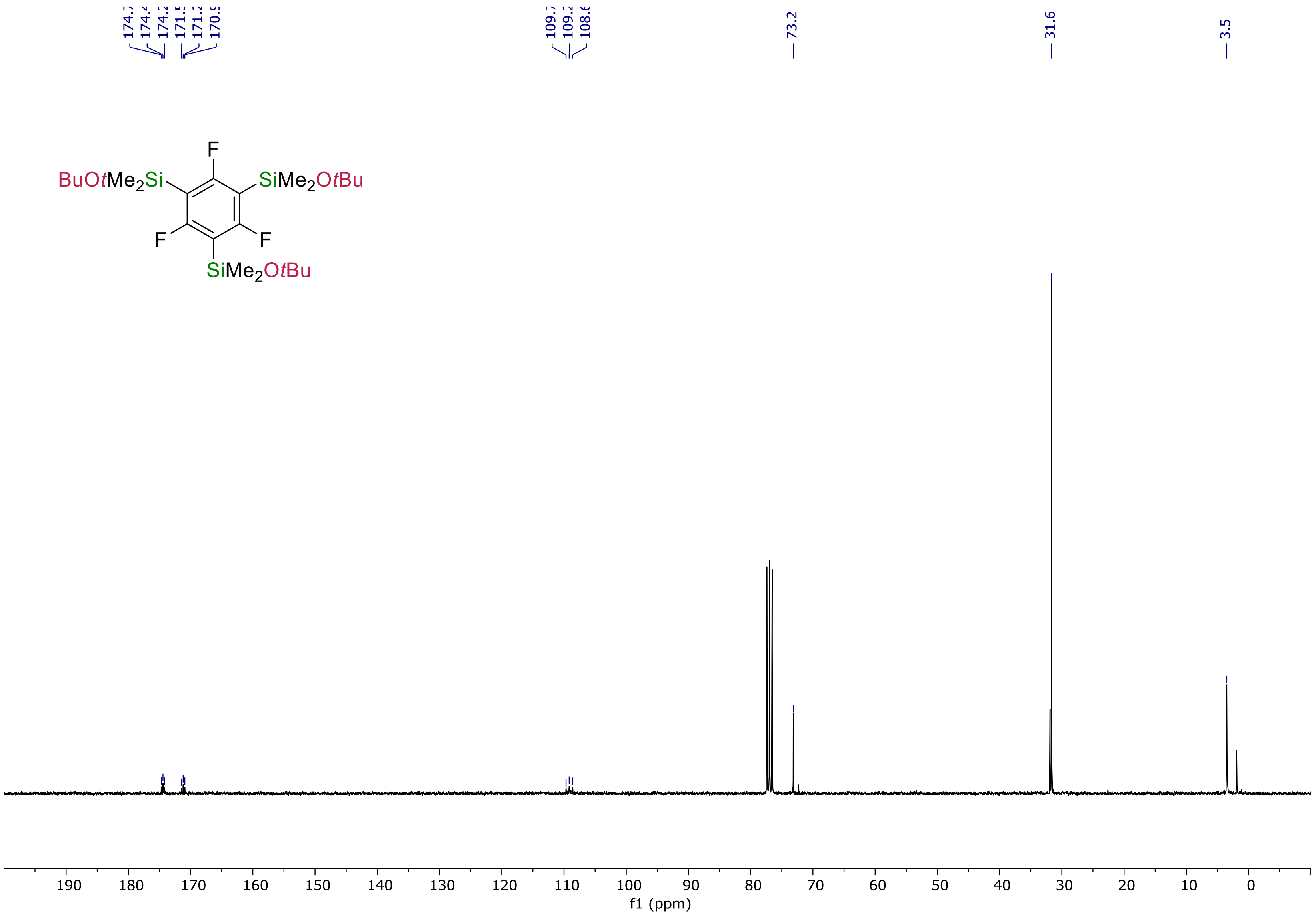

Figure S75: $^{13}C\{^{1}H\}$ NMR (75 MHz, $CDCl_3$) of compound **17a**$_3$.

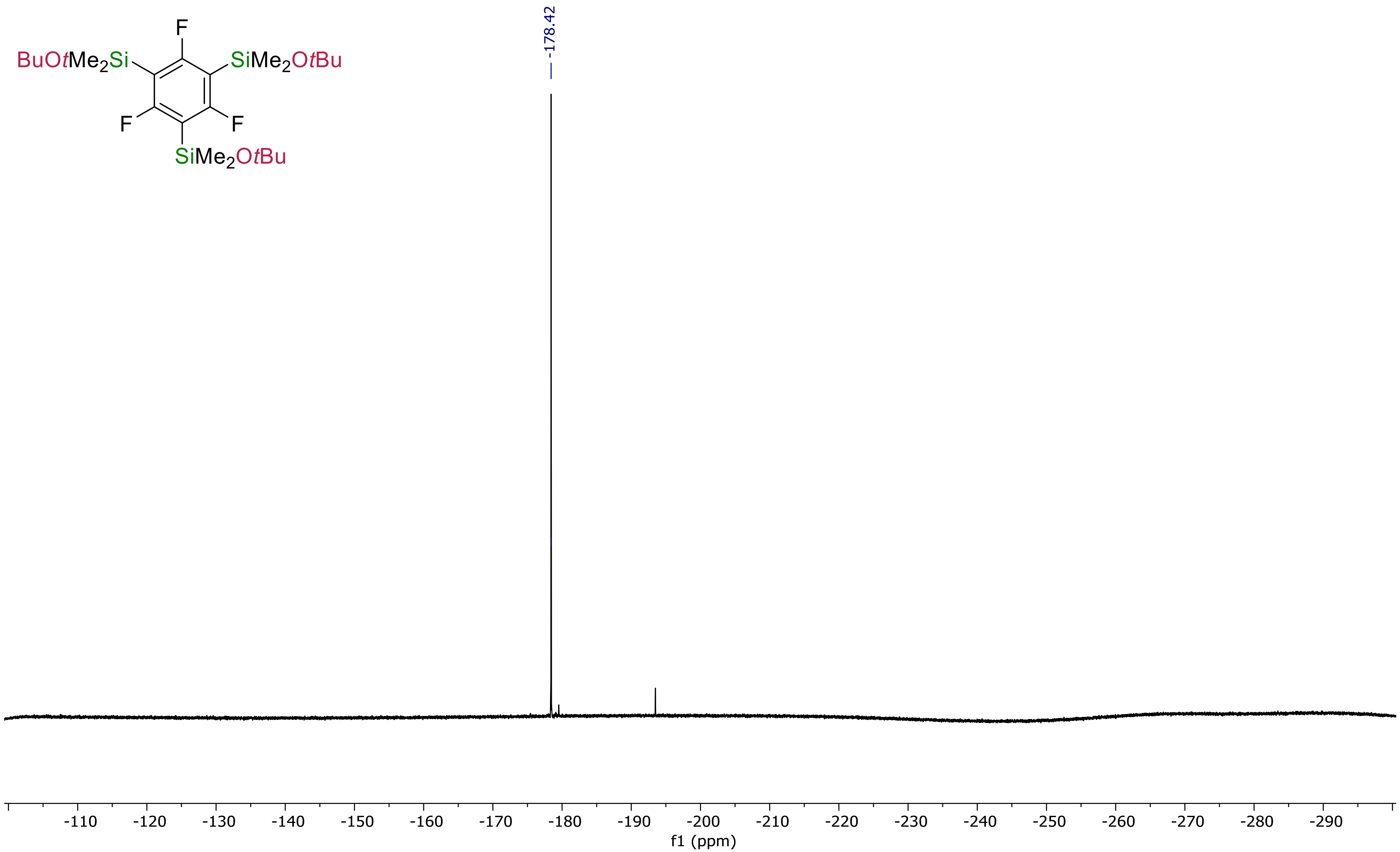

Figure S76: $^{19}F\{^{1}H\}$ NMR (282 MHz, $CDCl_3$) of compound **17a**$_3$.

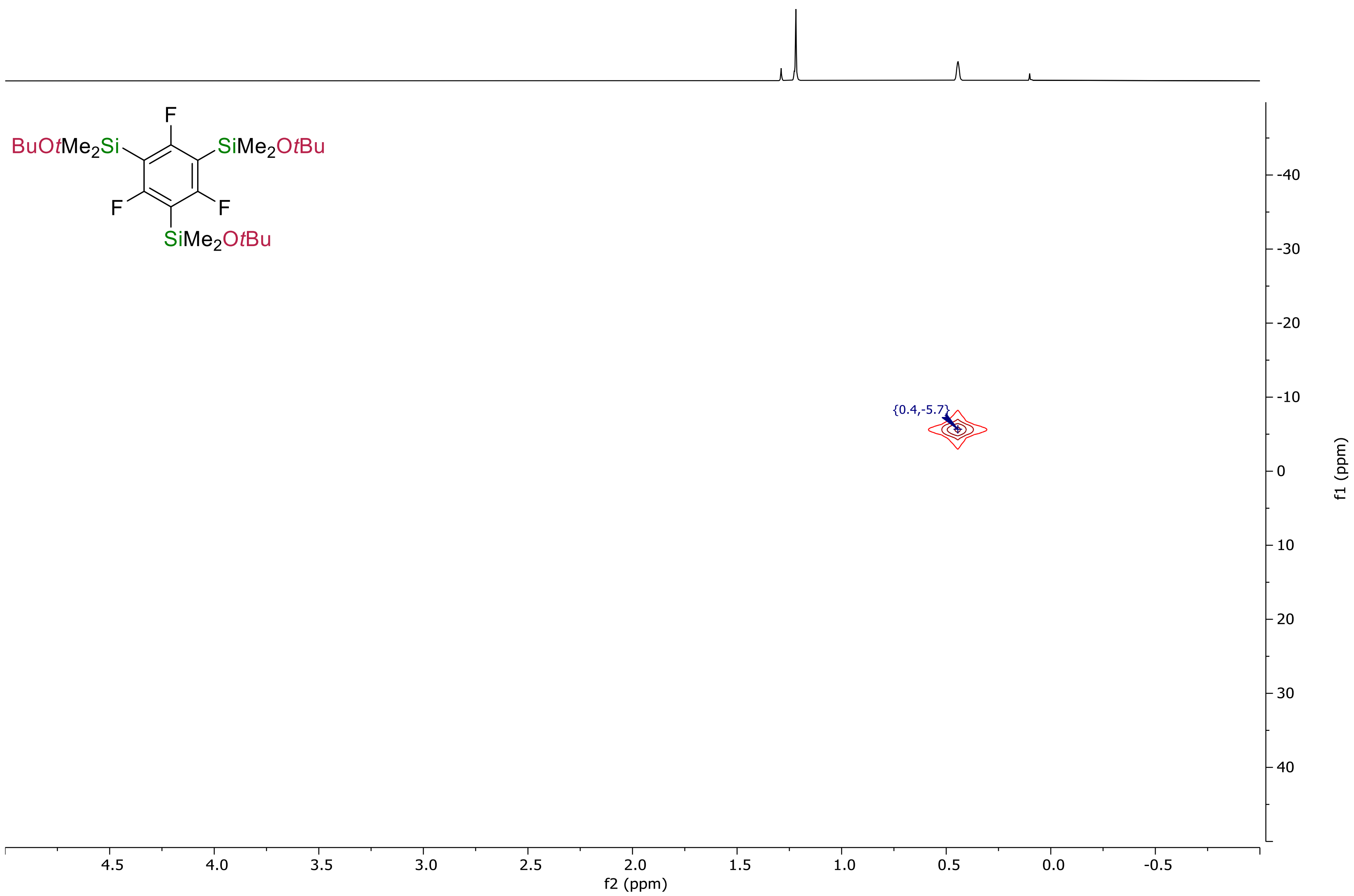

Figure S77: $^{1}H/^{29}Si$ HMQC NMR (300/60 MHz, $CDCl_3$) of compound **17a**3.

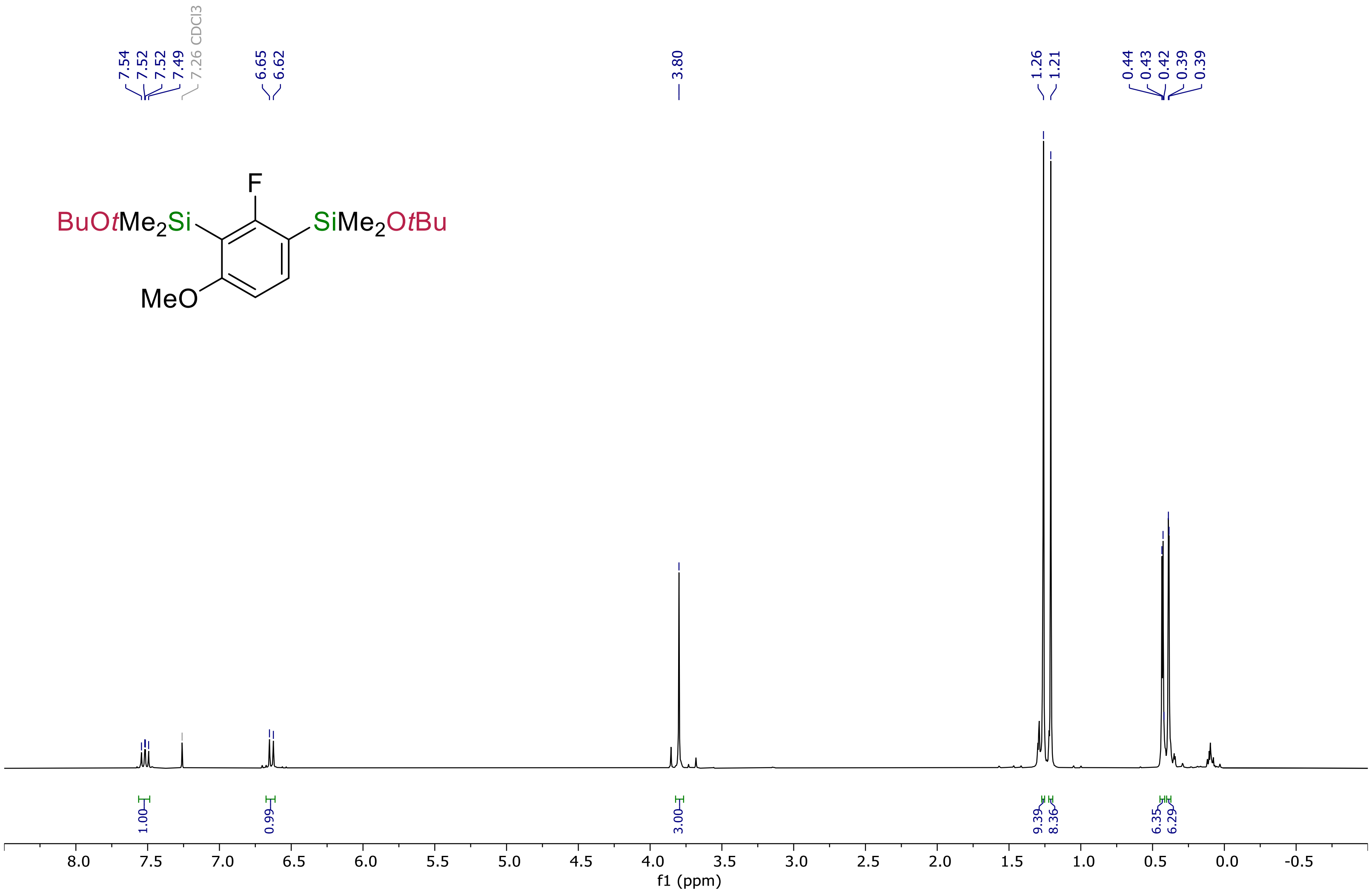

Figure S78: ^{1}H NMR (300 MHz, $CDCl_3$) of compound **18a**$_2$.

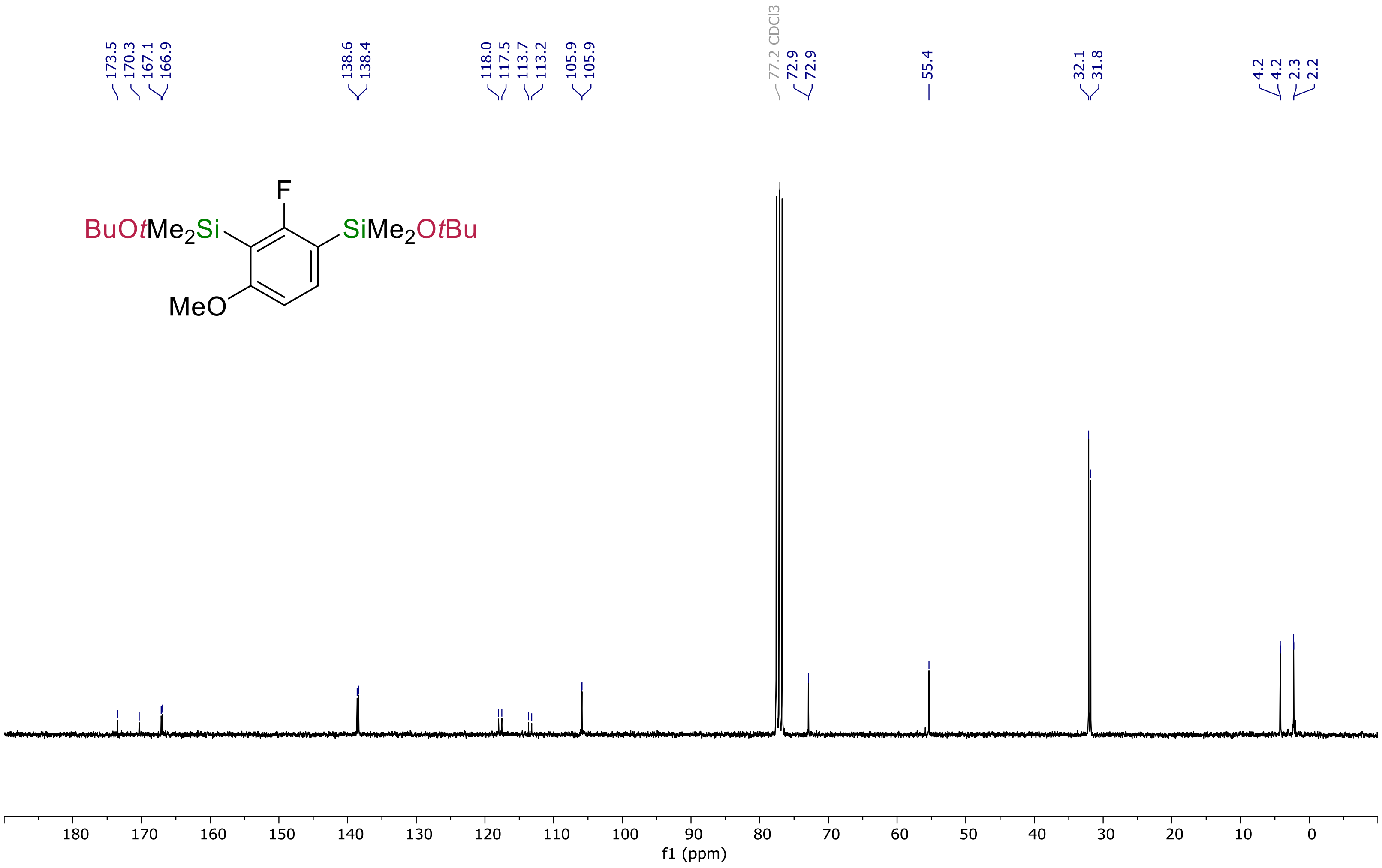

Figure S79: $^{13}C\{^1H\}$ NMR (75 MHz, $CDCl_3$) of compound **18a**$_2$.

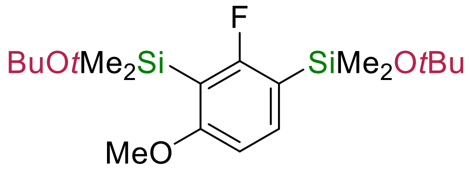

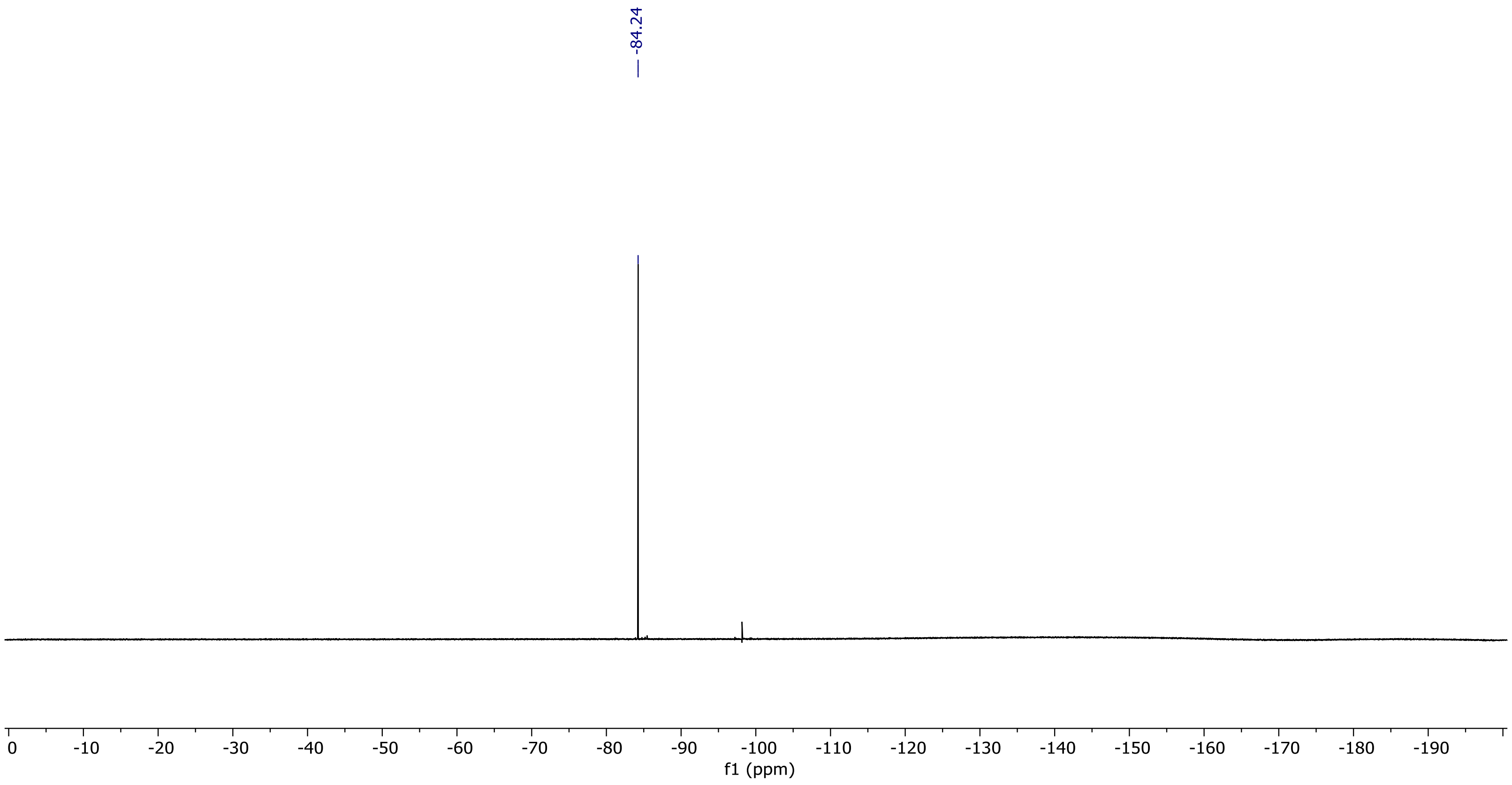

Figure S 80: $^{19}F\{^{1}H\}$ NMR (282 MHz, $CDCl_3$) of compound **18a**$_2$.

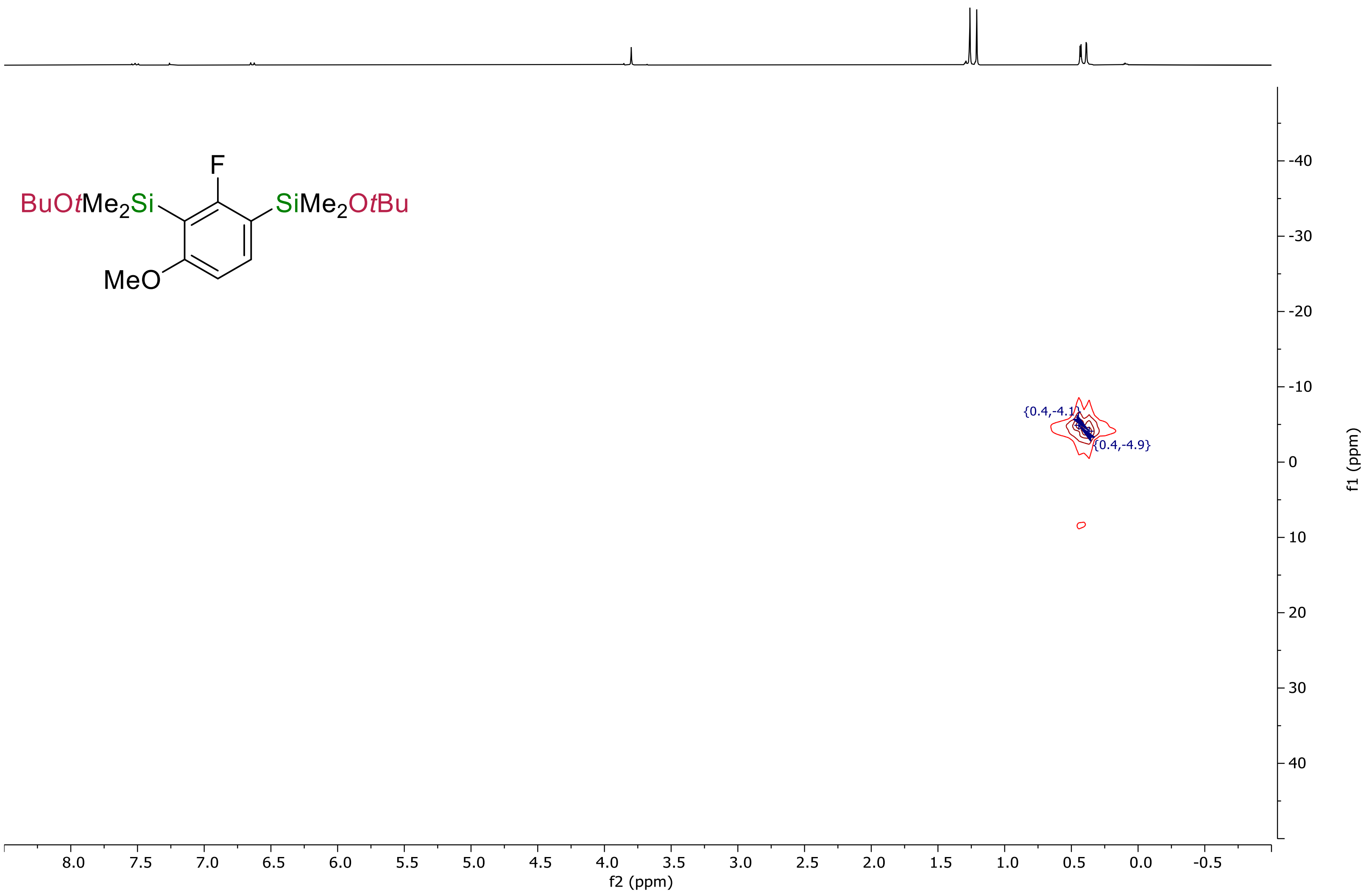

Figure S81: $^{1}H/^{29}Si$ HMQC NMR (300/60 MHz, $CDCl_3$) of compound **18a**2.

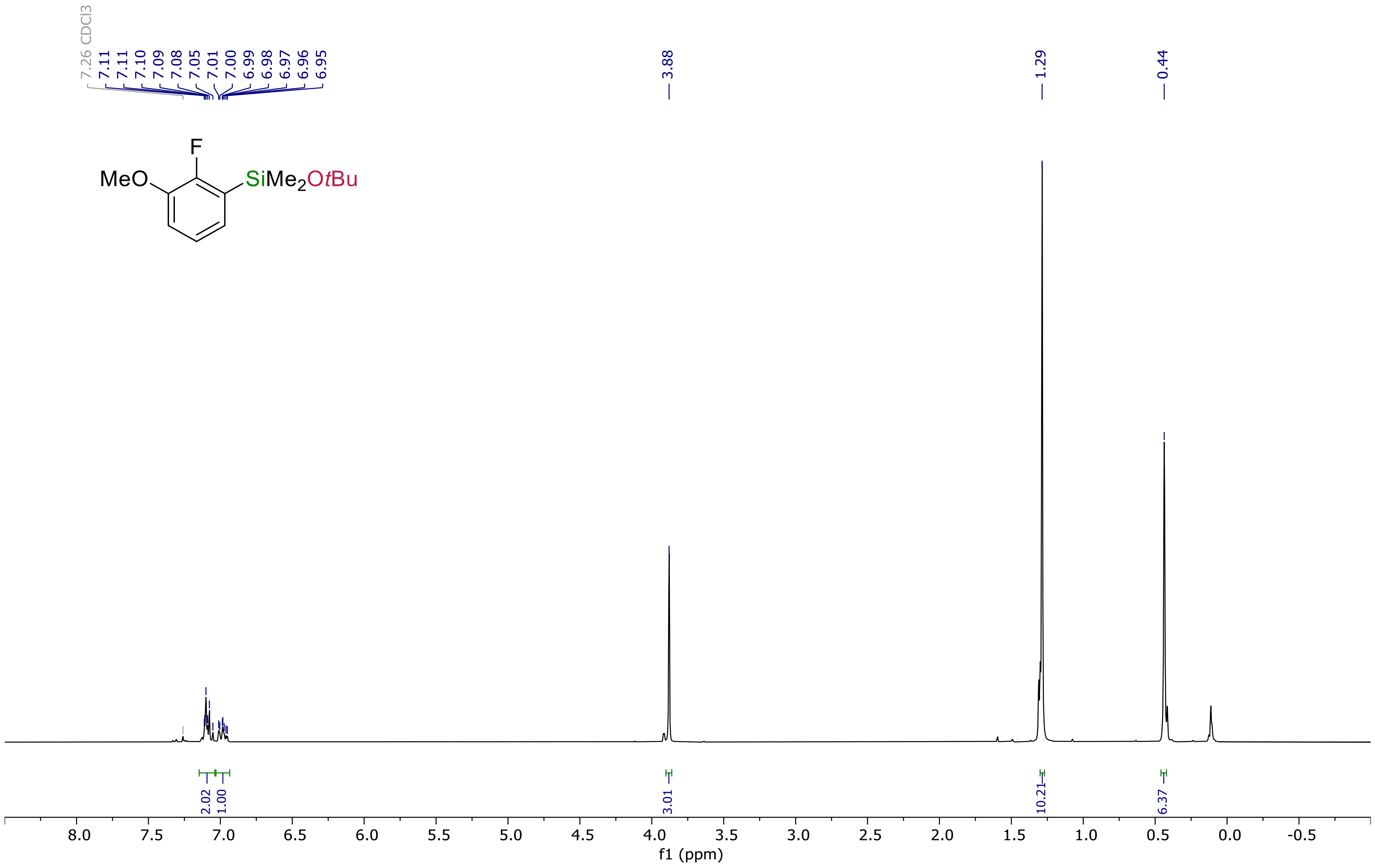

Figure S82: ^{1}H NMR (300 MHz, $CDCl_3$) of compound **19a**.

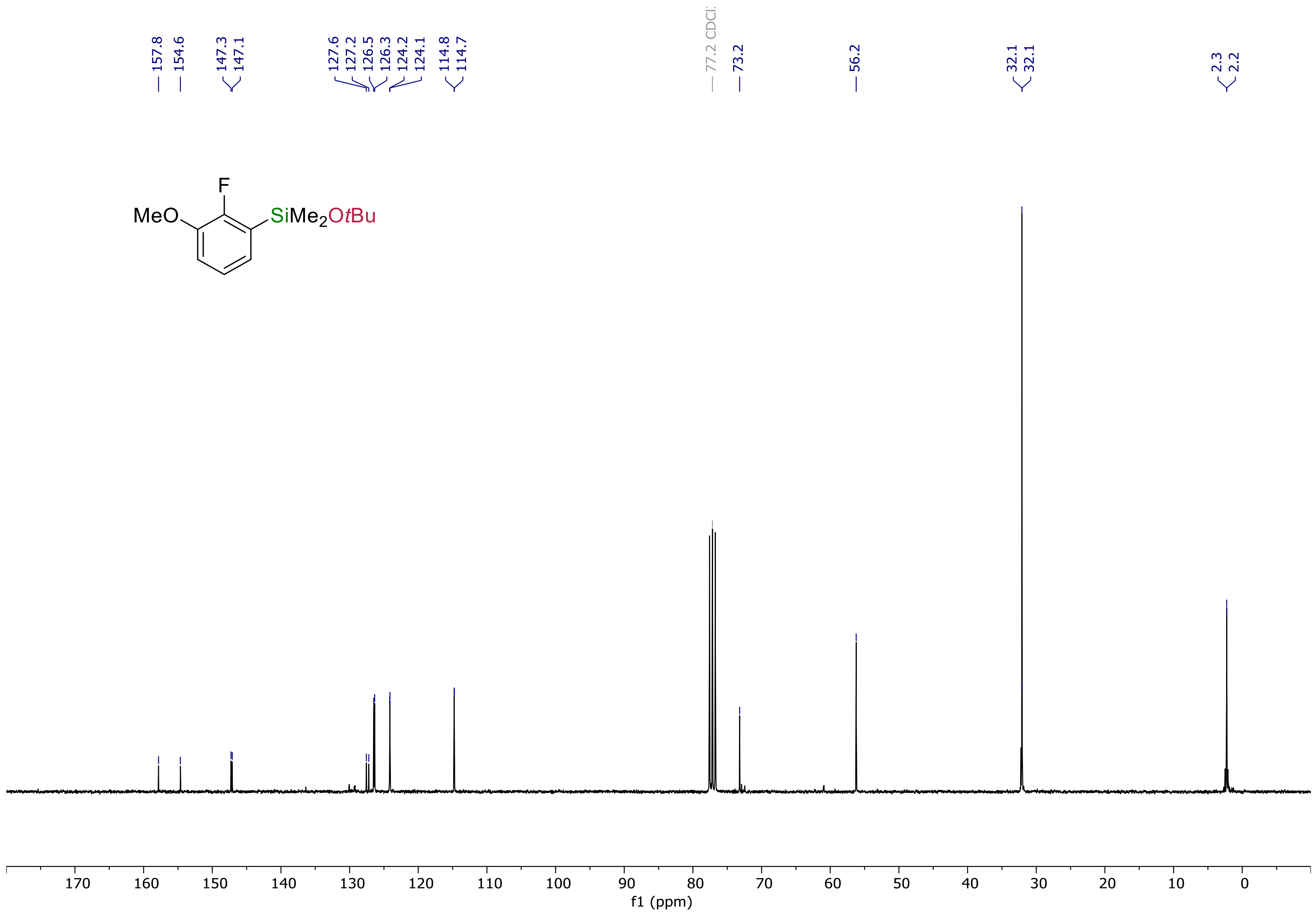

Figure S83: $^{13}C\{^{1}H\}$ NMR (75 MHz, $CDCl_3$) of compound **19a**.

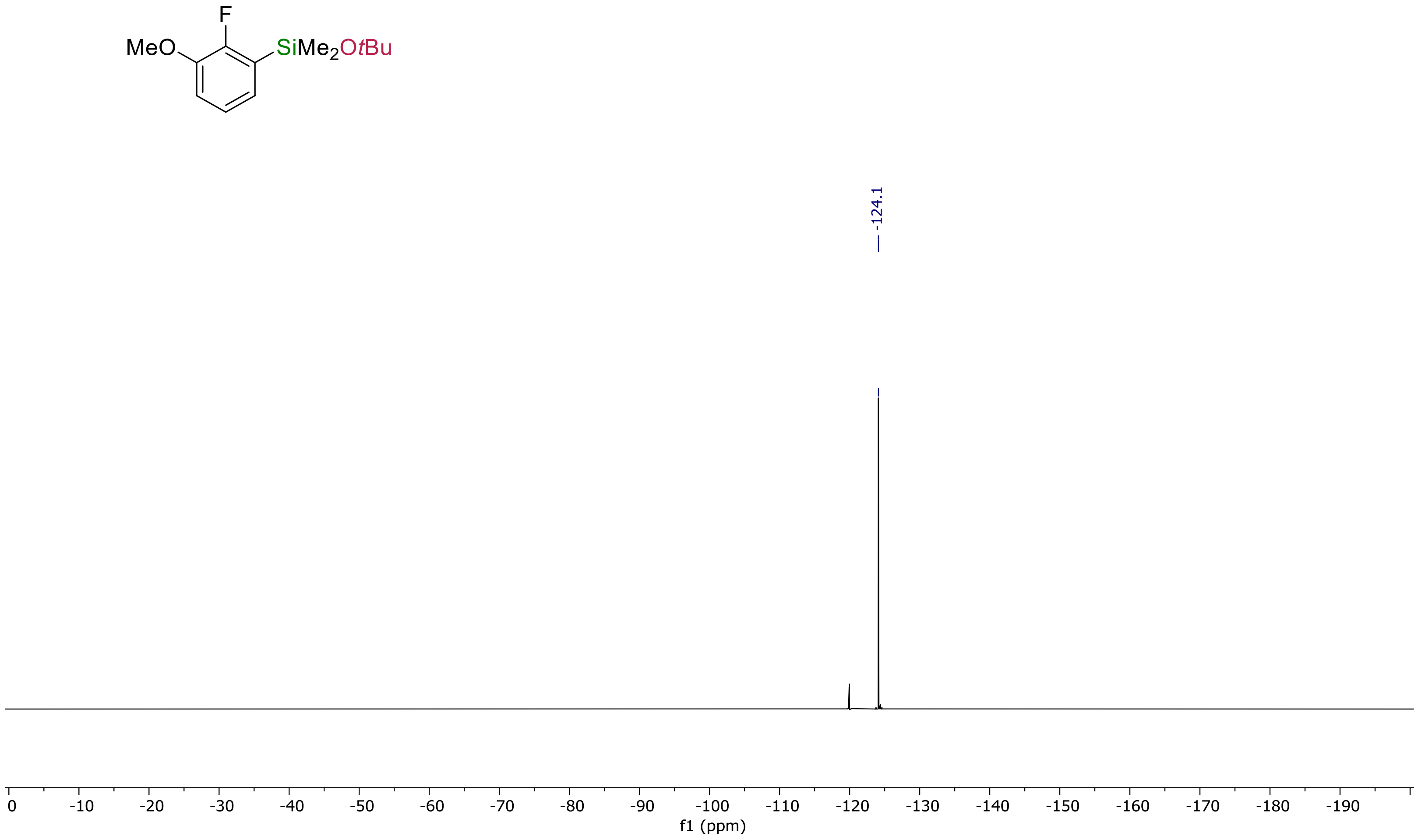

Figure S84: $^{19}F\{^{1}H\}$ NMR (282 MHz, $CDCl_3$) of compound **19a**.

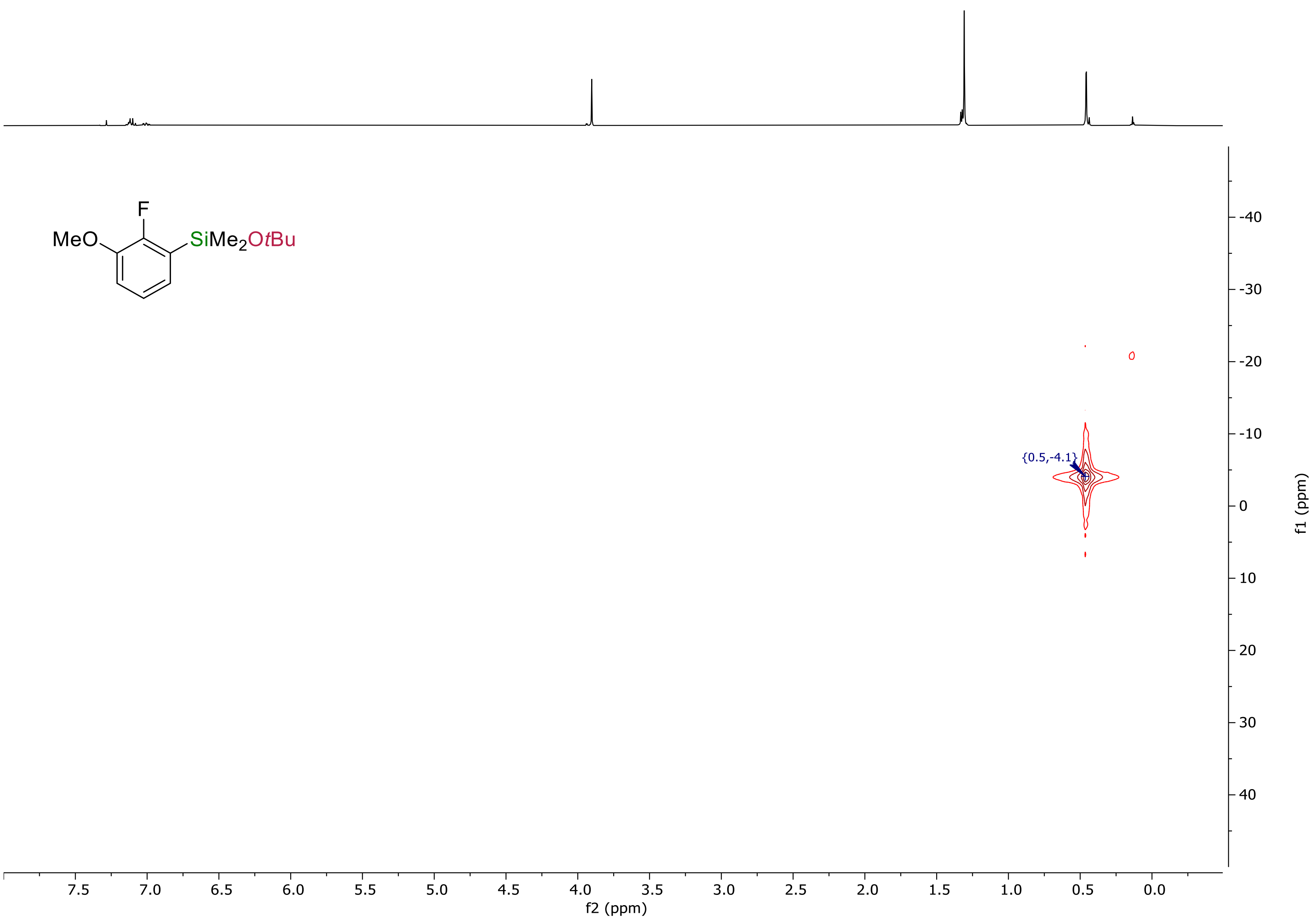

Figure S85: $^{1}H/^{29}Si$ HMQC NMR (300/60 MHz, $CDCl_3$) of compound **19a**.

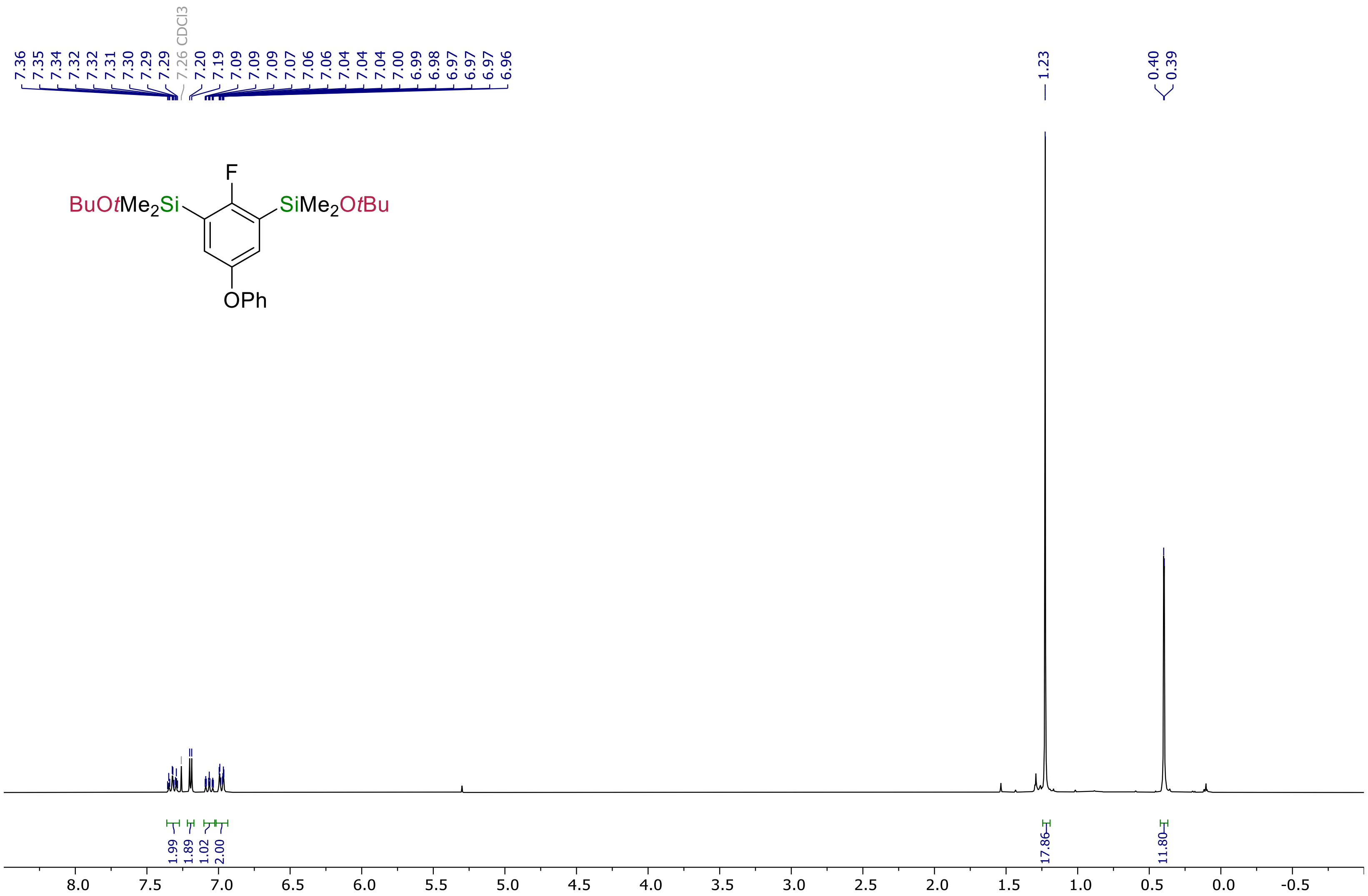

Figure S86: ^{1}H NMR (300 MHz, $CDCl_3$) of compound **20a$_2$**.

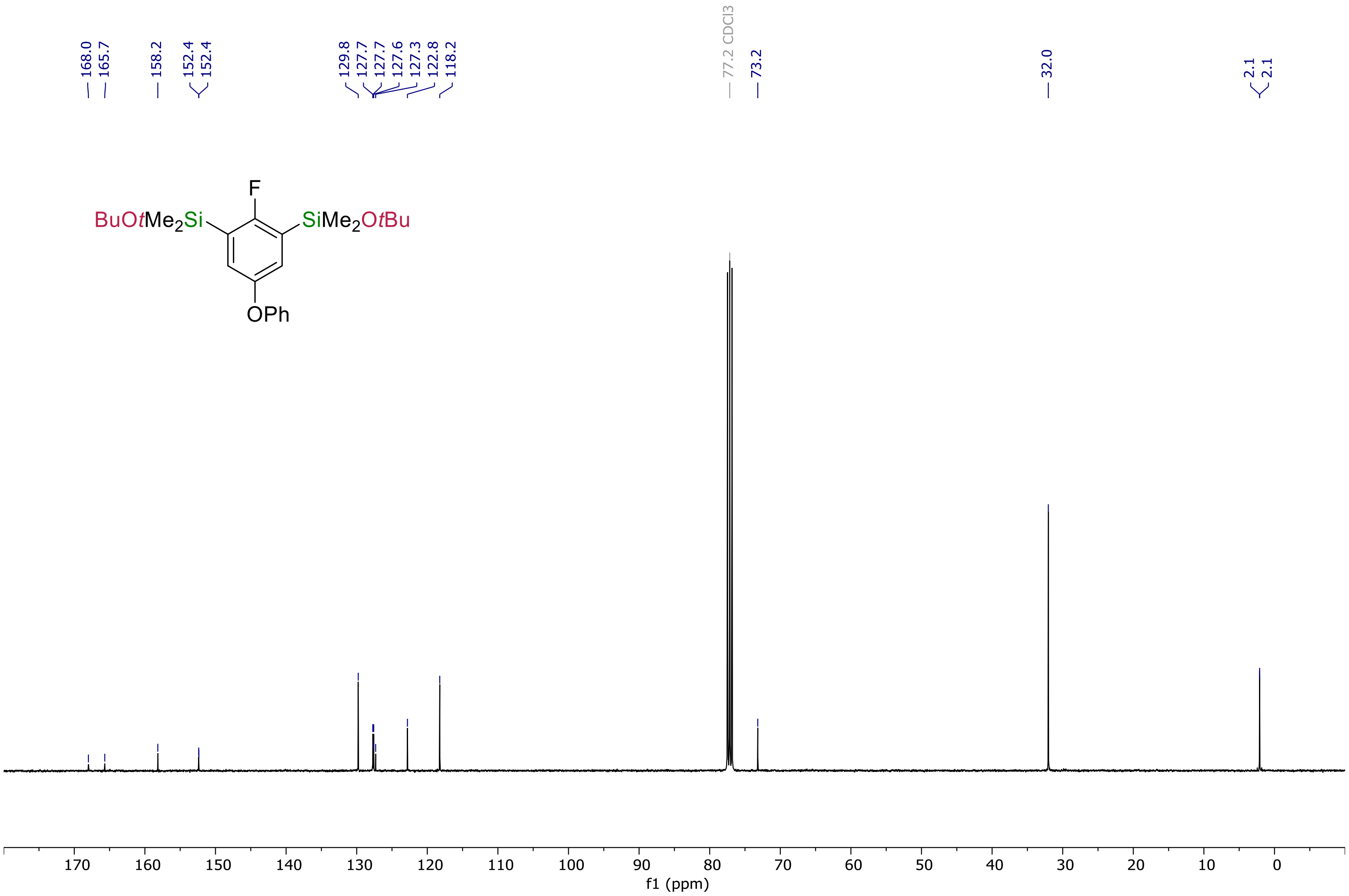

Figure S87: ^{13}C{^{1}H} NMR (75 MHz, $CDCl_3$) of compound **$20a_2$**.

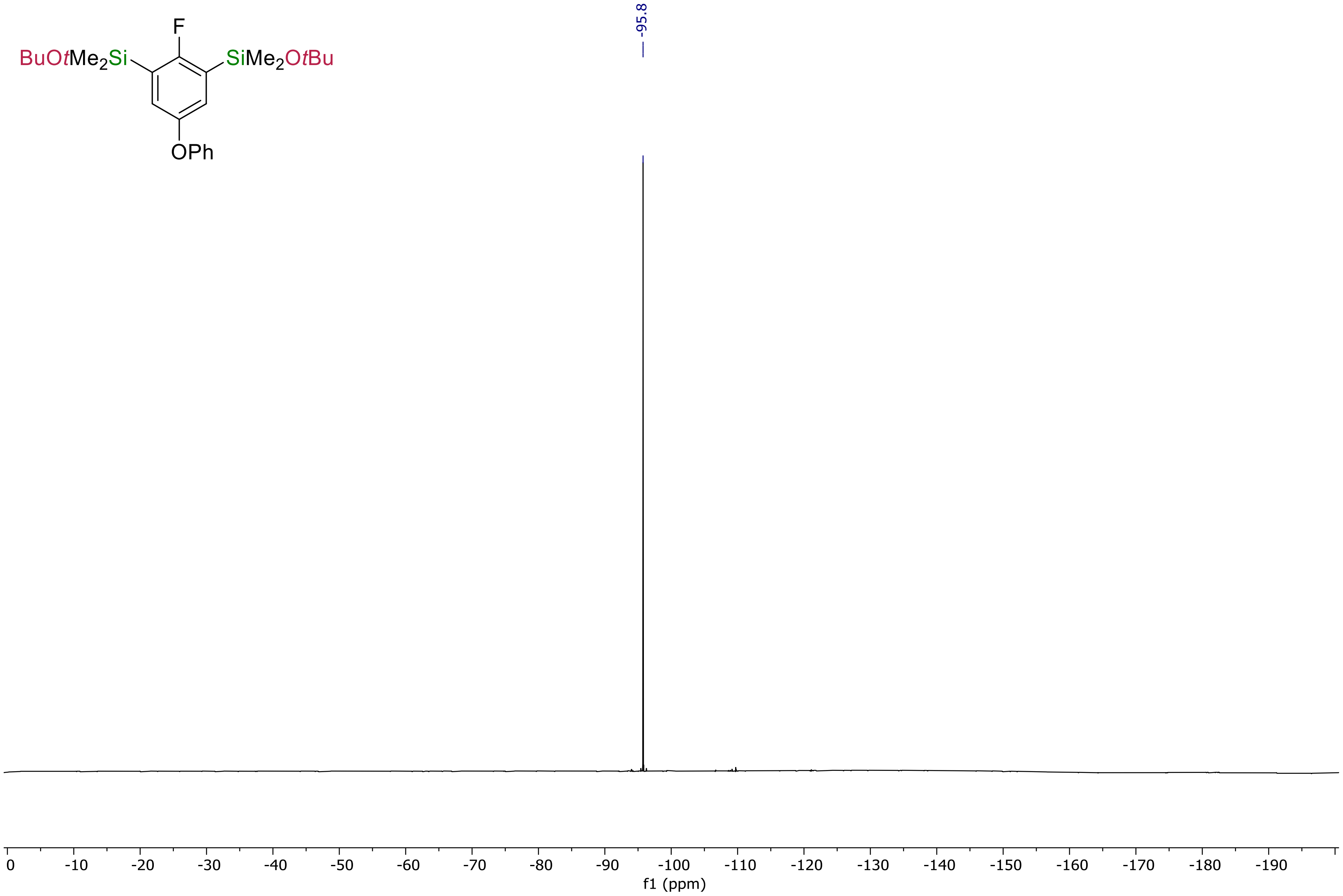

Figure S88: $^{19}F\{^{1}H\}$ NMR (282 MHz, $CDCl_3$) of compound **20a₂**.

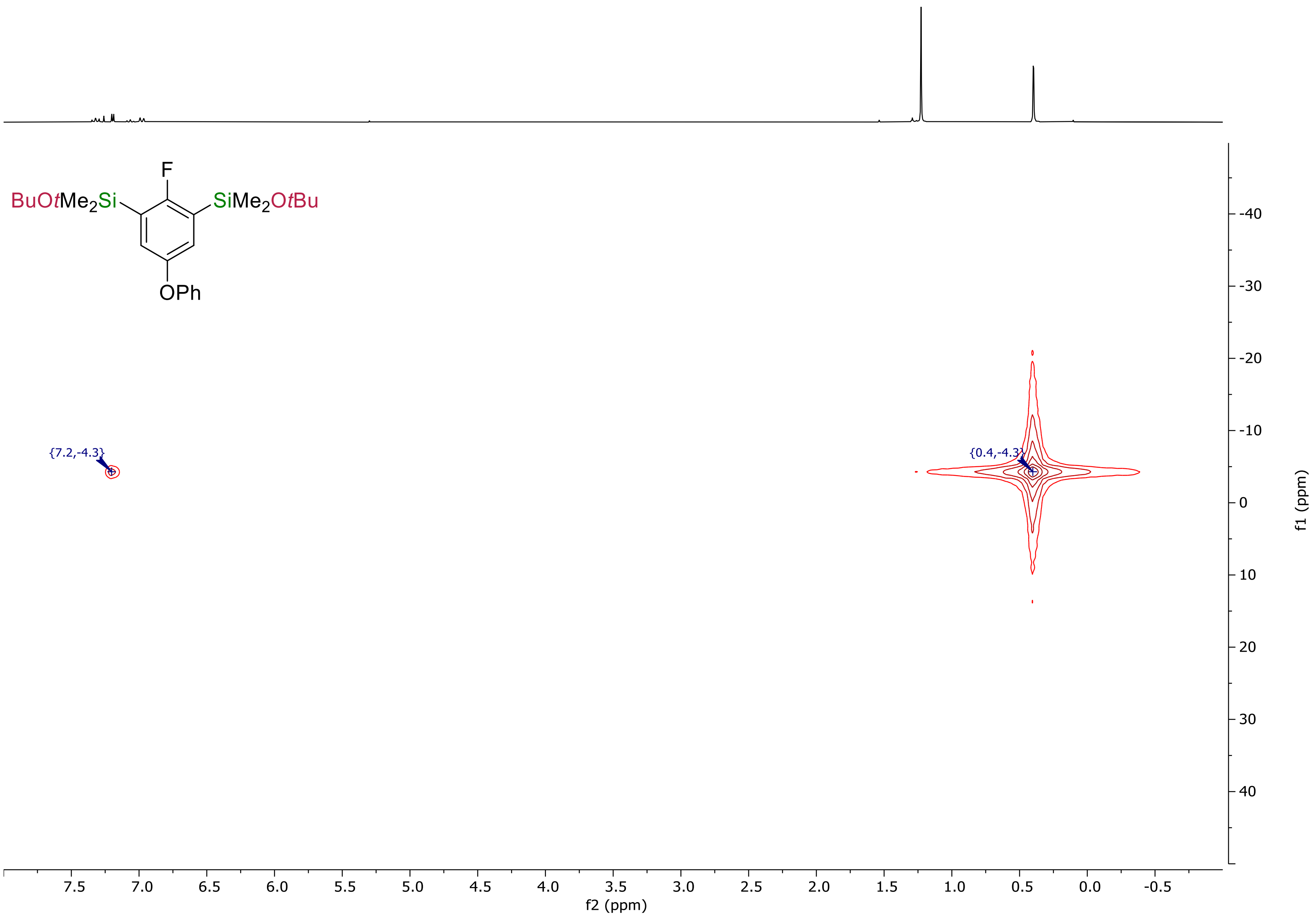

Figure S89: $^{1}H/^{29}Si$ HMQC NMR (300/60 MHz, $CDCl_3$) of compound **20a₂**.

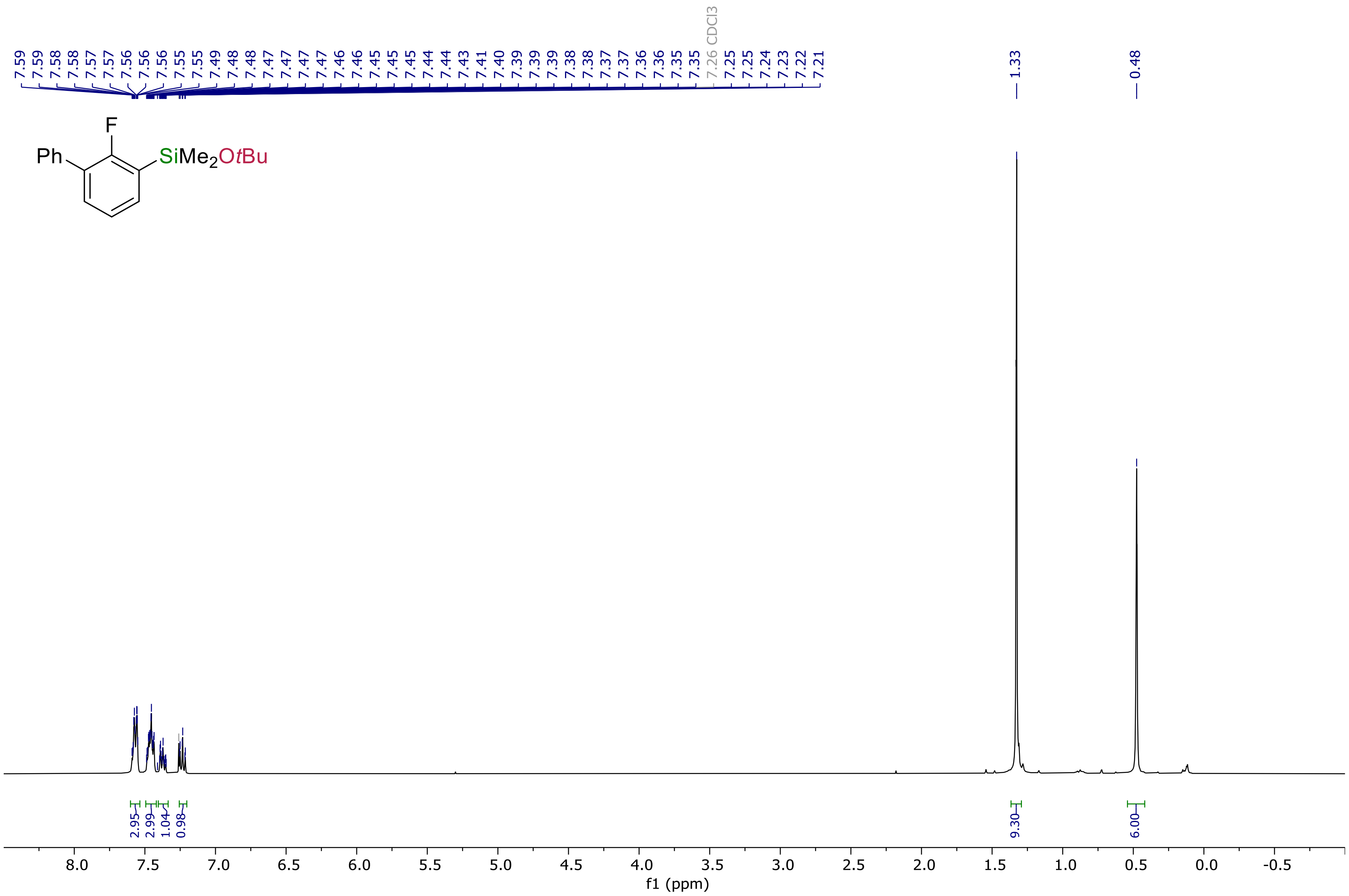

Figure S90: ^1H NMR (300 MHz, $CDCl_3$) of compound **21a**.

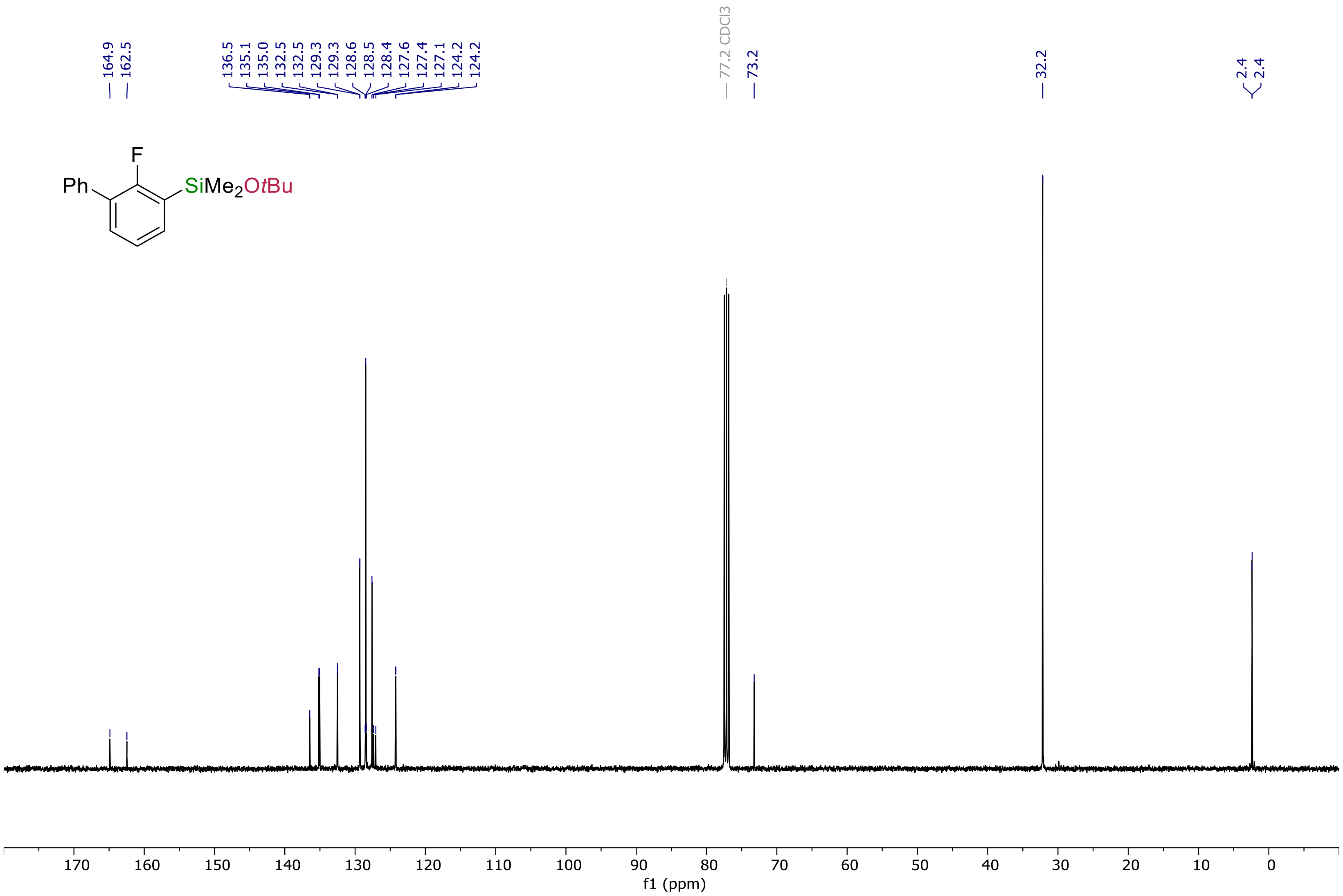

Figure S91: $^{13}C\{^{1}H\}$ NMR (75 MHz, $CDCl_3$) of compound 2**1a**.

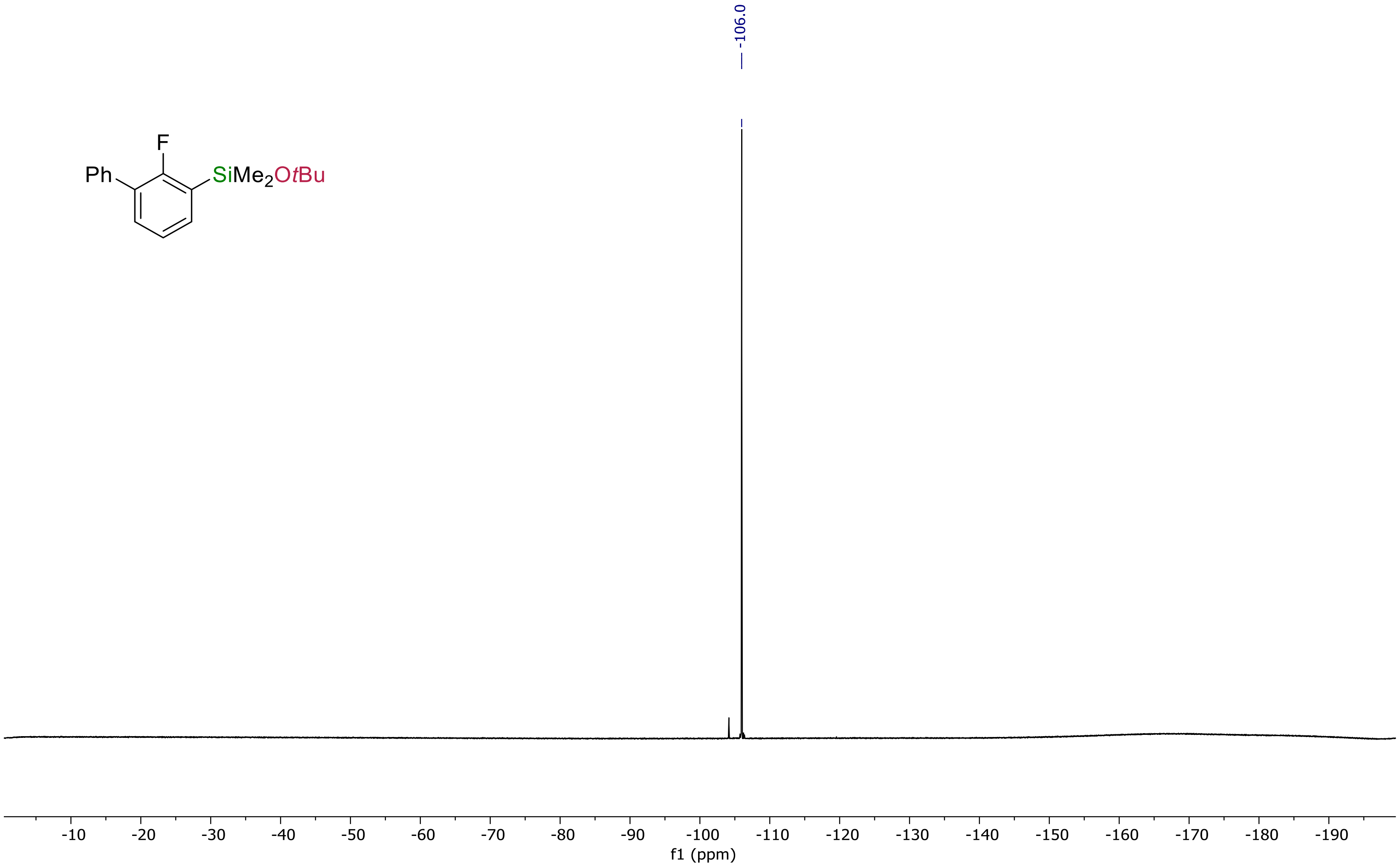

Figure S92: $^{19}F\{^{1}H\}$ NMR (282 MHz, $CDCl_3$) of compound **21a**.

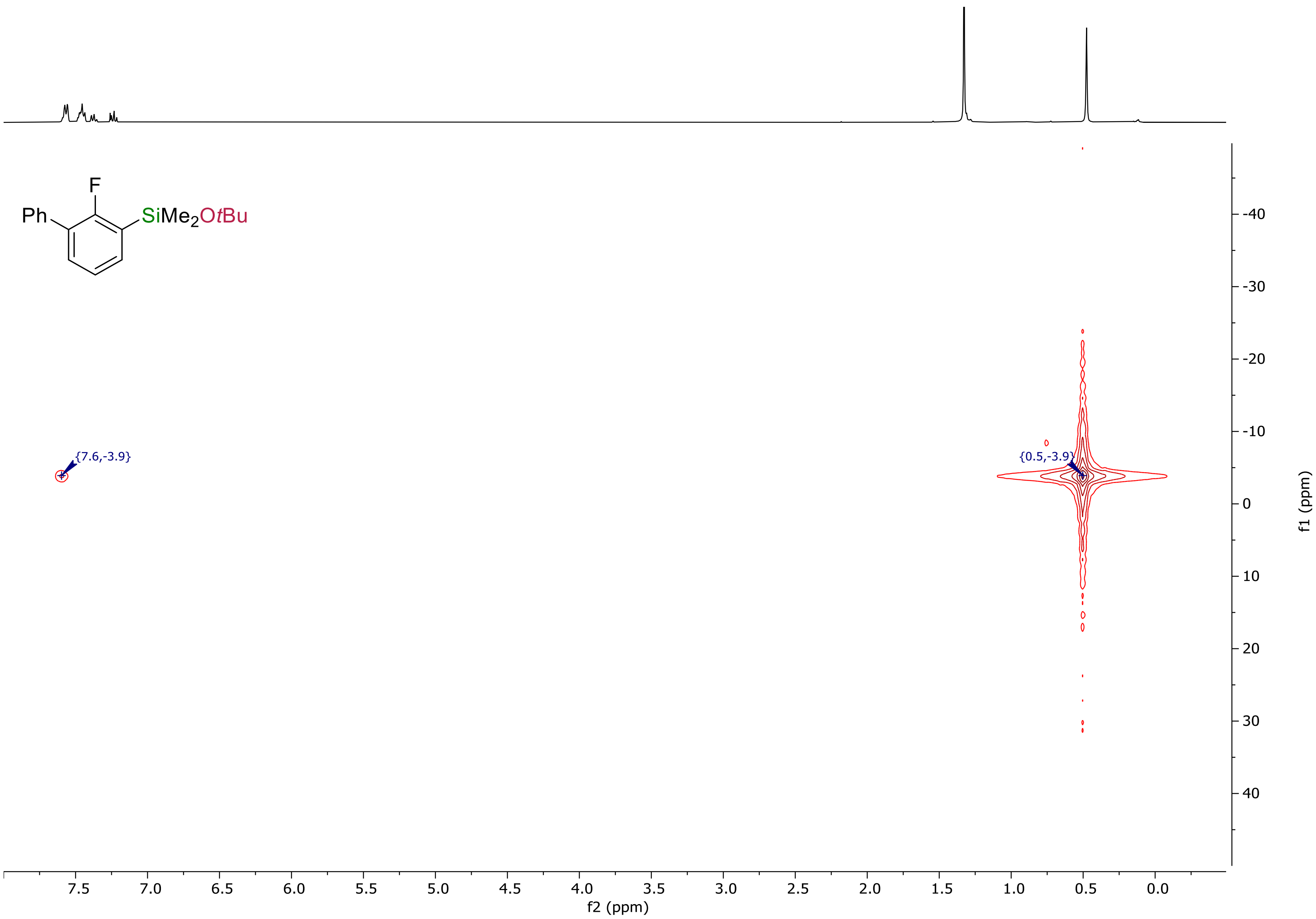

Figure S93: $^{1}H/^{29}Si$ HMQC NMR (300/60 MHz, $CDCl_3$) of compound **21a**.

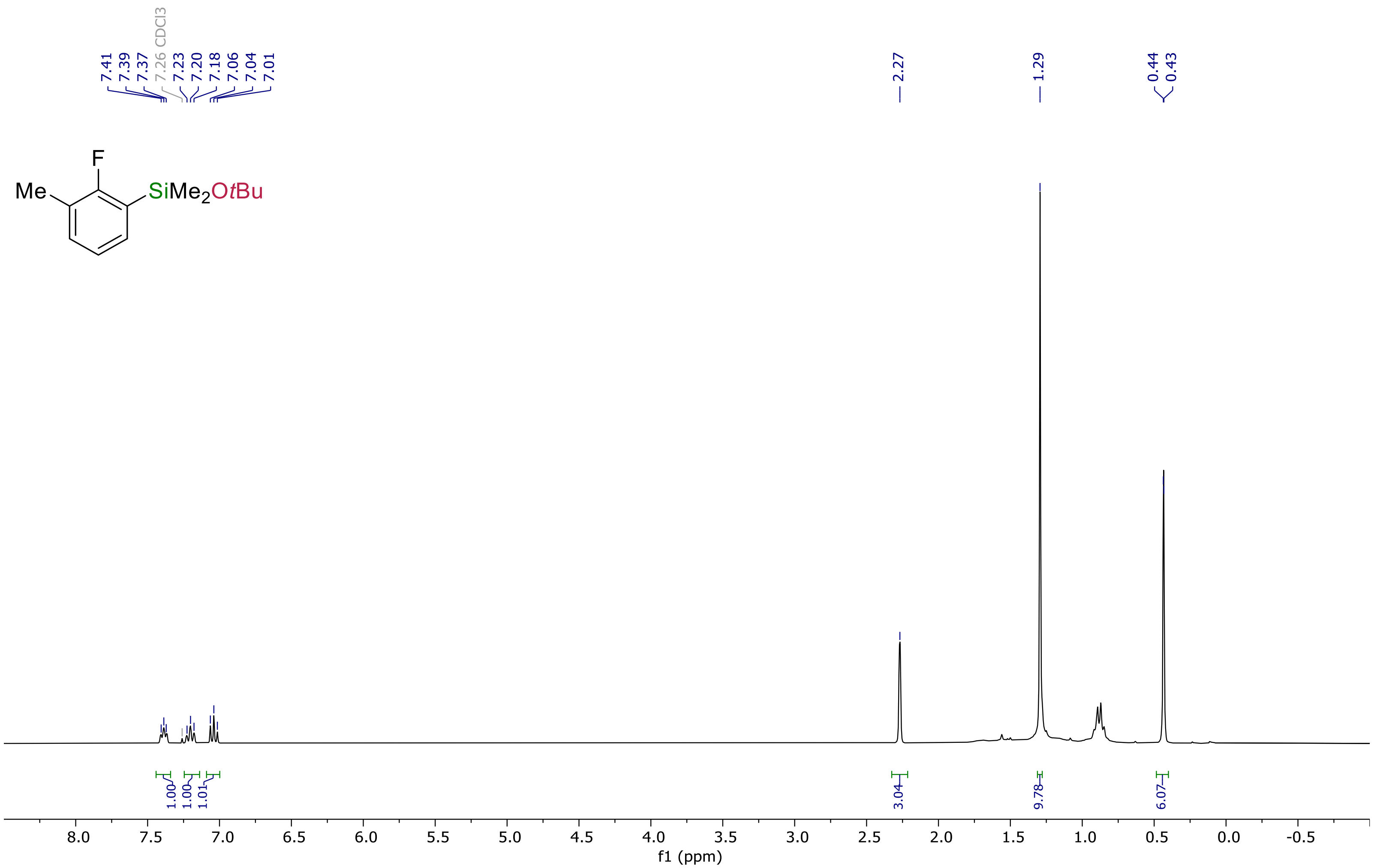

Figure S 94: ^{1}H NMR (300 MHz, $CDCl_3$) of compound **22a**.

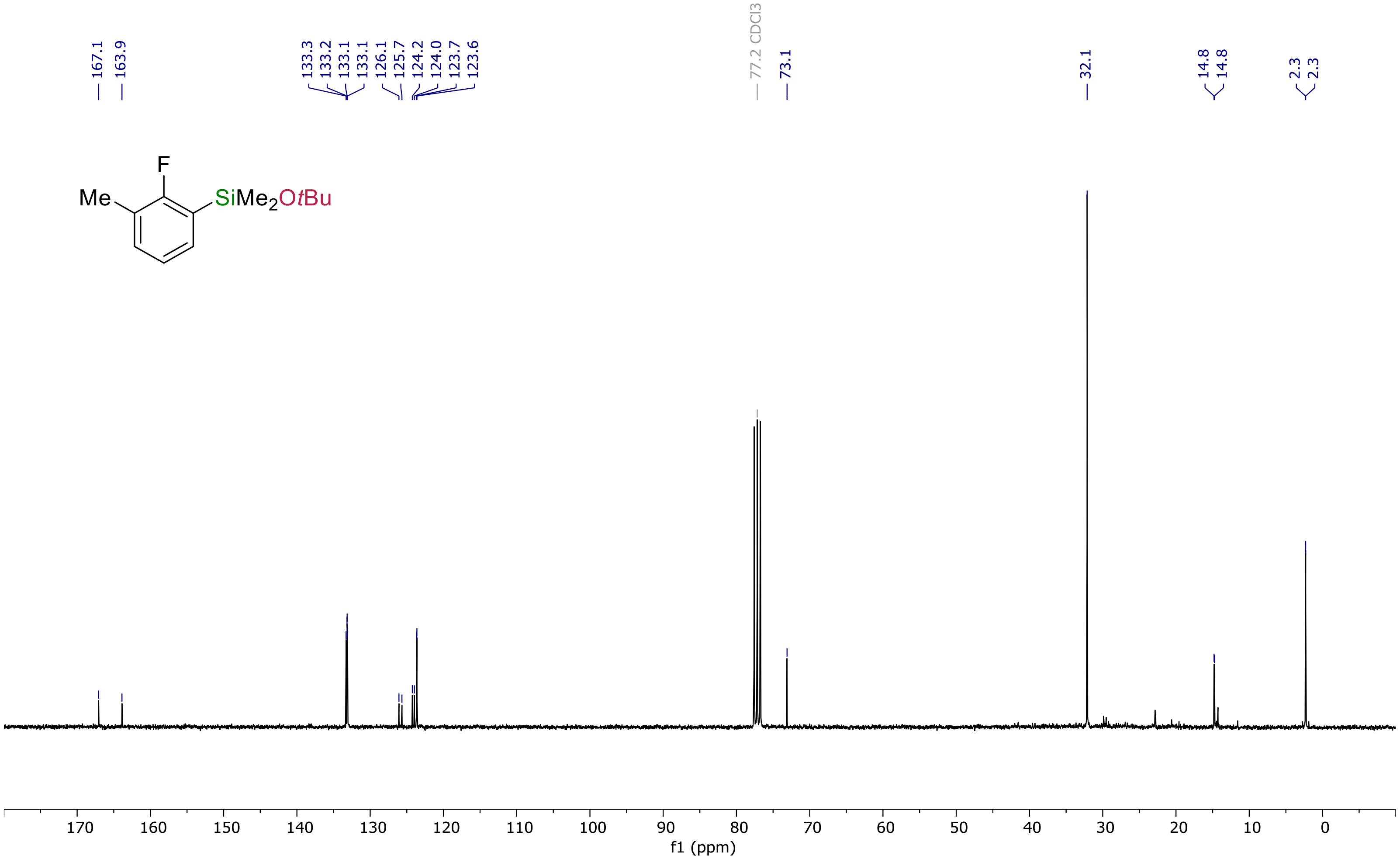

Figure S95: $^{13}C\{^{1}H\}$ NMR (75 MHz, $CDCl_3$) of compound 2**2a**.

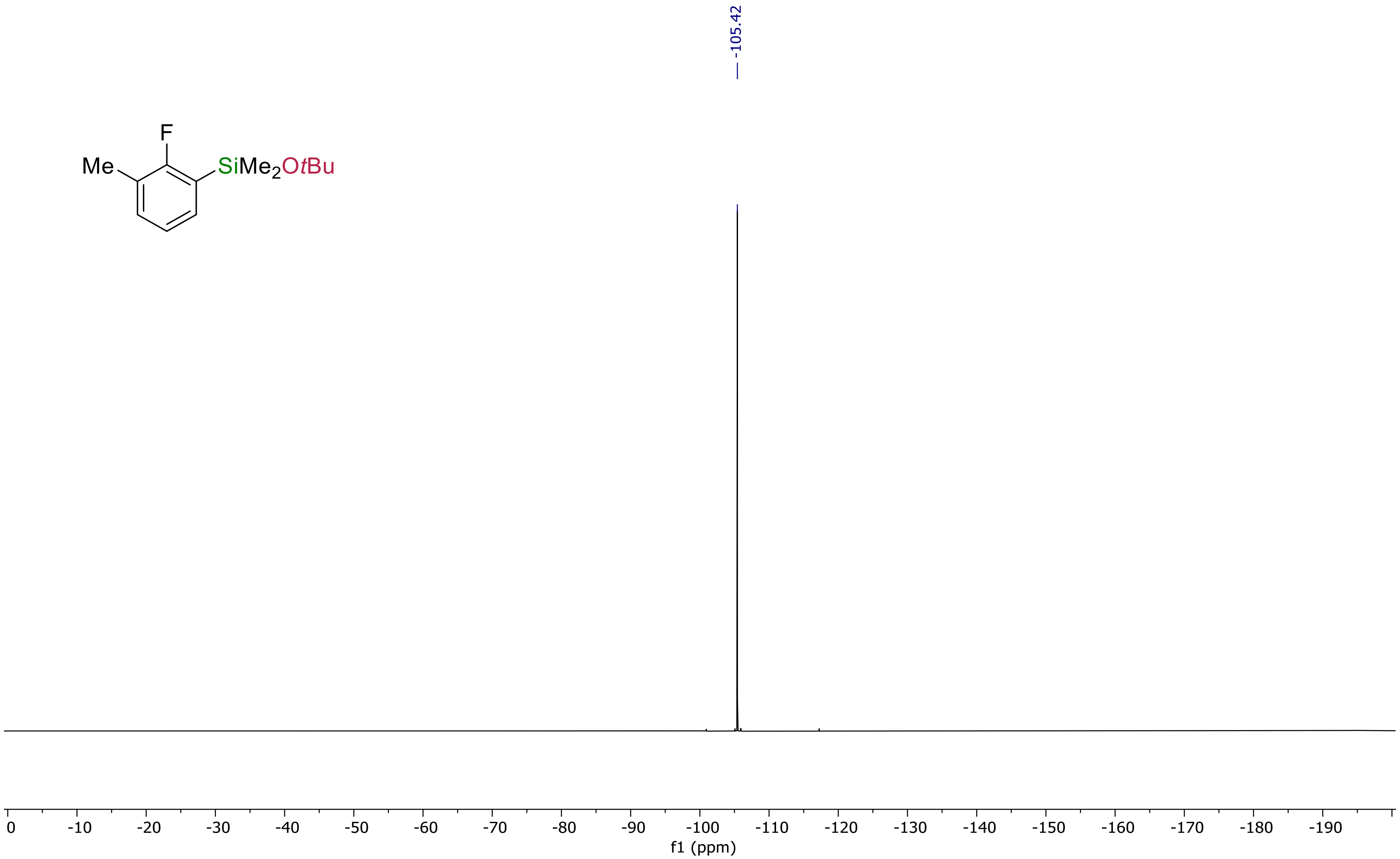

Figure S96: $^{19}F\{^1H\}$ NMR (282 MHz, $CDCl_3$) of compound **22a**.

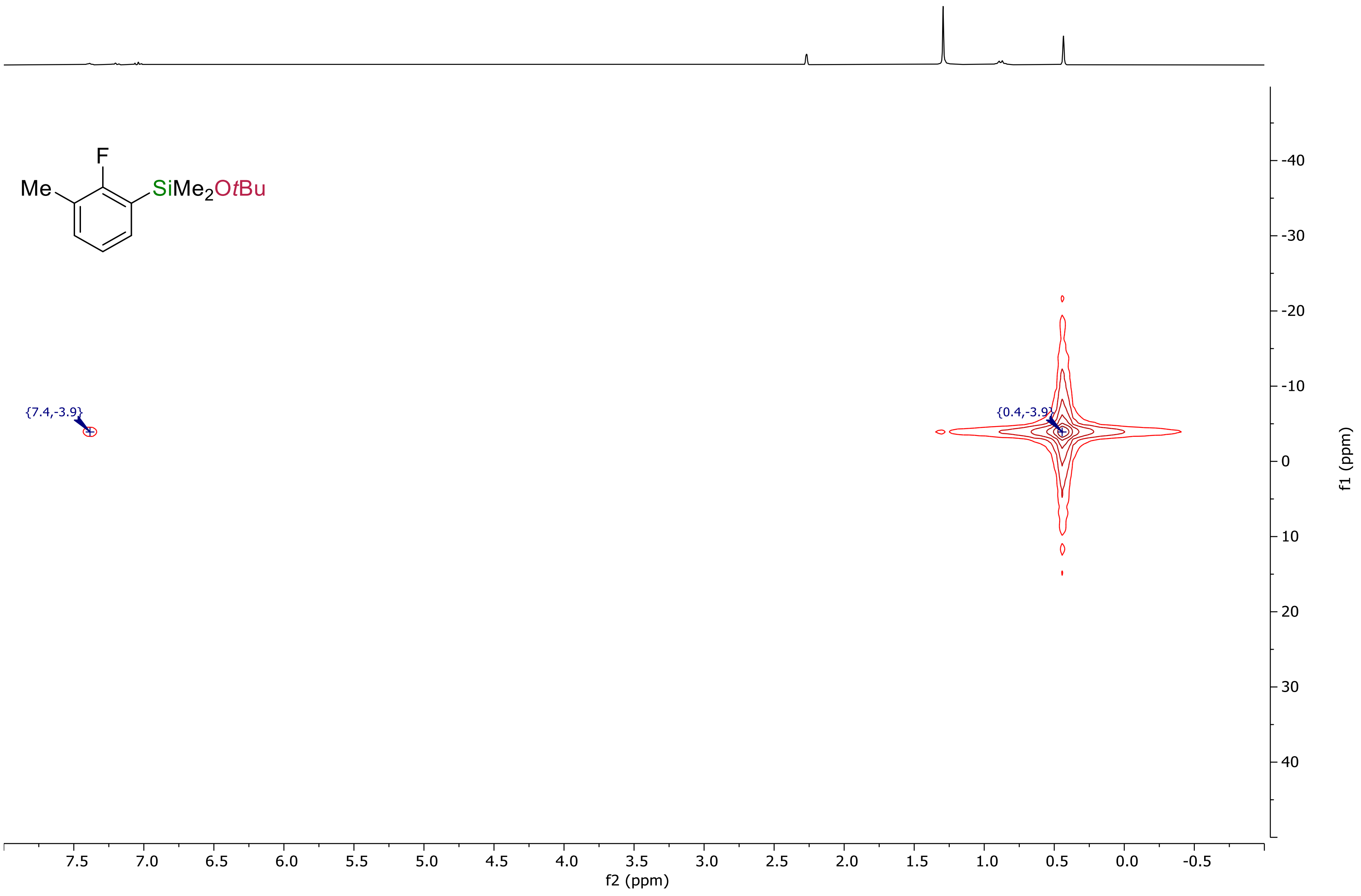

Figure S97: $^{1}H/^{29}Si$ HMQC NMR (300/60 MHz, $CDCl_3$) of compound **22a**.

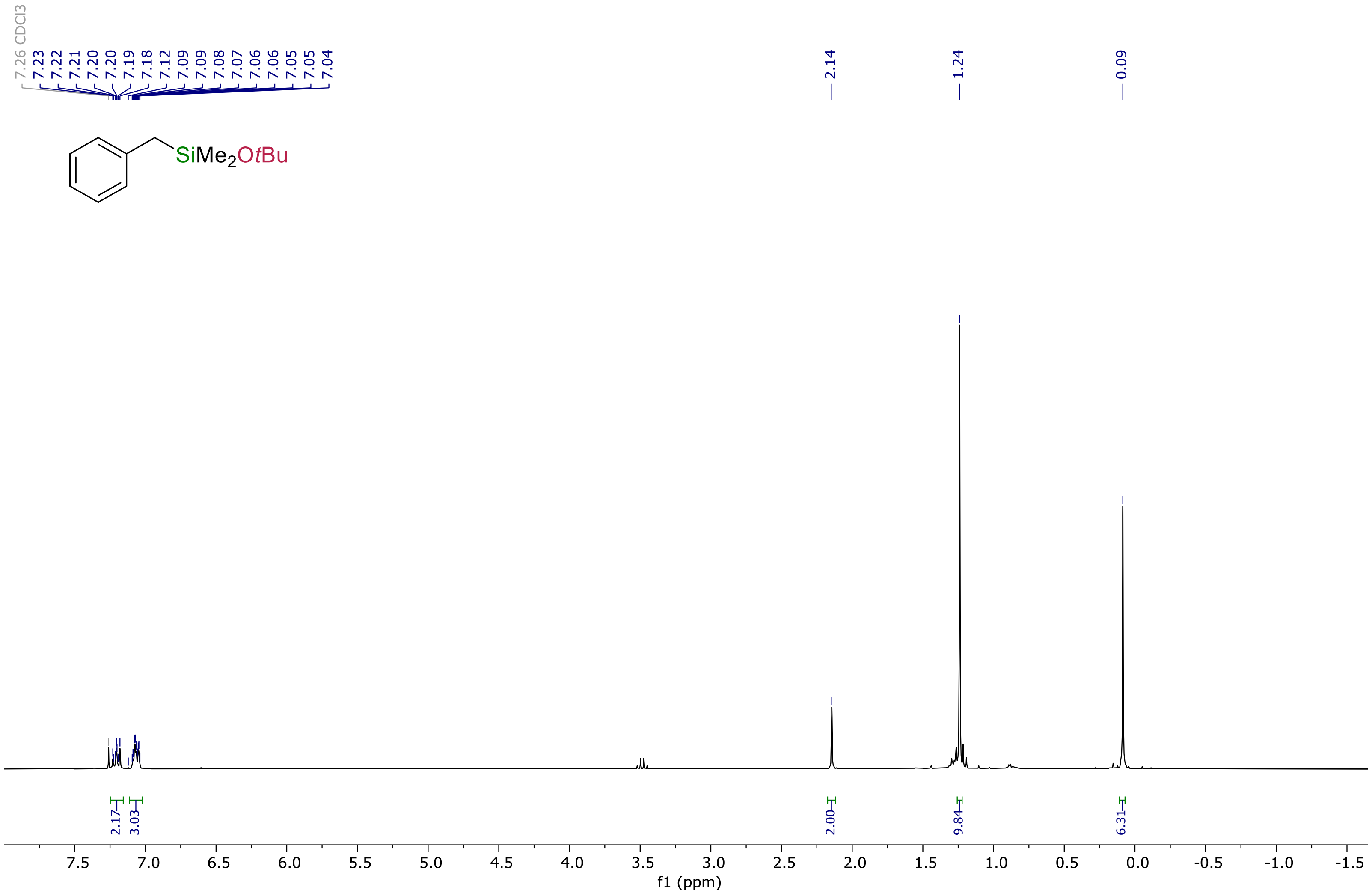

Figure S98: ^{1}H NMR (300 MHz, $CDCl_3$) of compound **23a**.

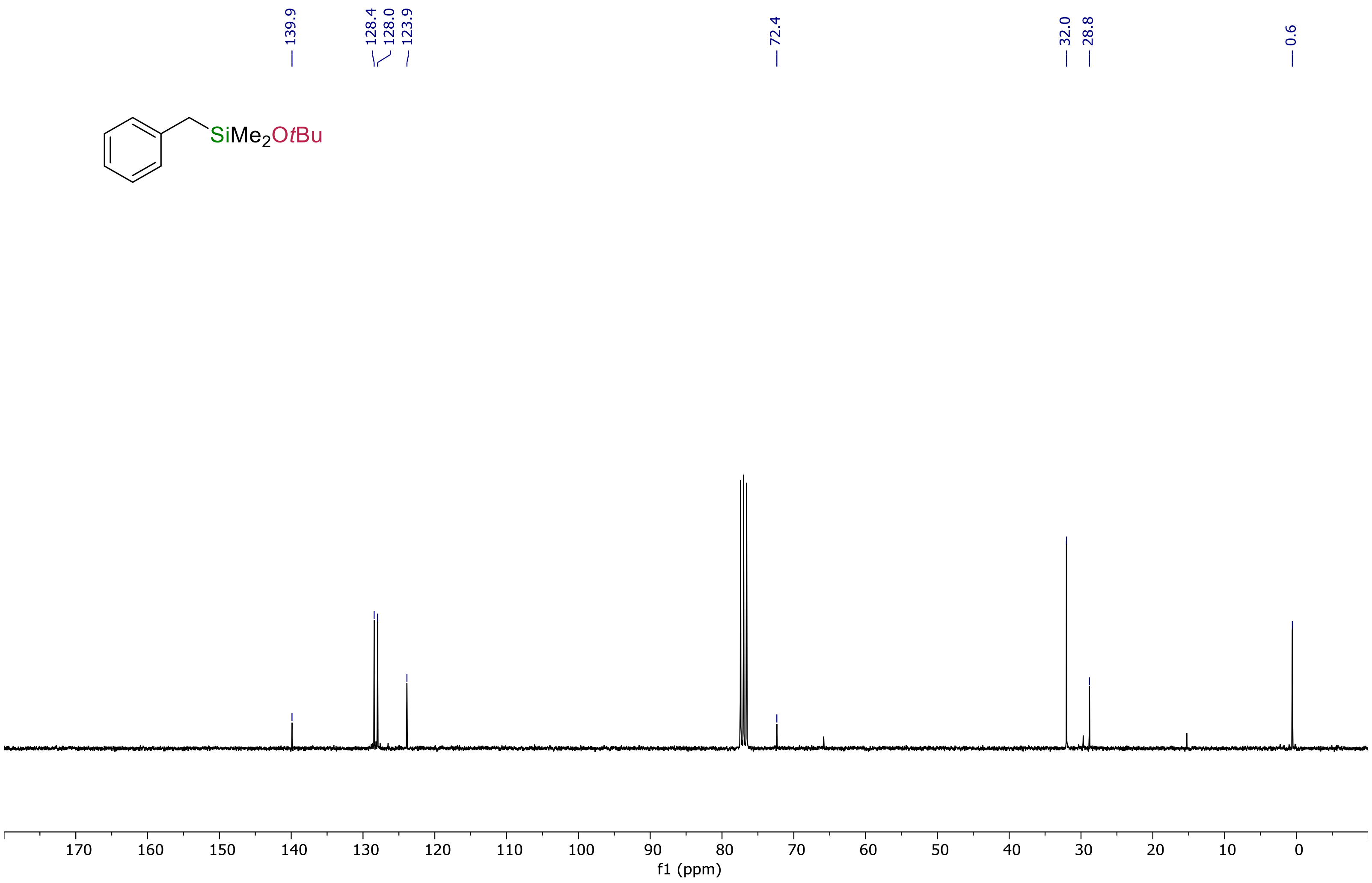

Figure S 99: $^{13}C\{^{1}H\}$ NMR (75 MHz, $CDCl_3$) of compound **23a**.

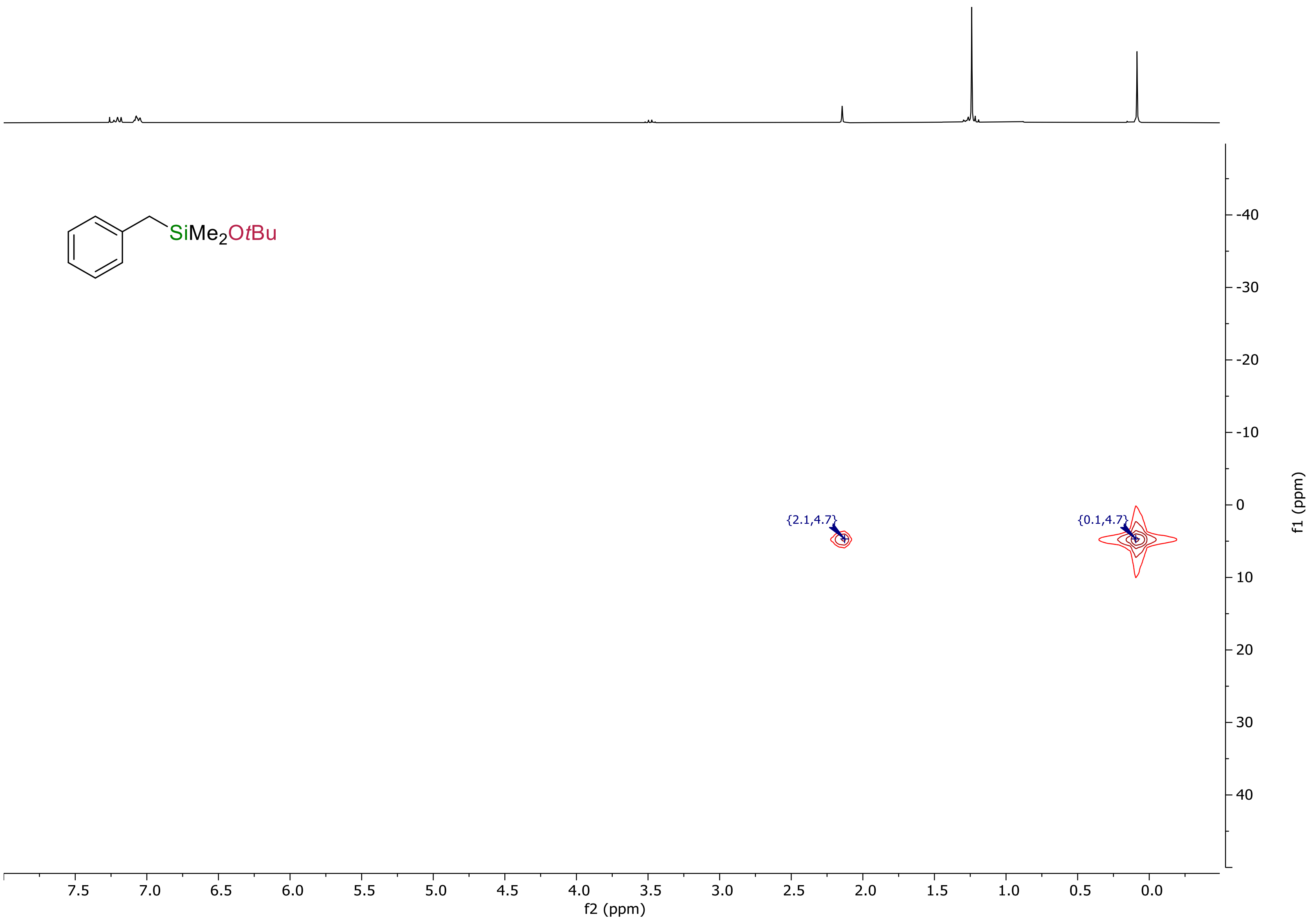

Figure S 100: $^{1}H/^{29}Si$ HMQC NMR (300/60 MHz, $CDCl_3$) of compound **23a**.

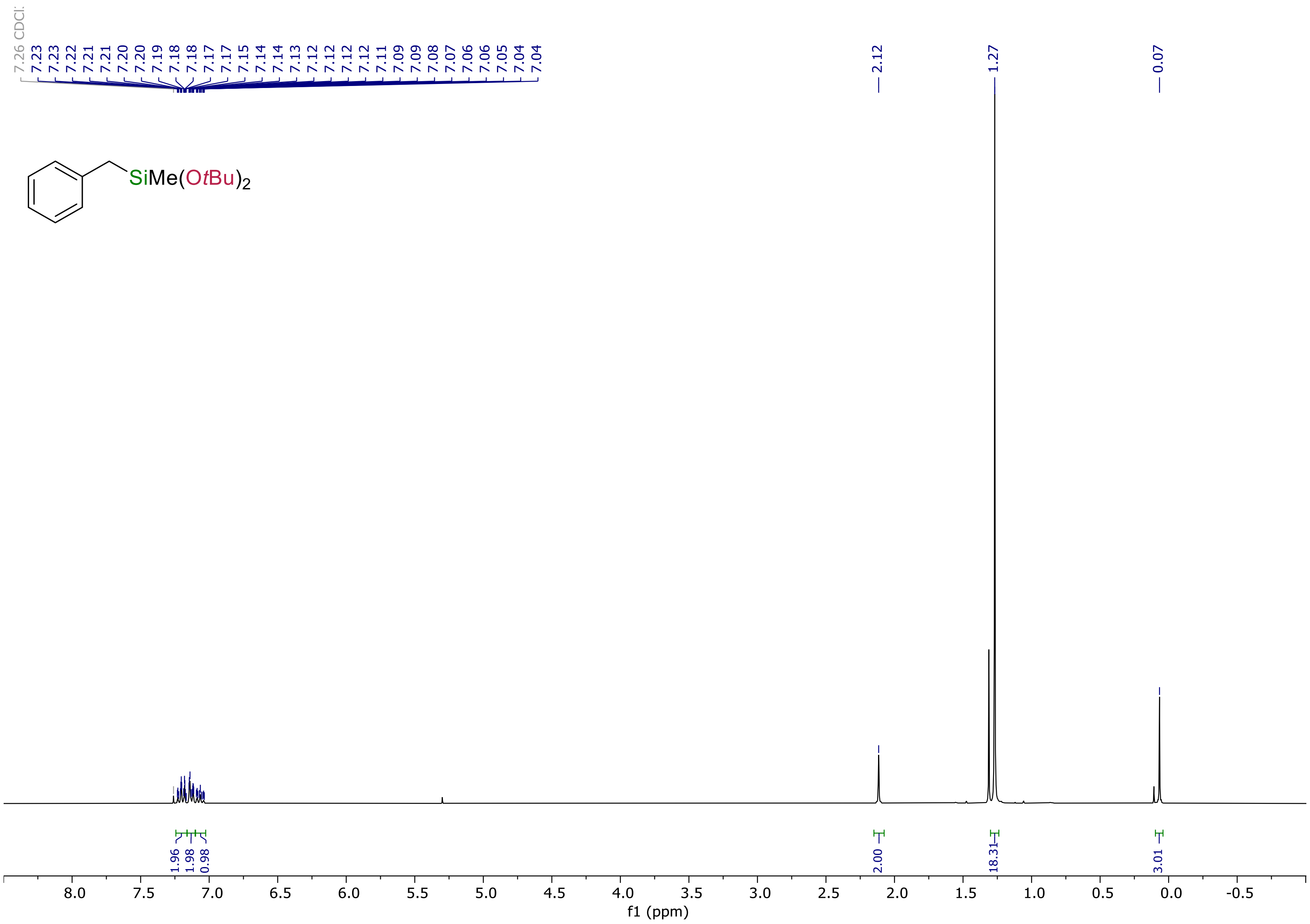

Figure S101: ^{1}H NMR (300 MHz, $CDCl_3$) of compound **23d**.

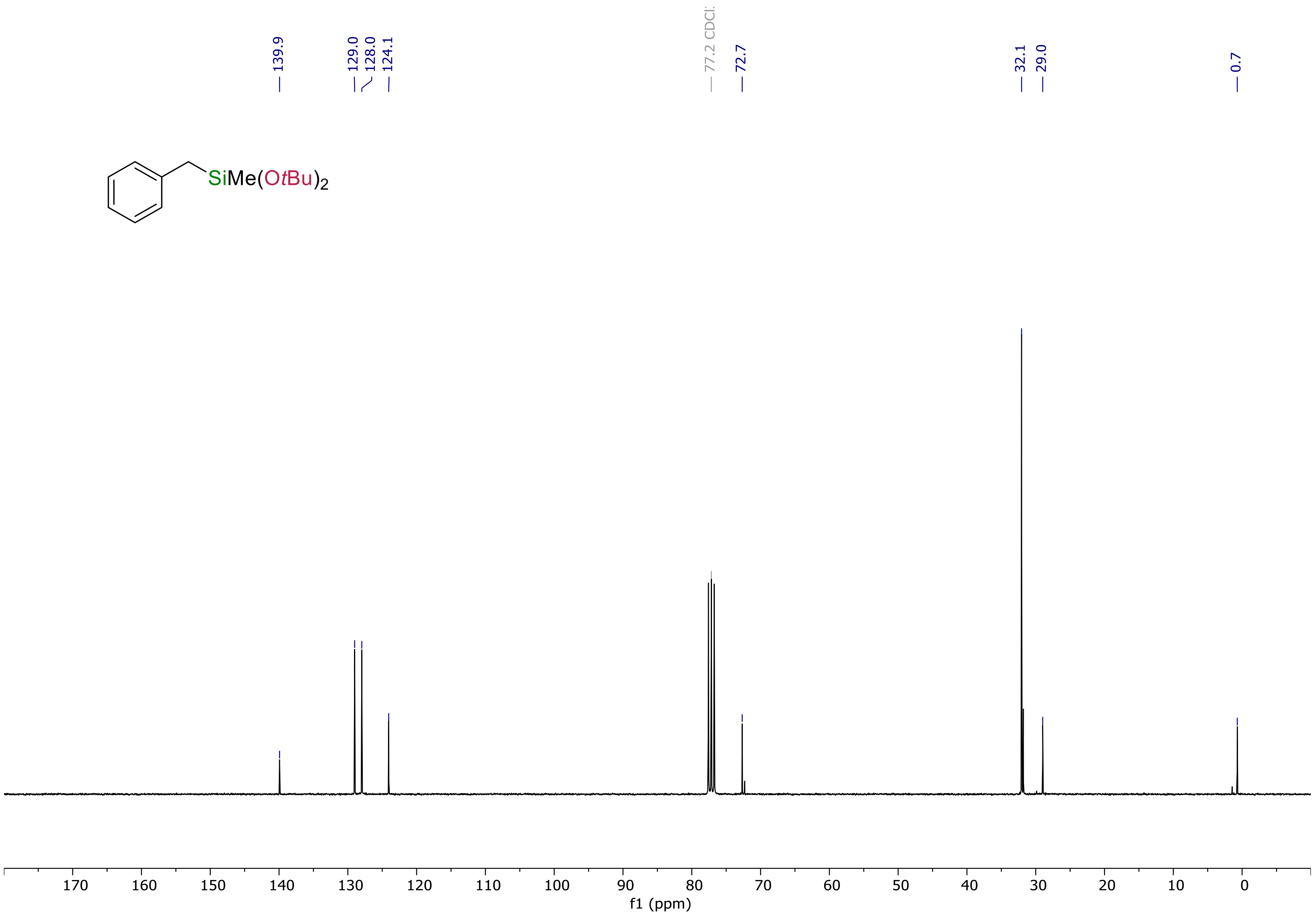

Figure S102: $^{13}C\{^{1}H\}$ NMR (75 MHz, $CDCl_3$) of compound **23d**.

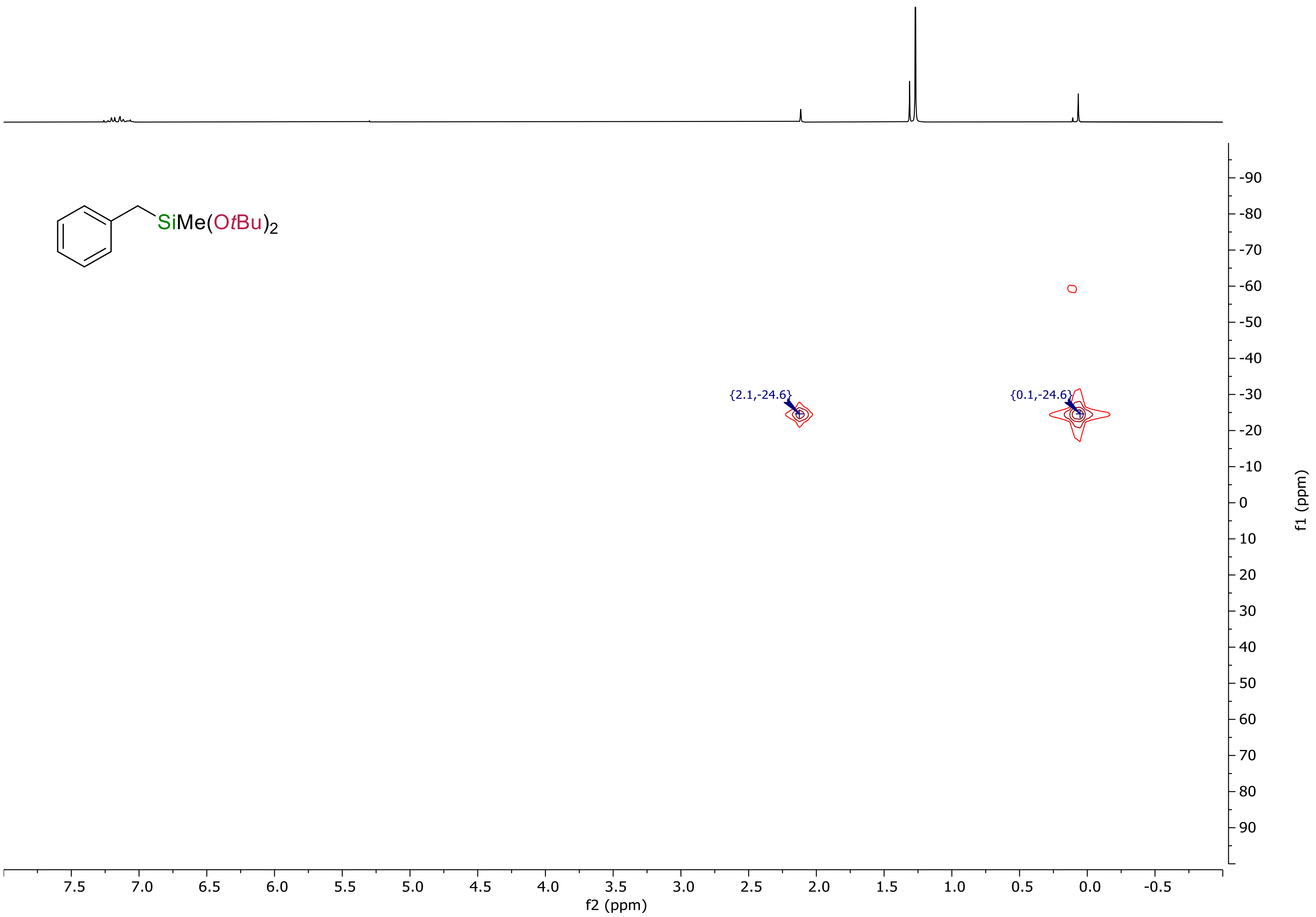

Figure S103: $^{1}H/^{29}Si$ HMQC NMR (300/60 MHz, $CDCl_3$) of compound **23d**.

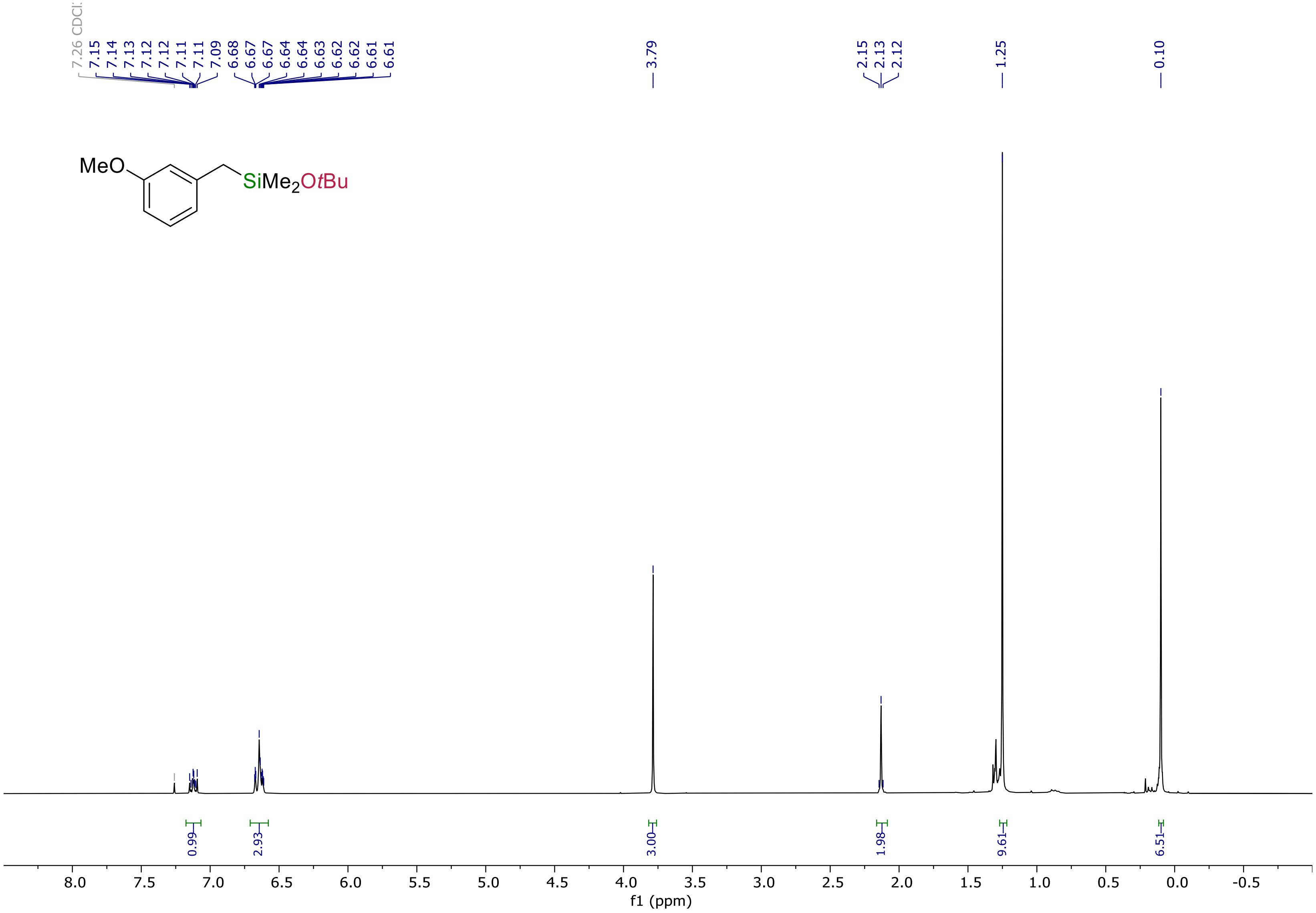

Figure S104: ^{1}H NMR (300 MHz, $CDCl_3$) of compound **24a**.

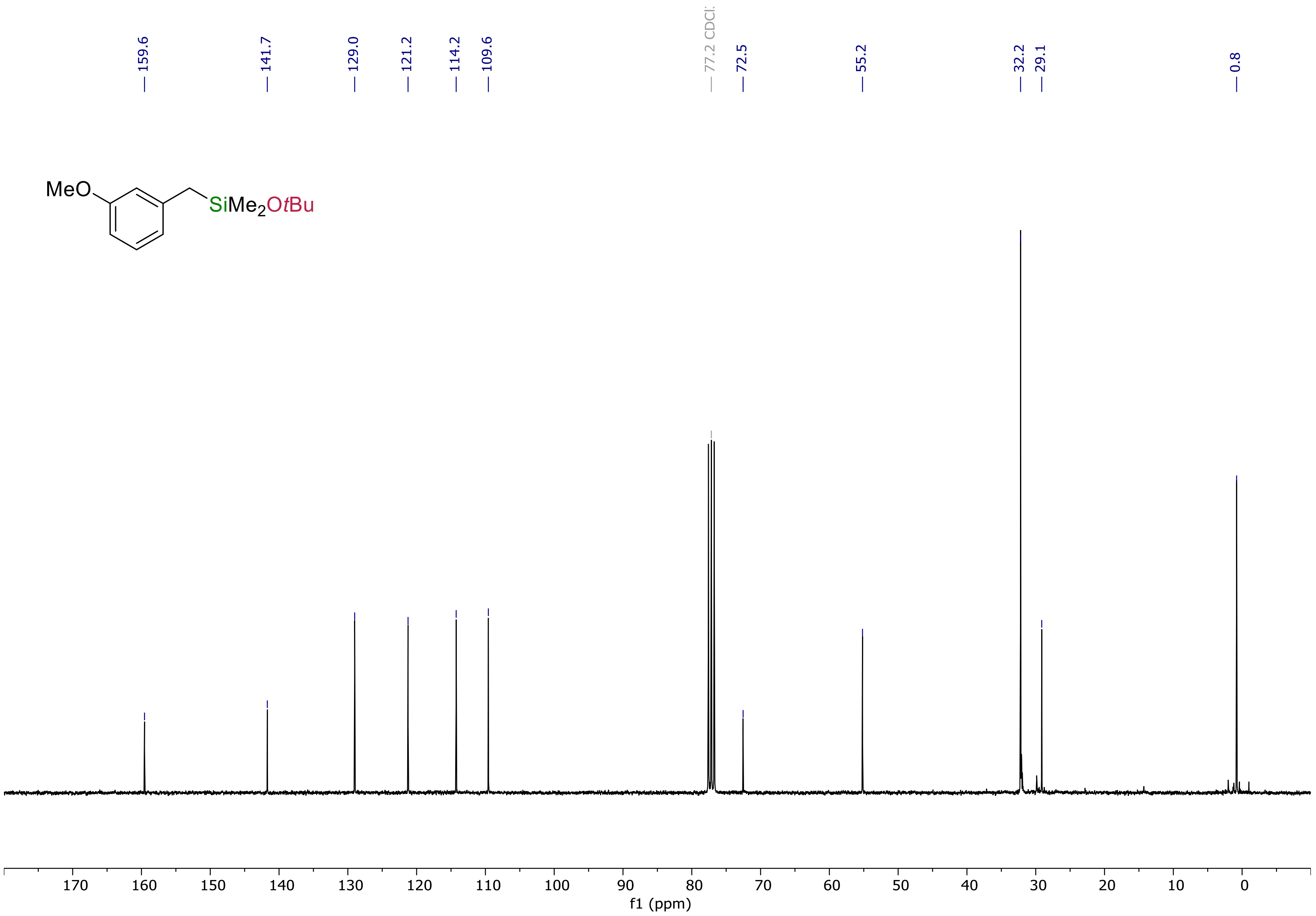

Figure S105: $^{13}C\{^{1}H\}$ NMR (75 MHz, $CDCl_3$) of compound **24a**.

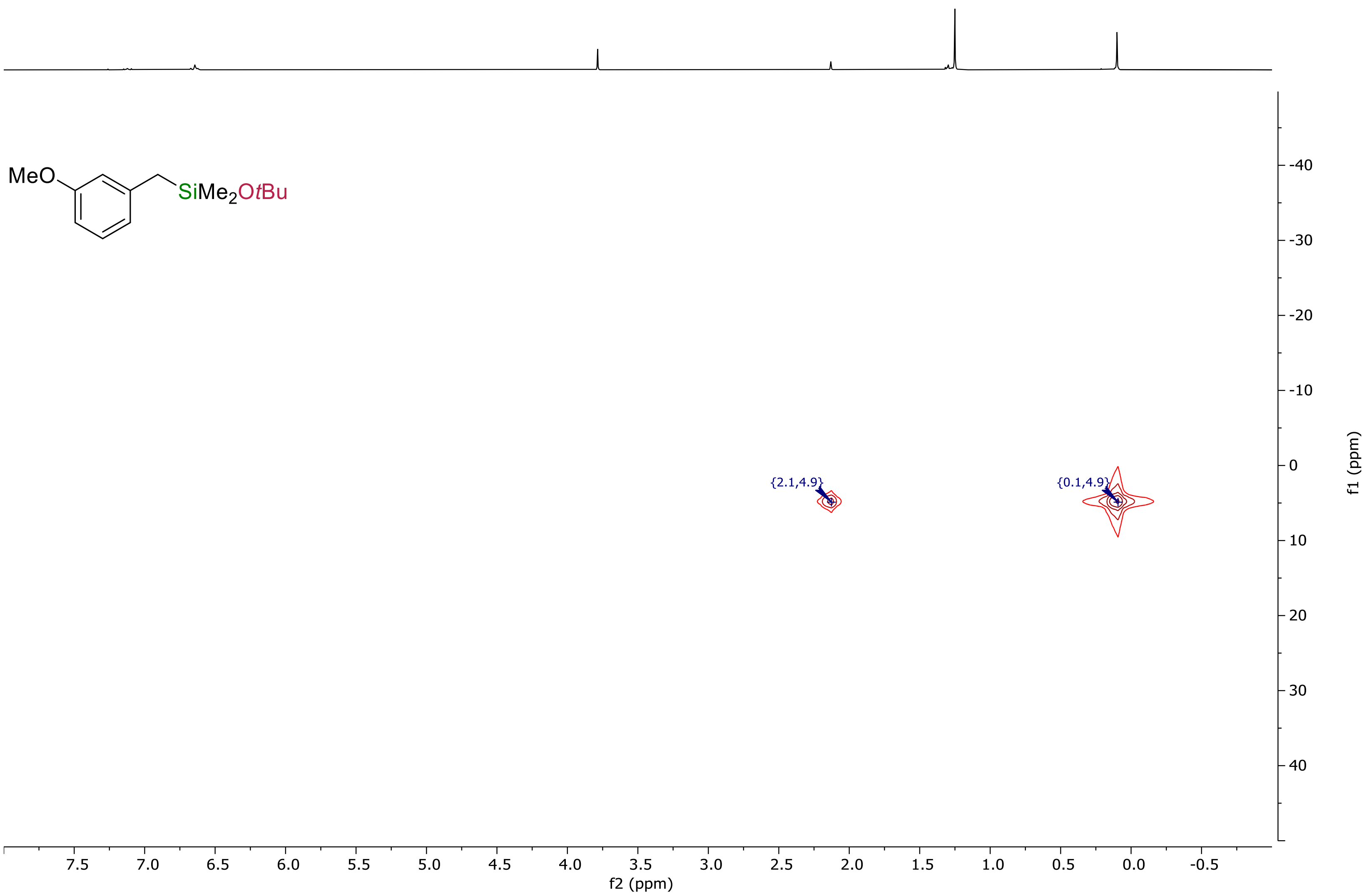

Figure S106: $^{1}H/^{29}Si$ HMQC NMR (300/60 MHz, $CDCl_3$) of compound **24a**.

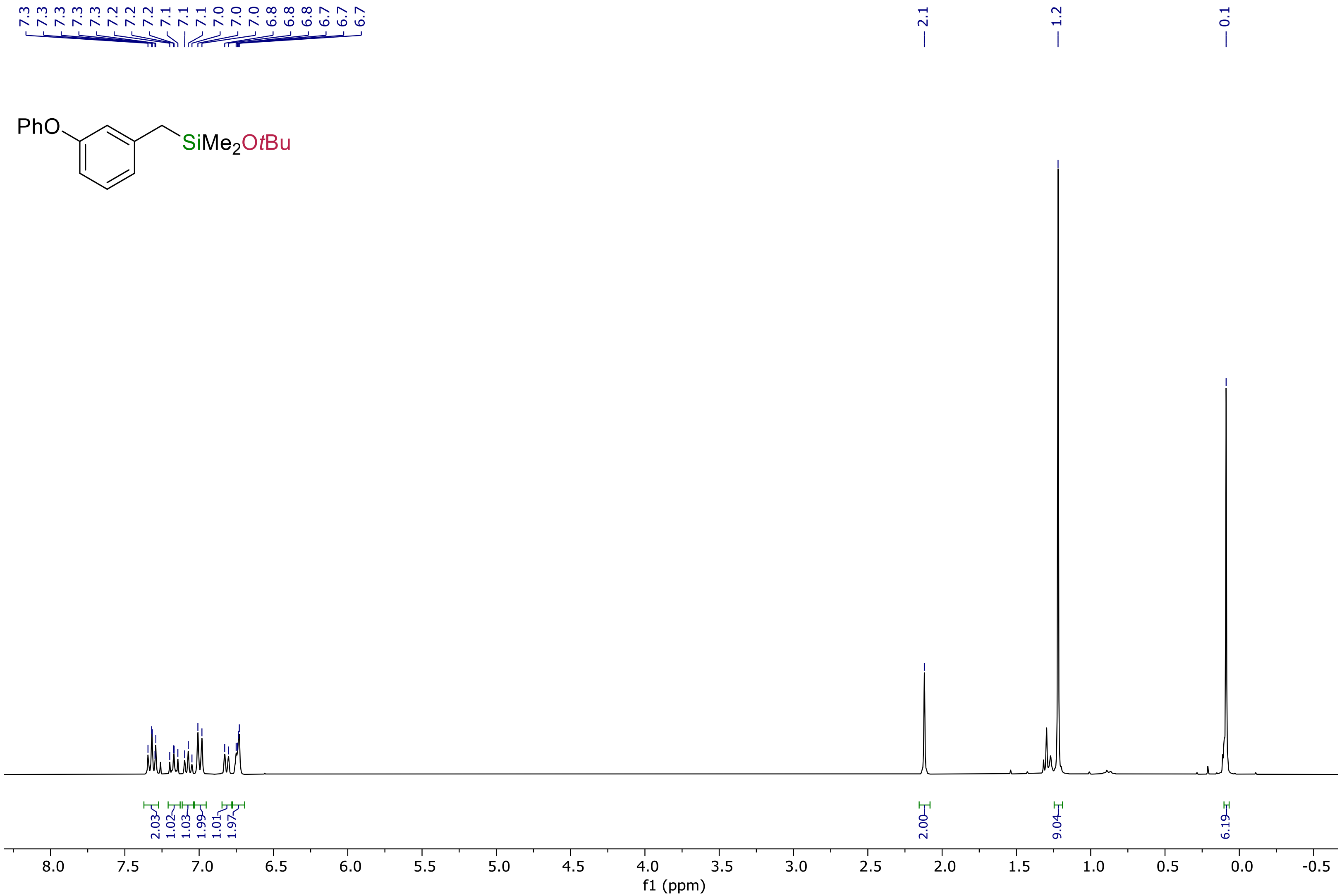

Figure S107: ^{1}H NMR (300 MHz, $CDCl_3$) of compound **26a**.

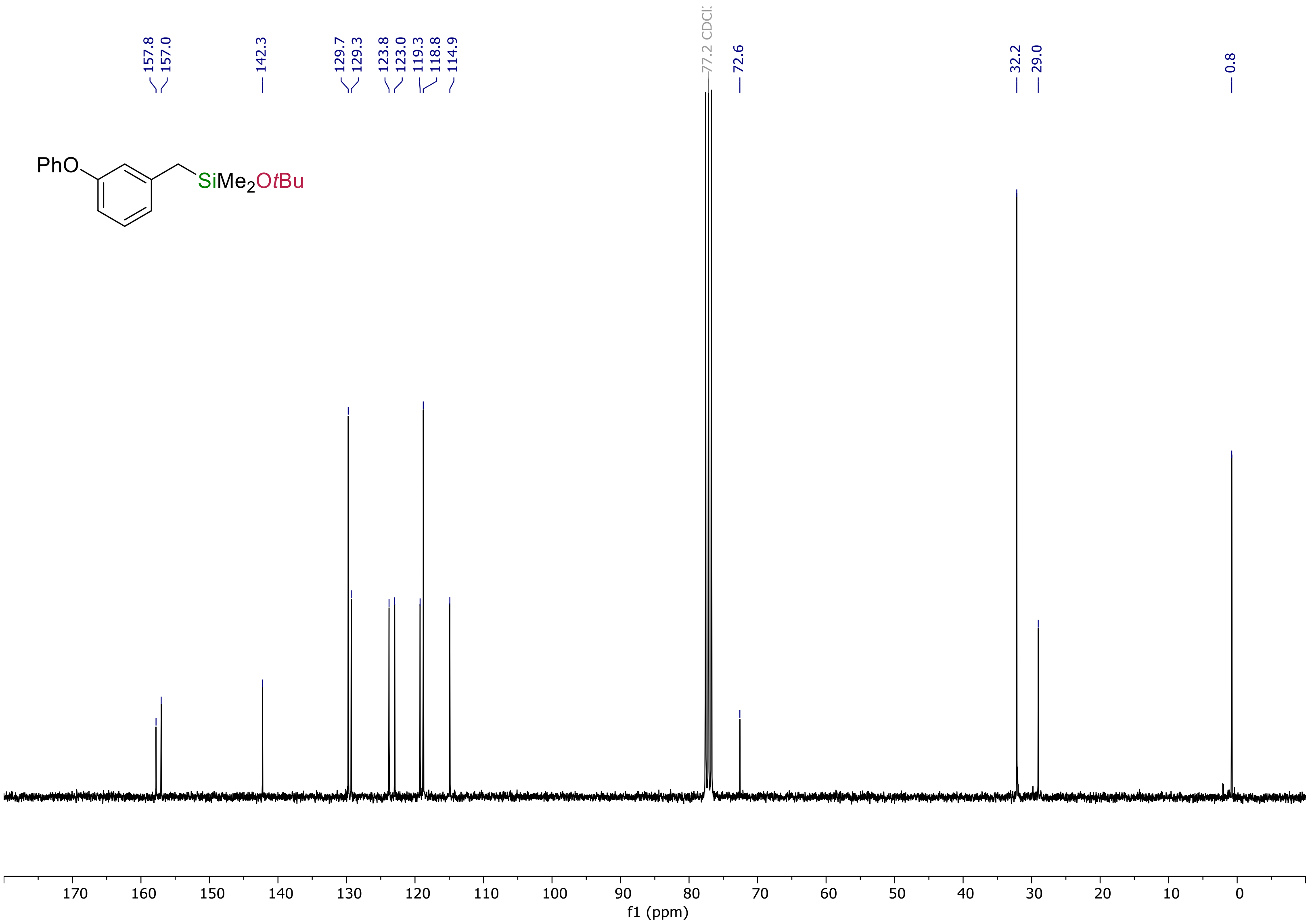

Figure S108: $^{13}C\{^{1}H\}$ NMR (75 MHz, $CDCl_3$) of compound **26a**.

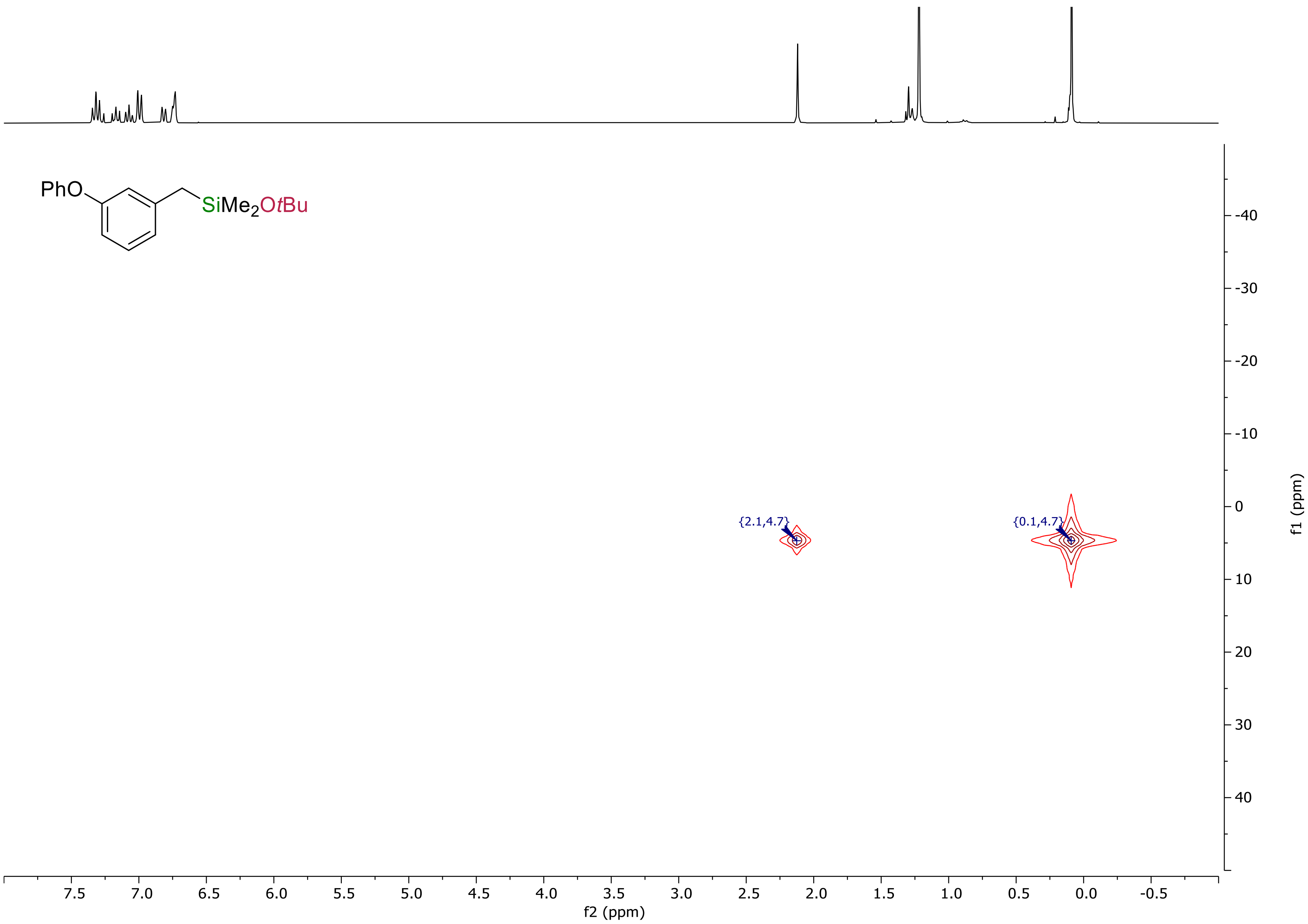

Figure S109: $^{1}H/^{29}Si$ HMQC NMR (300/60 MHz, $CDCl_3$) of compound **26a**.

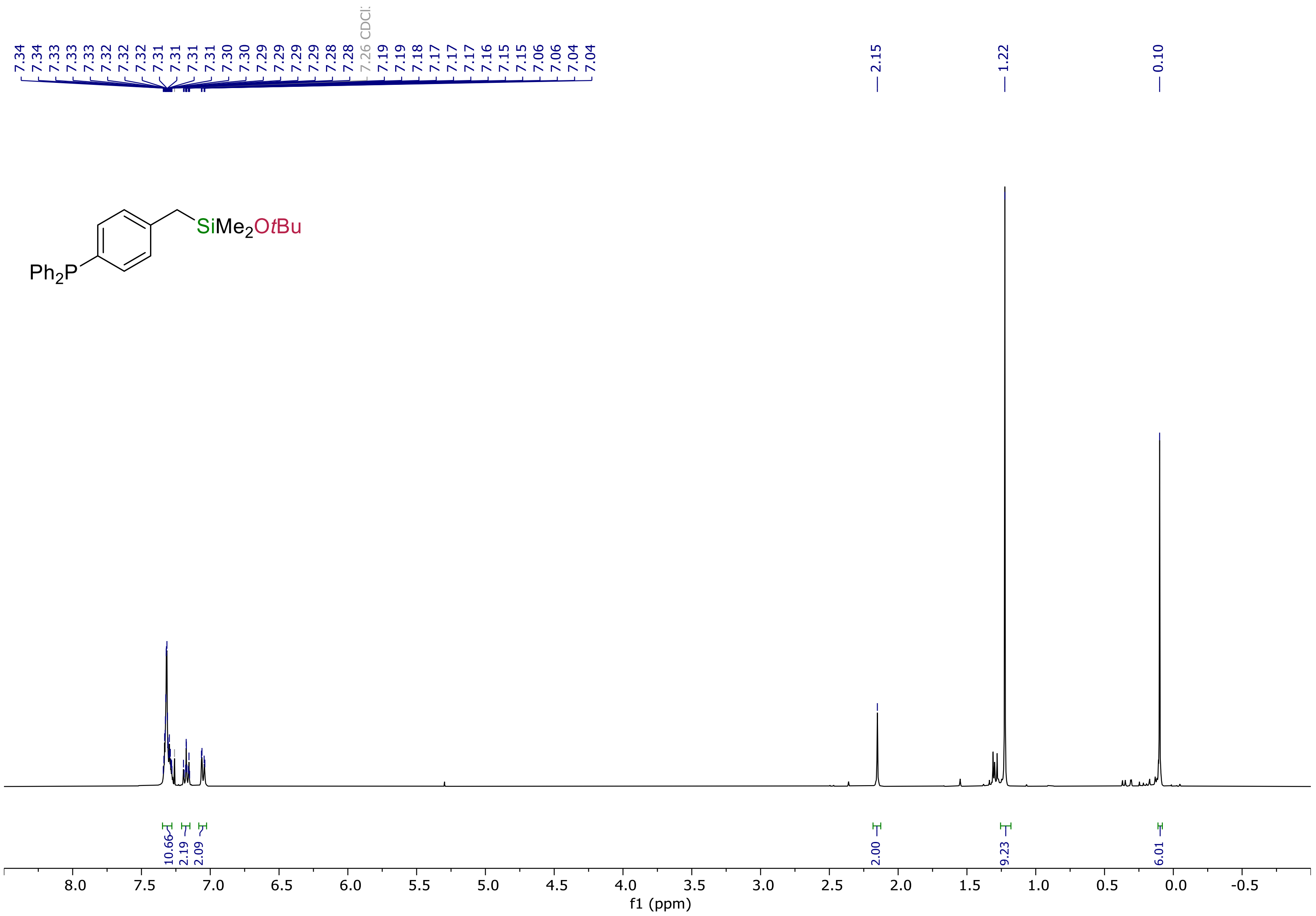

Figure S110: ^{1}H NMR (400 MHz, $CDCl_3$) of compound **28a**.

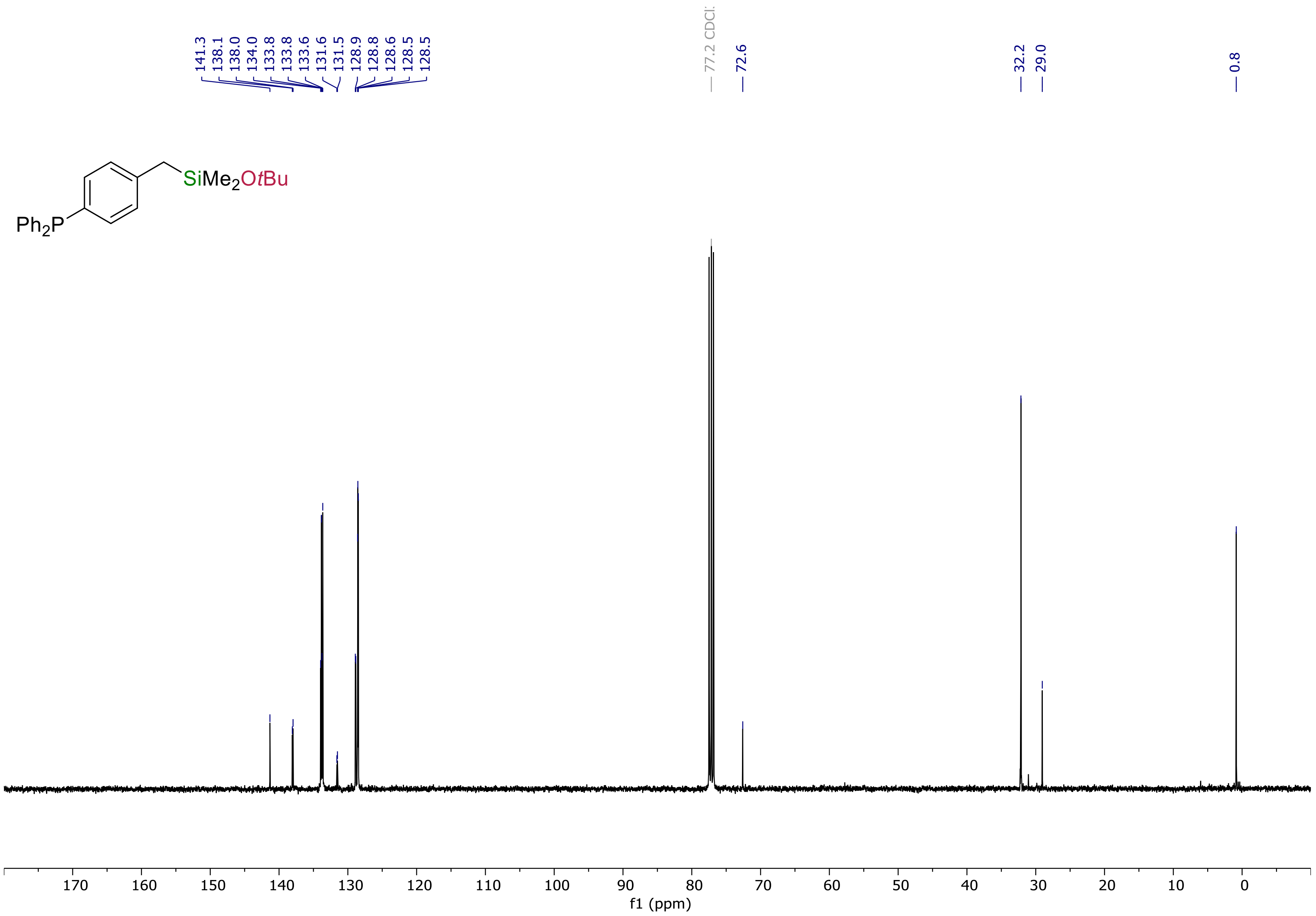

Figure S111: $^{13}C\{^1H\}$ NMR (101 MHz, $CDCl_3$) of compound **28a**.

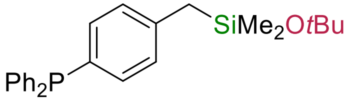

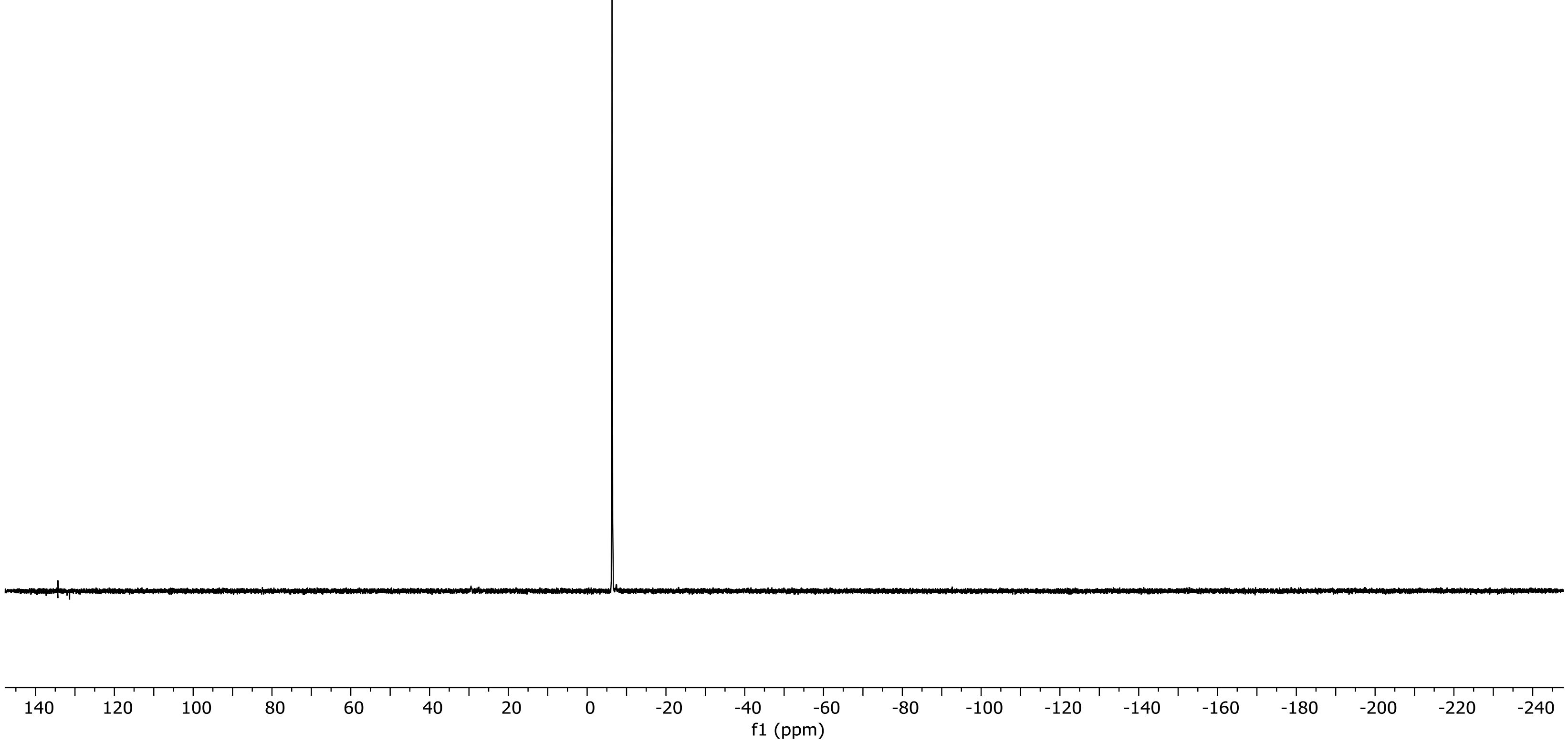

Figure S 112: $^{31}P\{^{1}H\}$ NMR (162 MHz, $CDCl_3$) of compound **28a**.

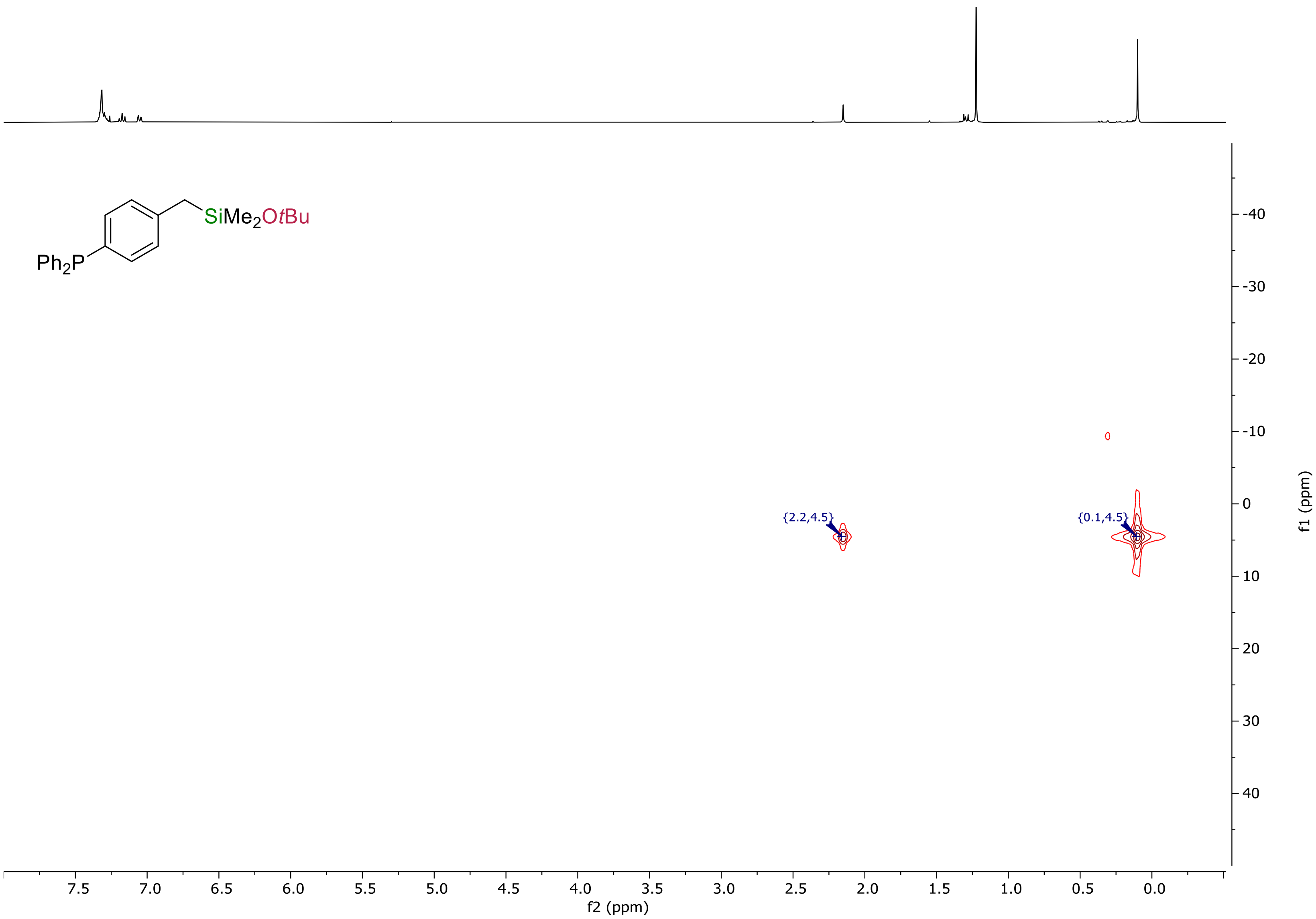

Figure S113: $^{1}H/^{29}Si$ HMQC NMR (400/79 MHz, $CDCl_3$) of compound **28a**.

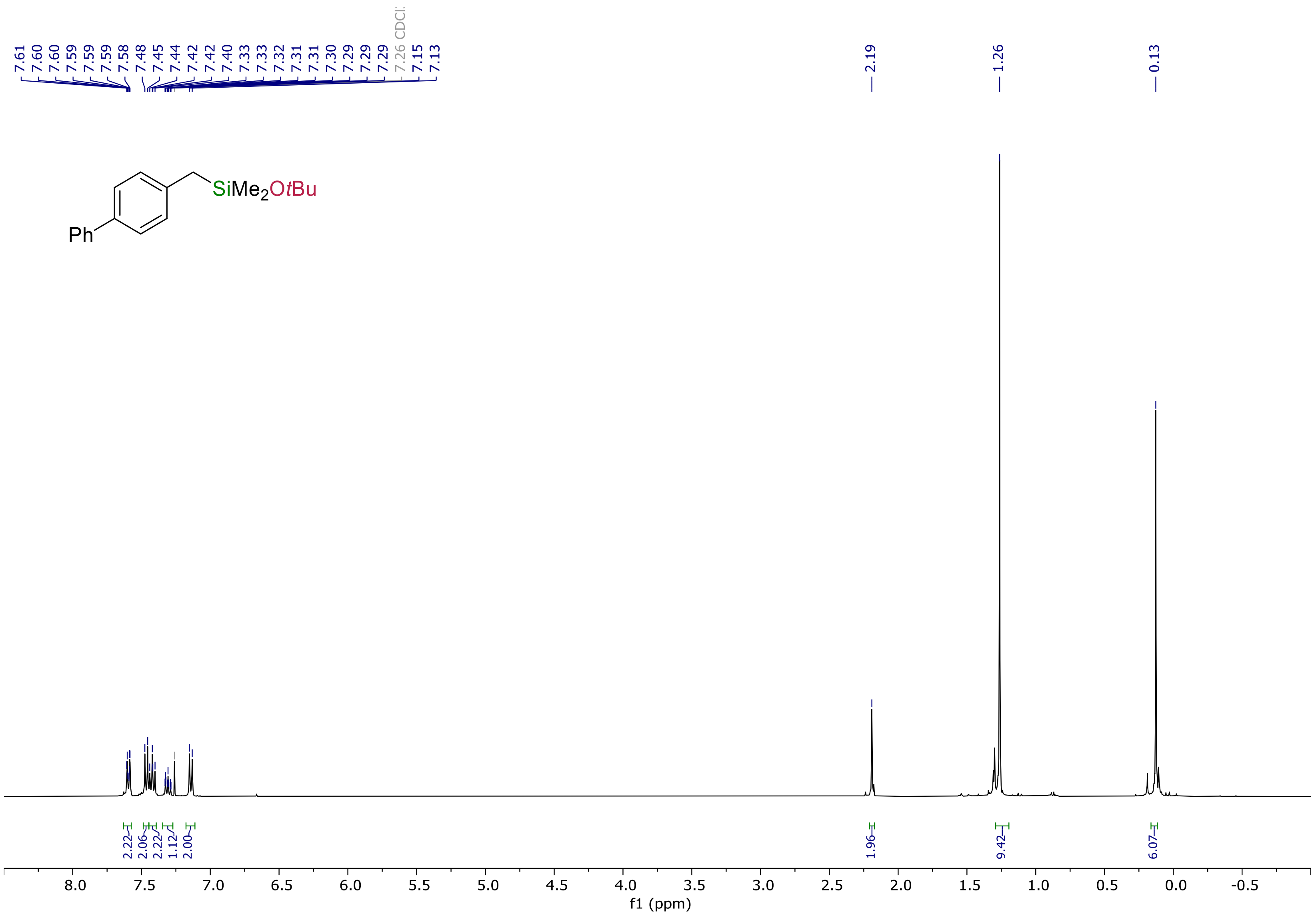

Figure S114: ^1H NMR (400 MHz, $CDCl_3$) of compound **29a**.

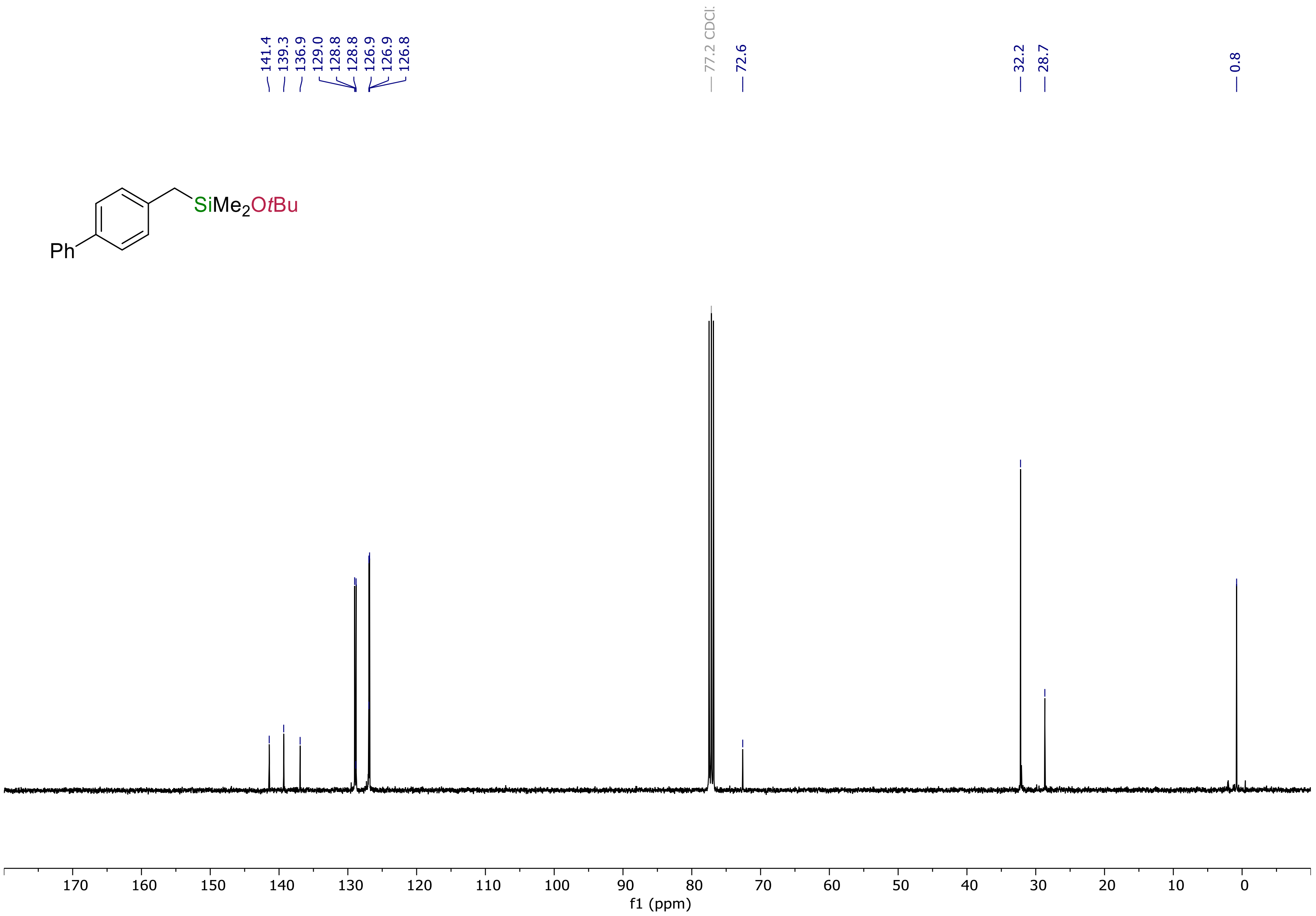

Figure S115: $^{13}C\{^{1}H\}$ NMR (101 MHz, $CDCl_3$) of compound **29a**.

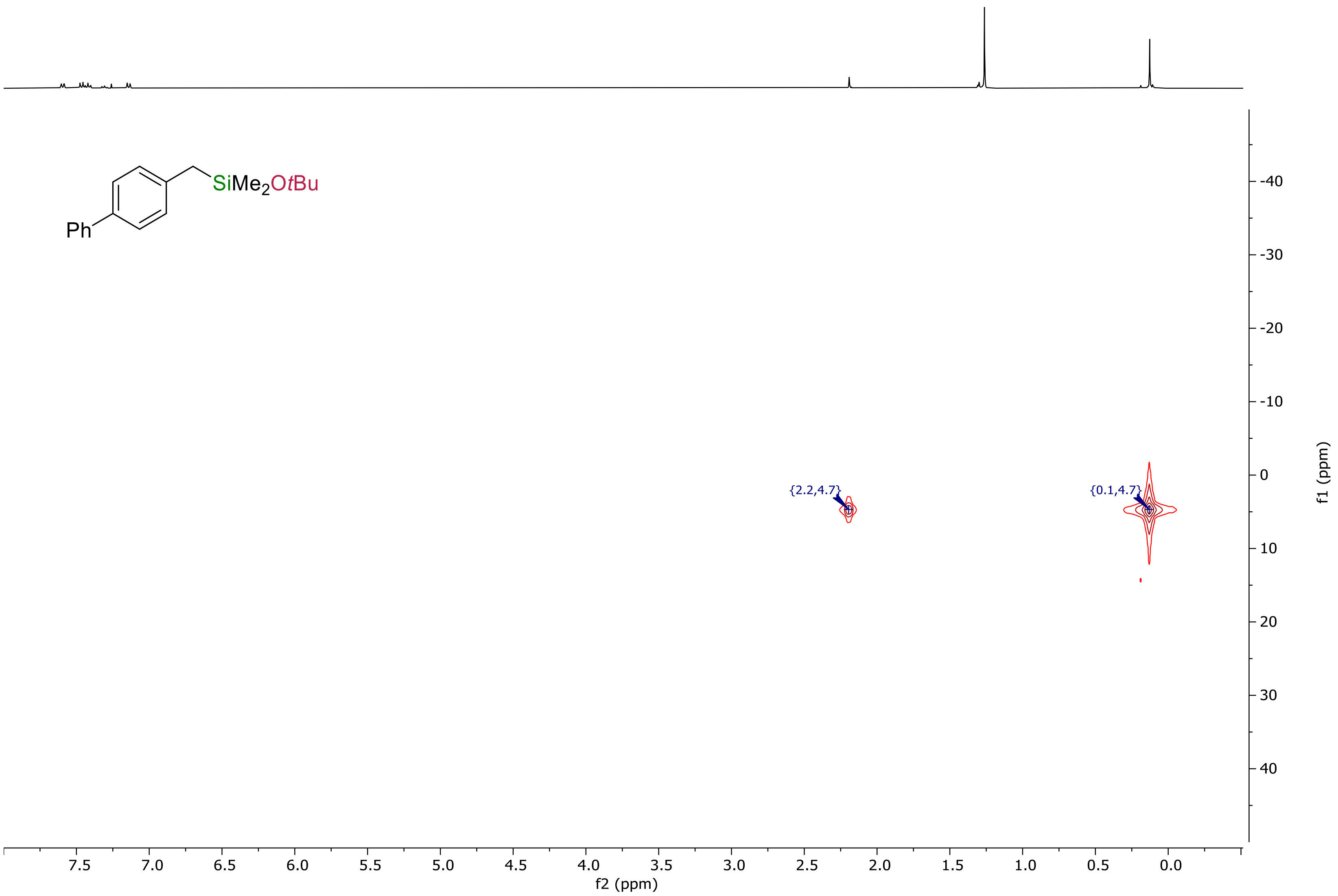

Figure S116: $^{1}H/^{29}Si$ HMQC NMR (400/79 MHz, $CDCl_3$) of compound **29a**.

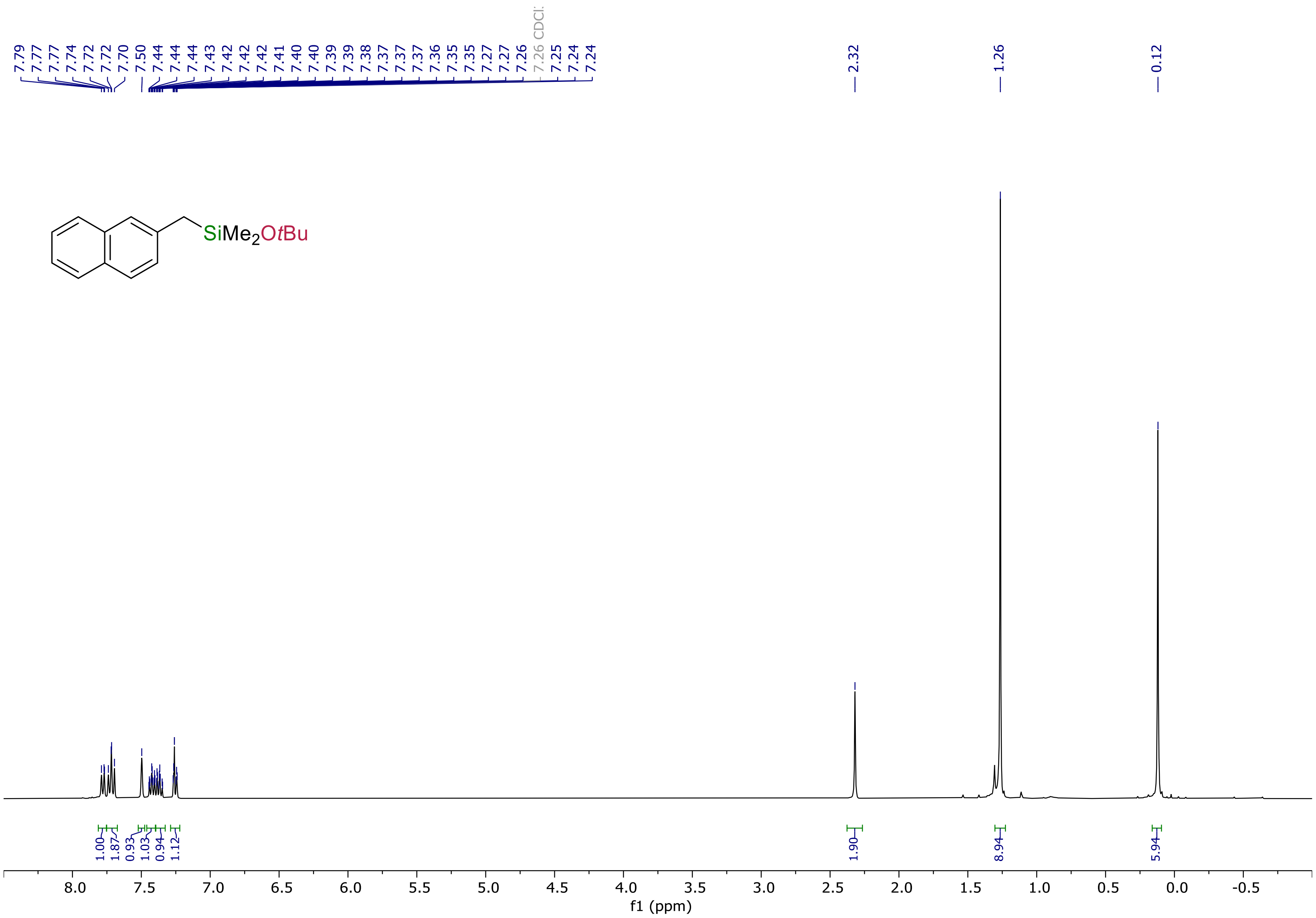

Figure S117: ^{1}H NMR (300 MHz, $CDCl_3$) of compound **30a**.

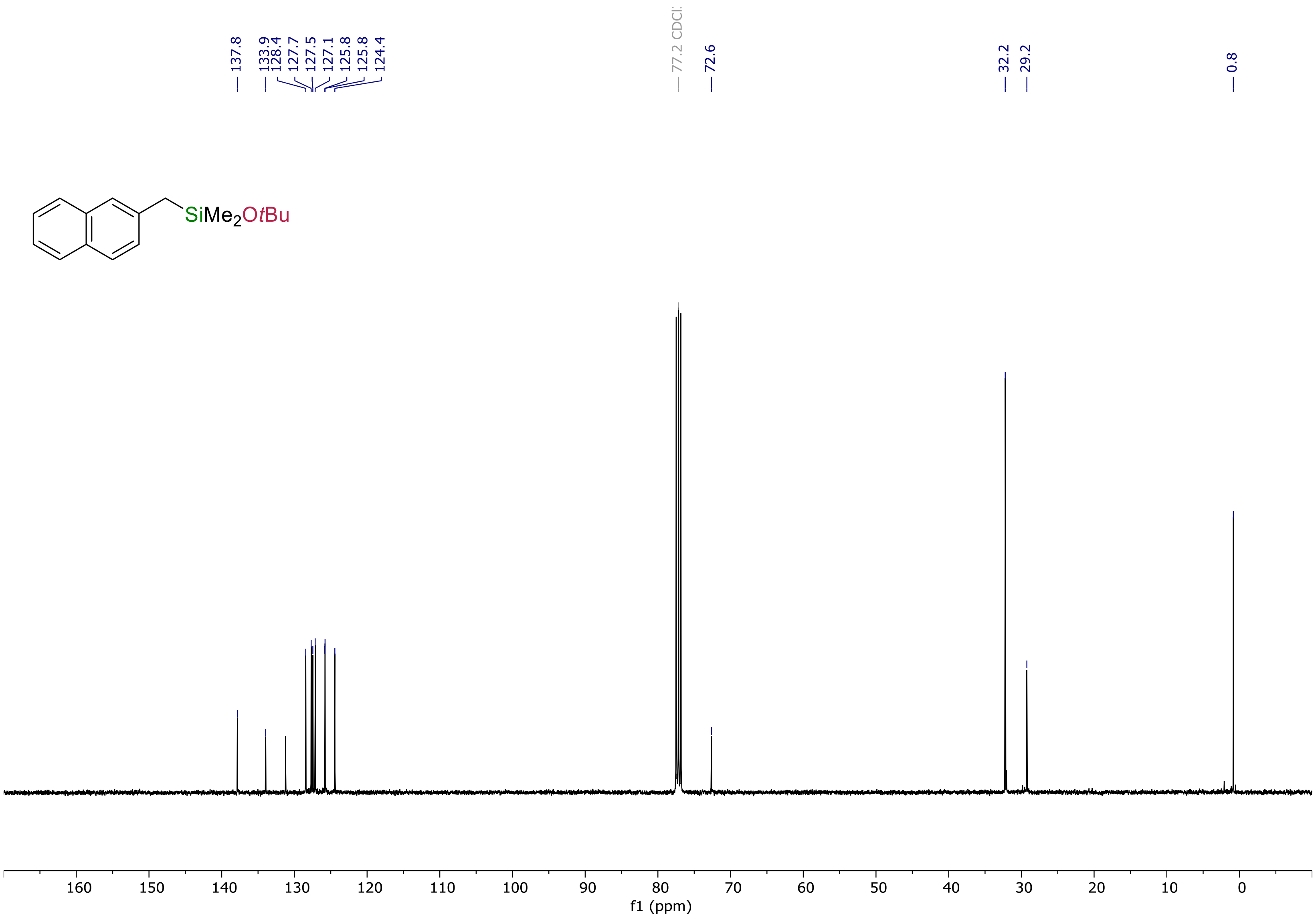

Figure S118: $^{13}C\{^{1}H\}$ NMR (75 MHz, $CDCl_3$) of compound **30a**.

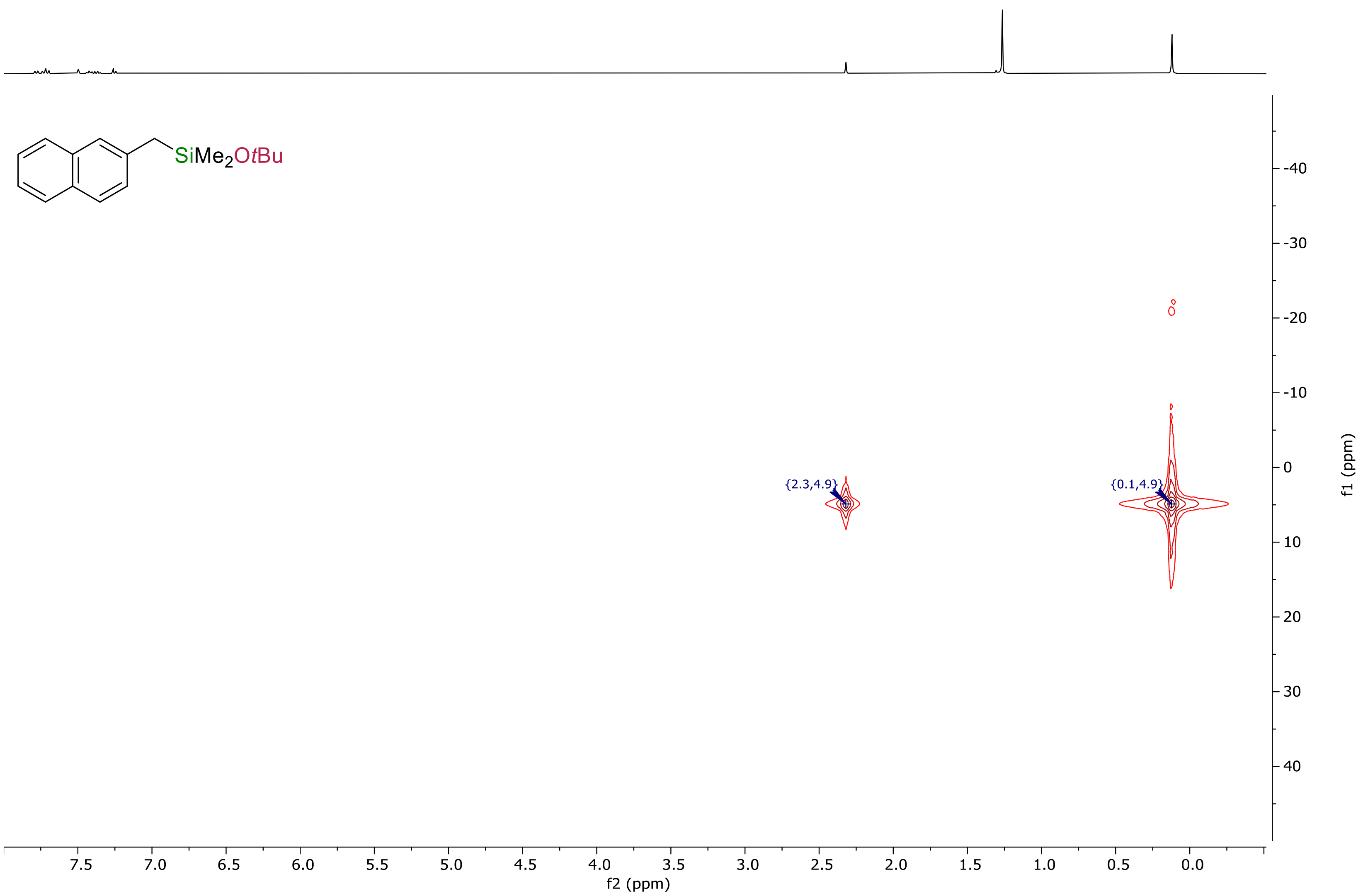

Figure S119: $^{1}H/^{29}Si$ HMQC NMR (300/60 MHz, $CDCl_3$) of compound **30a**.

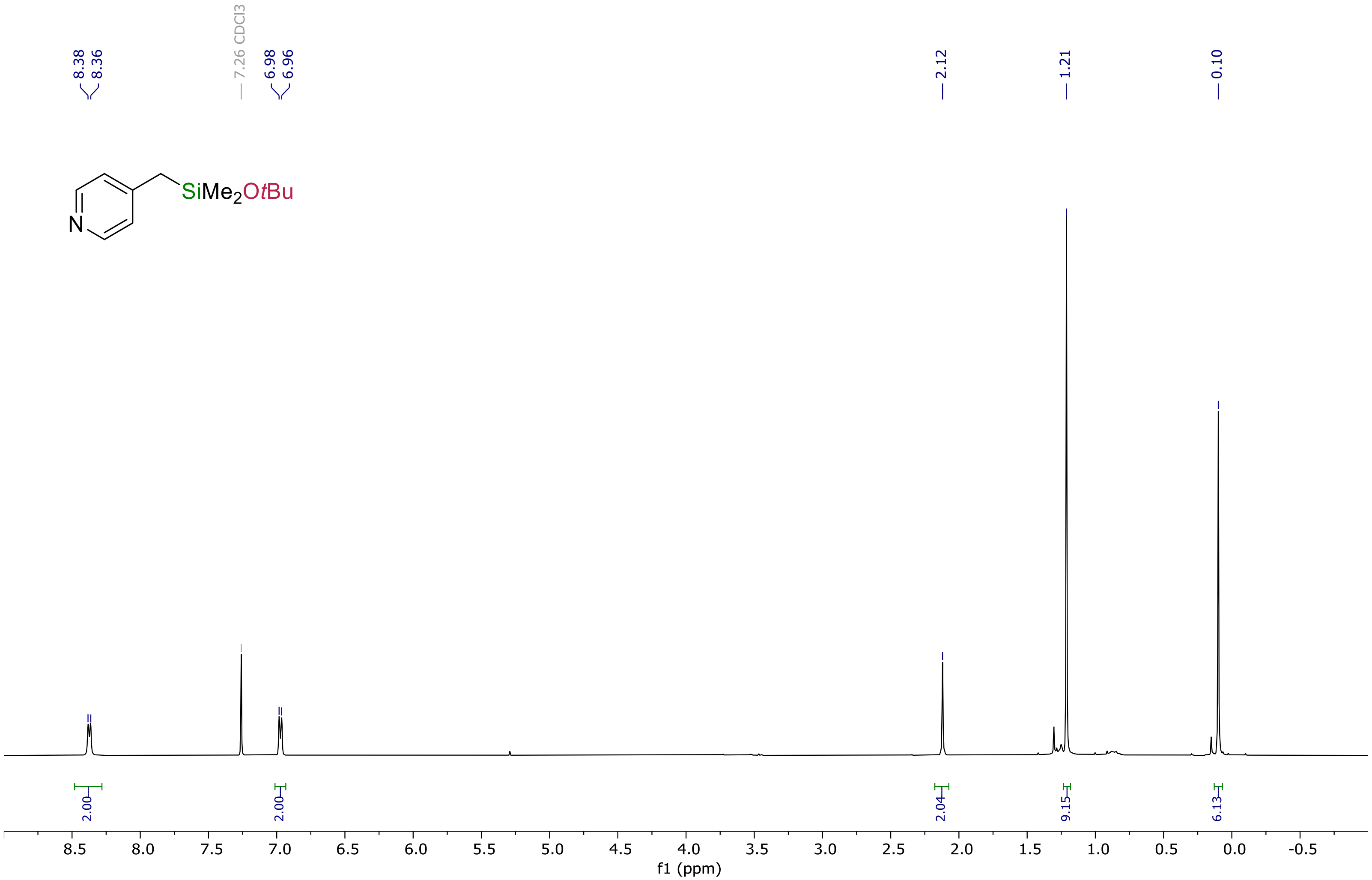

Figure S120: ^{1}H NMR (300 MHz, $CDCl_3$) of compound **31a**.

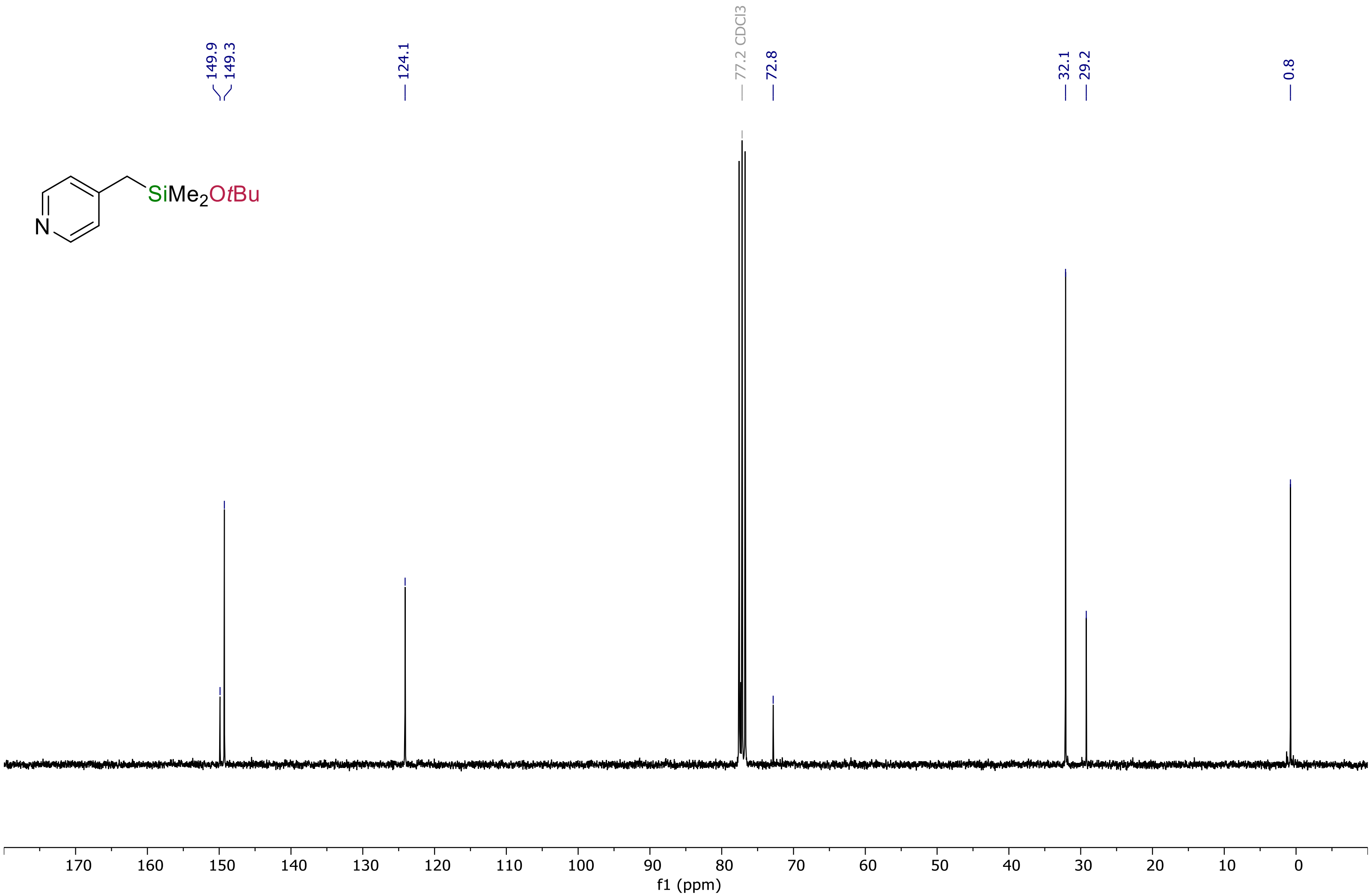

Figure S121: $^{13}C\{^1H\}$ NMR (75 MHz, $CDCl_3$) of compound **31a**.

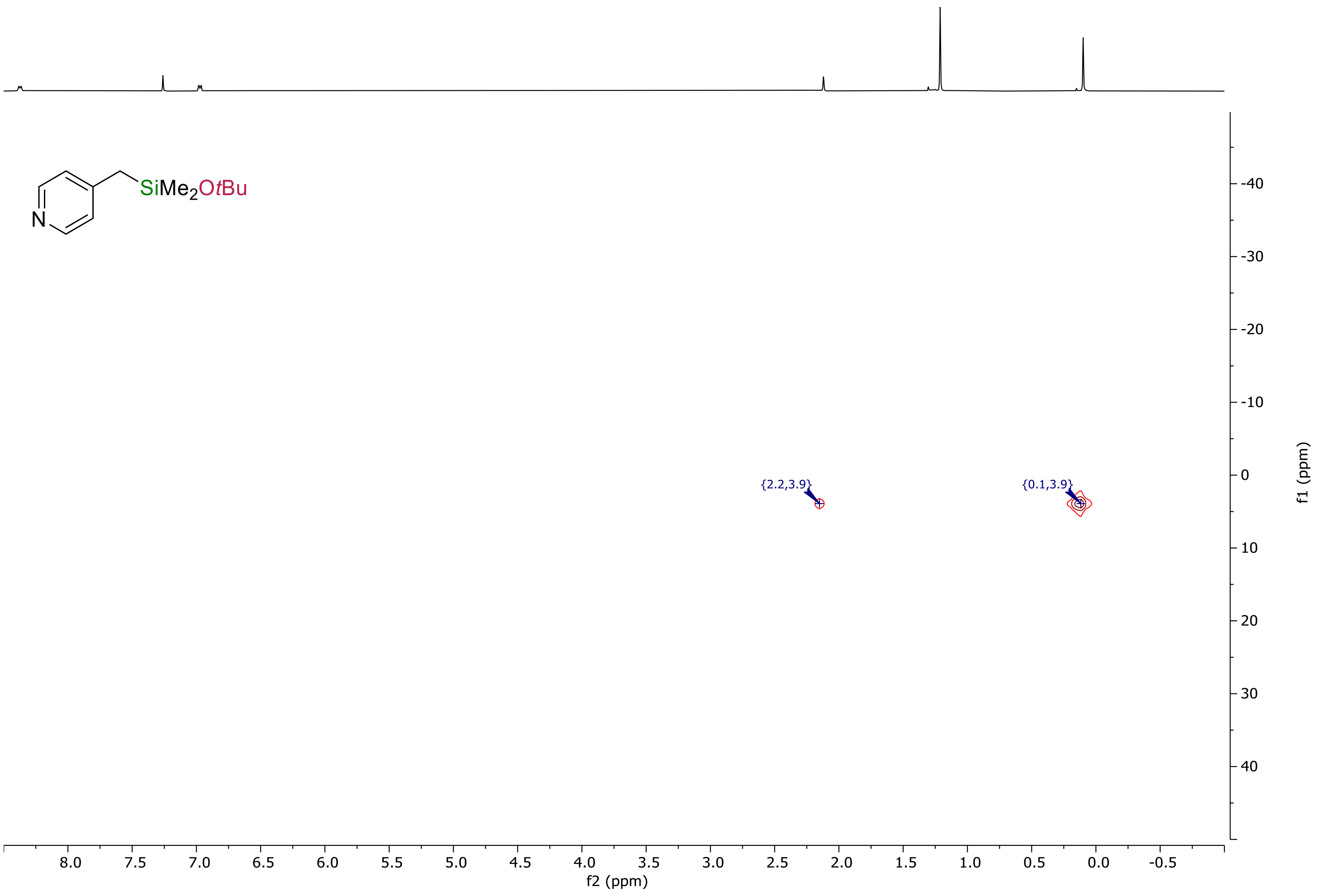

Figure S 122: $^{1}H/^{29}Si$ HMQC NMR (300/60 MHz, $CDCl_3$) of compound **31a**.

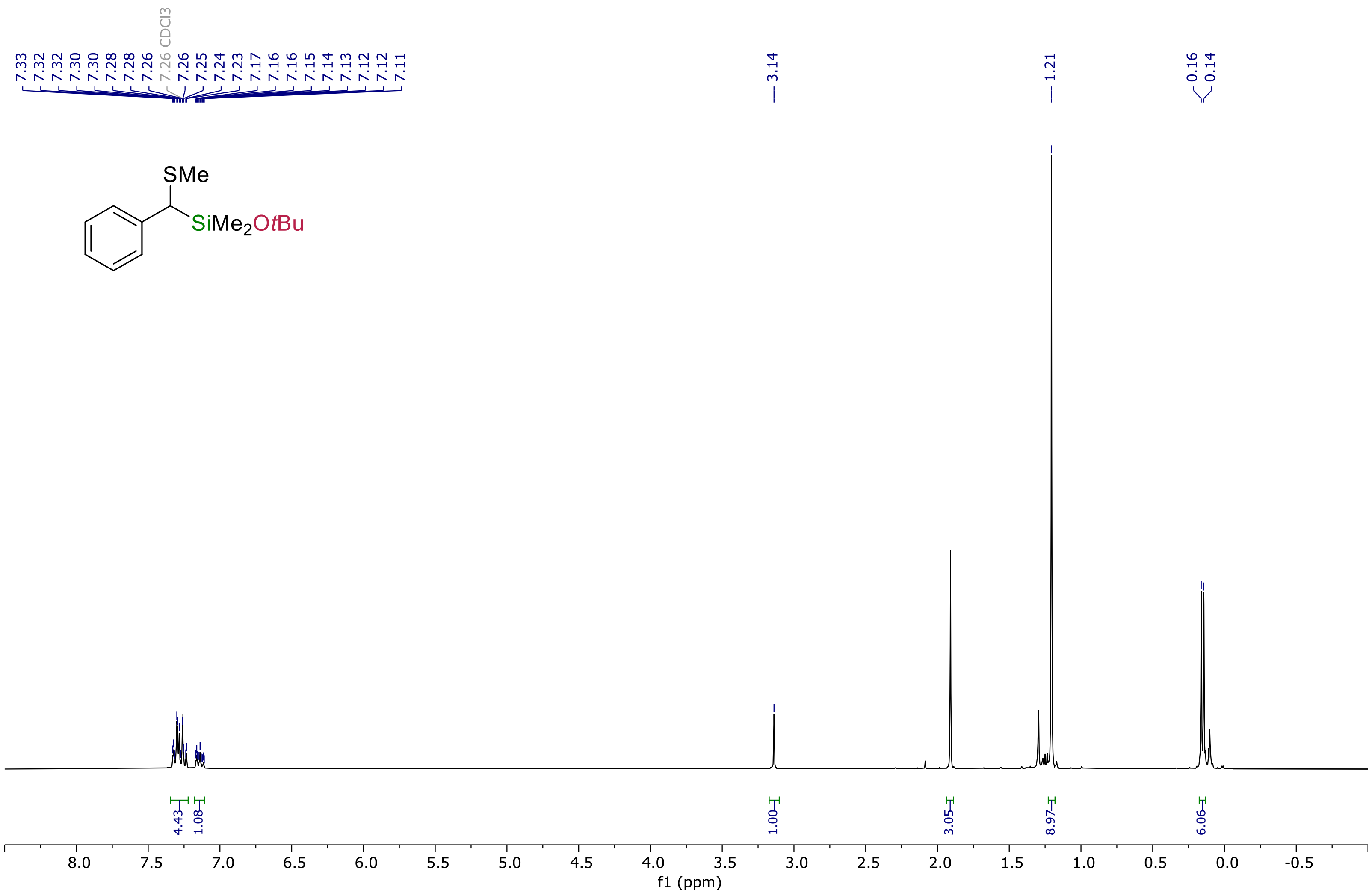

;

Figure S123: ^{1}H NMR (300 MHz, $CDCl_3$) of compound **32a**.

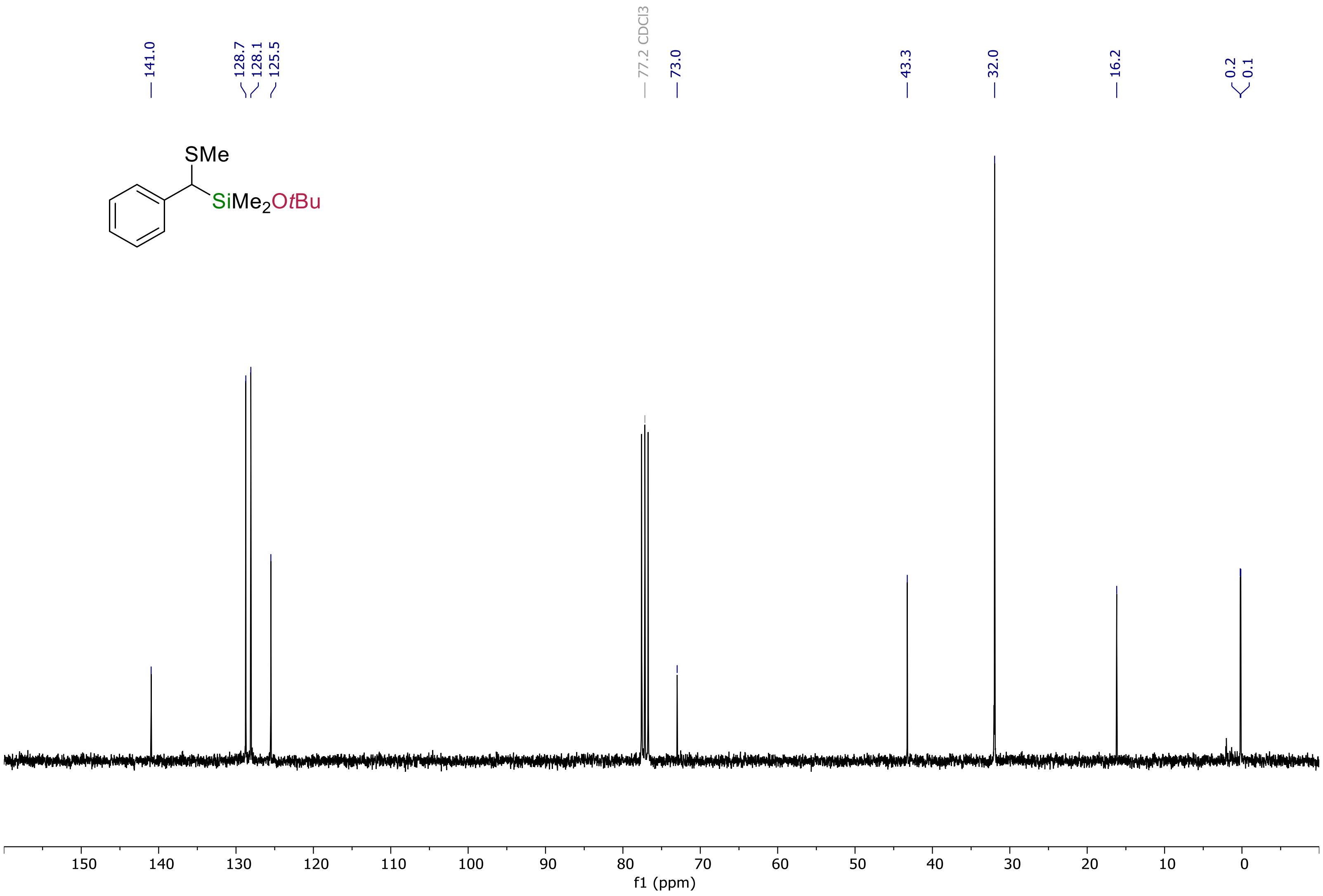

Figure S124: $^{13}C\{^{1}H\}$ NMR (75 MHz, $CDCl_3$) of compound **32a**.

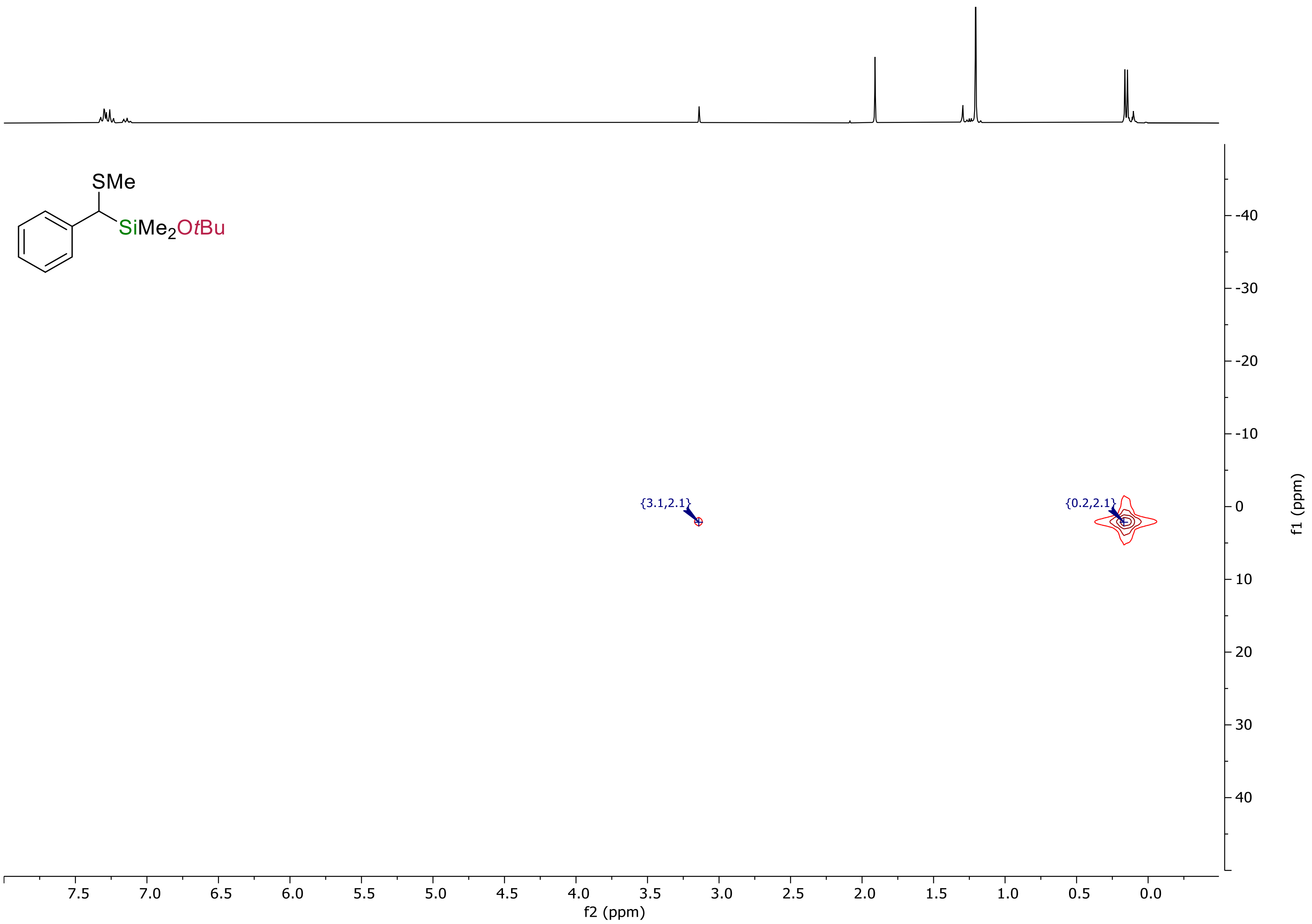

Figure S125: $^{1}H/^{29}Si$ HMQC NMR (300/60 MHz, $CDCl_3$) of compound **32a**.

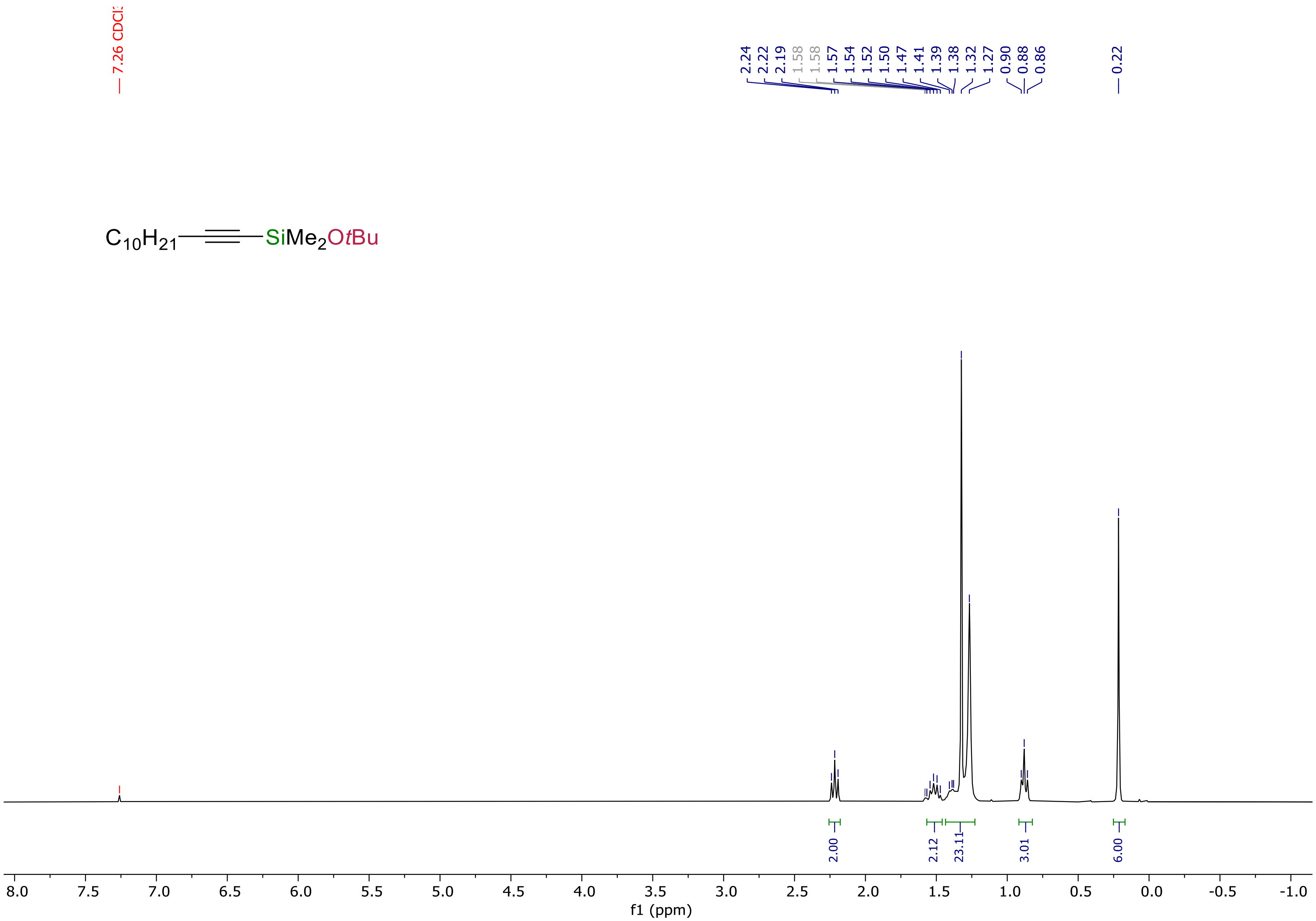

Figure S126: ^{1}H NMR (300 MHz, $CDCl_3$) of compound **33a**.

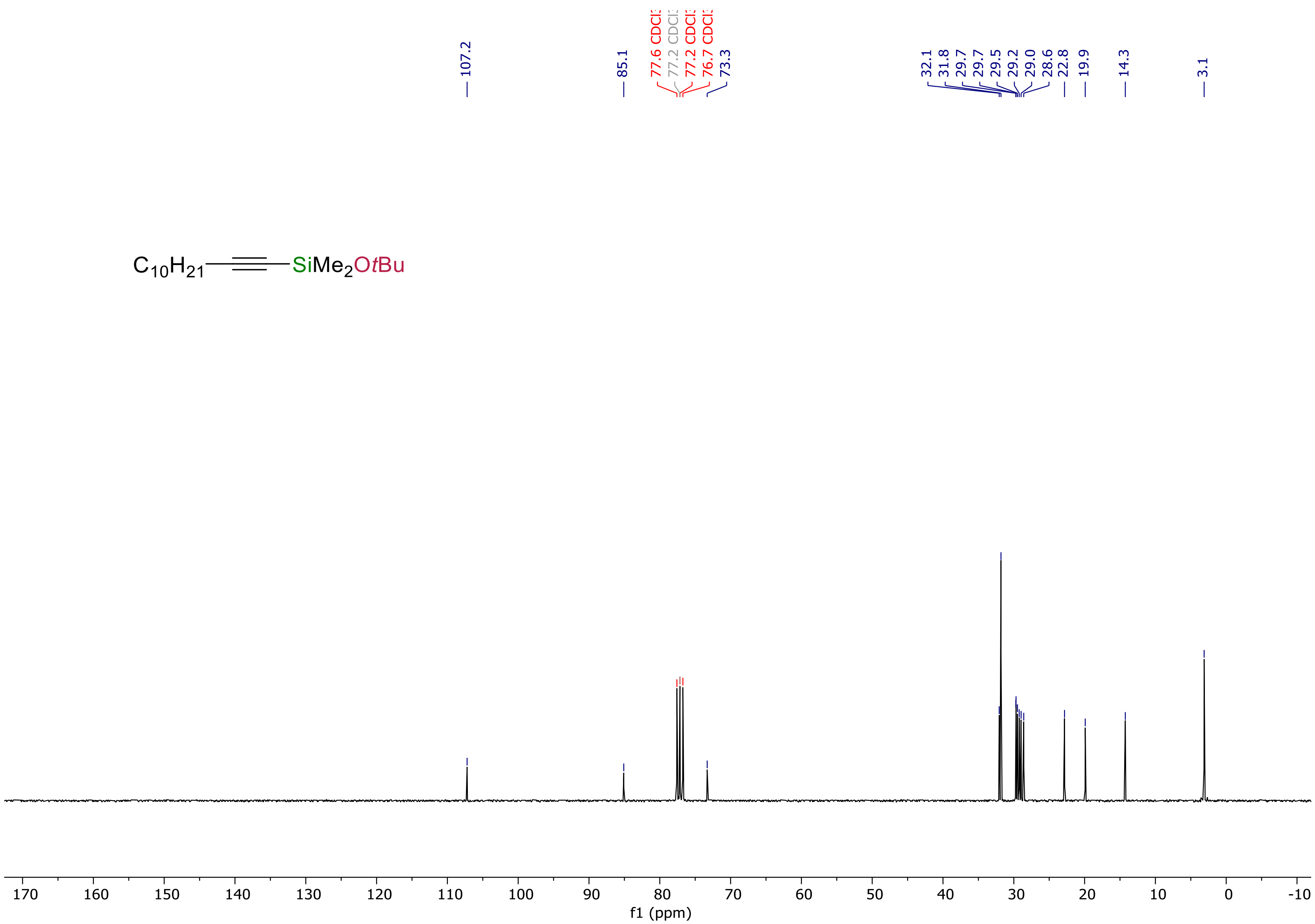

Figure S127: $^{13}C\{^{1}H\}$ NMR (101 MHz, CDCl3) of compound **33a**.

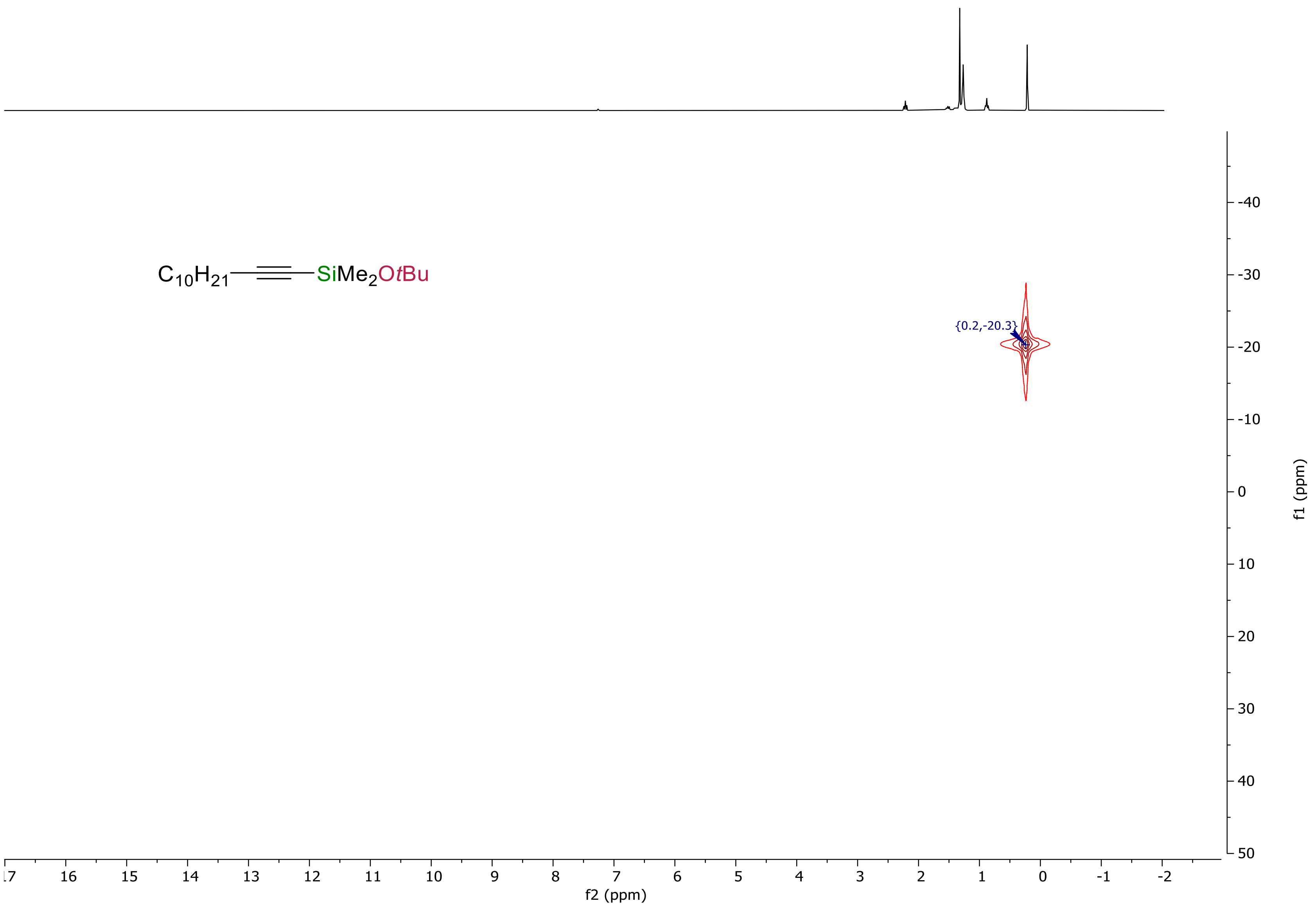

Figure S128: $^{1}H/^{29}Si$ HMQC NMR (300/79 MHz, $CDCl_3$) of compound **33a**.

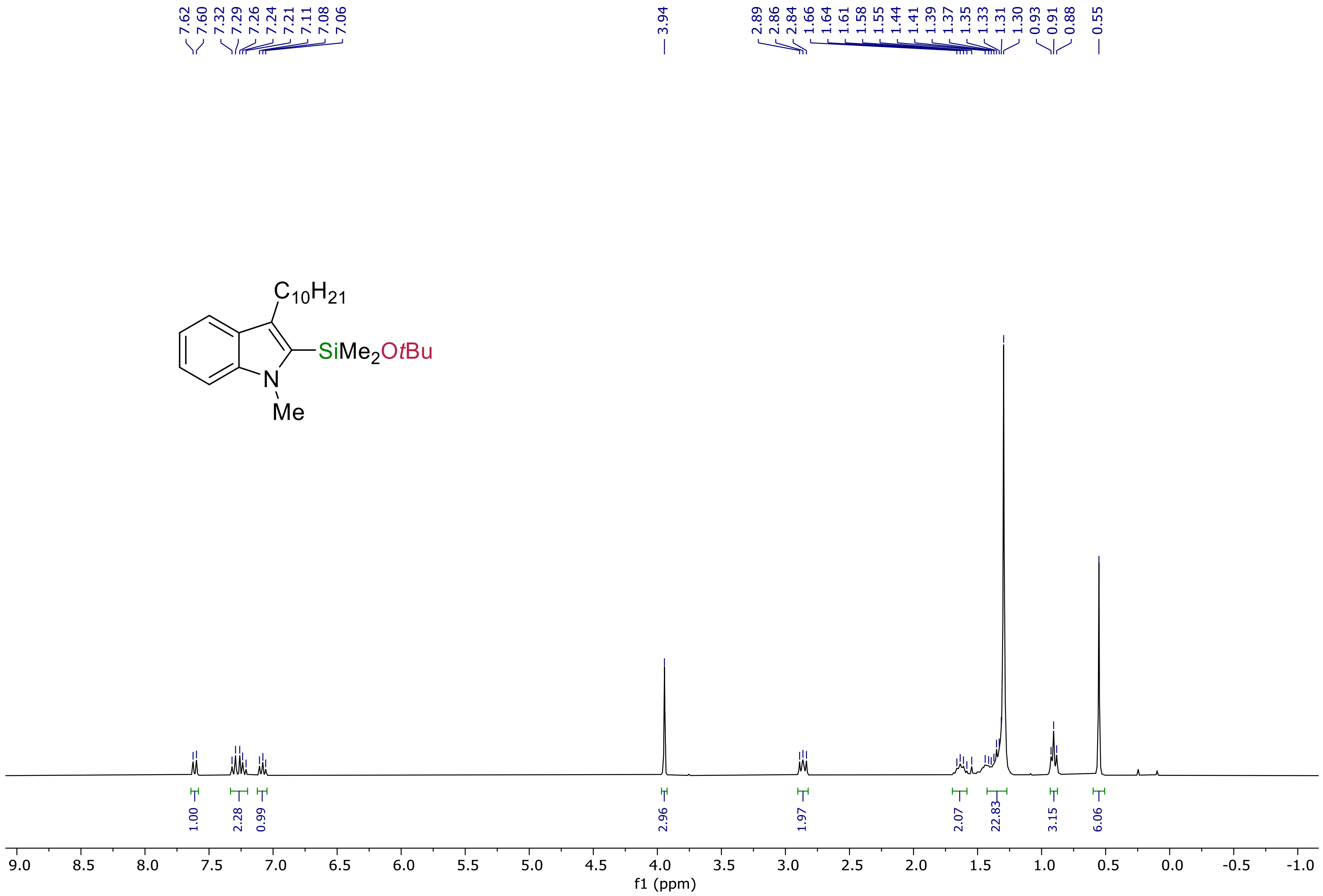

Figure S129: ^{1}H NMR (300 MHz, $CDCl_3$) of compound **34**.

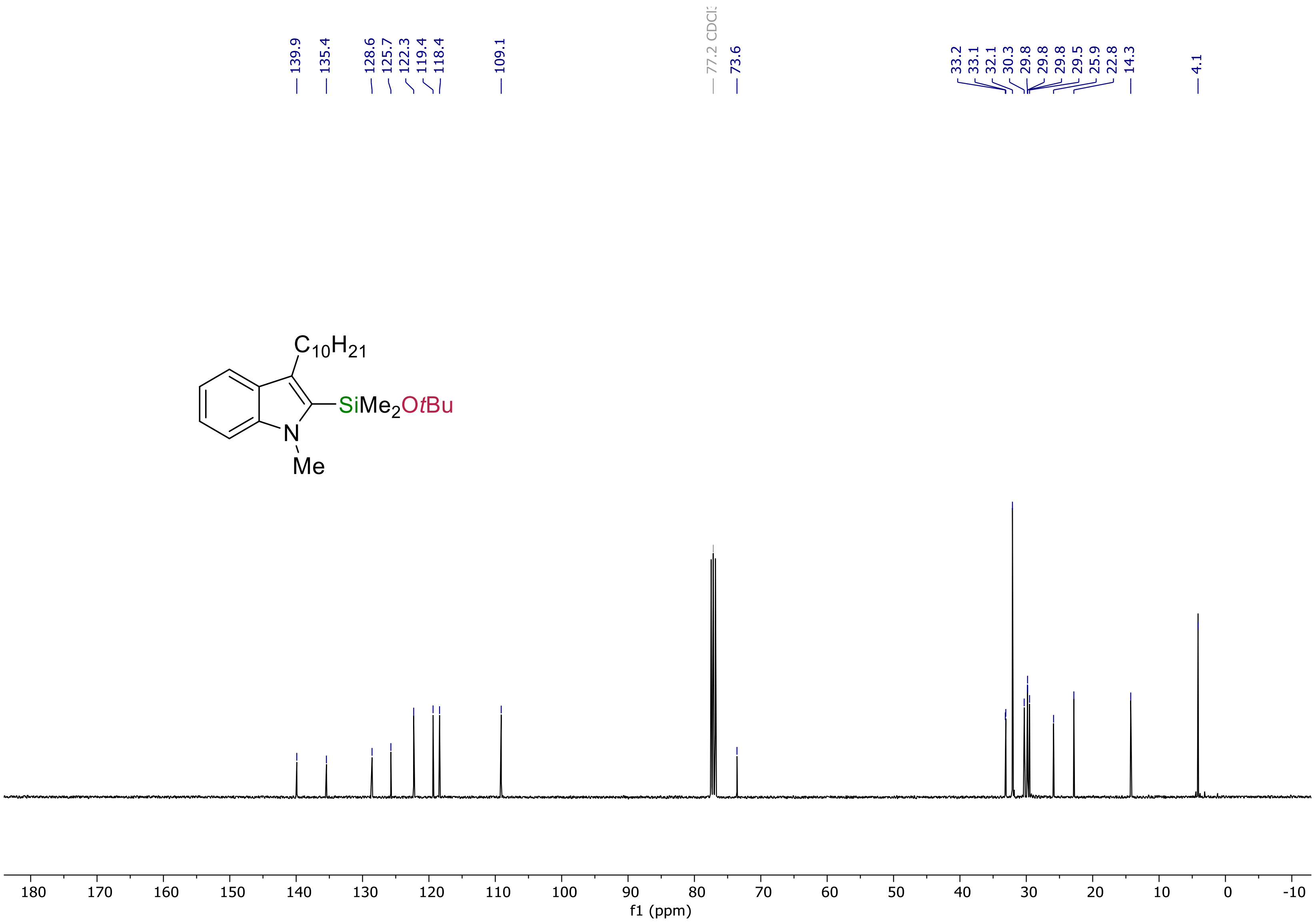

Figure S130: $^{13}C\{^{1}H\}$ NMR (101 MHz, CDCl3) of compound **34**.

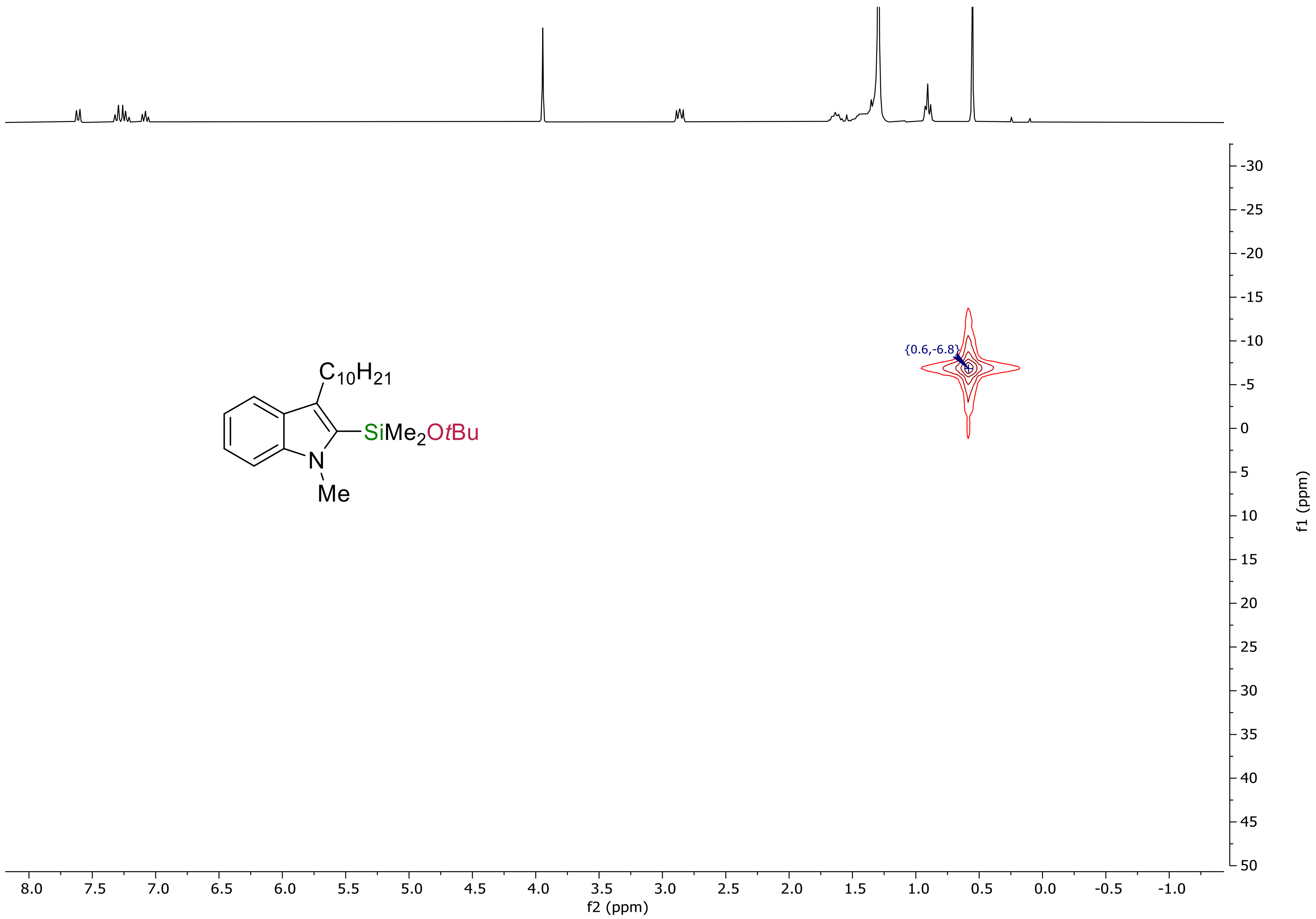

Figure S131: $^{1}H/^{29}Si$ HMQC NMR (300/79 MHz, $CDCl_3$) of compound **34**

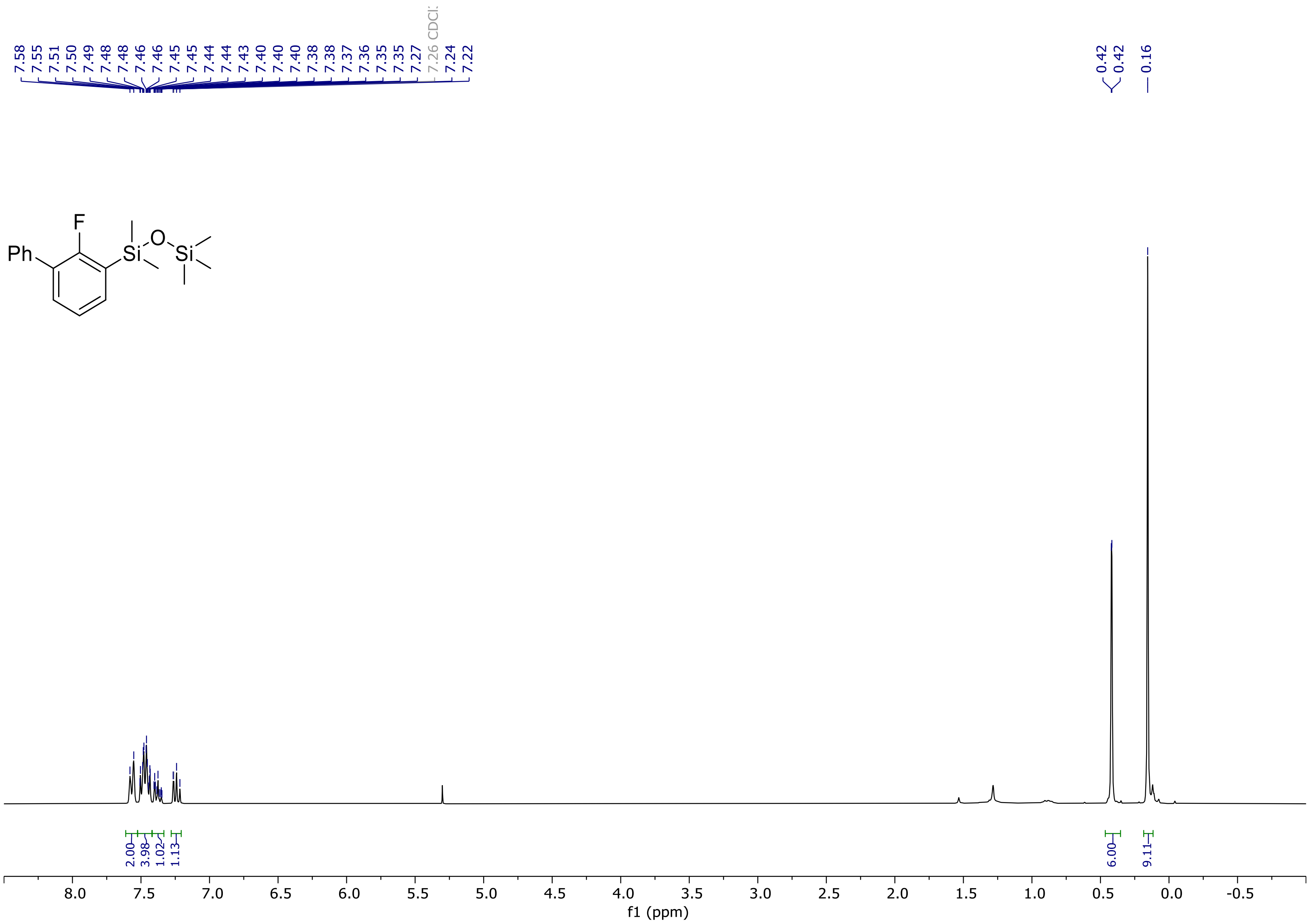

Figure S132: ^{1}H NMR (300 MHz, $CDCl_3$) of compound **35.**

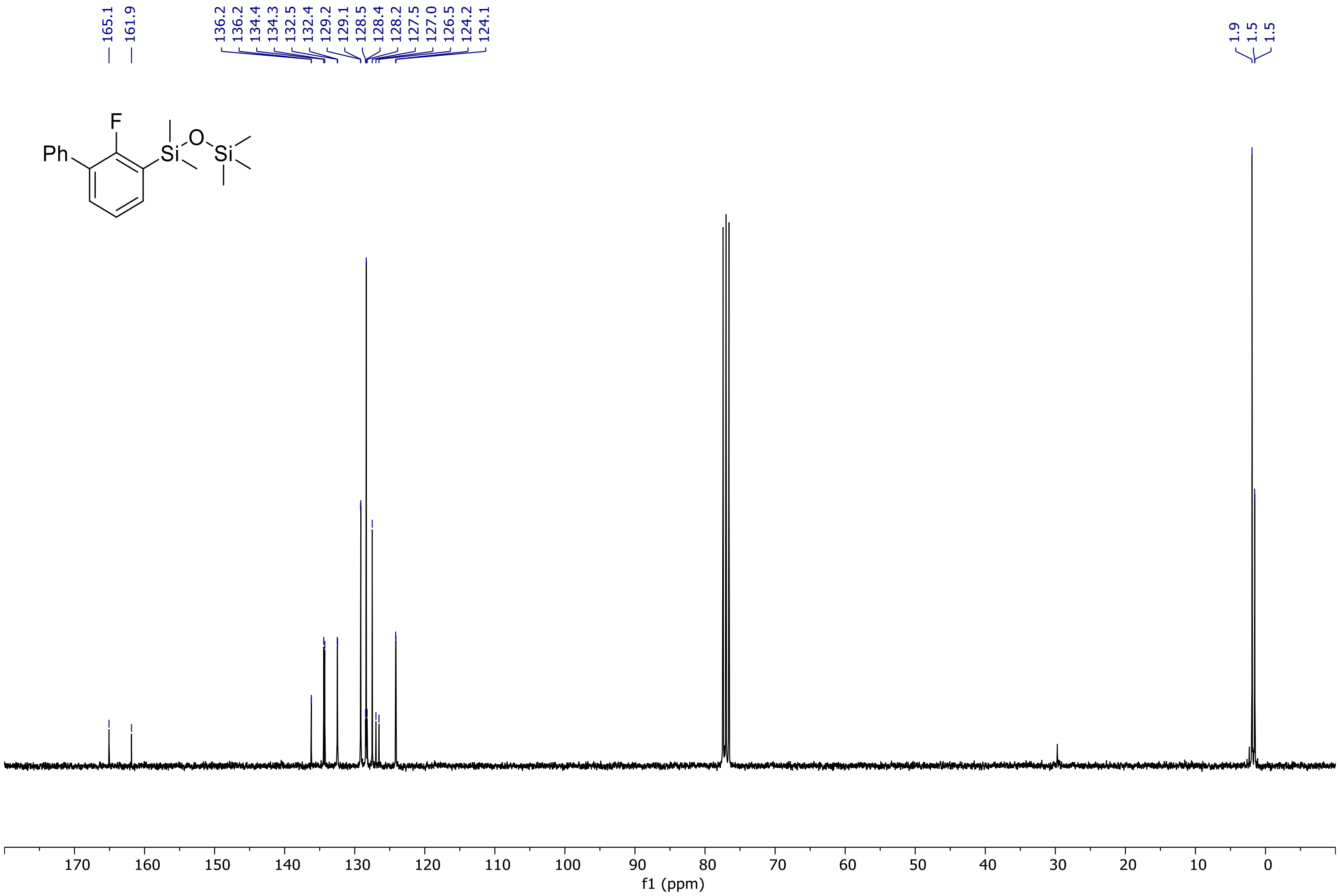

Figure S133: $^{13}C\{^{1}H\}$ NMR (75 MHz, $CDCl_3$) of compound **35**.

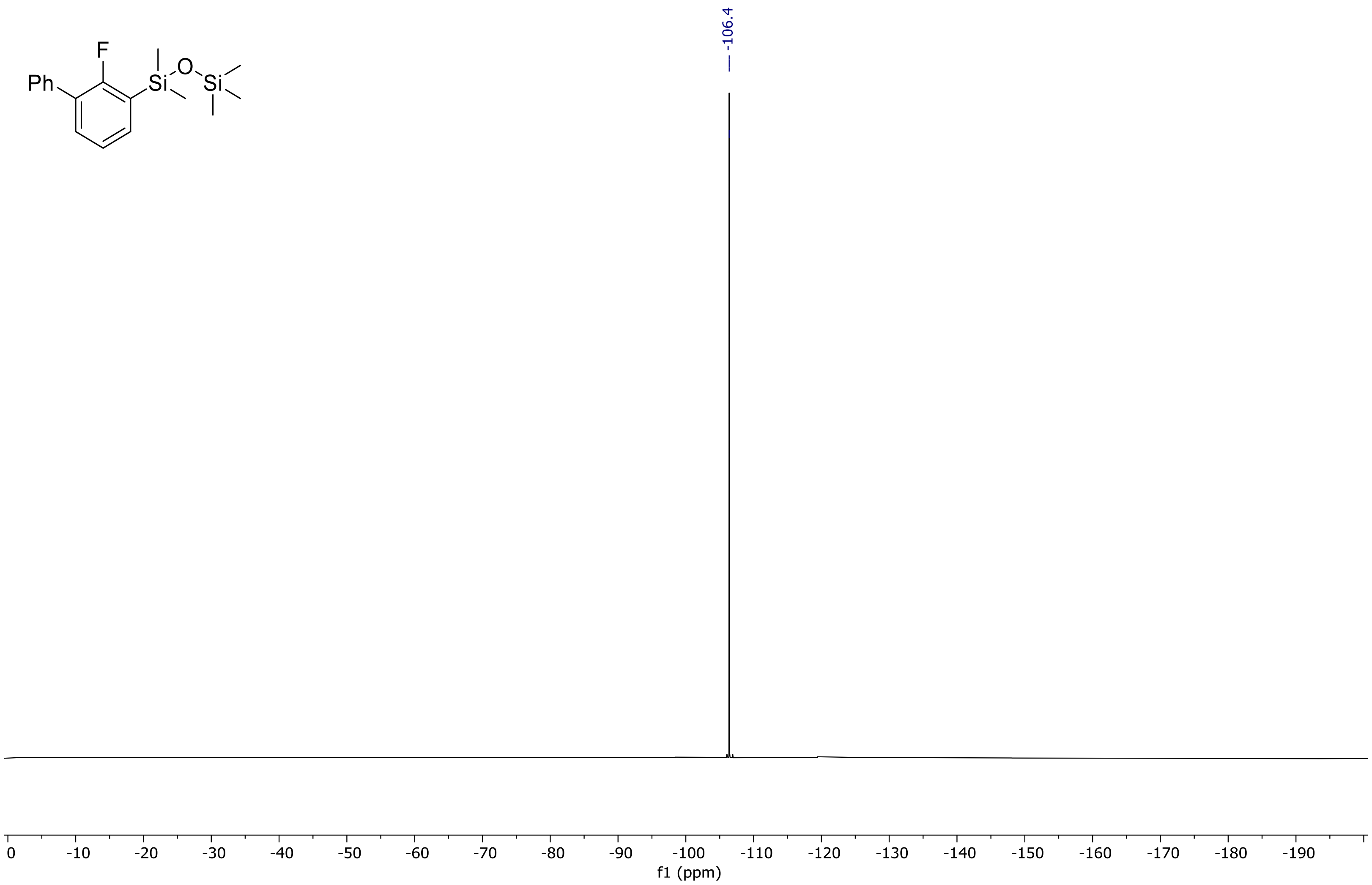

Figure S134: $^{19}F\{^{1}H\}$ NMR (282 MHz, $CDCl_3$) of compound **35**.

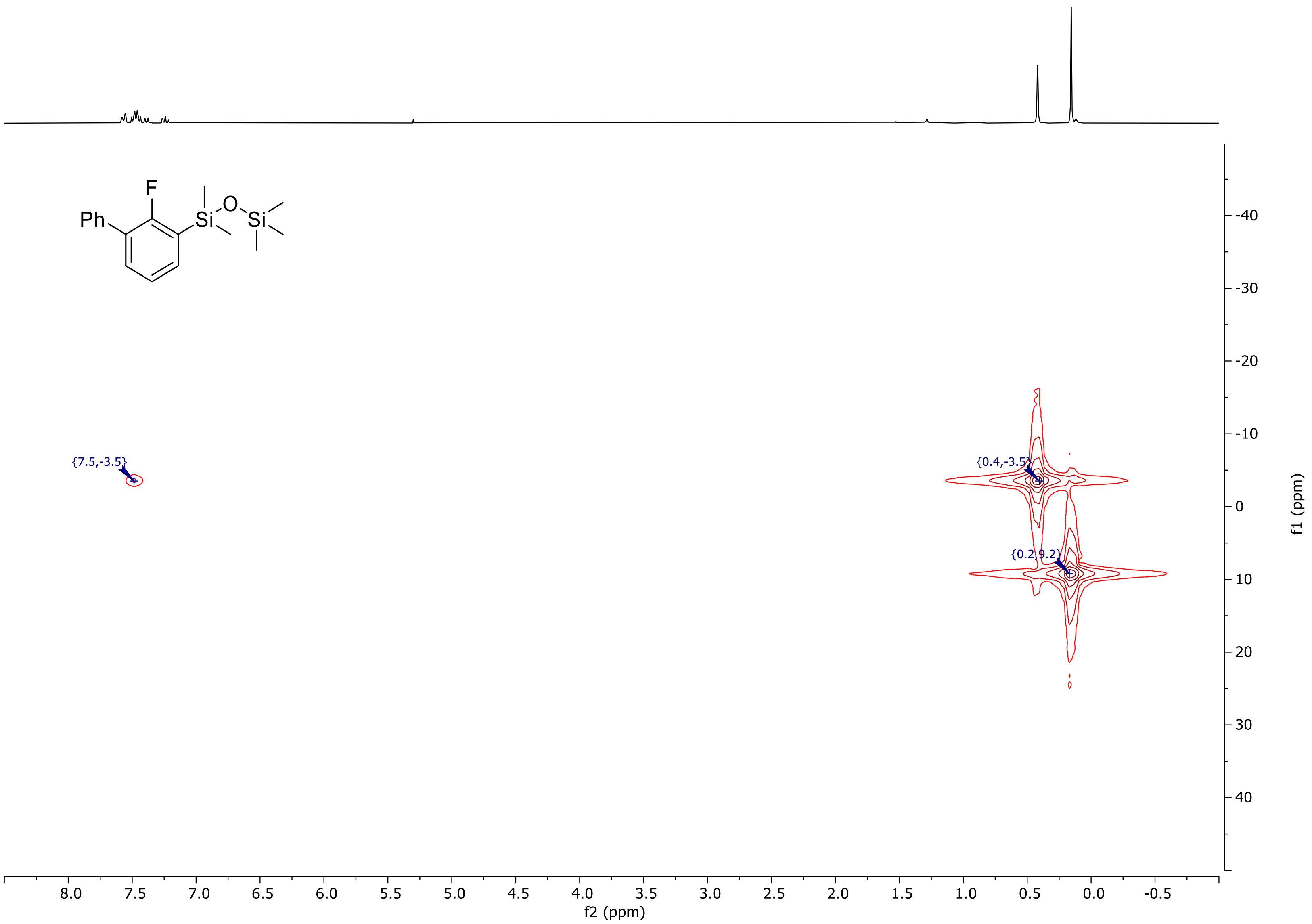

Figure S135: $^{1}H/^{29}Si$ HMQC NMR (300/60 MHz, $CDCl_3$) of compound **35**.

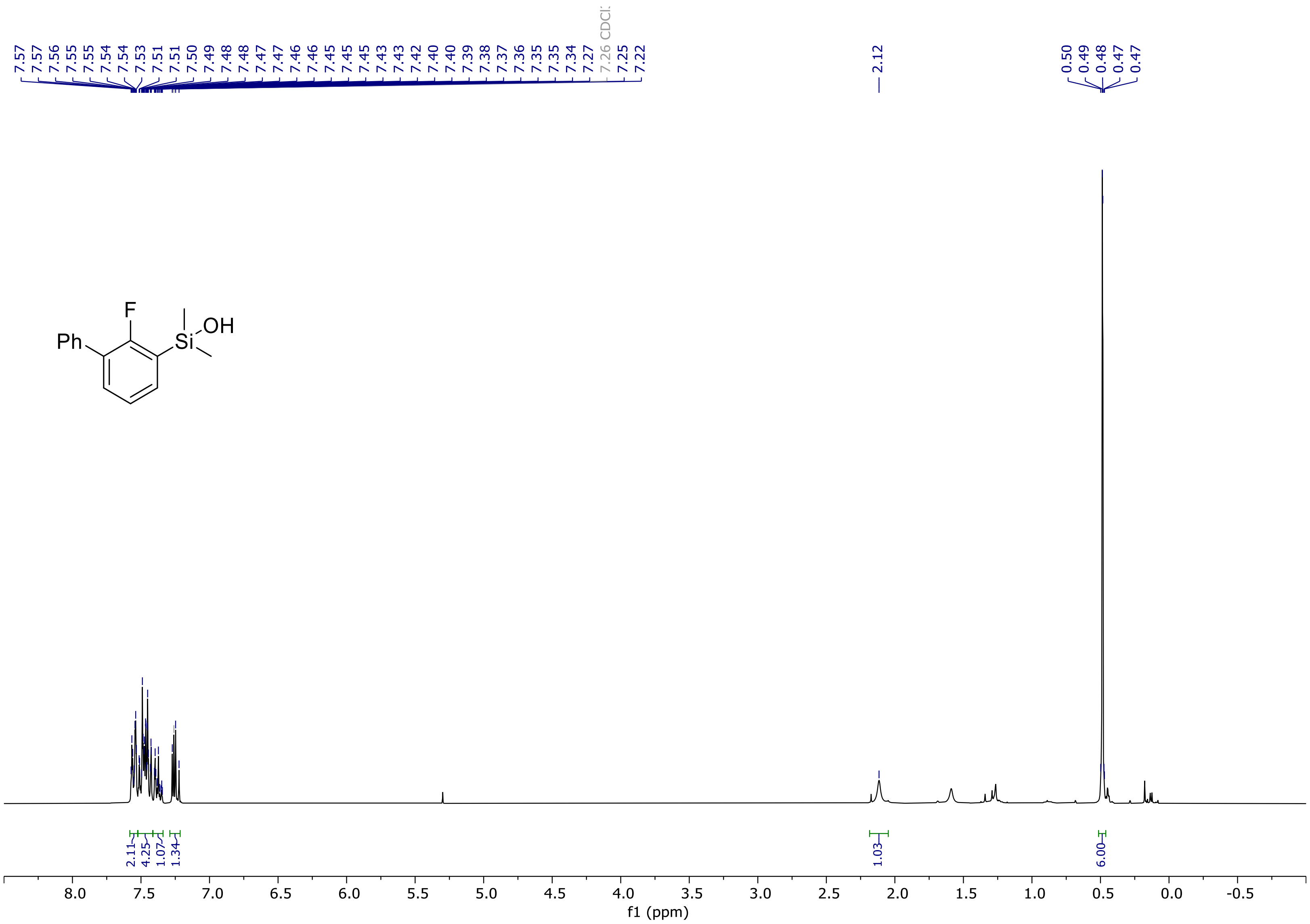

Figure S136: ^{1}H NMR (300 MHz, $CDCl_3$) of compound **36**.

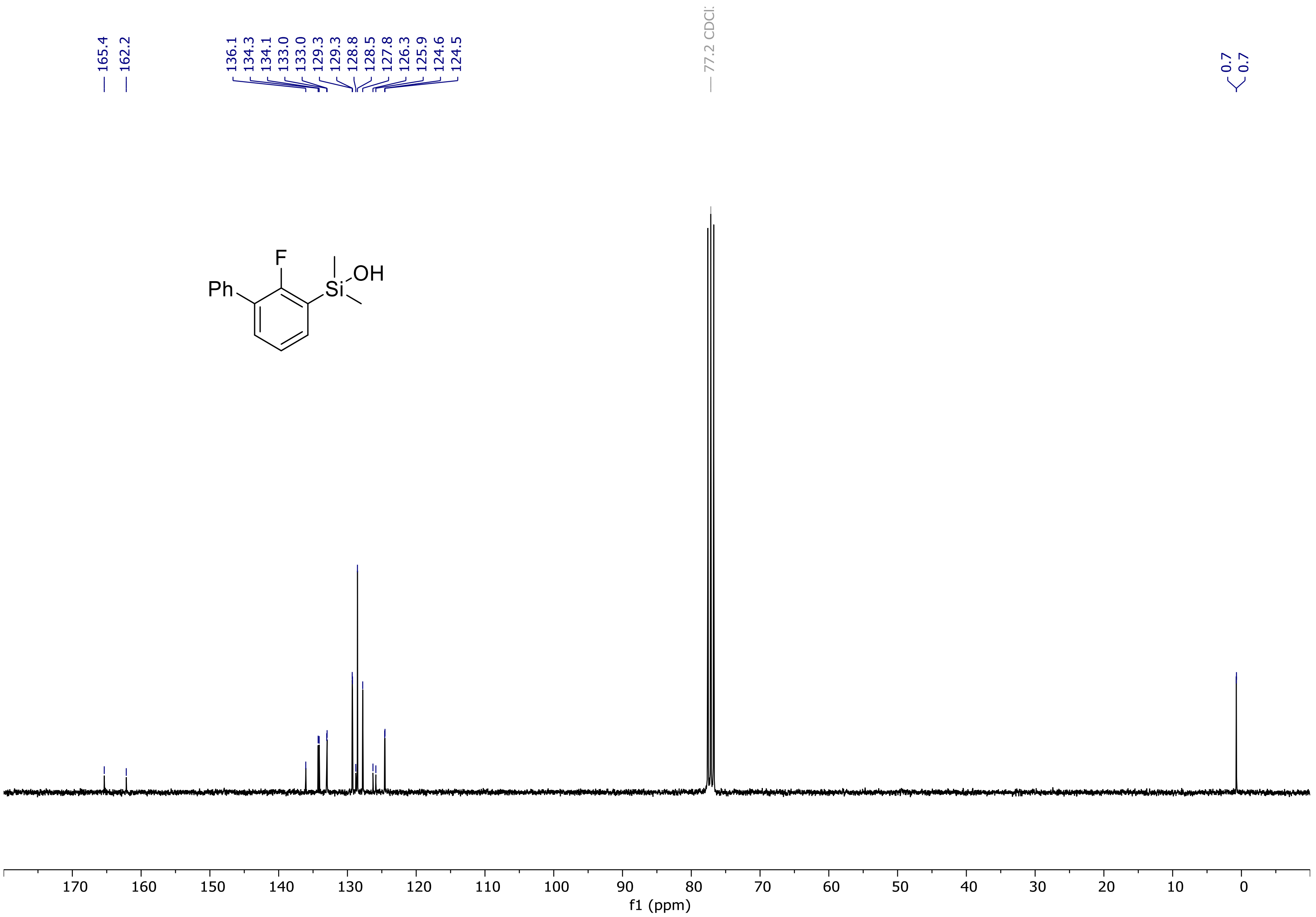

Figure S137: $^{13}C\{^{1}H\}$ NMR (75 MHz, $CDCl_3$) of compound **36**.

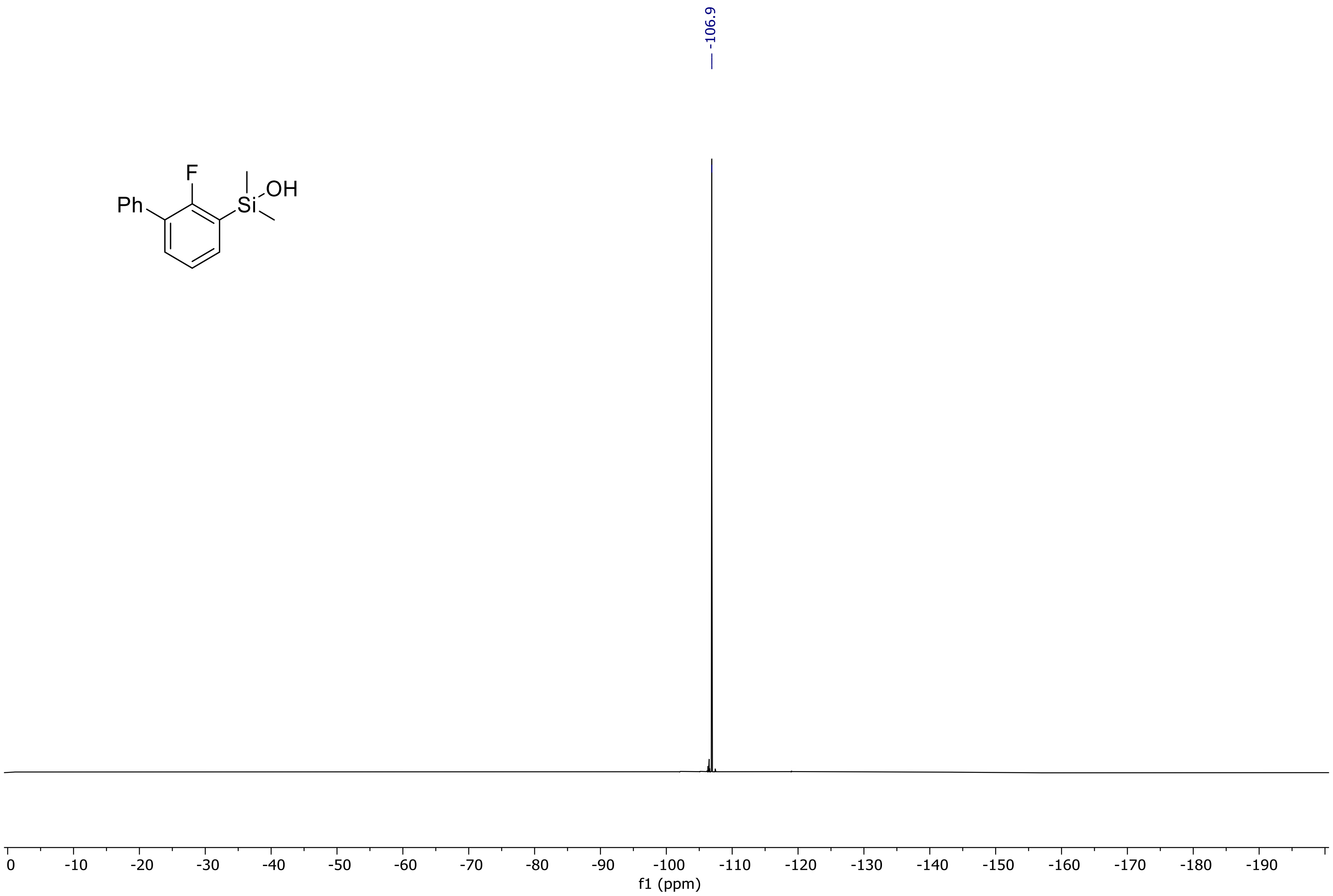

Figure S138: $^{19}F\{^{1}H\}$ NMR (282 MHz, $CDCl_3$) of compound **36**.

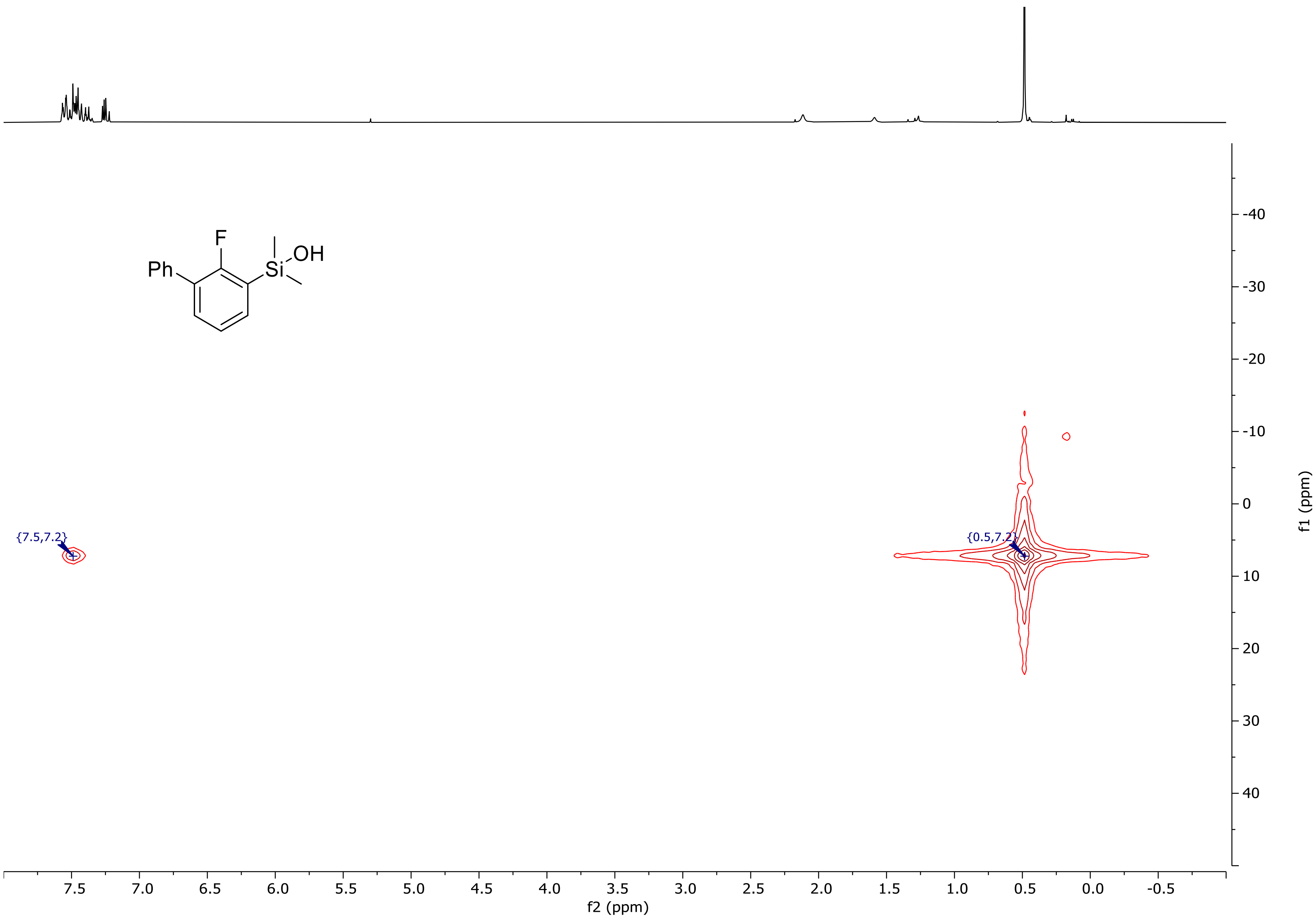

Figure S139: $^{1}H/^{29}Si$ HMQC NMR (300/60 MHz, $CDCl_3$) of compound **36**.

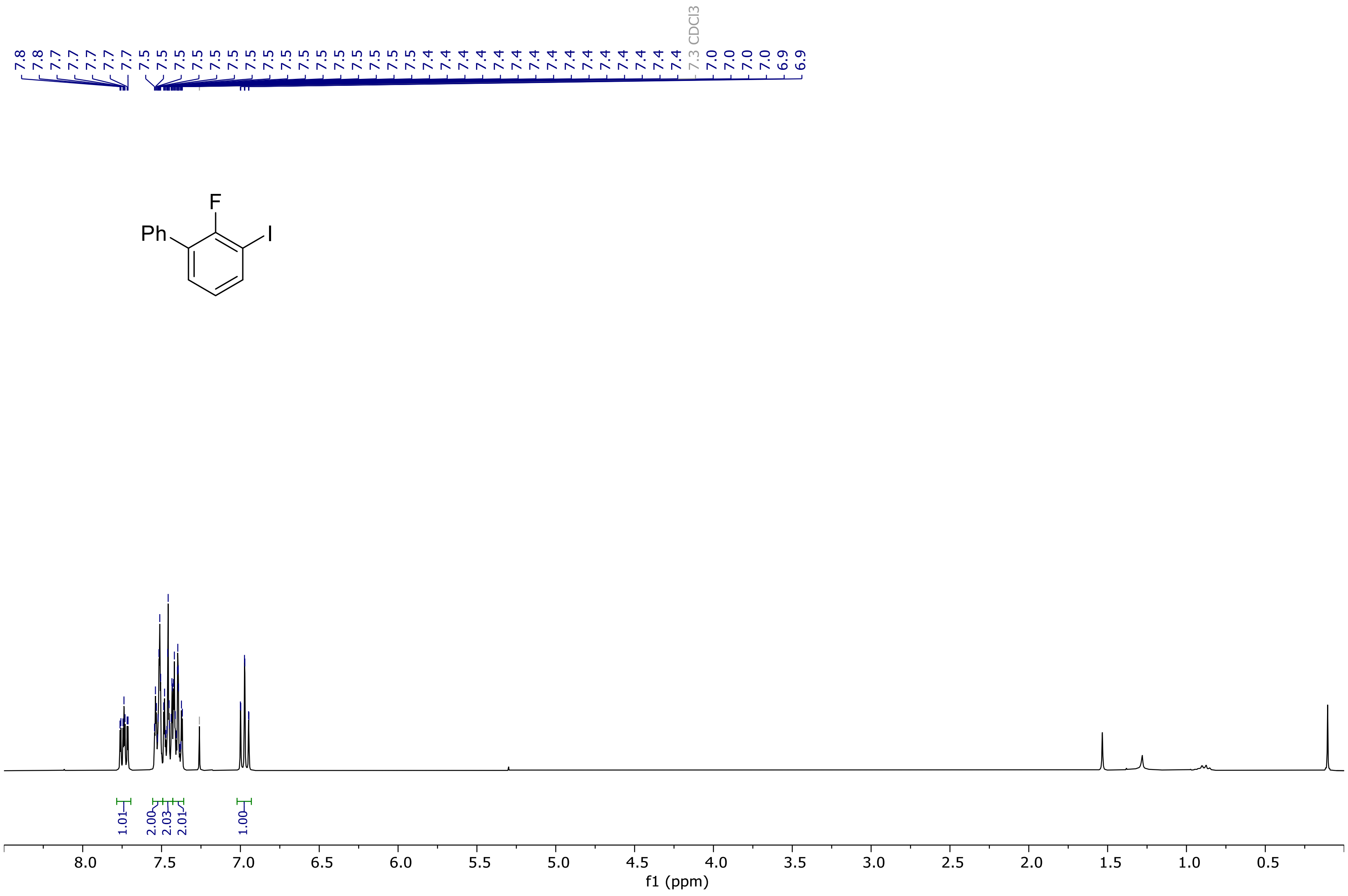

Figure S140: ^{1}H NMR (300 MHz, $CDCl_3$) of compound **38**.

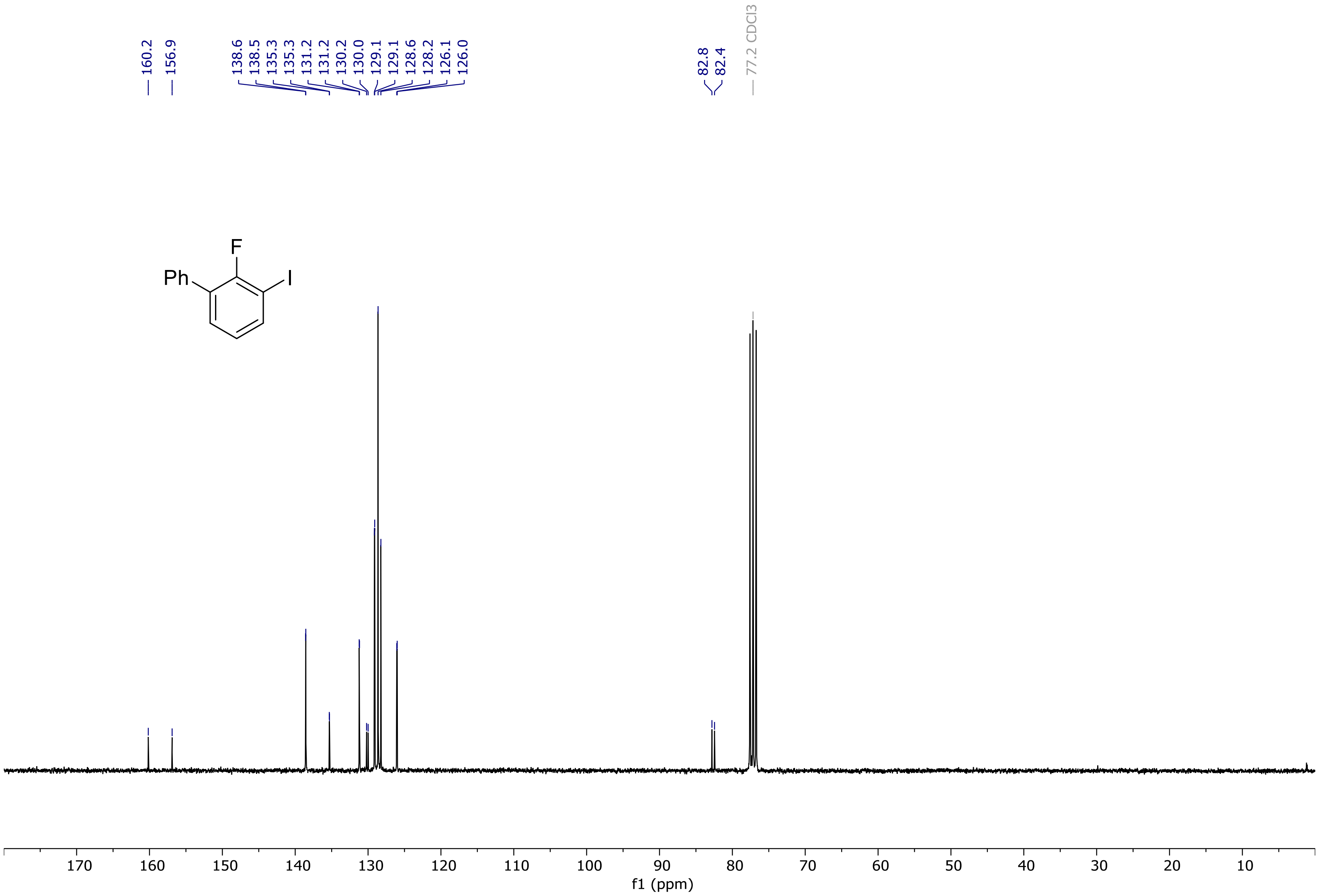

Figure S141: $^{13}C\{^{1}H\}$ NMR (75 MHz, $CDCl_3$) of compound **38**.

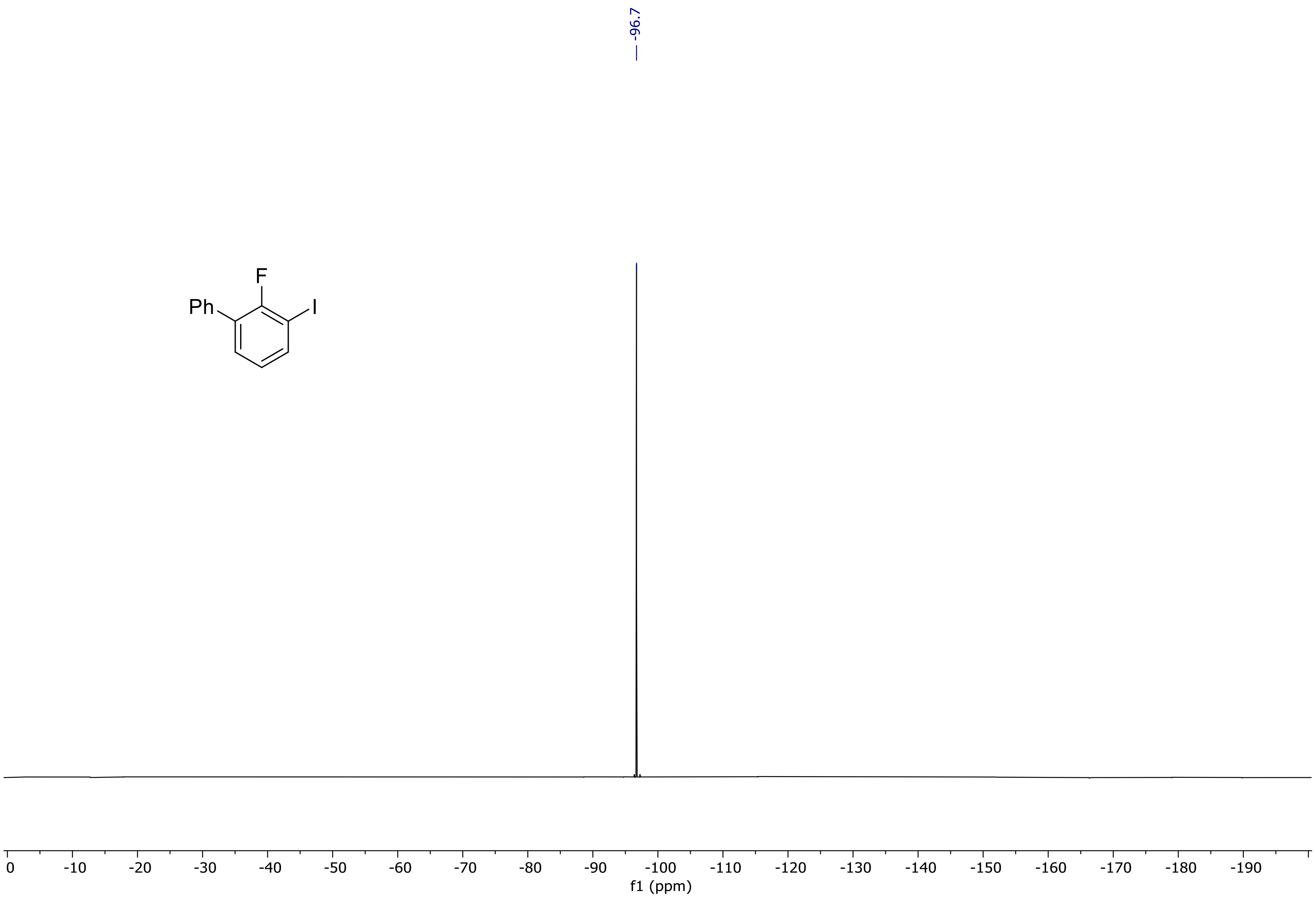

Figure S142: $^{19}F\{^{1}H\}$ NMR (282 MHz, $CDCl_3$) of compound **38**.

8. References

1 F. Turnu, A. Luridiana, A. Cocco, S. Porcu, A. Frongia, G. Sarais and F. Secci, *Org. Lett.*, 2019, **21**, 7329–7332.
2 B. Neil, L. Saadi, L. Fensterbank and C. Chauvier, *Angew. Chem. Int. Ed.*, 2023, **62**, e202306115.
3 S. Som, J. Choi, D. Katsoulis and K. L. Lee, *Chem. Sci.*, 2022, **13**, 10759–10764.
4 H. Saito, J. Shimokawa and H. Yorimitsu, *Chem. Sci.*, 2021, **12**, 9546–9555.
5 C. Cheng and J. F. Hartwig, *J. Am. Chem. Soc.*, 2015, **137**, 592–595.
6 Y. Ma, R. A. Woltornist, R. F. Algera and D. B. Collum, *J. Org. Chem.*, 2019, **84**, 9051–9057.
7 B. Neil, F. Lucien, L. Fensterbank and C. Chauvier, *ACS Catal.*, 2021, **11**, 13085–13090.
8 S. R. Docherty, D. P. Estes and C. Copéret, *Helv. Chim. Acta*, 2018, **101**.
9 K. Ando, T. Wada, M. Okumura and H. Sumida, *Org. Lett.*, 2015, **17**, 6026–6029.
10 M. Yoshikawa, R. Wakabayashi, M. Tamai and K. Kuroda, *New J. Chem.*, 2014, **38**, 5362–5368.
11 S. E. Denmark and J. D. Baird, *Tetrahedron*, 2009, **65**, 3120–3129.
12 R. Wakabayashi, K. Kawahara and K. Kuroda, *Angew. Chem. Int. Ed.*, 2010, **49**, 5273–5277.
13 S. Roesner and S. L. Buchwald, *Angew. Chem. Int. Ed.*, 2016, **55**, 10463–10467.
